# Phosphine Coordination to Ce, Nd, Np, and Pu in ^Ar^BiAMP*^t^* ^Bu^‐Supported Complexes and Their ^2^H and ^13^C Isotopologues

**DOI:** 10.1002/advs.78113

**Published:** 2026-10-04

**Authors:** Novan A. G. Gray, Yury Minko, Brenna K. Cashman, R. Joseph Lastowski, Adam R. Altenhof, Harris E. Mason, Daniel J. Lussier, Frank MacGregor, Andrew J. Gaunt, Aaron M. Tondreau

**Affiliations:** ^1^ Chemistry Division, Inorganic, Isotope, and Actinide Chemistry Los Alamos National Laboratory Los Alamos New Mexico USA; ^2^ Geothermal Energy & Geofluids Group ETH Zurich Zurich Switzerland

**Keywords:** actinides, aminophosphine, chelate, isotopic Labelling, lanthanides

## Abstract

^13^C‐ and ^2^H‐enriched bis((3,5‐dimethylphenyl)aminomethyl)*^tert^* butylphosphine (the natural abundance (NA) ligand **^Ar^BiAMP*^t^* ^Bu^**; the ^2^H‐ and ^13^C‐labelled analogues are denoted as ***d*_4_‐^Ar^BiAMP*^t^* ^Bu^** and **^13^C_2_‐^Ar^BiAMP*^t^* ^Bu^**, respectively), containing two secondary amines and a tertiary phosphine, was synthesized using a Kabachnik–Fields protocol. The coordination chemistry of the ligand isotopologues (including the NA derivative) was investigated with the paramagnetic lanthanides Ce and Nd and the paramagnetic actinide elements Np and Pu, furnishing **MI(^Ar^BiAMP*^t^* ^Bu^)(thf)_3_** (M = Ce(**1**), Nd(**2**), Np(**3**), Pu(**4**)), **MI(*d*_4_‐^Ar^BiAMP*^t^* ^Bu^)(thf)_3_** (M = Ce(**^2^H‐1**), Nd(**^2^H‐2**)) and **MI(^13^C_2_‐^Ar^BiAMP*^t^* ^Bu^)(thf)_3_** (M = Ce(**^13^C‐1**), Nd(**^13^C‐2**), Np(**^13^C‐3**), Pu(**^13^C‐4**)); each complex bears the metal in the 3+ oxidation state chelated by the amide groups and phosphorus atom of the ^Ar^BiAMP*^t^* ^Bu^ ligand. Multinuclear (^1^H, ^31^P, ^13^C, and ^2^H) NMR characterization of the complexes revealed wide windows of paramagnetically shifted resonances; the isotopically enriched derivatives allowed for the unambiguous identification of the methylene environments, highlighting the utility of the **^Ar^BiAMP*^t^* ^Bu^** ligands for providing rich spectroscopic signatures of paramagnetic *f*‐element coordination complexes. A combination of single‐crystal X‐ray diffraction and UV‐vis‐NIR spectroscopic techniques was also employed for the characterization of the compounds. Actinide complexes **3** and **4** possess rare examples of molecular transuranium element–phosphorus bonds and the first examples of transuranium element–phosphine interactions.

## Graphical abstract

A dianionic ligand designed to encourage metal‐phosphine coordination—bis((3,5‐dimethylphenyl)amidomethyl)*^tert^* butylphosphine (**^Ar^BiAMP*^t^* ^Bu^**)—was employed in the synthesis of paramagnetic complexes of Ce, Nd, Np, and Pu, producing **MI(^Ar^BiAMP*^t^* ^Bu^)(thf)_3_**. Isotopic labelling of the ligand with ^2^H and ^13^C, along with variable‐temperature NMR experiments, allowed for assignment of the NMR spectra. These are the first examples of Pu–phosphine and Np–phosphine interactions.

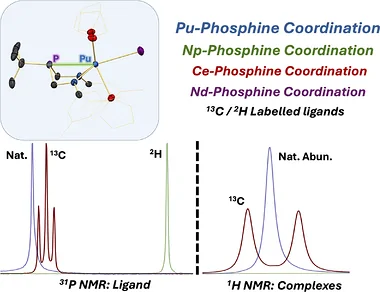

## 1 Introduction

Exploration into the fundamental behavior of the *f*‐elements continues to inform practical applications relevant to production of medical isotopes, separations science, and nuclear fuel waste remediation [1, 2, 3]. Generally, the hard electropositive *f*‐block metals preferentially bind to hard, oxygen‐based ligands over softer pnictogen donors such as nitrogen and phosphorus [4, 5]. However, the early actinide metals (U → Pu) exhibit a greater affinity for binding softer donors in comparison with the lanthanides [6, 7]. Rationale for this disparity in coordination behavior is supported by established differences in their physical properties: 4*f*‐metals tend to display core‐like orbital characteristics, while the 5*f*‐metals display modest but increased covalency in bonding due to the involvement of 5*f* and 6*d* orbitals [8, 9, 10, 11, 12]. These subtle differences in metal–ligand bonding between the actinide and lanthanide metals guide the development of application‐driven fields such as An/Ln separations and prompt fundamental investigations into actinide–ligand bonding properties.

While soft phosphorus donors generally coordinate *f*‐elements less favorably than nitrogen donors, literature reports include examples of complexes featuring phosphide, phosphinidene, and tertiary phosphine coordination to lanthanide and actinide metals [13, 14, 15, 16, 17, 18, 19, 20, 21]. While most lanthanides have demonstrated the ability to coordinate neutral phosphine donor ligands [22, 23, 24, 25, 26, 27, 28, 29, 30], such complexes of the actinide elements have been limited to uranium and thorium [31, 32, 33, 34, 35, 36, 37]. One example described a nearly 0.1 Å bond length contraction of U‐P (2.988(6) Å) versus the Ce‐P (3.086(3) Å) bond in homologous complexes, signifying a stronger interaction between the uranium and phosphine [32]. Such results encourage additional studies to investigate the bonding properties of actinides using phosphine‐based supporting ligands. Phosphorus donor complexes coordinating the transuranium elements are limited, with the phospholide‐supported neptunium and plutonium complexes [An(η^5^‐PC_4_Me_4_)_2_(µ‐η^1^:η^6^‐CH_2_Ph)_2_K(η^6^‐arene)] (An = Np, Pu; arene = benzene, toluene (Np only)) representing the existing examples of crystallographically authenticated molecular transuranium element–phosphorus bonds [38].

Substantial work has been presented in the literature exploring bifunctional N,P‐based ligands with *f*‐block metals [39, 40, 41, 42, 43, 44, 45, 46, 47, 48]. Our interests in exploring the fundamental aspects of soft donor ligand–*f*‐block element interactions inspired the development of **^Ar^BiAMP*^t^* ^Bu^**, (**^Ar^BiAMP*^t^* ^Bu^** = bis((3,5‐dimethylphenyl)amidomethyl)*^tert^* butylphosphine), a dianionic ligand designed to encourage phosphorus coordination to a metal center using the anionic amides as anchoring groups [49]. The resultant six‐membered metallacycle is hypothesized to adopt variable coordination motifs with respect to phosphorus — either bonding or non‐bonding — and to allow the degree of M–P interaction to be tuned through manipulation of the metal coordination sphere.

Our prior report focused on the interaction between the phosphorus atom of the ligand and the diamagnetic rare‐earth metals Y, La, and Lu [49]. With this report we sought to explore the installation of **^Ar^BiAMP*^t^* ^Bu^** on paramagnetic *f*‐block metals and gather the resultant NMR data of those coordination complexes. The paucity of established phosphine interactions with transuranium elements further encouraged our adaptation to neptunium and plutonium. Additionally, the extension of the **^Ar^BiAMP*^t^* ^Bu^** to the actinide elements was intended to facilitate comparison of the observed interactions between the 4*f*‐ and 5*f*‐metals and the phosphorus center. Of particular interest is exploration of the paramagnetic shifting of the phosphorus and methylene signals observed via NMR spectroscopy, both of which are likely to be affected due to their proximity to the paramagnetic metal centers.

This report builds upon the synthesis of **^Ar^BiAMP*^t^* ^Bu^** to include stable isotope labelling at the methylene fragment of the ligand (Scheme 1, Top). Further studies of the ligand synthesis include manipulation of stoichiometry to favor either **^Ar^BiAMP*^t^* ^Bu^**, **^Ar^BiAMP*^t^* ^Bu^‐CH_2_**, or ***^t^* ^Bu^P_2_N_2_^Ar^**. New metal complexes of natural abundance (NA), ^2^H, and ^13^C isotopologues of **^Ar^BiAMP*^t^* ^Bu^** are also presented, with the coordination chemistry of paramagnetic lanthanide and actinide metals Ce, Nd, Np, and Pu described (Scheme 1, Bottom). Spectroscopic investigations using solution NMR techniques highlight the rich array of NMR handles that **^Ar^BiAMP*^t^* ^Bu^** presents for coordination complexes. Reported paramagnetic species show wide chemical shift windows for the various nuclei, creating distinct signatures for all complexes in ^1^H, ^2^H, ^13^C, and ^31^P NMR studies. Isolation of the Np and Pu complexes provide the first crystallographically authenticated transuranium element–phosphine interactions.

**SCHEME 1:**
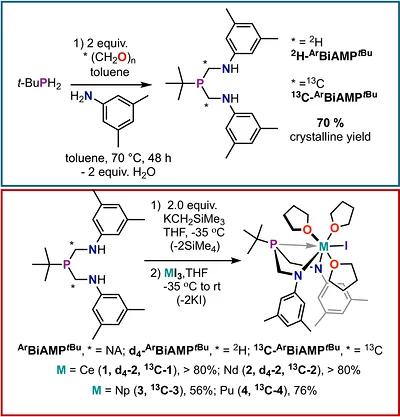
General schemes for the synthesis of the **^Ar^BiAMP*^t^* ^Bu^** isotopologues (Top) and the installation of in situ deprotonated **^Ar^BiAMP*^t^* ^Bu^** onto Ce, Nd, Pu, and Np (Bottom).

## 2 Results and Discussion

### 2.1 Developments for the Synthesis of ^Ar^BiAMP*^t^* ^Bu^ Isotopologues

Investigation of the three‐component, one‐pot Kabachnik–Fields reaction employed in the **^Ar^BiAMP*^t^* ^Bu^** synthesis aimed to provide further understanding of the observed product distribution and a more holistic description of the formation of **^Ar^BiAMP*^t^* ^Bu^** and its cyclic byproducts [49]. It was previously hypothesized that precise control of stoichiometry was responsible for isolation of **^Ar^BiAMP*^t^* ^Bu^** [49, 50, 51, 52, 53, 54]. Consistent with that hypothesis, stoichiometrically modified procedures were used to obtain both the established side product **^Ar^BiAMP*^t^* ^Bu^‐CH_2_** as well as a previously unidentified insoluble side product, 3,7‐di‐*tert*‐butyl‐1,5‐bis(3,5‐dimethylphenyl)‐1,5‐diaza‐3,7‐diphosphacyclooctane (***^t^* ^Bu^P_2_N_2_^Ar^**) (Table 1, Left).

**TABLE 1:** (Left) The employed reaction stoichiometries of the Kabachnik–Fields reaction to preferentially generate a family of related products. (Right) The expanded asymmetric unit cell of the solid‐state structure of ***^t^* ^Bu^P_2_N_2_^Ar^** is presented at 50% probability ellipsoids with hydrogen atoms omitted for clarity. Judicious alteration of stoichiometry of the three‐component reaction has allowed for efficient isolation of select PN‐based fragments.

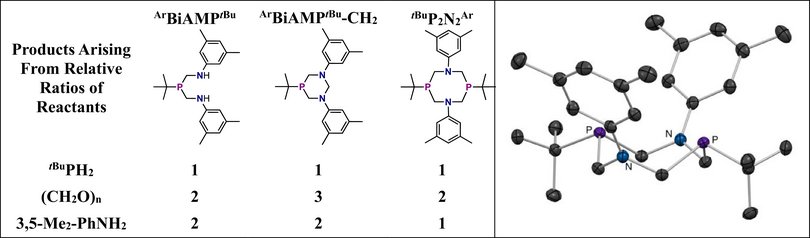

Synthesis of **^Ar^BiAMP*^t^* ^Bu^‐CH_2_** was pursued according to a stoichiometrically modified procedure described for **^Ar^BiAMP*^t^* ^Bu^**. An additional equivalent of paraformaldehyde was added to the reaction mixture following the addition of aniline, and the respective final stoichiometry was 1:2:3 for phosphine, aniline, and paraformaldehyde, providing **^Ar^BiAMP*^t^* ^Bu^‐CH_2_** in moderate crystalline yields; the product was spectroscopically indistinguishable from the sample reported previously [49].

Deliberate synthesis of ***^t^* ^Bu^P_2_N_2_^Ar^** was performed by employing a 1:1:2 stoichiometry respectively of phosphine, aniline and paraformaldehyde, providing the desired compound ***^t^* ^Bu^P_2_N_2_^Ar^** in good yields, as confirmed by NMR spectroscopy and solid‐state X‐ray characterization (Table 1, Right). The acquired ^1^H NMR spectrum showed the symmetry‐expected number of resonances consistent with the desired molecule (Figures S15–S18). One resonance ascribed to the *^t^* Bu group was found as a doublet (*J*_PH_ = 11 Hz) centered at δ 1.28 ppm integrating to 18 protons. The two distinct aromatic C–H groups (*ortho* and *para*) were respectively centered at δ 6.31 and δ 6.30 ppm; the aryl methyl resonance was centered at δ 2.21 ppm and integrated to 12 protons. Finally, two methylene resonances were found as a pair of doublets of doublets centered at δ 4.39 (*J*_1_ = 14.8, *J*_2_ = 11.5 Hz) and δ 3.47 ppm (*J*_1_ = 14.8, *J*_2_ = 4.9 Hz) integrating to four protons each.

Crystals suitable for single‐crystal X‐ray diffraction (SC‐XRD) studies were collected from dissolution of isolated product in hot toluene followed by slow cooling of the solution, yielding thin needles. The SC‐XRD data were solved in the monoclinic space group *C*2*c*, and the asymmetric unit cell comprised one half of the complex. The nitrogen atom is observed as planar, with the sum of the angles surrounding nitrogen found at 359.92°. The phosphorus remains pyramidal with an accessible lone pair and the sum of the angles about the phosphorus substituents found at 300.41°.

The previous investigation into the **^Ar^BiAMP*^t^* ^Bu^** system was expanded to include an NMR study of the organic proligand and the related ^2^H and ^13^C isotopologues. NMR experiments on the free ligands were performed in THF‐*d*_8_ to maintain consistency with those of the THF‐stable metal complexes of this study **MI(^Ar^BiAMP*^t^* ^Bu^)(thf)_3_** (M = Ce(**1**), Nd(**2**), Np(**3**), Pu(**4**)). There are two clearly resolved sets of methylene resonances corresponding to the axial, H_ax_, and equatorial, H_eq_, protons. Solution NMR experiments performed previously showed a complex coupling pattern, and to begin this study, additional experiments were performed to deconvolute the unexpected pattern. The resonances were well modelled using simulations in SPINACH [55] as an AA′BB′X system (Figure 1a) where X is the phosphorus atom. Efforts to decouple homonuclear H‐H coupling were performed using PSYCHE experiments (Pure Shift Yielded by Chirp Excitation) [56, 57], employing a previously established sequence [58]. Analysis of the data reveals *J*_HP_ = 4.7 Hz for the δ 3.41 ppm shift (Figure 1b). Collection and analysis of ^1^H{^31^P} NMR spectral data further confirmed that the shift at δ 3.41 ppm displays coupling of *J*_HP_ = 4.7 Hz and the shift at δ 3.29 ppm does not display measurable *J*_HP_ coupling (Figure 1c).

**FIGURE 1:**
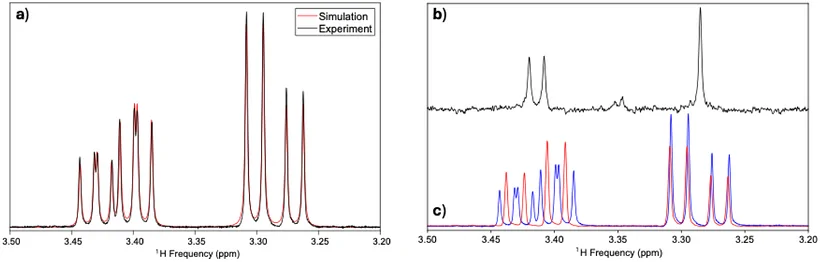
(a) Simulation (red trace) and experiment (black trace) of the methylene protons observed in THF‐*d*_8_ of **^Ar^BiAMP*^t^* ^Bu^** at room temperature are provided. (b) An expansion of the PSYCHE ^1^H‐^1^H homonuclear decoupled NMR spectrum of **^Ar^BiAMP*^t^* ^Bu^** highlighting the *J*_PH_ = 4.7 Hz for the resonance at 𝛿 3.41 ppm. (c) The ^1^H{^31^P} NMR spectrum of **^Ar^BiAMP*^t^* ^Bu^** (red trace) overlaid with the non‐decoupled ^1^H NMR resonance of **^Ar^BiAMP*^t^* ^Bu^** (blue trace).

Over time in THF‐*d*_8_, the non‐first‐order coupling pattern is replaced with a simpler pattern in the methylene region (Figures S53, S54), with changes associated with unintended reactivity observed in the spectrum. Two resonances attributed to the diastereotopic methylene protons were found at δ 3.29 ppm (*J*_HH_ = 12.9 Hz) and a doublet of doublets centered at δ 3.41 ppm (*J*_HH_ = 12.9 Hz; *J*_HP_ = 4.7 Hz). We rationalize this as a change in molecular conformation, where **^Ar^BiAMP*^t^* ^Bu^** is found in a rigid pseudo‐cyclohexane conformation arising from intramolecular hydrogen bonding between the diamino groups that forms an N–H⟵N pattern (Figure S54), as observed in the solid‐state structure [49]. We posit that this conformation is retained in non‐coordinating solvents such as benzene. However, THF may disrupt the hydrogen bonding and cyclohexane‐like arrangement, eliminating the magnetic inequivalence from the coupling pattern. Once the N–H moieties can freely rotate, the formation of **^Ar^BiAMP*^t^* ^Bu^‐CH_2_** as observed by ^31^P{^1^H} NMR spectroscopy suggests that the amines are nucleophilic and further react and degrade.

The deuterated isotopologue ***d*_4_‐^Ar^BiAMP*^t^* ^Bu^** was synthesized using *d*_2_‐paraformaldehyde according to the procedure described for the NA material. Analysis of the resultant crystalline material suggested successful incorporation of the desired deuterium label; the ^1^H NMR spectrum showed a spectrum similar to that of the NA complex, but resonances for the diastereotopic methylene protons (located at δ 3.38 and δ 3.26 ppm) were absent. Expectedly, the ^2^H NMR spectrum of the product showed broadened resonances at δ 3.31 and δ 3.19 ppm. The high concentration used for the ^2^H NMR solution and instability of ***d*_4_‐^Ar^BiAMP*^t^* ^Bu^** in THF gave rise to decomposition over the course of the collection, and the resulting spectrum contained additional ^2^H resonances (Figure S7). The ^13^C{^1^H} spectrum displayed the methylene carbon as a complex multiplet centered at δ 40.1 ppm, though the resonances were poorly resolved from the baseline. The ^31^P{^1^H} resonance of the *d*_4_‐isotopologue is shifted by 1.5 ppm in comparison with the NA compound (δ ‐9.8 vs δ ‐8.4 ppm, respectively), a larger‐than‐expected secondary isotope effect. We propose that the effects of the deuterium labelling are cumulative, contributing to the large observed change in chemical shift [59].

The synthesis of the ^13^C‐labelled isotopologue was performed using an analogous reaction employing ^13^C_2_‐paraformaldehyde, producing similar yields and purity to previous syntheses. Isolation of the crystalline product and analysis using NMR spectroscopy revealed formation of the desired doubly labelled **^13^C_2_‐^Ar^BiAMP*^t^* ^Bu^**. Analysis of the ^1^H NMR spectrum revealed an additional large coupling (*J*_CH_ = 136 Hz) for the methylene protons in addition to the complex coupling scheme observed previously; the spectrum was modelled as an AA′BB′MX system, with M representing the ^13^C‐labelled carbon atom. As expected, the ^13^C NMR experiments revealed a prominent doublet (δ 40.7 ppm, *J*_CP_ = 13.4 Hz) attributed to the labelled methylene groups. The ^31^P resonance for **^13^C_2_‐^Ar^BiAMP*^t^* ^Bu^** appears as a triplet (*J*_PC_ = 13.4 Hz) and is shifted by 0.2 ppm as compared to **^Ar^BiAMP*^t^* ^Bu^** (δ ‐8.6 vs δ ‐8.4 ppm, respectively).

### 2.2 Synthesis and Solid‐State Characterization of Natural Abundance (NA), ^2^H, and ^13^C Isotopologues of MI(^Ar^BiAMP*^t^* ^Bu^)(thf)_3_ (M = Ce(1), Nd(2), Np(3), Pu(4))

Installation of the NA **^Ar^BiAMP*^t^* ^Bu^** ligand on the lanthanide metals Ce and Nd was achieved via salt metathesis as previously established [49]. Following in situ deprotonation of **^Ar^BiAMP*^t^* ^Bu^**, the cold (−35°C) ligand‐THF solution was added to a cold (−35°C) stirring slurry of the appropriate MI_3_(thf)_4_ starting materials (M = Ce, Nd). The products following work‐up, **MI(^Ar^BiAMP*^t^* ^Bu^)(thf)_3_** (M = Ce, **1;** M = Nd, **2**), were isolated as large colorless blocks for **1** in 82% yield, and blue‐violet blocks for **2** in 84% yield. Acquisition and analysis of SC‐XRD data arising from single crystals of **1** (Figure 2, left) and **2** confirmed assignment of the desired seven‐coordinate complexes — found in distorted capped trigonal prism geometry (vide infra) — that are isostructural and isomorphous to the Y, La, and Lu analogues [49]. The structures of **1** and **2** were solved in the orthorhombic space group *P*2_1_2_1_2_1_ and the asymmetric unit cell contains a single molecule of the title compounds. The M–X (X = N, O, I) distances in cerium complex **1** were each found to be ∼0.03 Å longer than the corresponding distances in neodymium complex **2**, consistent with the difference in the trivalent ionic radii of neodymium vs. cerium (0.03 Å) [60]. The M–P distances in **1** and **2** were measured at 3.177(1) and 3.146(1) Å, respectively, well within the sum of the van der Waals radii of the respective element pairs (4.78 Å for **1**; 4.85 Å for **2**) [61] and approaching the sum of the covalent radii of the same (ΣCov_Ce–P_ = 3.11 Å; ΣCov_Nd–P_ = 3.08 Å) [62].

**FIGURE 2:**
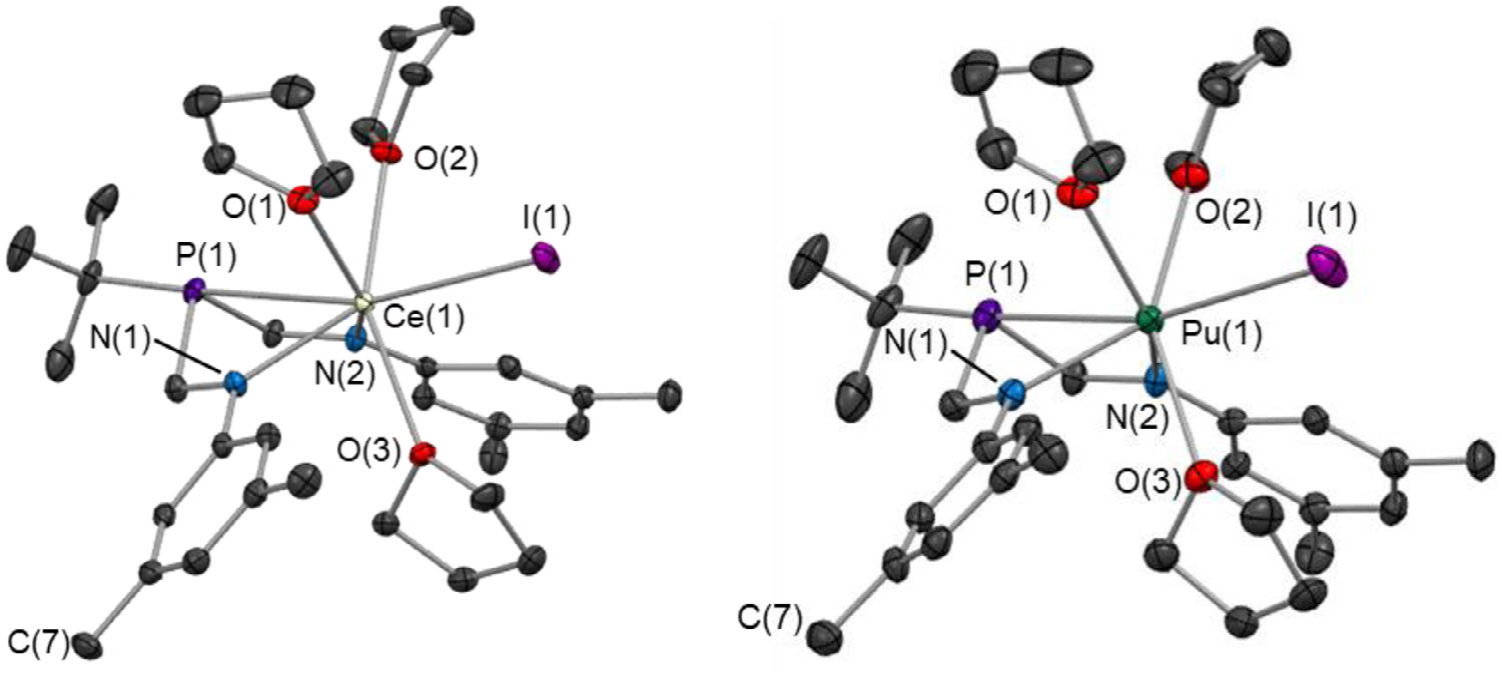
The X‐ray structures of complexes **1** and **4** are presented at 50% probability ellipsoids with hydrogen atoms omitted for clarity; complexes **2** and **3** are isostructural.

With the successful application of the **^Ar^BiAMP*^t^* ^Bu^** ligand to a range of rare‐earth metals, extension of its chemistry to the actinide elements was pursued. The NA neptunium and plutonium complexes **AnI(^Ar^BiAMP*^t^* ^Bu^)(thf)_3_** (An = Np(**3**), Pu(**4**) (Figure 2, right)) and their ^13^C isotopologues **AnI(^13^C_2_‐^Ar^BiAMP*^t^* ^Bu^)(thf)_3_** (An = Np(**^13^C‐3**), Pu(**^13^C‐4**)) were prepared following analogous procedures to those performed for **1** and **2**. Although the deuterated isotopologues of complexes **1** and **2** provided a valuable handle for the identification of the methylene groups, the ^13^C analogues were found to provide equally informative data without requiring an additional spectroscopic experiment (^2^H NMR spectroscopy). Consequently, the **^13^C_2_‐^Ar^BiAMP*^t^* ^Bu^** ligand was selected as the ideal candidate for coordination chemistry and spectroscopic investigations with the transuranium actinides where experiments are material‐limited due to safety and operational factors.

The neptunium product **NpI(^Ar^BiAMP*^t^* ^Bu^)(thf)_3_** was isolated as dark red crystals in 56% yield (**3**/**^13^C‐3**; sometimes as a waxy solid), while the plutonium congener **PuI(^Ar^BiAMP*^t^* ^Bu^)(thf)_3_** was isolated as blue‐green crystals (**4**/**^13^C‐4**) in 76% yield. Analyses of SC‐XRD data obtained from suitable single crystals of **3** and **4** revealed the target seven‐coordinate complexes, which were found isostructural and isomorphous to the lanthanide analogues. The solid‐state structures of **3** and **4** featured the actinide metals in the 3+ oxidation state with κ^3^‐coordination of the **^Ar^BiAMP*^t^* ^Bu^** ligand to the metal centers through the two nitrogen donors and the central phosphorus donor. Three THF ligands and an iodide ligand complete the metal coordination sphere. The An–I and An–O_(THF)_ distances in complexes **3** and **4** are within the reported ranges of crystallographically characterized Np(III) and Pu(III) complexes [63]. However, the An–N distances in **3** (2.352(9) and 2.358(9) Å) and **4** (2.329(6) and 2.332(6) Å) are notably shorter than previously reported Np(III) and Pu(III) amido complexes bearing carbon‐based substituents at the nitrogen atom (2.447–2.496 Å for Np; 2.428 Å for Pu) [64], but are comparable to An(III) (An = Np, Pu) silylamido complexes (2.338(3) Å for Np [65], 2.315(10) Å for Pu) [66]. More notably, the Np–P and Pu–P linkages in **3** and **4** represent the second examples of crystallographically characterized molecular Np–phosphorus and Pu–phosphorus bonds, respectively, and provide the first crystallographically authenticated examples of transuranium element–phosphine interactions.

With reported phospholide complexes [An(η^5^‐PC_4_Me_4_)_2_(µ‐η^1^:η^6^‐CH_2_Ph)_2_K(η^6^‐arene)] (An = Np, Pu; arene = benzene, toluene (Np only)) [38] serving as the only literature examples of crystallographically authenticated molecular Np and Pu complexes bearing An–P bonds, the metal–phosphorus distances of these species were compared to those of **3** and **4**. The neptunium phospholide structures contain Np–P distances ranging from 2.9147(11) to 2.971(3) Å, while the plutonium analogue produced Pu–P distances of 2.9533(13) and 2.9816(14) Å [38]. These interactions are shorter than those presented for **3** (3.138(3) Å) and **4** (3.112(2) Å). However, an absence of a notable difference in M─P bond lengths of the reported phospholide‐supported complexes exists when comparing Ce to the Np or Pu congeners with that system [38].

In contrast to the Cp‐like motif of the phospholide ligand, **^Ar^BiAMP*^t^* ^Bu^** maintains a flexible, unconstrained phosphine moiety that can freely coordinate to, or remain distal, from the coordinated metal centers. For complexes **1–4**, the metal–phosphorus distances exist well within the sum of the van der Waals radii [61] of the respective elements and are approaching the sum of the covalent radii [62], suggesting the presence of genuine M–P interactions (Table 2).

**TABLE 2:** Comparison of the M–P distances in complexes **1–4** to the sum of the van der Waals radii and covalent radii of the respective metal/phosphorus pairs.

Complex	M–P Distance (Å)	ΣvdW_(M–P)_ (Å) [61]	ΣCov_(M–P)_ (Å) [62]
**1**	3.1773(7)	4.78	3.11
**2**	3.1461(7)	4.85	3.08
**3**	3.138(3)	4.72	2.97
**4**	3.112(2)	4.71	2.94

Attempts to generate the analogous U(III) complex following the same procedure used for **1–4** led to the exclusive identification of a U(IV) product bearing two ^Ar^BiAMP*^t^* ^Bu^ ligands and a THF ligand at the metal. These and additional results focusing on M^4+^ starting materials will be elaborated upon further in a future report. Adaptation of this scope of chemistry to Np and Pu allows for the study of An(III) bonding and subsequent comparison to Ln(III) in a rare situation where Th and U are unable to provide synthetic access to the needed comparison points, necessitating the use of transuranium elements. No evidence for the generation of Ce^4+^ species was observed at any time during the synthesis of **1** in this study.

UV‐vis‐NIR analyses were performed on compounds **2–4**, for which the solid‐ and solution‐state spectra were in good agreement for each respective complex, supporting the identity and purity of the bulk products. Complex **2** shows the multiple electronic transitions expected for a Nd^3+^ ion (Figures S55, S56). For **3** and **4**, the vis‐NIR region bears a series of low‐intensity transitions that were observed in both complexes, arising from Laporte‐forbidden *f–f* transitions, consistent with the Np(III) and Pu(III) ions in **3** and **4**, respectively (Figure 3 and Figures S57–S64). In the UV‐vis region, relatively intense transitions were recorded at 344 nm (in **3**) and 353 nm (in **4**), likely attributed to ligand‐centered transitions (i.e., π→π*). Complex **4** displays sharp and relatively weak absorbance bands that are consistent with established Pu^3+^ ions; the bonding description of the complex (vide infra) is likely dominated by the 5*f* orbitals with small amounts of 6*d* orbital participation that may be the origin of the stronger intensity absorptions in the high‐energy wavelengths below 500 nm.

**FIGURE 3:**
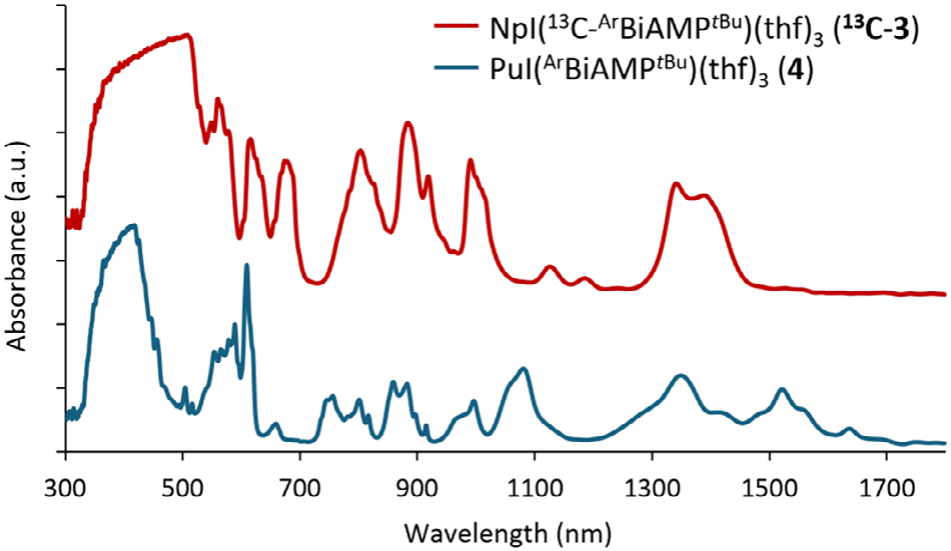
An overlay of the solid‐state UV‐vis‐NIR data for complexes **3** and **4** is presented with an offset on the y‐axis. The abnormal slope observed for **^13^C‐3** is an artefact arising from detector saturation.

The M–P distances in complexes **1** and **2** along with those of the previously reported isomorphous lanthanide compounds **MI(^Ar^BiAMP*^t^* ^Bu^)(thf)_3_** (M = La, Lu) and additional praseodymium and thulium analogues [67] were plotted against the Shannon 6‐coordinate trivalent ionic radii of the respective metals [68], revealing a linear relationship (R^2^ = 0.98; Figure 4). This analysis points to the presence of lanthanide–phosphine interactions which are primarily electrostatic in nature, with negligible changes in strength as a function of the lanthanide metal identity. Interestingly, inclusion of the An–P distances from neptunium and plutonium complexes **3** and **4** into the regression analysis results in a notable reduction of linearity (R^2^ decreases from 0.98 to 0.76; Figure S65), with An–P distances that are contracted relative to their respective near‐isoradial lanthanide surrogates (Figure S65). In contrast to the phospholide complexes [38], notable bond contractions are observed with **3** and **4** as compared to lanthanide analogues. The Np–P bond is contracted compared to its corresponding isoradial surrogate (0.039 Å compared to Ce), while the Pu–P bond is shorter than its near‐isoradial lanthanide surrogate complexes by 0.065 Å (Ce) and 0.042 Å (Pr). These data point to the presence of potentially stronger interactions between Np and Pu and the phosphine donor compared to the lanthanides.

**FIGURE 4:**
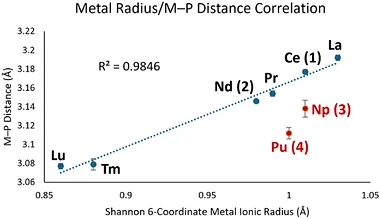
Correlation plot of the M–P distances vs. 6‐coordinate trivalent metal ionic radii for complexes **1–4** and **MI(^Ar^BiAMP^tBu^)(thf)_3_** (M = La, Pr, Tm, Lu). Actinide complexes **3** (Np) and **4** (Pu) are denoted as red points on the graph. Error bars are shown to 3σ. Linear regression analysis (R^2^ value) reflects only the lanthanide members denoted as blue points on the plot (see Figures S66–S71 for other linear regression plots).

The radially extended 5*f* and 6*d* orbital manifolds of the actinides could potentially enhance covalent contributions to bonding in the actinide analogues over those of the lanthanides, resulting in a stronger interaction. The capped trigonal prism geometry [69, 70] places the z‐axis orthogonal to the tetragonal plane formed between the amide ligands and two coordinated THF molecules; the “cap” of the geometry is then the phosphorus atom, which lies ∼20° off normal to the z‐axis (Figure 5, left). The geometry of the phosphorus atom is reminiscent of both seesaw and tetrahedral geometries; however, the ^tBu^C–P–M angles are nearly linear for all complexes (Figure S74), with an angle of 175.0105(10)° in **4**. The angle suggests that the lone pair resides in a predominantly *p*‐based orbital that is directed at the metal center (Figure 5). Due to the constraints of the chelate, the geometry of the phosphorus atom would bear limited orbital overlap between the phosphine *p*‐orbital and the orbitals of the metal center (Figure 5, right). This bonding arrangement necessitates a symmetry‐allowed 𝛔‐bonding orbital from the metal. In the case of the actinides Np and Pu, the orbital would either be the empty 6*dz* ^2^ or 5*fz* ^3^ orbitals. This described arrangement induces the dative P→M interaction while also allowing a ligand‐to‐metal bonding interaction that potentially participates with both 6*d*z^2^ and 5*f*z^3^ orbitals [6, 7, 8, 9, 12, 71]. Given the narrow UV‐vis‐NIR absorptions observed in the data for both **3** and **4**, it is likely that the metal electrons reside predominantly in the 5*f* shell, and bonding interactions that employ the 6*d* orbitals are minor. The same bonding interaction concerning the 4*f* elements will be more limited due to the low‐energy, core‐like orbitals. Additional bonding arrangements and discussion are presented in Section S.2.4.

**FIGURE 5:**
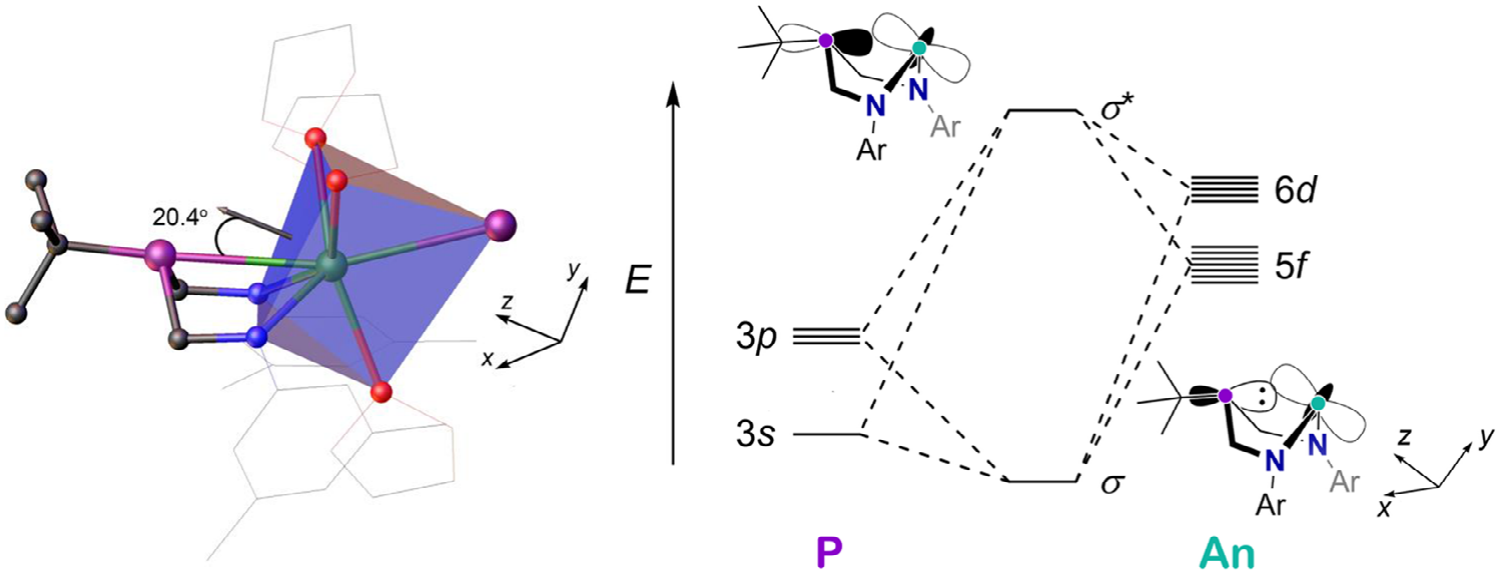
A truncated MO diagram focusing on the P–An interaction between the phosphorus and actinide orbitals; geometric constraints limit the idealized linear overlap in the bonding orbitals.

Calculations are being pursued to compare the bonding descriptions between the 4*f* and 5*f* elements, as well as comparisons exploring the trends in An–P bonding of the 5*f* elements when comparing Np → Am. Additionally, we are exploring the solution dynamics of **^Ar^BiAMP*^t^* ^Bu^**‐supported coordination complexes using NMR techniques to establish energetic differences in bonding between the phosphorus atom of **^Ar^BiAMP*^t^* ^Bu^** and a series of metal centers. These ongoing studies will provide physical data from NMR studies with which to validate the computationally derived energetic landscapes of the An–P coordination; results of these studies will be reported when appropriate.

### 2.3 NMR Spectroscopy Characterization and Discussion of Natural Abundance (NA), ^2^H, and ^13^C Isotopologues of MI(^Ar^BiAMP*^t^* ^Bu^)(thf)_3_ (M = Ce(1), Nd(2), Np(3), Pu(4))

Analyses of **1** and **2** using ^1^H, ^13^C, and ^31^P NMR techniques were performed and the resulting spectra are presented in the Supporting Information. The ^1^H NMR spectra comprise broad resonances between δ 27.7 and δ −3.5 ppm (**1**) and δ 41.1 and δ −3.8 ppm (**2**), which support the expected paramagnetic designation of the complexes. The solution ^1^H NMR data for compounds **1** and **2** are consistent with their crystal structures and rapid rotation of aryl groups around N–C*_ipso_* bonds (Table 3) except that the *o*‐aryl resonance for compound **1** is unobservable at room temperature likely due to broadening from a combination of coalescence effects of arene rotation and paramagnetic effects of the Ce^3+^ ion; the *o*‐aryl resonance for compound **2** is observed as a broadened (FWHM = 1750 Hz) resonance at δ 8.5 ppm. The ^31^P resonance was observed at δ ‐0.9 ppm (**1**) or δ 287.8 ppm (**2**), shifted from the free‐ligand value of δ −8.4 ppm.

**TABLE 3:** ^1^H, ^2^H, ^13^C, and ^31^P chemical shift values (ppm) with relevant coupling provided for the methylene (CH_2_/CD_2_) groups and phosphorus atoms in complexes **1**, **^2^H‐1**, **^13^C‐1**, **2**, **^2^H‐2**, **^13^C‐2**, **^13^C‐3**, **^13^C‐4**. Data recorded at 25°C in THF‐*d*_8_ unless otherwise noted.

Complex	*δ* ^1^H NMR (C *H*_2_)	*δ* ^2^H NMR (C *D*_2_)	*δ* ^31^P NMR	*δ* ^13^C NMR (*C* H_2_/*C* D_2_)
**^Ar^BiAMP*^t^* ^Bu^**	3.41, 3.29	—	−8.4	40.8
**1**	27.7, 0.1	—	−0.9	—
**^2^H‐1**	—	27.3 (*J*_P–D_ 12 Hz), 0.2	−3.3	—
**^13^C‐1**	26.7 (*J*_C–H_ 128 Hz), 0.1 (*J*_C–H_ 131 Hz)	—	−1.0	30.9–29.6
**2**	41.1, 17.4	—	287.8	—
**^2^H‐2**	—	41.0, 17.5	279.8	—
**^13^C‐2**	41.0 (*J*_C–H_ 134 Hz), 17.5 (*J*_C–H_ 132 Hz)	—	286.8	1.9
**^13^C‐3**	15.9 (*J*_C–H_ 139 Hz), 15.4 (*J*_C–H_ 131 Hz)	—	231.1	−154.2
**^13^C‐4**	5.6 (*J*_C–H_ 125 Hz), 3.3 (*J*_C–H_ 121 Hz) ^a)^	—	−35.0	8.3

^a)^ Data recorded at 35°C.

Analysis of deuterated isotopologues **^2^H‐1** and **^2^H‐2** was performed with ^1^H, ^2^H, and ^31^P NMR techniques. As expected, the ^1^H NMR spectra lacked methylene resonances while the ^2^H NMR spectra contained new resonances at δ 27.3 and δ 0.2 ppm for **^2^H‐1** and at δ 41.0 and δ 17.5 ppm for **^2^H‐2** attributable to the methylene environments (Table 2); *J*‐coupling to the ^31^P nucleus is only observable on the ^2^H resonance of the Ce compound **^2^H‐1** at δ 27.3 ppm and manifests as a doublet (*J*_P‐D_ = 12 Hz). Analysis of the ^31^P{^1^H} spectrum of **^2^H‐1** showed a singlet resonance at δ ‐3.3 ppm, whereas in **^2^H‐2** the resonance is found at δ 279.8 ppm. These shifts from the NA values are consistent with the chemical shift differences observed between ***d*_4_‐^Ar^BiAMP*^t^* ^Bu^** and **^Ar^BiAMP*^t^* ^Bu^** arising from secondary isotope effects.

The ^1^H NMR spectra of ^13^C‐labelled isotopologues **^13^C‐1** and **^13^C‐2** contain the expected chemical shifts (Table 3). The resonances attributed to the methylene protons were observed as doublets centered at δ 26.7 (*J*_C‐H_ = 128 Hz) and 0.1 (*J*_C‐H_ = 131 Hz) ppm for **^13^C‐1** and at δ 41.0 (*J*_C‐H_ = 134 Hz) and 17.5 (*J*_C‐H_ = 132 Hz) ppm for **^13^C‐2** due to coupling with the enriched ^13^C nuclei; these values are appropriate for *sp* ^3^ C─H bonds. The methylene ^13^C nuclei in **^13^C‐1** and **^13^C‐2** give rise to resonances centered at δ 30 and δ 1.9 ppm, respectively. The observed resonances in the ^31^P{^1^H} spectra for **^13^C‐1** (δ −1.0 ppm) and **^13^C‐2** (δ 286.8 ppm) are consistent with the other isotopologues with minor secondary isotope shifts observed.

The ^1^H NMR spectra of **^13^C‐3** and **^13^C‐4** display broad resonances between approximately δ 1 and δ 15 ppm and are consistent with the solid‐state structures elucidated by SC‐XRD. The methylene resonances in the ^1^H NMR spectrum of **^13^C‐3** were observed as sharp doublets at δ 15.9 (*J*_C‐H_ = 139 Hz) and δ 15.4 (*J*_C‐H_ = 131 Hz) ppm at room temperature that shifted to δ 14.6 and δ 13.8 at 50°C, while those for **^13^C‐4** were recorded as broad doublets (similar to what was observed for **^13^C‐1** and **^13^C‐2**) which overlapped with other resonances in the spectrum at room temperature (Figure S46). Upon heating to 35°C, the methylene signals for **^13^C‐4** shifted away from other overlapping resonances, clearly revealing the doublet peaks centered at δ 3.3 (*J*_C‐H_ = 121 Hz) and δ 5.6 ppm (*J*_C‐H_ = 125 Hz) (Figure S48). Additionally, heating of **^13^C‐3** to 50°C resulted in the sharpening of the aryl methyl and *p*‐aryl proton resonances at δ 2.0 and δ 7.7 ppm (Figure S43), respectively, which were formerly broadened into the baseline at room temperature (Figure S42).

The ^13^C{^1^H} spectra revealed a relatively sharp resonance at δ ‐154.2 ppm (in **^13^C‐3**) and a broad resonance at δ 8.3 ppm (in **^13^C‐4**), attributable to the labelled methylene carbons in each complex. The ^31^P{^1^H} NMR spectra for **^13^C‐3** and **^13^C‐4** reveal a single broad resonance at δ 231.1 ppm (in **^13^C‐3**) and δ ‐35.0 ppm (in **^13^C‐4**), appearing downfield and upfield of the resonance for the free ligand, respectively.

## 3 Conclusion

Further investigation into the Kabachnik–Fields reaction provided synthetic control to yield the related series of compounds **^Ar^BiAMP*^t^* ^Bu^**, **^Ar^BiAMP*^t^* ^Bu^‐CH_2_**, and ***^t^* ^Bu^P_2_N_2_^Ar^** in good yields. **^Ar^BiAMP*^t^* ^Bu^** was determined to be unstable, undergoing undesired reactivity in THF‐*d*_8_ despite stability in C_6_D_6_. This increased understanding of reaction conditions and stability allowed for the efficient synthesis of the stable isotope‐labelled ligands ***d*_4_‐^Ar^BiAMP*^t^* ^Bu^** and **^13^C_2_‐^Ar^BiAMP*^t^* ^Bu^**, which (along with the NA variant) were complexed to the paramagnetic *f*‐elements Ce, Nd, Np and Pu, furnishing **MI(^Ar^BiAMP*^t^* ^Bu^)(thf)_3_** (M = Ce (**1**), Nd (**2**), Np (**3**), Pu (**4**)), **MI(*d*_4_‐^Ar^BiAMP*^t^* ^Bu^)(thf)_3_** (M = Ce (**^2^H‐1**), Nd (**^2^H‐2**)) and **MI(^13^C_2_‐^Ar^BiAMP*^t^* ^Bu^)(thf)_3_** (M = Ce (**^13^C‐1**), Nd (**^13^C‐2**), Np (**^13^C‐3**), Pu (**^13^C‐4**)). The isotopically enriched complexes gave rise to diverse NMR spectra which were informative for the unambiguous identification of the methylene environments, demonstrating the use of the **^Ar^BiAMP*^t^* ^Bu^** framework for future studies of solution‐phase fluxional dynamics. High‐quality single‐crystal X‐ray diffraction data revealed a linear relationship between the metal–phosphorus bond lengths and metal ionic radii for lanthanide complexes **MI(^Ar^BiAMP*^t^* ^Bu^)(thf)_3_** (M = La, Ce, Pr, Nd, Tm, Lu), while the actinide species **AnI(^Ar^BiAMP*^t^* ^Bu^)(thf)_3_** (M = Np, Pu) demonstrated shorter An–P interactions relative to their near‐isoradial lanthanide counterparts. Furthermore, the X‐ray structures of complexes **3** and **4** represent not only the second examples of molecular complexes bearing transuranium–phosphorus interactions but also provide the first crystallographically authenticated examples of transuranium element–phosphine donor bonds.

## Author Contributions

**Novan A. G. Gray**: investigation, methodology, writing – review and editing, writing – original draft. **Yury Minko**: investigation, methodology, writing – review and editing. **Brenna K. Cashman**: investigation, writing – original draft, writing – review and editing, visualization. **R. Joseph Lastowski**: formal analysis, visualization, writing – review and editing, investigation. **Adam R. Altenhof**: formal analysis, resources, investigation, visualization, writing – review and editing. **Harris E. Mason**: formal analysis, resources, writing – review and editing. **Daniel J. Lussier**: resources, investigation, writing – review and editing. **Frank Macgregor**: resources, writing – review and editing. **Andrew J. Gaunt**: resources, supervision, funding acquisition, writing – review and editing. **Aaron M. Tondreau**: conceptualization, methodology, formal analysis, supervision, resources, project administration, visualization, writing – review and editing, writing – original draft, funding acquisition.

## Conflicts of Interest

The authors declare no conflicts of interest.

## Supporting Information

The data supporting this manuscript have been uploaded as part of the supplementary information (SI). The data in the SI include experimental descriptions, spectra, and X‐ray diffraction data for all complexes. CCDC deposition numbers (2527016–2527022) [72] contain the supplementary crystallographic data for this paper. The authors have cited additional references within the Supporting Information [73, 74, 75, 76, 77, 78, 79, 80].

## Supporting information




**Supporting File**: advs78113‐sup‐0001‐SuppMat.pdf.

## Data Availability

The data that supports the findings of this study are available in the supplementary material of this article.
