# Supplementary material for: Phosphine Coordination to Ce, Nd, Np, and Pu in ArBiAMP t Bu‐Supported Complexes and Their 2H and 13C Isotopologues
Source: Adv Sci (Weinh). 2026 Oct 4:e78113. Online ahead of print. doi: 10.1002/advs.78113 (PMC13634574; doi:10.1002/advs.78113)
Supplement: Supplementary file 1 [file ADVS-9999-e78113-s001.pdf]

Phosphine coordination to Ce, Nd, Np and Pu in ^{Ar}BiAMP^{tBu}-supported complexes and their ^{2}H and ^{13}C isotopologues

Novan A. G. Gray [a†], Yury Minko [a†], Brenna K. Cashman [a†], R. Joseph Lastowski [a], Adam R. Altenhof [b], Harris E. Mason [a], Daniel J. Lussier [a], Frank MacGregor [a], Andrew J. Gaunt [a], and Aaron M. Tondreau [a*]

[a] Chemistry Division, Inorganic, Isotope, and Actinide Chemistry
Los Alamos National Laboratory
Los Alamos, New Mexico 87545, United States
E-mail: tondreau_a@lanl.gov

[b] Geothermal Energy & Geofluids Group
ETH Zurich
Zurich 8092, Switzerland

† These authors contributed equally to this work

Table of Contents

Glossary of Acronyms **S3**

1. Experimental Details

1.1 General Considerations **S4**

1.2 Synthetic Details **S5-S9**

2. Supplementary Data and Spectra

2.1 NMR Spectra **S10-S36**

2.2 UV-vis-NIR data **S37-S43**

2.3 Supplemental Correlation Graphs **S44-S46**

2.4 Additional P-M Bonding Discussion **S48**

3. Single Crystal X-ray Diffraction Data **S49**

3.1 CCDC deposition numbers **S49**

3.2 3,7-di^{tert}butyl-1,5-bis(3,5-dimethylphenyl)-1,5,3,7-diazadiphosphocane (**P_2N_2**) **S50**

3.3 CeI(^{Ar}BiAMP^{tBu})(thf)$_3$ (**1**) **S52**

3.4 NdI(^{Ar}BiAMP^{tBu})(thf)$_3$ (**2**) **S54**

3.5 NpI(^{Ar}BiAMP^{tBu})(thf)$_3$ (**3**) **S56**

3.6 PuI(^{Ar}BiAMP^{tBu})(thf)$_3$ (**4**) **S58**

3.7 PrI(^{Ar}BiAMP^{tBu})(thf)$_3$ **S60**

3.8 TmI(^{Ar}BiAMP^{tBu})(thf)$_3$ **S62**

4. References **S64**

Glossary of Ligand Acronyms

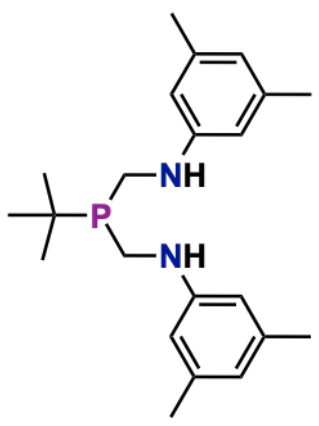

^{Ar}BiAMP^{tBu}

bis((3,5-dimethylphenyl)-aminomethyl)^{tert}butylphosphine

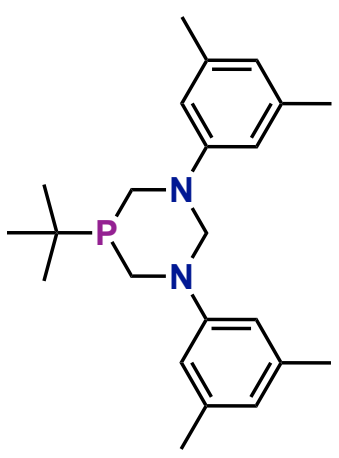

1,3-bis(3,5-dimethylphenyl)-5-tert-butyl-1,3-diaza-5-phosphacyclohexane ((^{Ar}BiAMP^{tBu})CH_2)

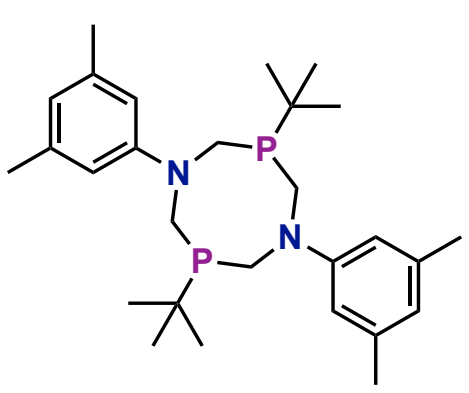

3,7-di-*tert*-butyl-1,5-bis(3,5-dimethylphenyl)-1,5-diaza-3,7-diphosphacyclooctane ($^{tBu}P_2N_2^{Ar}$)

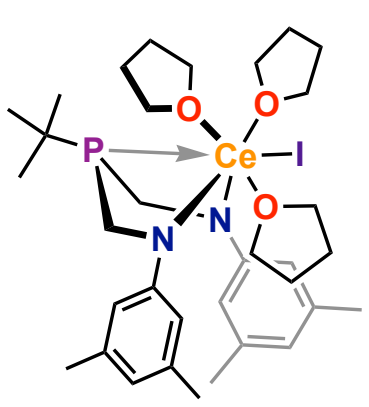

CeI(^{Ar}BiAMP^{tBu})(thf)$_3$ (1)

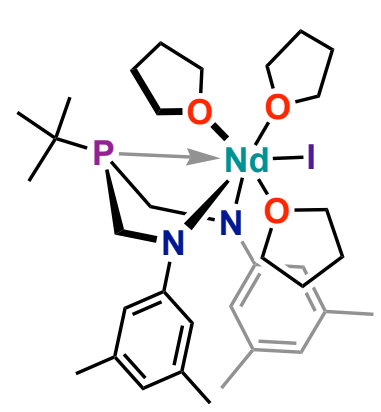

NdI(^{Ar}BiAMP^{tBu})(thf)$_3$ (2)

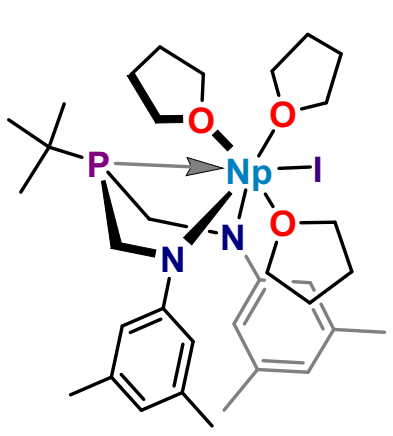

NpI(^{Ar}BiAMP^{tBu})(thf)$_3$ (3)

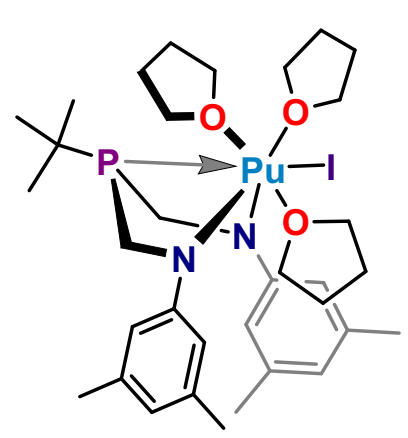

PuI(^{Ar}BiAMP^{tBu})(thf)$_3$ (4)

1. Experimental Details

1.1 General Considerations

Caution! *The ^{237}Np ($t_{½}$ = 2.144×10^{6} years) (Q_{α} = 4.958 MeV) and ^{239}Pu ($t_{½}$ = 24,110 years) (Q_{α} = 5.244 MeV) isotopes – and their daughters – present serious health threats due to their* α-, *β-,* and γ-*emissions. All manipulations of these isotopes were conducted in a radiation laboratory equipped with high efficiency particulate air (HEPA) filtered hoods and in negative pressure gloveboxes. Additional safeguards included continuous air monitoring and use of hand-held radiation monitoring equipment. Entrance to the laboratory space was controlled with a hand and foot radiation monitoring instrumentation and a full body personal contamination monitoring station. The handling of free-flowing solids was restricted to negative pressure gloveboxes equipped with HEPA filters. In addition to standard laboratory PPE, aqueous solutions were handled using multiple layers of latex gloves combined with DuPont™ Tyvek® 400 sleeves to provide overlapping coverage of the arms.*

Caution! *Na/K is a low-melting reactive alloy of sodium and potassium and should be handled only by appropriately trained individuals. Here we use a 1:1 (mol) ratio to generate the liquid alloy to dry the solvents.*

Syntheses and manipulations of transuranium-containing materials were conducted under UHP helium (AirGas) with rigorous exclusion of oxygen and water employing a negative-pressure Vacuum Atmospheres NexGen™ glovebox. Air- and moisture-sensitive manipulations for lanthanide-based complexes were carried out in an MBraun dry box containing a purified argon atmosphere. The solvents THF, toluene, and pentane (Sigma) were dried on Na/K, filtered through dry, neutral alumina, stored on 4 Å molecular sieves, and tested with Na-benzophenone ketyl radical before use. The compounds ^{tert}butylphosphine (95 % Strem), 3,5-dimethylaniline (99 %, Sigma), paraformaldehyde (95 %, Sigma), $^{2}H_2$-paraformaldehyde (99 atom %, Sigma), ^{13}C-paraformaldehyde (99 atom %, Cambridge Isotope Laboratories) were degassed and stored in an inert atmosphere drybox before use. Anhydrous NdI_3 (99.9 %, VWR) and CeI_3 (99.9 %, Sigma) were used as received and kept in an inert atmosphere drybox before use. THF-d_8 was dried using Na/K alloy, filtered through dry, neutral alumina, and stored under argon on 4 Å molecular sieves. $NpI_3(thf)_4$ and $PuI_3(thf)_4$,[64] (trimethylsilyl)methyl potassium,[73] and **^{Ar}BiAMP^{tBu}**,[49] were prepared according to published procedures.

All NMR measurements for the lanthanide metal complexes were conducted in J. Young valve NMR tubes sealed under an inert atmosphere of argon. For transuranium samples, a solution of analyte was loaded into a 4 mm FEP NMR tube liner that was protected from surface contamination with Parafilm while inside a transuranium glovebox. The liner was sealed with two PTFE plugs, brought out of the glovebox, and verified to be free of surface contamination after the parafilm was removed (α-particle detection instruments and a Ludlum 3030E smear count to detect both α- and β-particles); the liner was then loaded into a J-Young tap appended 5 mm NMR tube or a regular NMR tube. In the case of the J-Young loading, the headspace was then evacuated (in the glovebox antechamber) and refilled with He (using the glovebox atmosphere) to provide an inert atmosphere headspace above the sample. ^{1}H, ^{13}C{^{1}H} and ^{31}P{^{1}H} NMR data collection was performed on a 400 MHz Bruker Avance II spectrometer at room temperature or variable temperatures where indicated. ^{1}H, ^{2}H, ^{13}C{^{1}H}, and ^{31}P{^{1}H} NMR spectra for non-transuranic samples were recorded on a Bruker Avance 400 MHz spectrometer operating at 400.132 MHz, 61.42 MHz, 100.693 MHz, and 161.978 MHz, respectively. The ^{1}H and ^{13}C chemical shifts of all spectra are reported relative to $SiMe_4$ or *via* secondary standards based on residual solvent peaks relative to $SiMe_4$; the ^{31}P chemical shifts are reported relative to the referenced ^{1}H NMR spectra. CHN analyses were performed at both Midwest Microlabs and the University of Rochester CENTC Elemental Analysis Facility, B14 Hutchison Hall, 120 Trustee Road, Rochester, NY 14627. *Note on Elemental Analysis: The metal complexes routinely failed elemental analysis, with analytical results consistent with the loss of THF, an observation consistent with related complexes previously reported.*

For transuranium samples, solution phase electronic absorption spectra were collected at ambient temperature using a Varian Cary 6000i UV-vis-NIR spectrometer. The solution was contained in a low volume (1 mL) screw-capped quartz cuvette (1 cm path length) that was loaded in a transuranium glovebox using Parafilm to protect the exterior surface of the cuvette and cap from radioactive contamination (parafilm was removed in fume hood and exterior surfaces of the cuvettes were monitored with α-particle detection instruments prior to data acquisition). Data was collected from 275 to 1,600 nm. For non-transuranium samples, electronic absorption spectra were collected at room temperature on a Cary 5000 UV-vis-NIR spectrophotometer from Agilent Technologies with different concentrations, which are noted. Solid-state UV-vis-NIR measurements were made on single crystals which were placed on a quartz

slide under NVH oil and spectra were obtained using a CRAIC Technologies 2030PV microspectrophotometer; for absorbance measurements, data was collected from 300 to 1800 nm.

Transuranium crystals for single-crystal X-ray diffraction were mounted in NVH oil inside 0.5 mm quartz capillaries (Hampton Research). The quartz capillaries were inserted through silicone stoppers and placed inside test tubes to allow handling inside the transuranium glovebox while mounting crystals without contaminating the exterior surface of the capillary. The capillaries were then cut with nail clippers to appropriate size for mounting on a goniometer. The ends of the cut capillaries were sealed with hot capillary wax before being removed from the glovebox for coating in clear nail varnish (Hard as Nails™) to provide shatter-resilience. During the clipping and wax sealing steps, care must be taken to avoid the capillary touching any contaminated surfaces (this is achieved by the introduction of fresh petri dishes, forceps, clippers, and wax, as needed in conjunction with careful handling techniques to avoid contamination transfer). Following removal from the glovebox, the exterior surfaces of the capillaries were monitored with α-particle detection instruments prior to transport to the X-ray diffraction laboratory. For non-transuranium crystals, single crystals of complexes suitable for X-ray diffraction were coated with OVH oil or Krytox™ in a dry box, placed on a nylon loop and then transferred to a goniometer head of a Bruker D8 Quest equipped with a graphite-monochromatized molybdenum Kα X-ray tube (λ= 0.71073 Å) and a CMOS detector, or the goniometer head of a Bruker D8 Quest using a Mo Kα (λ = 0.71073 Å) IμS 3.0 Microfocus source X-ray generator.[74] A hemisphere routine was used for data collection and determination of lattice constants. The space group was identified, and the data were processed using the Bruker SAINT+ program and corrected for absorption using SADABS.[75,76] The structures were solved using direct methods (SHELXTL) completed by subsequent Fourier synthesis and refined by full-matrix least-squares procedures.[77,78] Olex2 software was used as the graphical interface.[79,80] Crystallographic data for all structures are available from the Cambridge Structural Database.

Graphical Key for the NMR Assignments: Correlating Shifts to Structure. The resonances for the BiAMP fragment are related to their assignment in the NMR spectra through the shorthand notation given.

^{1}H NMR:
1: tBuP
2: Me_{Ar}
3: N-H
4: 5: CH_2
6: *o*-CH
7: *p*-CH

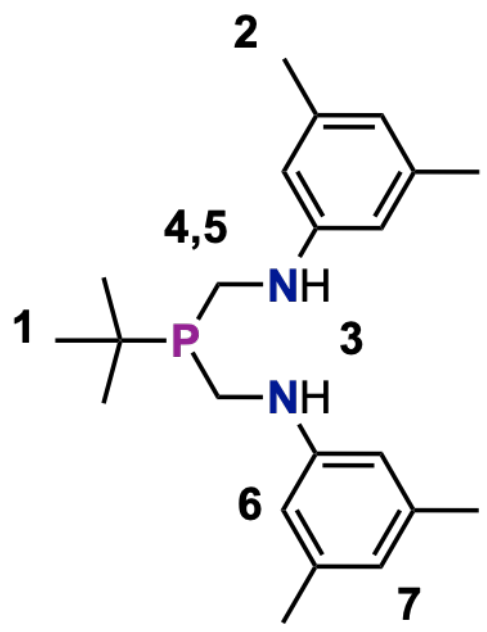

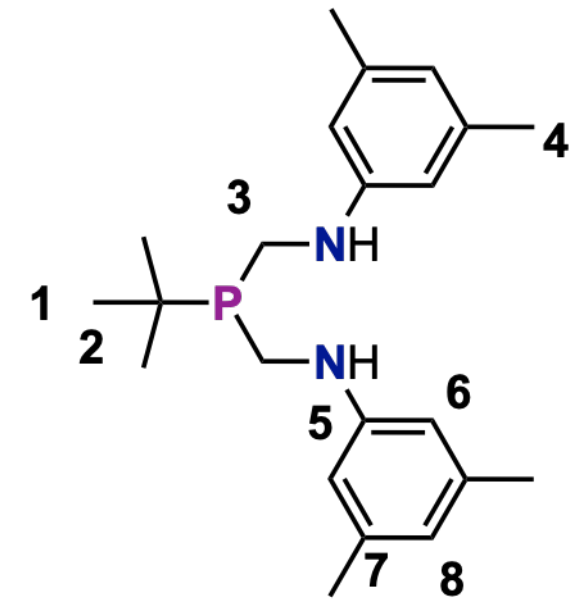

^{13}C NMR:
1: tBu-Me
2: tBu-C
3: CH_2
4: Me_{Ar}
5: N-Ar_{ipso}
6: *o*-CH
7: *m*-CMe
8: *p*-CH

1.2 Synthetic Details

Synthesis of bis((3,5-dimethylphenyl)-aminomethyl)^{tert}butylphosphine (^{Ar}BiAMP^{tBu}) and stable isotope-labelled (d_4-^{Ar}BiAMP^{tBu}), ($^{13}C_2$-^{Ar}BiAMP^{tBu}).

The compound **^{Ar}BiAMP^{tBu}** was made according to previously published procedures.[49] Additional characterization using NMR spectroscopy was performed in THF-d_8 to maintain consistency between ligand and metal complexes. The stable isotope labelled ligands were compared similarly to the natural abundance versions with the use of ^{13}C-CH_2O and ^{2}H-CH_2O to give the products **d_4-^{Ar}BiAMP^{tBu}, ^{13}C-^{Ar}BiAMP^{tBu}**, respectively, which were isolated in 67 % and 71 % respective crystalline yields.

^{Ar}BiAMP^{tBu} - ^{1}H NMR (400.13 MHz, THF-d_8) δ ppm 6.27 (s, 4H, *o*-CH), 6.24 (s, 2H, *p*-CH), 4.57 (brt, 2H, N-H), 3.41 (dt, J_1 = 12.8 Hz, J_2 = 5.2 Hz; 2H, CH_2), 3.29 (dd, J_1 = 12.4 Hz, J_2 = 5.2 Hz; 2H, CH_2), 2.16 (s, 12H, Me_{Ar}), 1.16 (d, J = 11.1 Hz; 9H, tBuP). ^{13}C{^{1}H} NMR (100.62 MHz, THF-d_8) δ ppm 150.4 (d, J = 7.3 Hz, N-Ar_{ipso}), 138.8 (*m*-CMe), 119.6 (*p*-CH), 111.8 (*o*-CH), 40.8 (d, J = 13.5 Hz, CH_2), 28.8 (d, J = 13.3 Hz, tBu-C), 28.2 (d, J = 12.7 Hz, tBu-Me), 21.9 (Me_{Ar}). ^{31}P{^{1}H} NMR (161.98 MHz, THF-d_8) δ ppm -8.38 (s, tBuP). ***Note:*** *We rationalize the symmetric ^{1}H NMR spectrum due to degenerate hydrogen bond interconversion between the N-H donor-acceptor pairs.*

d_4-^{Ar}BiAMP^{tBu} - ^{1}H NMR (400.13 MHz, THF-d_8) δ ppm 6.26 (s, 2H, *p*-CH), 6.23 (s, 4H, *o*-CH), 4.55 (brs, 2H, N-H), 2.15 (s, 12H, Me_{Ar}), 1.15 (d, J = 11.1 Hz; 9H, tBuP). ^{2}H NMR (61.42 MHz, THF-d_8) δ 3.31, 3.19. ^{13}C{^{1}H} NMR (100.62 MHz, THF-d_8) δ ppm 150.3 (d, J = 7.3 Hz, N-Ar_{ipso}), 138.7 (*m*-CMe), 119.4 (*p*-CH), 111.7 (*o*-CH), 40.1 (m, J = 13.1 Hz, CH_2), 28.6 (d, J = 12.6 Hz, tBu-C), 28.1 (d, J = 13.2 Hz, tBu-Me), 21.8 (Me_{Ar}). ^{31}P{^{1}H} NMR (161.98 MHz, THF-d_8) δ ppm -9.83 (s, tBuP).

$^{13}C_2$-^{Ar}BiAMP^{tBu} - ^{1}H NMR (400.13 MHz, THF-d_8) δ ppm 6.44 (s, 2H, *p*-CH), 6.26 (s, 4H, *o*-CH), 3.64 (brs, 2H, N-H), 3.25 (dt, J_1 = 12.8 Hz, J_2 = 5.2 Hz; 2H, CH_2), 3.12 (dd, J_1 = 12.4 Hz, J_2 = 5.2 Hz; 2H, CH_2), 2.21 (s, 12H, Me_{Ar}), 0.95 (d, J = 11.6 Hz; 9H, tBuP). ^{13}C{^{1}H} NMR (100.62 MHz, THF-d_8) δ ppm 149.2 (d, J = 8.0 Hz, N-Ar_{ipso}), 138.7 (*m*-CMe), 120.3 (*p*-CH), 111.8 (*o*-CH), 40.7 (d, J = 13.1 Hz, CH_2), 28.0 (d, J = 12.1 Hz, tBu-C), 27.7 (d, J = 12.1 Hz, tBu-Me), 21.8 (Me_{Ar}). ^{31}P{^{1}H} NMR (161.98 MHz, THF-d_8) δ ppm -8.6 (t, J = 12.1 Hz, tBuP).

Synthesis of 5-(*tert*-butyl)-1,3-bis(3,5-dimethylphenyl)-1,3,5-diazaphosphinane (^{Ar}BiAMP^{tBu}-CH_2) and NMR characterization of stable isotope-labelled (d_4-^{Ar}BiAMP^{tBu}-CH_2), ($^{13}C_2$-^{Ar}BiAMP^{tBu}-CH_2).

The compound **^{Ar}BiAMP^{tBu}-CH_2** was previously published as a minor component of the synthesis of **^{Ar}BiAMP^{tBu}**.[49] Synthesis of the NA **^{Ar}BiAMP^{tBu}-CH_2** was performed purposefully here, while the NMR characterization of the ^{2}H and ^{13}C isotopologues was performed using isolation techniques described previously[49] from the synthesis of **d_4-^{Ar}BiAMP^{tBu}** and **$^{13}C_2$-^{Ar}BiAMP^{tBu}**.

Synthesis of 1,3-bis(3,5-dimethylphenyl)-5-tert-butyl-1,3-diaza-5-phosphacyclohexane ((^{Ar}BiAMP^{tBu})CH_2)
Route A: The phosphacyclohexane derivative **(^{Ar}BiAMP^{tBu})CH_2** is a byproduct in the synthesis of **^{Ar}BiAMP^{tBu}** and can be isolated as previously described.[49]

Route B: A suspension of paraformaldehyde (79.5 mg; 2.649 mmol) and *tert*-butylphosphine (124.9 mg; 1.318 mmol) was heated to 90 °C until the paraformaldehyde completely dissolved (c.a. 1 hr) after which 3,5-dimethylaniline (321.0 mg; 2.649 mmol) and additional paraformaldehyde (39.8 mg; 1.324 mmol) were added to the colorless solution. The resulting pale orange suspension was stirred at 70 °C for 24 hrs before solvent was removed under vacuum leaving a waxy off-white residue. The residue was repeatedly suspended in minimal amounts of pentane and re-dried leaving **(^{Ar}BiAMP^{tBu})CH$_2$** as a slightly tacky white solid. Dissolution of the crude solid in pentane followed by cooling to -35 °C resulted in the formation of fine colorless needles (192.7 mg; 0.535 mmol) in 40.4% crystalline yield. The NMR spectra were indistinguishable from those reported previously.

Synthesis of 3,7-di-*tert*-butyl-1,5-bis(3,5-dimethylphenyl)-1,5-diaza-3,7-diphosphacyclooctane (^{tBu}P$_2$N$_2$^{Ar})

Route A: **^{tBu}P$_2$N$_2$^{Ar}** is a byproduct in the previously reported synthesis of **^{Ar}BiAMP^{tBu}**.[3] During the course of the reaction, **^{tBu}P$_2$N$_2$^{Ar}** precipitated as a fine white powder which was collected by filtration and washed with pentane to provide **^{tBu}P$_2$N$_2$^{Ar}** in 7.0% yield. The isotopologues ***d*$_8$- ^{tBu}P$_2$N$_2$^{Ar}** and **^{13}C- ^{tBu}P$_2$N$_2$^{Ar}** were isolated from the synthesis of the corresponding labelled **^{Ar}BiAMP^{tBu}**.

Route B: Paraformaldehyde (57.5 mg; 1.916 mmol) and *tert*-butylphosphine (100.0 mg; 1.054 mmol) were heated to 90 °C until all paraformaldehyde dissolved (c.a. 1 hr) yielding a clear, colorless oil to which a dilute toluene solution of 3,5-dimethylaniline (116.1 mg; 0.958 mmol) was slowly added dropwise. During the addition of aniline, a fine white precipitate began to form, and the resulting suspension was allowed to stir at 70 °C for 48 hr. After cooling to room temperature, the suspension was filtered and the collected precipitate washed thoroughly with pentane to yield **^{tBu}P$_2$N$_2$^{Ar}** as a white powder (76.0 mg; 0.162 mmol) in 33.8% yield.

^{tBu}P$_2$N$_2$^{Ar} :^{1}H NMR (400.13 MHz, C*D*Cl$_3$) δ 6.31 (s, *o*-CH, 4H), 6.30 (s, *p*-CH, 2H), 4.39 (dd, CH$_2$, J_1 = 14.8, J_2 = 11.5 Hz, 4H), 3.47 (dd, CH$_2$ J_1 = 14.8, J_2 = 4.9 Hz, 4H), 2.21(s, Me$_{Ar}$, 12H), 1.28 (d, ^{t}BuP, 18H, J$_{PH}$ = 11 HZ) .^{13}C{^{1}H} NMR (100.62 MHz, C*D*Cl$_3$) δ 146.07, 138.36, 118.30, 110.71 (t, *J* = 2.6 Hz), 55.07 (d, *J* = 24.4 Hz), 29.70 (d, *J* = 16.0 Hz), 28.40 – 26.55 (m), 22.05. ^{31}P{^{1}H} NMR (161.98 MHz, C*D*Cl$_3$) δ -26.99.

***d*$_8$-^{tBu}P$_2$N$_2$^{Ar}**: ^{1}H NMR (400.13 MHz, C*D*Cl$_3$) δ 6.09-5.98 (m, *o*-CH, *p*-CH 6H), 2.21(s, Me$_{Ar}$, 12H), 1.28 (d, ^{t}BuP, 18H, J$_{PH}$ = 10 Hz). ^{2}H NMR (61.42 MHz, C*H*Cl$_3$) δ 4.37 (br. s), 3.45 (br. s). ^{13}C{^{1}H} NMR (100.62 MHz, C*D*Cl$_3$) δ 146.26, 138.55, 118.49, 110.91, 54.4 (br), 29.88 (d, *J* = 15.4 Hz), 27.81 (m), 22.27. ^{31}P{^{1}H} NMR (161.98 MHz, C*D*Cl$_3$) δ -28.4.

^{13}C- ^{tBu}P$_2$N$_2$^{Ar}: ^{1}H NMR (400.13 MHz, C*D*Cl$_3$) δ 6.32-6.30 (m, *o*-CH, *p*-CH 6H), 4.40 (dt, CH$_2$, J_1 = 135.2, J_2 = 13.3 Hz, 4H), 3.48 (ddq, CH$_2$, J_1 = 135.9, J_2 = 14.8, J_3 = 5 Hz, 4H) 2.21(s, Me$_{Ar}$, 12H), 1.28 (d, ^{t}BuP, 18H, J$_{PH}$ = 10 Hz). ^{13}C{^{1}H} NMR (100.62 MHz, C*D*Cl$_3$) δ 146.1, 138.36, 118.32, 110.73, 55.07 (d, *J* = 24.0 Hz), 29.68 (d, 15.5 Hz), 27.58 (d, 11.6 Hz), 22.04. ^{31}P{^{1}H} NMR (161.98 MHz, C*D*Cl$_3$) δ -27.0 (t, J$_{CH}$ = 23.1 Hz).

General Procedure I: Synthesis of MI(^{Ar}BiAMP^{tBu})(thf)$_3$ (M = Ce, Nd)

In a glove box under inert Ar atmosphere, MI$_3$ (1.0 equiv.) was placed in a 20 mL scintillation vial, suspended in THF and stirred at ambient temperature for 1 h until a white slurry of MI$_3$(thf)$_4$ formed. The resulting mixture was cooled to -35 °C. In a separate reaction vial, **^{Ar}BiAMP^{tBu}** ligand (1.01 equiv.) was dissolved in THF and cooled to -35 °C. A freshly prepared solution of (trimethylsilyl)methyl potassium (2.02 equiv.) in cold THF was then added dropwise to the pre-cooled solution of **^{Ar}BiAMP^{tBu}**. This reaction mixture was then cooled at -35 °C for 20 min, and the resulting clear light-green homogeneous solution of the **^{Ar}BiAMP^{tBu}** bis(potassium) salt was added dropwise to the cold stirring slurry of the MI$_3$-thf adduct. The reaction mixture was then allowed to warm to room temperature and was stirred for 3 h. White precipitate was separated from the reaction mixture using centrifugation (10 min @5,000 rpm) and filtration through a glass fiber filter. The obtained homogeneous filtrate was concentrated under reduced pressure to *ca.* 2-3 mL, and the product was crystallized using either a vapor diffusion of pentane into the THF solution, or by layering *n*-pentane at -35 °C.

***tert*-Butyl(bis(methyleneamido3,5-dimethylphenyl))phosphine-cerium(III)iodide tris(tetrahydrofuran) complex CeI(^{Ar}BiAMP^{tBu})(thf)$_3$ (1)**

The title compound was prepared according to General Procedure I using CeI$_3$ (175 mg; 0.34 mmol) suspended in approximately 3 ml of THF. Bis(potassium) salt of **^{Ar}BiAMP^{tBu}** ligand was prepared in 2 ml of THF using **^{Ar}BiAMP^{tBu}** (122 mg; 0.343 mmol) and (trimethylsilyl)methylpotassium (87 mg; 0.686 mmol). The title compound was isolated as crystalline off-white solid (173 mg; 0.28 mmol) in 82% crystalline yield. Single crystals suitable for SC-XRD analysis were obtained by slow vapor diffusion at -35 °C (48-72 h) using pentane and concentrated THF solution of the title compound.

^{1}H NMR (400.13 MHz, THF-*d*$_8$) δ 27.7 (s, fwhm = 80 Hz 2H, CH$_2$), 4.10 (s, 2H, *p*-CH), 0.07 (s, fwhm = 30 Hz, 2H, CH$_2$), -0.82 (s, fwhm = 360 Hz, 12H, Me$_{Ar}$), -3.52 (s, 9H, tBu-C). ^{31}P{^{1}H} NMR (161.98 MHz, THF-*d*$_8$) δ –0.93 (s,

fwhm = 90 Hz). Elemental Analysis (*see note above*) for: $C_{34}H_{55}IN_2O_3PCe$ calcd. %C, 48.74, %H, 6.62, %N 3.34; Anal. %C, 45.75, %H, 6.23, %N, 3.86.

***tert*-Butyl(bis(methylene-*d*$_2$-amido-3,5-dimethylphenyl))phosphine-cerium(III)iodide tris(tetrahydrofuran) complex CeI(*d*$_4$-^{Ar}BiAMP^{tBu})(thf)$_3$ (^{2}H-1).** The title compound was prepared according to General Procedure I using CeI_3 (245.5 mg; 0.47 mmol) suspended in 4 ml of THF. Bis(potassium) salt of ***d*$_4$-^{Ar}BiAMP^{tBu}** ligand was prepared in 3 ml of THF using ***d*$_4$-^{Ar}BiAMP^{tBu}** (171.2 mg; 0.475 mmol) and (trimethylsilyl)methylpotassium (120 mg; 0.95 mmol). The title compound was isolated as crystalline off-white solid (308.8 mg; 0.32 mmol) in 78% crystalline yield.

^{1}H NMR (400.13 MHz, THF-*d*$_8$) δ 4.08 (s, 2H, *p*-CH), -0.83 (s, fwhm = 370 Hz, 12H, Me$_{Ar}$), -3.44 (s, 9H, tBu-C). ^{2}H NMR (61.42 MHz, THF-*d*$_8$) δ 27.3 (d, $^2J_{PD}$ = 11.6 Hz, 2D, CD$_2$), 0.24 (s, fwhm = 17 Hz, 2D, CD$_2$). ^{31}P{^{1}H} NMR (161.98 MHz, THF-*d*$_8$) δ -3.3 (s, fwhm = 90 Hz).

***tert*-Butyl(bis(methylene-^{13}C-amido-3,5-dimethylphenyl))phosphine-cerium(III)iodide tris(tetrahydrofuran) complex CeI(^{13}C$_2$-^{Ar}BiAMP^{tBu})(thf)$_3$ (^{13}C-1).** The title compound was prepared according to General Procedure I using CeI_3 (250 mg; 0.48 mmol) suspended in 4 ml of THF. Bis(potassium) salt of **^{13}C$_2$-^{Ar}BiAMP^{tBu}** ligand was prepared in 3 ml of THF using **^{13}C$_2$-^{Ar}BiAMP^{tBu}** (173.9 mg; 0.485 mmol) and (trimethylsilyl)methylpotassium (123 mg; 0.98 mmol). The title compound was isolated as crystalline off-white solid (330 mg; 0.35 mmol) in 82% crystalline yield.

^{1}H NMR (400.13 MHz, THF-*d*$_8$) δ 26.7 (d, $^1J_{CH}$ = 127.6 Hz, 2H, CH$_2$), 4.10 (s, 2H, *p*-CH), 0.09 (d, $^1J_{CH}$ = 130.8 Hz, 2H, CH$_2$), -0.82 (s, fwhm = 330 Hz, 12H, Me$_{Ar}$), -3.50 (s, 9H, tBu-C). ^{13}C{^{1}H} NMR (100.60 MHz, THF-*d*$_8$) δ ppm 30.9–29.6 (br, CH$_2$). ^{31}P{^{1}H} NMR (161.98 MHz, THF-*d*$_8$) δ –0.96 (s, fwhm = 60 Hz).

***tert*-Butyl(bis(methyleneamido-3,5-dimethylphenyl))phosphine-neodymium(III)iodide tris(tetrahydrofuran) complex NdI(^{Ar}BiAMP^{tBu})(thf)$_3$ (2).**

The title compound was prepared according to General Procedure I using NdI_3 (525 mg; 1.0 mmol) suspended in 5 ml of THF. Bis(potassium) salt of **^{Ar}BiAMP^{tBu}** ligand was prepared in 4 ml of THF using **^{Ar}BiAMP^{tBu}** (360.1 mg; 1.01 mmol) and (trimethylsilyl)methylpotassium (255.2 mg; 2.02 mmol). The title compound was isolated as crystalline blue solid (710 mg; 0.845 mmol) in 84.5 % crystalline yield. Single crystals suitable for SC-XRD analysis were obtained by slow vapor diffusion at -35 °C (48-72 h) using pentane and concentrated THF solution of the title compound.

^{1}H NMR (400.13 MHz, THF-*d*$_8$) δ. 41.1 (s, fwhm = 30 Hz, 2H, CH$_2$), 17.4 (s, fwhm = 17 Hz, 2H, CH$_2$), 8.49 (s, fwhm = 1750 Hz, 4H, *o*-CH), 5.60 (s, 2H, *p*-CH), 1.30 (s, fwhm = 360 Hz, 12H, Me$_{Ar}$), -3.81 (s, 9H, tBu-C). ^{31}P{^{1}H} NMR (161.98 MHz, THF-*d*$_8$) δ 287.8 (s, fwhm = 110 Hz, tBuP). Elemental Analysis (*see note above*) for: $C_{34}H_{55}INdN_2O_3P$ calcd. %C, 48.81, %H, 6.63, %N, 3.35; Anal. %C, 47.49, %H, 6.50, %N, 3.13.

***tert*-Butyl(bis(methylene-*d*$_2$-amido-3,5-dimethylphenyl))phosphine-neodymium(III)iodide tris(tetrahydrofuran) complex NdI(*d*$_4$-^{Ar}BiAMP^{tBu})(thf)$_3$ (^{2}H-2).**

The title compound was prepared according to General Procedure I using NdI_3 (262.5 mg; 0.5 mmol) suspended in 4 ml of THF. Bis(potassium) salt of ***d*$_4$-^{Ar}BiAMP^{tBu}** ligand was prepared in 3 ml of THF using ***d*$_4$-^{Ar}BiAMP^{tBu}** (182.1 mg; 0.505 mmol) and (trimethylsilyl)methylpotassium (128 mg; 1.01 mmol). The title compound was isolated as crystalline blue solid (335.6 mg; 0.40 mmol) in 80% crystalline yield.

^{1}H NMR (400.13 MHz, THF-*d*$_8$) δ. 8.66 (s, fwhm = 1610 Hz, 4H, *o*-CH), 5.63 (s, 2H, *p*-CH), 1.33 (s, fwhm = 350 Hz, 12H, Me$_{Ar}$), -3.67 (s, 9H, tBu-C). ^{2}H NMR (61.42 MHz, THF-*d*$_8$) δ 41.0 (s, fwhm = 21 Hz, 2D, CD$_2$), 17.5 (s, fwhm = 15 Hz, 2D, CD$_2$). ^{31}P{^{1}H} NMR (161.98 MHz, THF-*d*$_8$) δ 279.8 (s, fwhm = 130 Hz, tBuP).

***tert*-Butyl(bis(methylene-^{13}C-amido-3,5-dimethylphenyl))phosphine-neodymium(III)iodide tris(tetrahydrofuran) complex NdI(^{13}C$_2$-^{Ar}BiAMP^{tBu})(thf)$_3$ (^{13}C-2).**

The title compound was prepared according to General Procedure I using NdI_3 (262.5 mg; 0.50 mmol) suspended in 4 ml of THF. Bis(potassium) salt of **^{13}C$_2$-^{Ar}BiAMP^{tBu}** ligand was prepared in 3 ml of THF using **^{13}C$_2$-^{Ar}BiAMP^{tBu}** (181 mg; 0.505 mmol) and (trimethylsilyl)methylpotassium (127.6 mg; 1.01 mmol). The title compound was isolated as crystalline blue solid (350 mg; 0.415 mmol) in 83 % crystalline yield.

^{1}H NMR (400.13 MHz, THF-*d*$_8$) δ 41.0 (d, $^1J_{CH}$ = 134.0 Hz, 2H, CH$_2$), 17.5 (d, $^1J_{CH}$ = 131.7 Hz, 2H, CH$_2$), 8.69 (s, fwhm = 1690 Hz, 4H, *o*-CH), 5.64 (s, 2H, *p*-CH), 1.33 (s, fwhm = 360 Hz, 12H, Me$_{Ar}$), -3.72 (s, 9H, tBu-C). ^{13}C{^{1}H}

NMR (100.06 MHz, THF-d_8) δ 2.65–1.19 (m, C̲H$_2$). ^{31}P{^{1}H} NMR (161.98 MHz, THF-d_8) δ ppm 286.8 (s, fwhm = 140 Hz, tBuP̲).

Note: *The Tm and Pr congeners complexes were made similarly to all other complexes reported but were prepared on a small scale (~15 mg total MI$_3$) in preparation for work with Pu on small scales. This is not meant to be a full reporting of these molecules as the discussion focuses on the isoradial Ln/An pairs, but rather simply to provide stronger solid-state structural analysis of the series.*

PrI(^{Ar}BiAMP^{tBu})(thf)$_3$; 1H NMR (400.13 MHz, THF-d_8) 50.8 (s, 2H), 10.3 (s, fwhm = 21 Hz, 2H), 5.4 (s, fwhm = 5 Hz, 2H), 0.0 (s, fwhm = 726 Hz, 12H, ArMe), -7.5 (d, $^3J_{PH}$ 6.3 Hz, 9H, ^{t}Bu). ^{31}P{^{1}H} NMR (161.98 MHz, THF-d_8) 165.5 (s, fwhm = 34 Hz).

TmI(^{Ar}BiAMP^{tBu})(thf)$_3$: ^{1}H NMR (400.13 MHz, THF-d_8) 119.5 (s, fwhm = 3740 Hz 2H), 110.0 (s, fwhm = 181 Hz, 9H, ^{t}Bu), ~-1.7 (s, fwhm = 2192 Hz, 12H, ArMe), -25.6 (s, fwhm = 19 Hz, 4H, *o*-Ar), ~-67 (s, fwhm = 12198 Hz, 2H), ~-89 (s, fwhm = 6260 Hz, 2H). ^{31}P{^{1}H} NMR (161.98 MHz, THF-d_8) 801.7 (s, fwhm = 1341 Hz).

tert-Butyl(bis(methylene-^{13}C-amido-3,5-dimethylphenyl))phosphine-neptunium(III)iodide tris(tetrahydrofuran) complex NpI($^{13}C_2$-^{Ar}BiAMP^{tBu})(thf)$_3$ (^{13}C-3).

A 20 mL scintillation vial with a stir bar was charged with NpI$_3$(thf)$_4$ (25.0 mg, 0.0276 mmol) and ~1 mL of THF was added to the solid, forming an orange slurry. The resulting mixture was cooled to –35 °C. In a separate vial, **$^{13}C_2$-^{Ar}BiAMP^{tBu}** ligand (9.9 mg, 1.0 equiv.) was dissolved in THF and cooled to –35 °C. A freshly prepared solution of (trimethylsilyl)methyl potassium (7.0 mg, 2.0 equiv.) in cold THF was then added dropwise to the pre-cooled solution of **$^{13}C_2$-^{Ar}BiAMP^{tBu}**. This reaction mixture was then cooled at –35 °C for 20 min, and the resulting clear light-green homogeneous solution of the "**$K_2$$^{13}C_2$-^{Ar}BiAMP^{tBu}**" salt was added dropwise to the cold stirring slurry of NpI$_3$(thf)$_4$, resulting in the slight darkening of the orange mixture and the formation of white precipitate. The reaction was allowed to stir at room temperature for 2 hours, after which time the slurry was filtered through a glass fiber plug to remove KI, and the mother liquors were collected into a pre-weighed 4 mL scintillation vial. The solution was then reduced *in vacuo* to ~0.1 mL, layered with ~3mL of pentane and left to recrystallize at –35 °C for 4 days. The mother liquors were decanted, and the resultant dark red crystals/waxy solids were dried under reduced pressure for ~5 minutes. **NpI($^{13}C_2$-^{Ar}BiAMP^{tBu})(thf)$_3$ (^{13}C-3)** was obtained in 56 % yield (14.3 mg, 0.0155 mmol) as either a dark red waxy solid or dark red crystals.

^{1}H NMR (400.13 MHz, 50 °C, THF-d_8) δ ppm. 14.6 (m, "*J*" = 137.5 Hz, 2H, CH̲$_2$), 13.8 (m, "*J*" = 131.2 Hz, 2H, CH̲$_2$), 8.08 (s, 9H, tBu̲-C), 7.74 (s, fwhm = 400 Hz, 4H, *o*-CH̲). 4.32 (s, 2H, *p*-CH̲), 1.95 (s, fwhm = 40 Hz, 12H, Me̲$_{Ar}$). ^{13}C{^{1}H} NMR (100.06 MHz, 25 °C, THF-d_8) δ ppm -154.2 (s, C̲H$_2$). ^{31}P{^{1}H} NMR (161.98 MHz, 25 °C, THF-d_8) δ ppm -231.1 (s, fwhm = 45 Hz, tBuP̲).

tert-Butyl(bis(methylene-^{13}C-amido-3,5-dimethylphenyl))phosphine-plutonium(III)iodide tris(tetrahydrofuran) complex PuI($^{13}C_2$-^{Ar}BiAMP^{tBu})(thf)$_3$ (^{13}C-4).

A 20 mL scintillation vial with a stir bar was charged with PuI$_3$(thf)$_4$ (20.0 mg, 0.0220 mmol) and ~1 mL of THF was added to the solid to form a tan-colored slurry. The resulting mixture was cooled to –35 °C. In a separate vial, **$^{13}C_2$-^{Ar}BiAMP^{tBu}** ligand (7.9 mg, 1.0 equiv.) was dissolved in THF and cooled to –35 °C. A freshly prepared solution of (trimethylsilyl)methyl potassium (5.5 mg, 2.0 equiv.) in cold THF was then added dropwise to the pre-cooled solution of **$^{13}C_2$-^{Ar}BiAMP^{tBu}**. This reaction mixture was then cooled at –35 °C for 20 min, and the resulting clear light-green homogeneous solution of the "**$K_2$$^{13}C_2$-^{Ar}BiAMP^{tBu}**" salt was added dropwise to the cold stirring slurry of PuI$_3$(thf)$_4$, resulting in the formation of a light blue-green solution with concomitant precipitation of KI. The reaction was allowed to stir at room temperature for 2 hours, after which time the slurry was filtered through a glass fiber plug to remove KI, and the mother liquors were collected into a pre-weighed 4 mL scintillation vial. The solution was then reduced *in vacuo* to ~0.1 mL, layered with ~3 mL of pentane and left to recrystallize at –35 °C for 2 days. The mother liquors were decanted, and the blue-green crystals were dried under reduced pressure for ~5 minutes. **PuI($^{13}C_2$-^{Ar}BiAMP^{tBu})(thf)$_3$ (^{13}C-4)** was obtained in 76 % yield (15.6 mg, 0.0166 mmol) as blue-green crystals.

^{1}H NMR (400.13 MHz, 35 °C, THF-d_8) δ ppm. 6.62 (s, fwhm = 150 Hz, 4H, *o*-CH̲), 5.89 (s, 2H, *p*-CH̲), 5.57 (d, $^1J_{CH}$ = 125.4 Hz, 2H, CH̲$_2$), 3.26 (d, $^1J_{CH}$ = 120.7 Hz, 2H, CH̲$_2$), 2.06 (s, 12H, Me̲$_{Ar}$), 1.97 (s, 9H, tBu̲-C). ^{13}C{^{1}H} NMR (100.06 MHz, 25 °C, THF-d_8) δ ppm 8.29 (s, fwhm = 90 Hz, C̲H$_2$). ^{31}P{^{1}H} NMR (161.98 MHz, 25 °C, THF-d_8) δ ppm -35.0 (s, fwhm = 75 Hz, tBuP̲).

Note: *Syntheses of the natural abundance Np and Pu complexes **(^{Ar}BiAMP^{tBu})NpI(thf)$_3$ (3)** and **(^{Ar}BiAMP^{tBu})PuI(thf)$_3$ (4)** were conducted under identical conditions and scales, which produced similar yields to those of the respective ^{13}C isotopologues (**^{13}C-3** and **^{13}C-4**).*

2. Supplementary Data and Spectra

2.1 NMR spectra

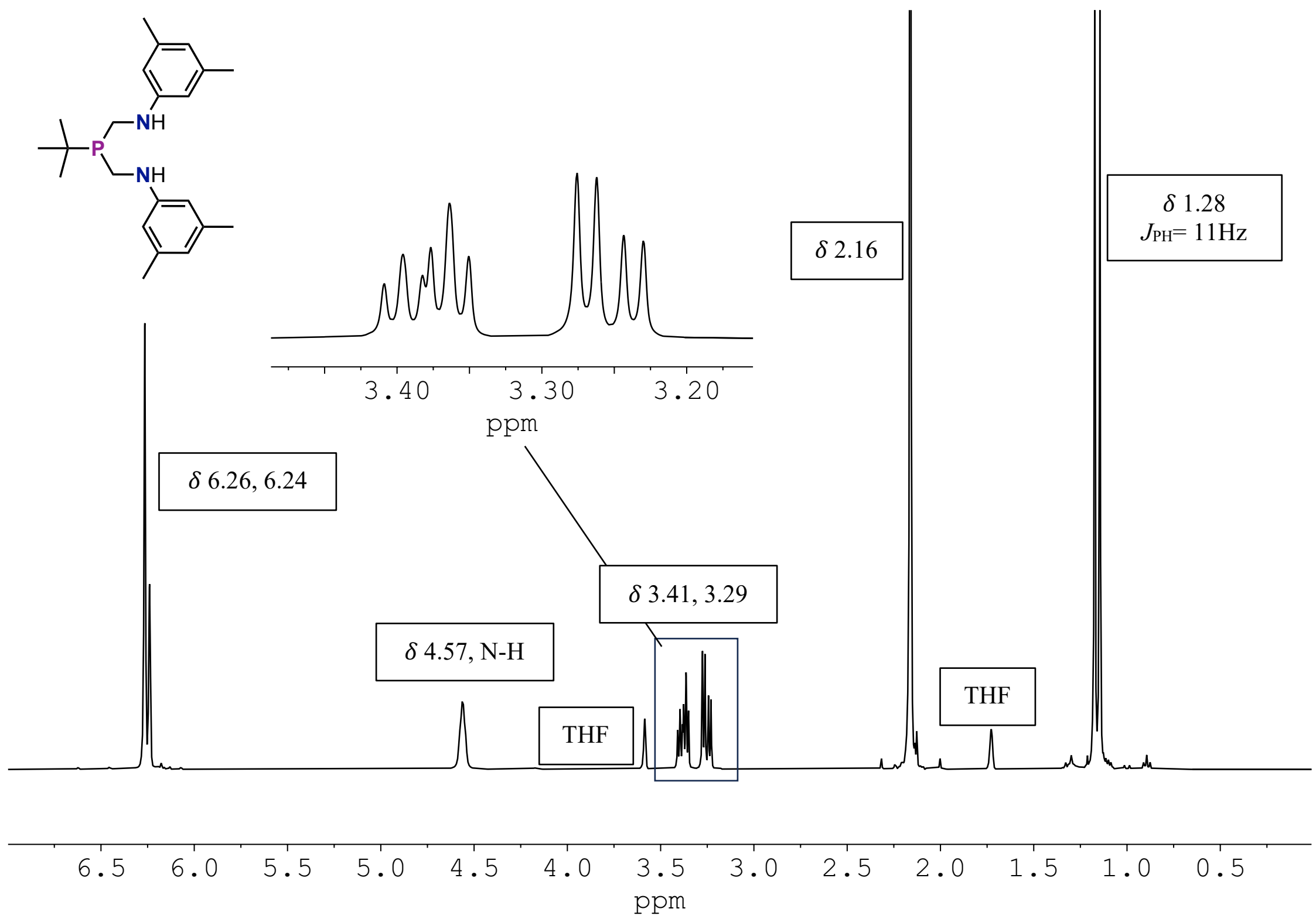

Figure S1. ^{1}H NMR spectrum of ^{Ar}**BiAMP**^{tBu} acquired in THF-d_8 at room temperature.

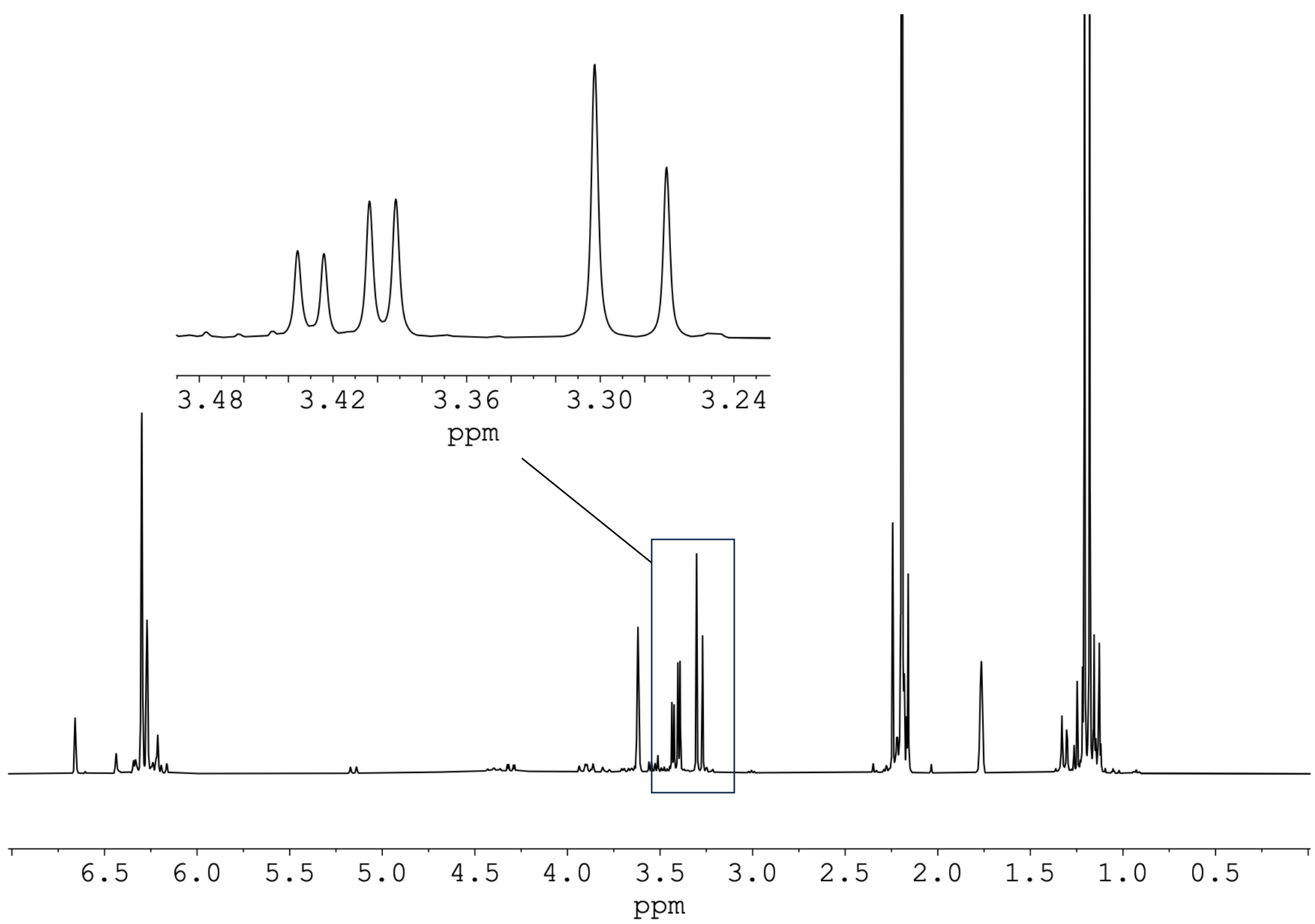

Figure S2. ^{1}H NMR spectra of ^{Ar}**BiAMP**^{tBu} acquired in THF-d_8 after 24 hours in solution.

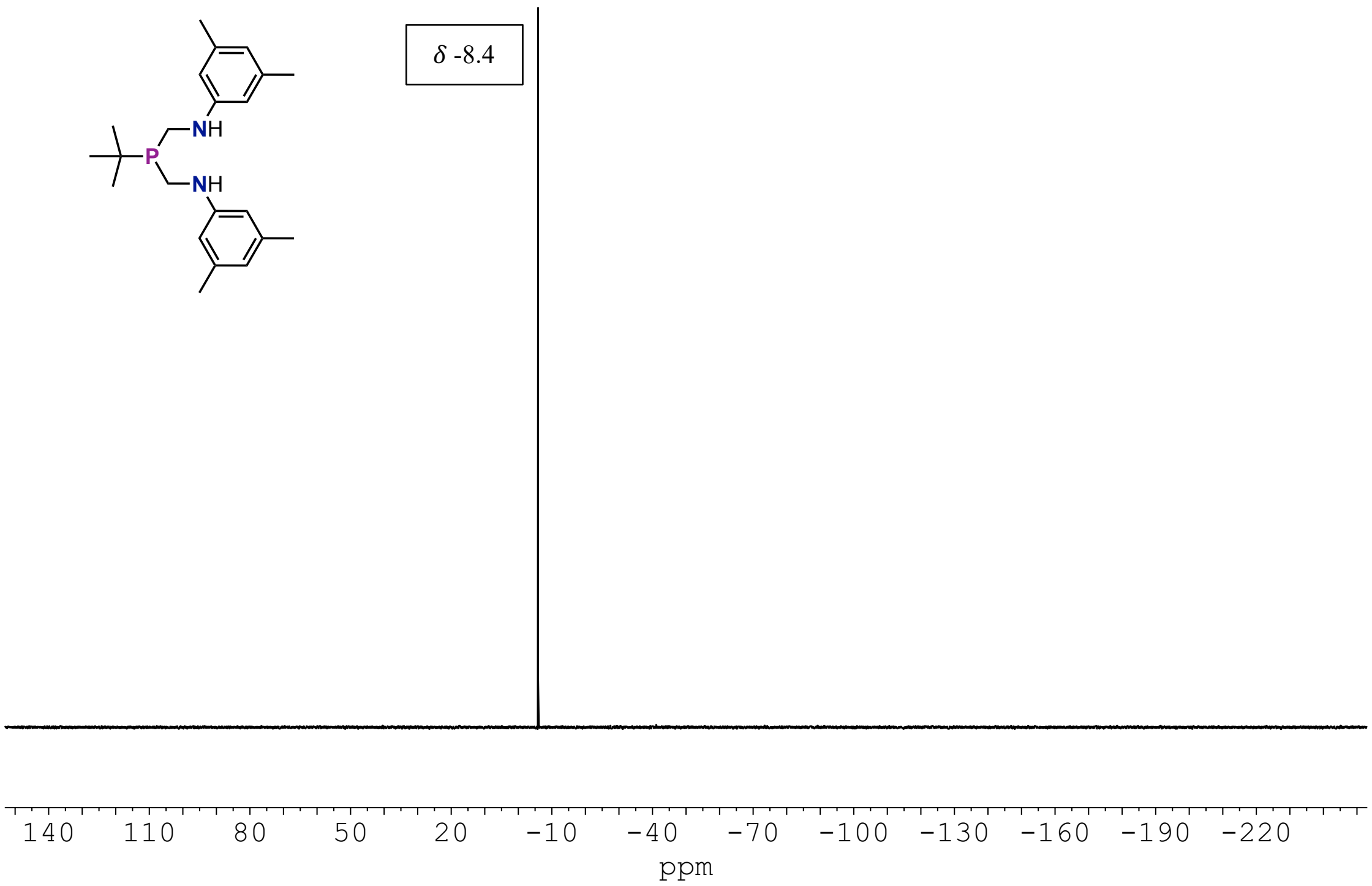

Figure S3. $^{31}P\{^{1}H\}$ NMR spectrum of ^{Ar}**BiAMP**^{tBu} acquired in THF at room temperature.

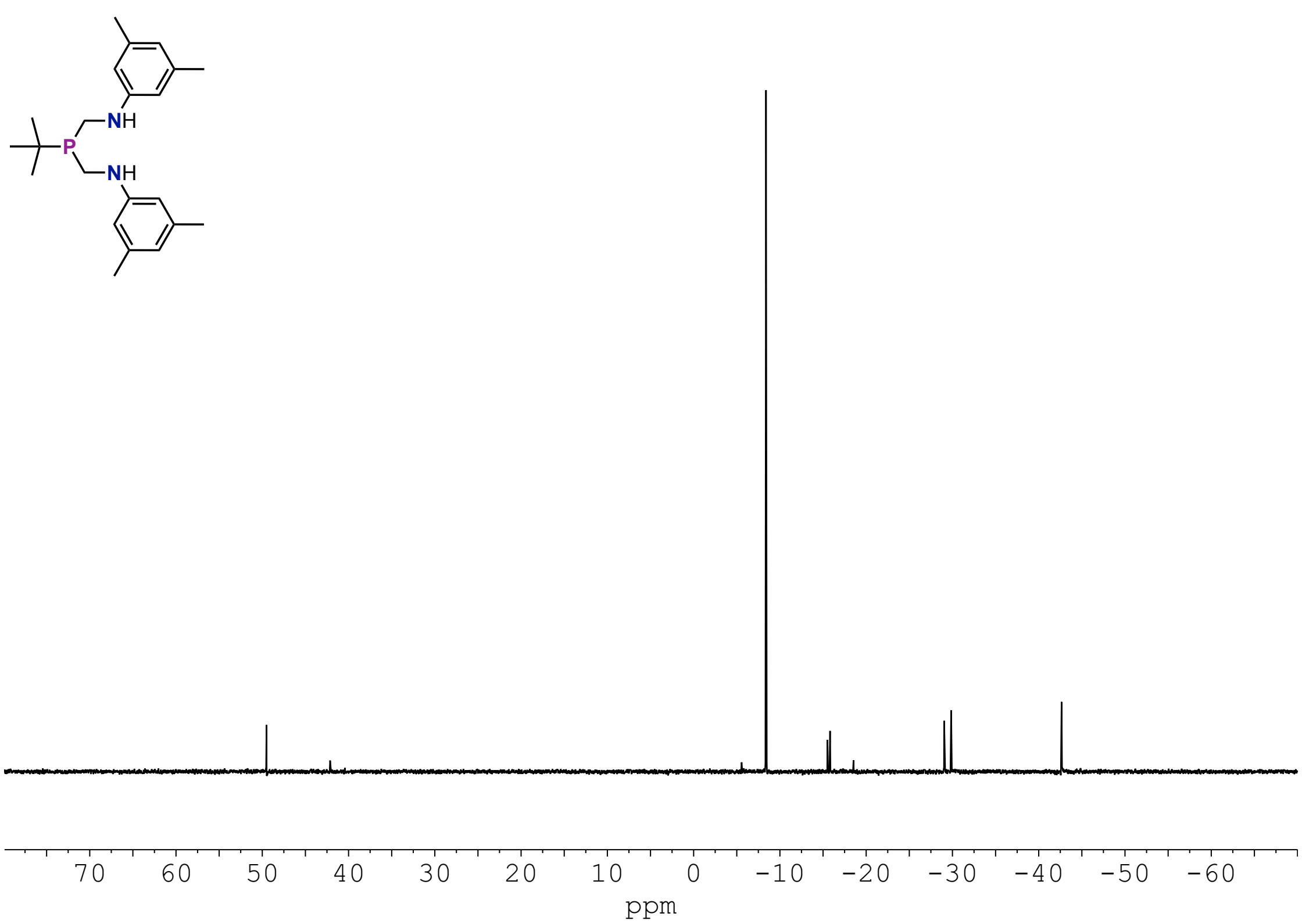

Figure S4. $^{31}P\{^{1}H\}$ NMR spectrum of ^{Ar}**BiAMP**^{tBu} acquired in THF at room temperature after 24 hours.

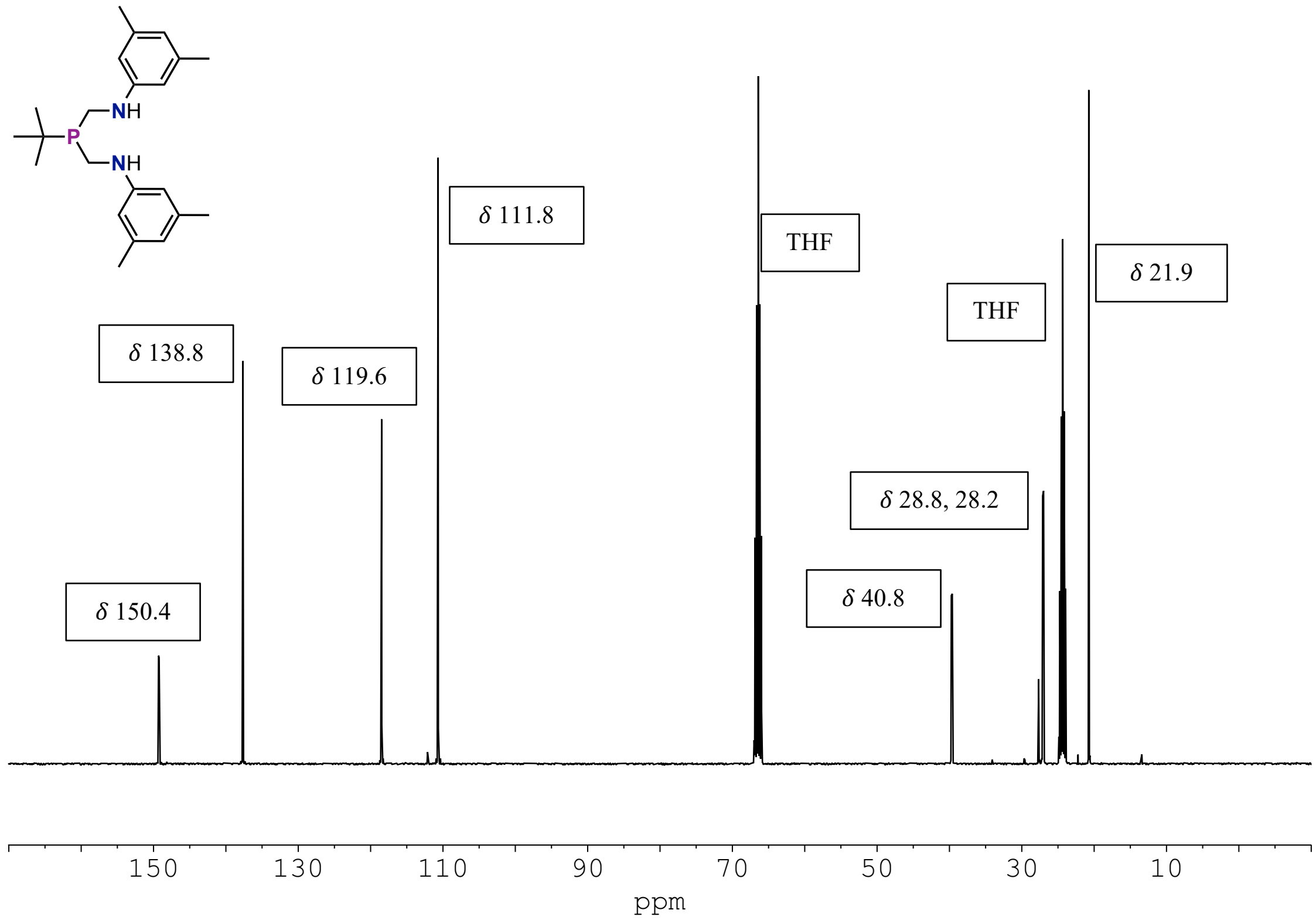

Figure S5. $^{13}C\{^1H\}$ NMR spectrum of **^{Ar}BiAMP^{tBu}** acquired in THF-d_8 at room temperature.

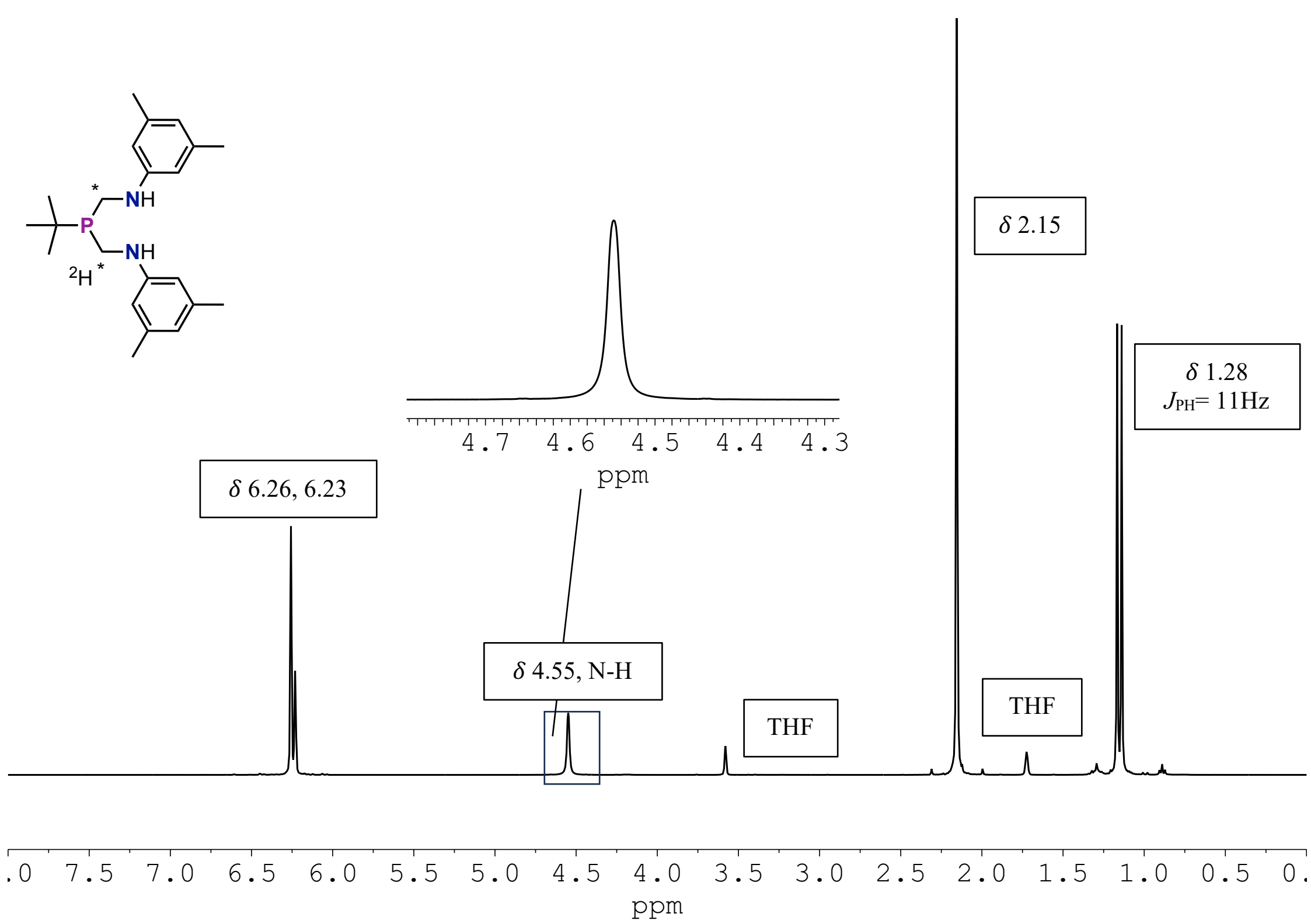

Figure S6. ^1H NMR spectrum of ***d*$_4$-^{Ar}BiAMP^{tBu}** acquired in THF-d_8 at room temperature.

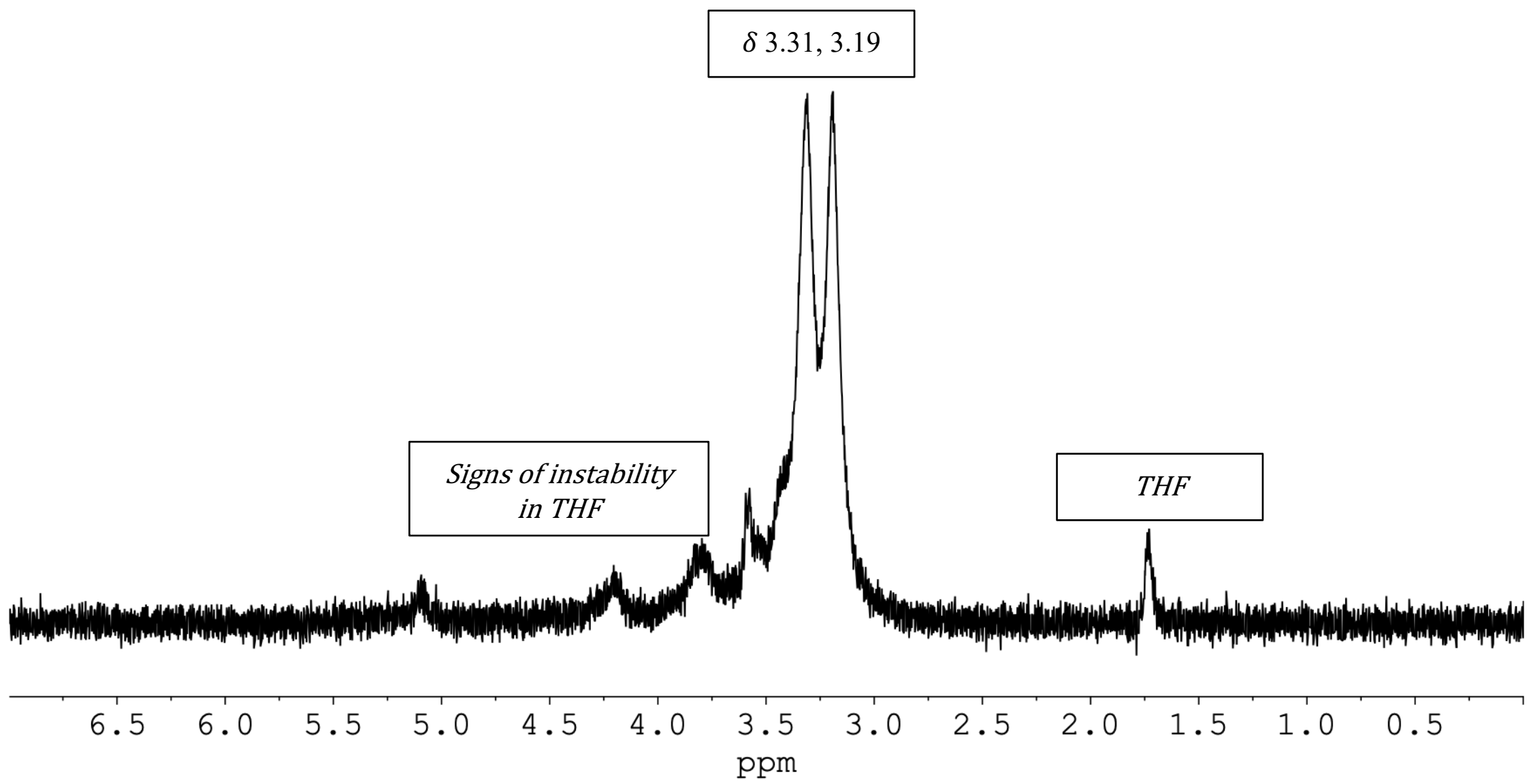

Figure S7. ^{2}H NMR spectrum of ***d*$_4$-^{Ar}BiAMP^{tBu}** acquired in THF at room temperature.

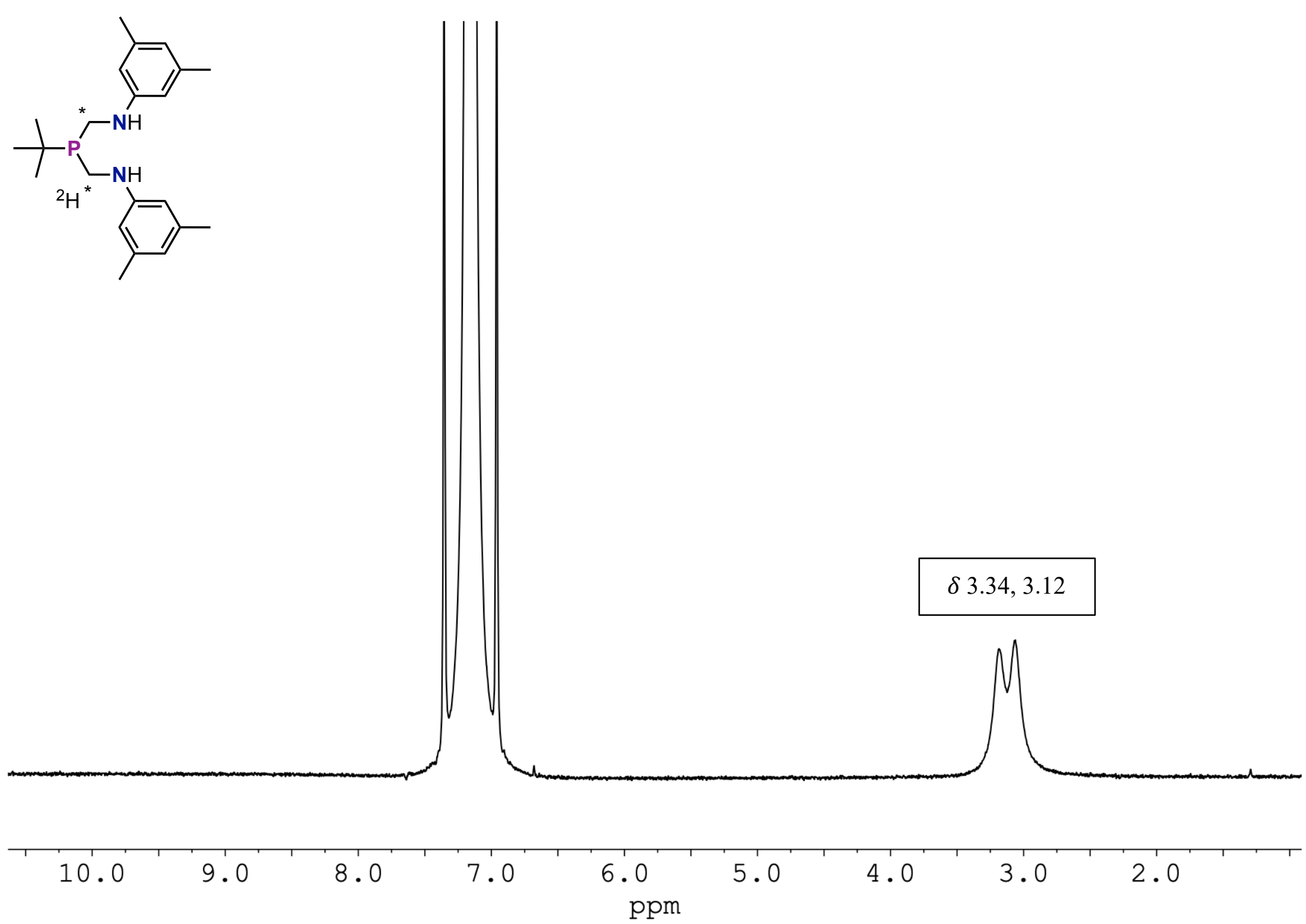

Figure S8. ^{2}H NMR spectrum of ***d*$_4$-^{Ar}BiAMP^{tBu}** acquired in C_6H_6 at room temperature.

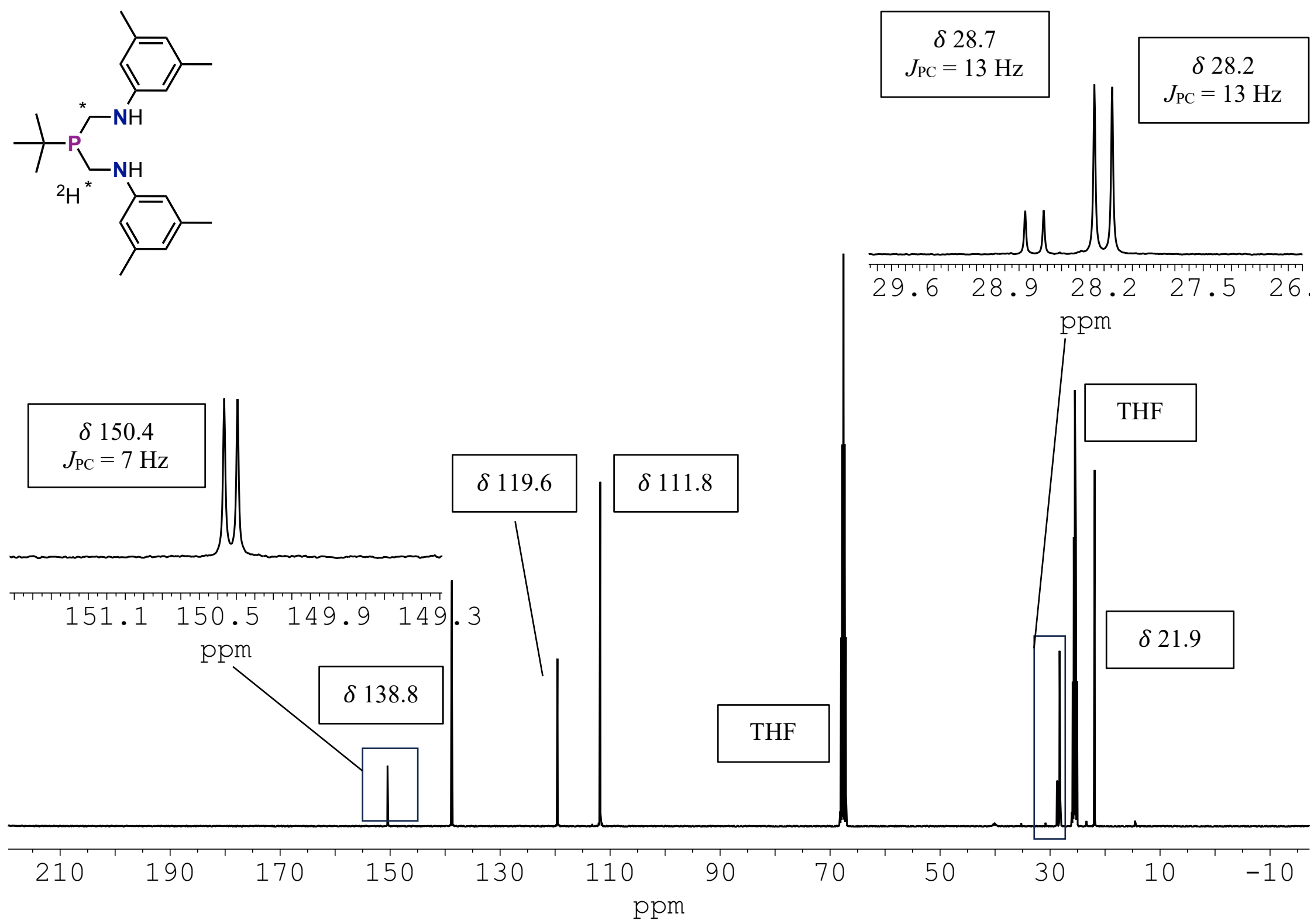

Figure S9. $^{13}C\{^{1}H\}$ NMR spectrum of ***d*₄-^{Ar}BiAMP^{tBu}** acquired in THF-d_8 at room temperature.

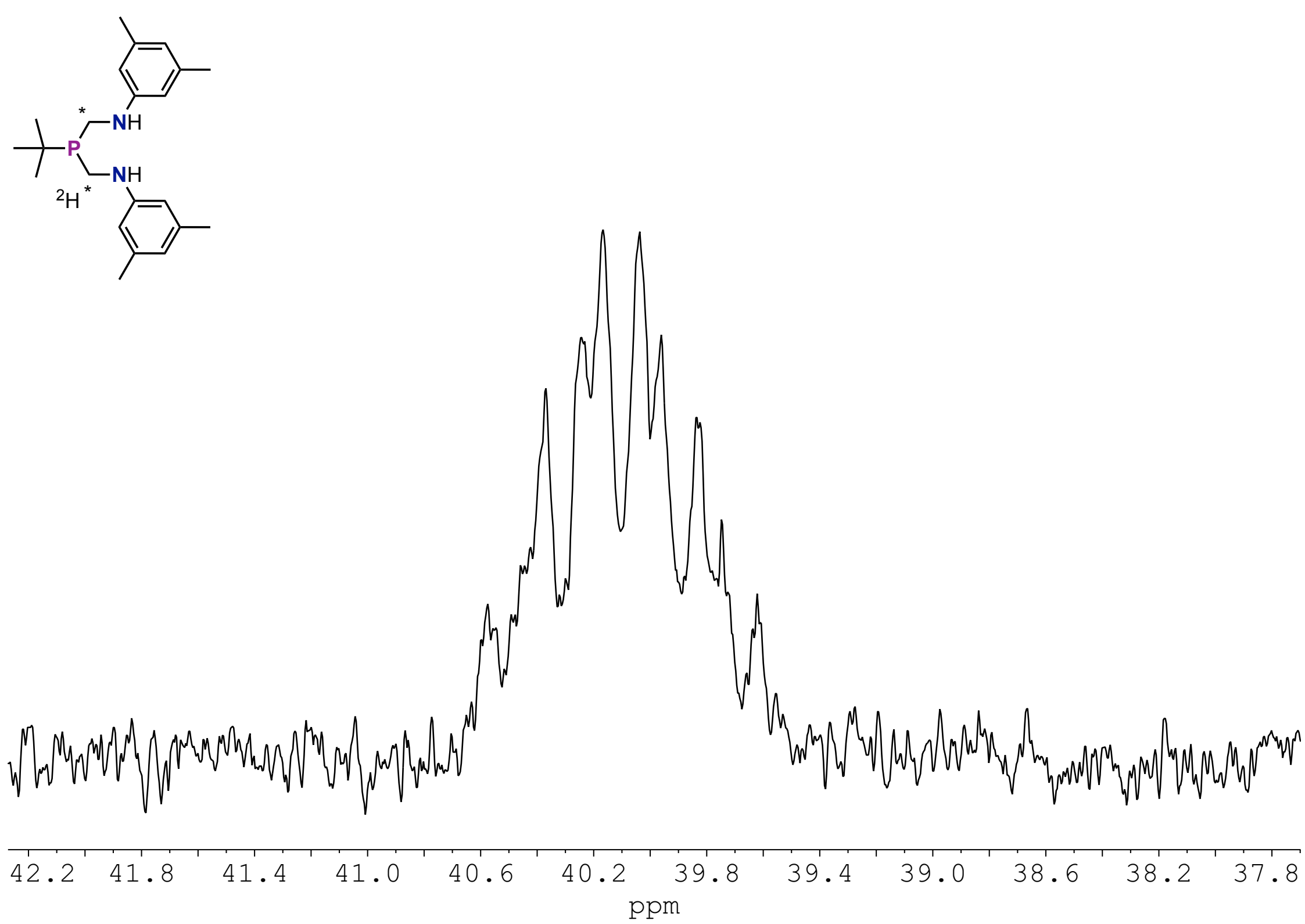

Figure S10. Expansion of the methylene resonance observed in the $^{13}C\{^{1}H\}$ NMR spectrum of ***d*₄-^{Ar}BiAMP^{tBu}** acquired in THF-d_8 at room temperature.

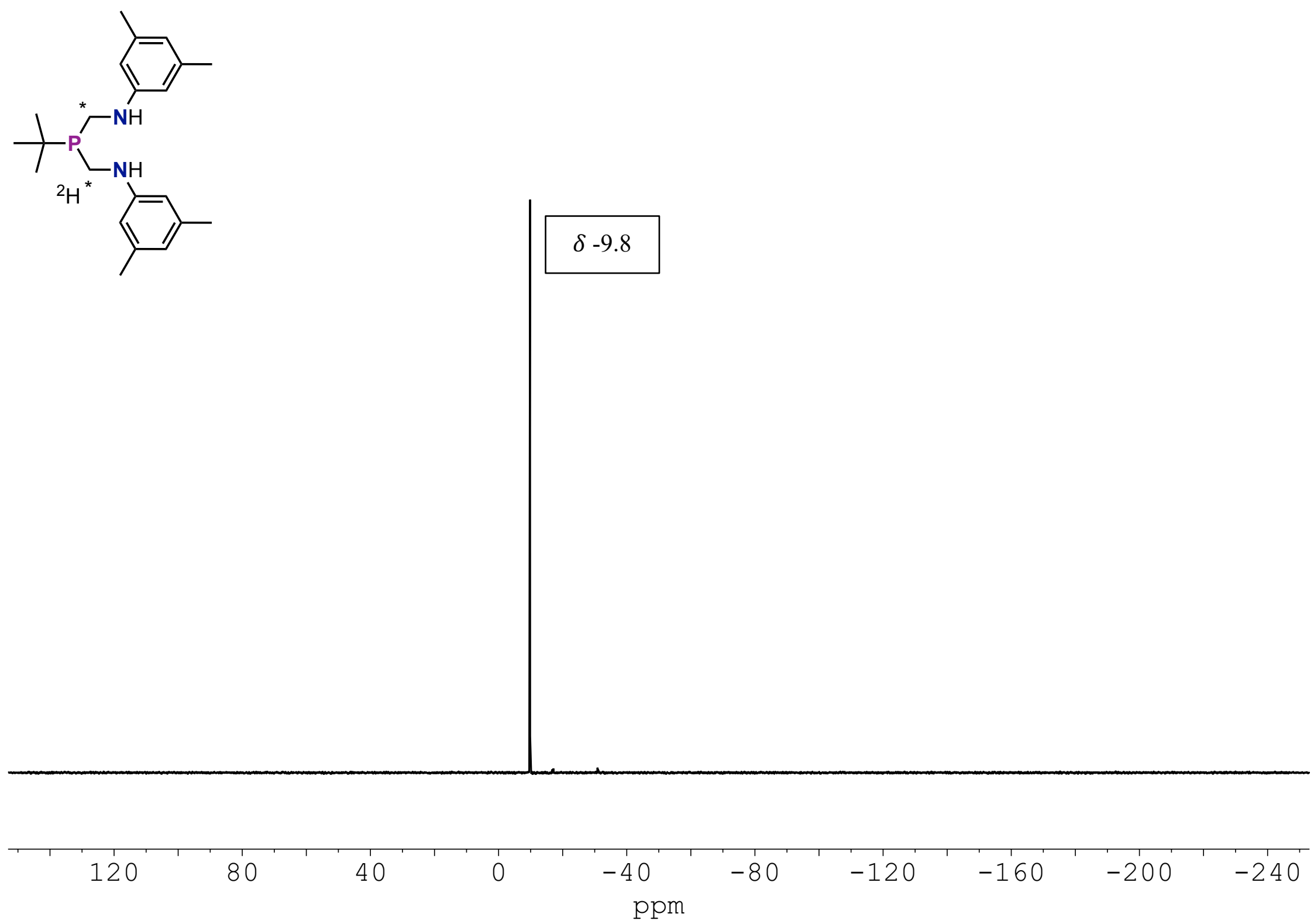

Figure S11. $^{31}P\{^1H\}$ NMR spectrum of ***d*₄-^{Ar}BiAMP^{tBu}** acquired in THF-d_8 at room temperature.

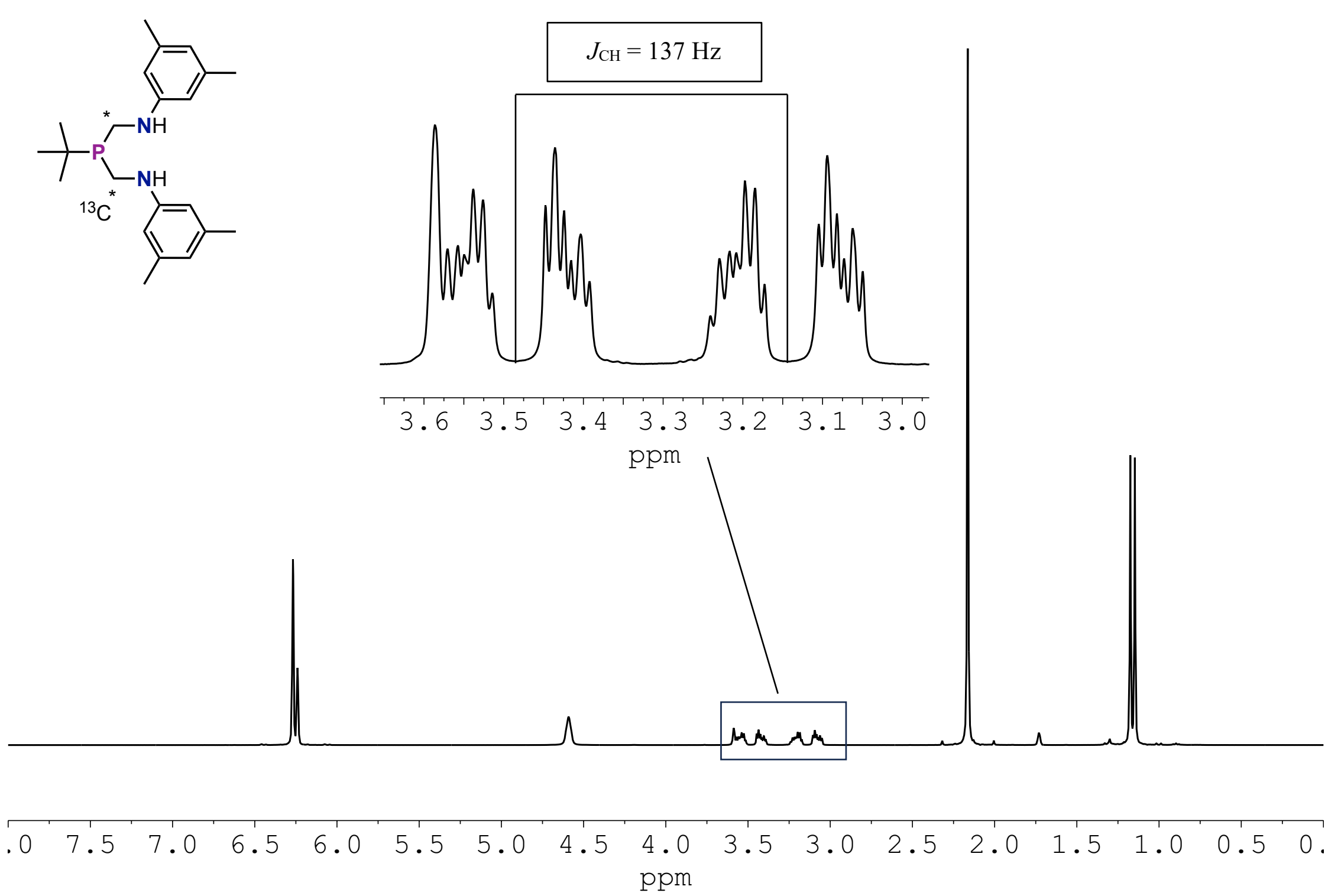

Figure S12. ^1H NMR spectrum of **$^{13}C_2$-^{Ar}BiAMP^{tBu}** acquired in THF-d_8 at room temperature.

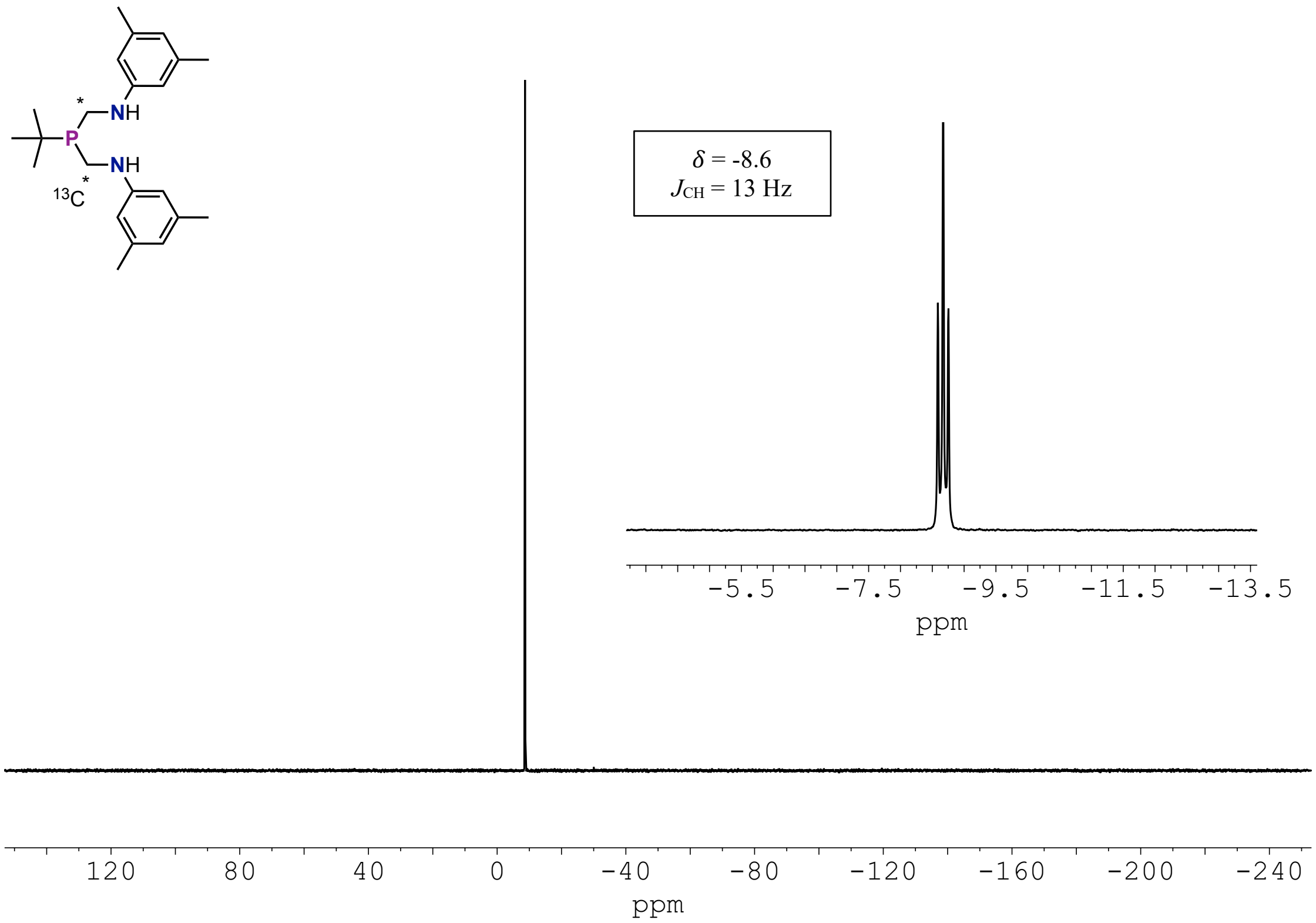

Figure S13. $^{31}P\{^{1}H\}$ NMR spectrum of **$^{13}C_2$-^{Ar}BiAMP^{tBu}** acquired in THF-d_8 at room temperature.

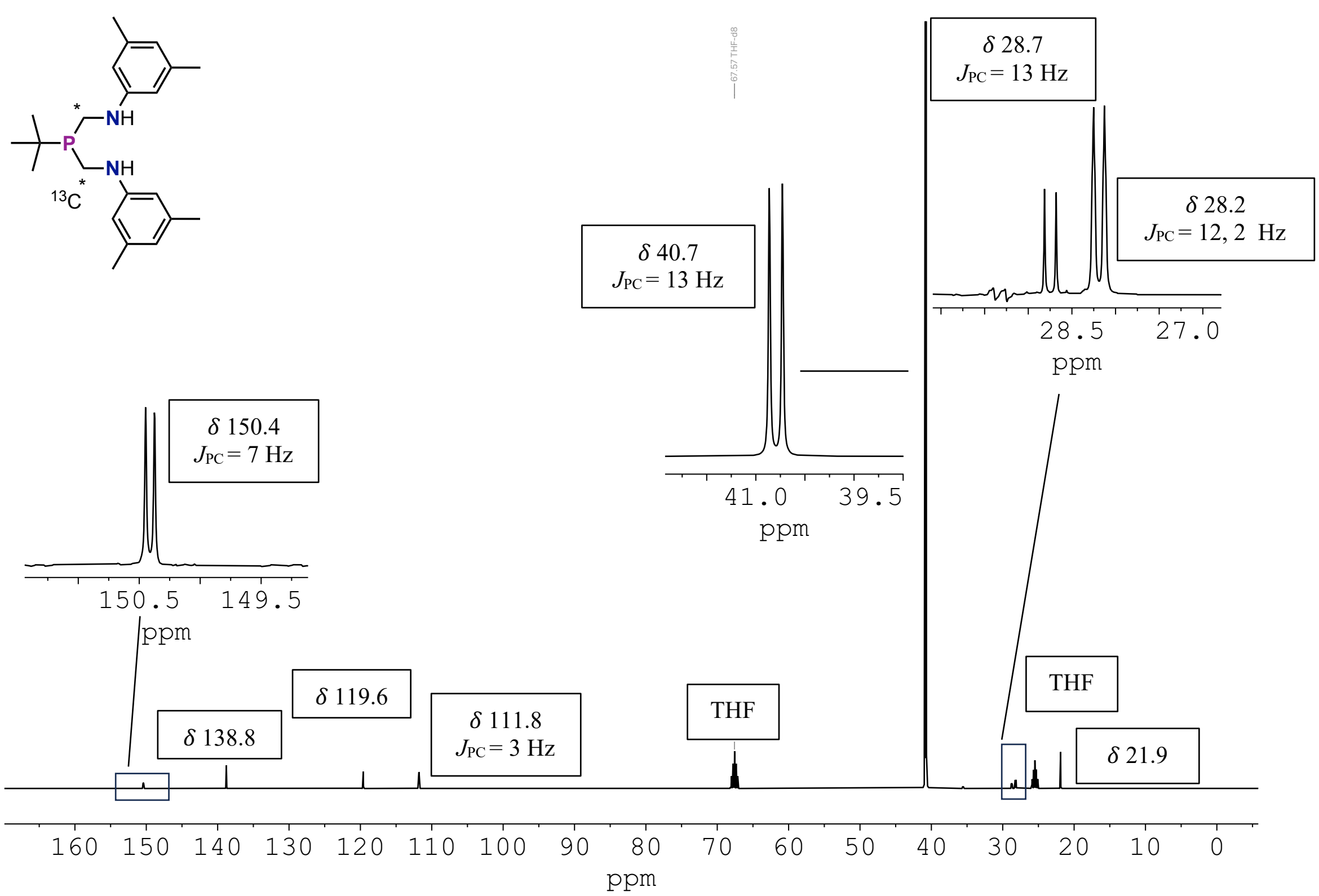

Figure S14. $^{13}C\{^{1}H\}$ NMR spectrum of **$^{13}C_2$-^{Ar}BiAMP^{tBu}** acquired in THF-d_8 at room temperature.

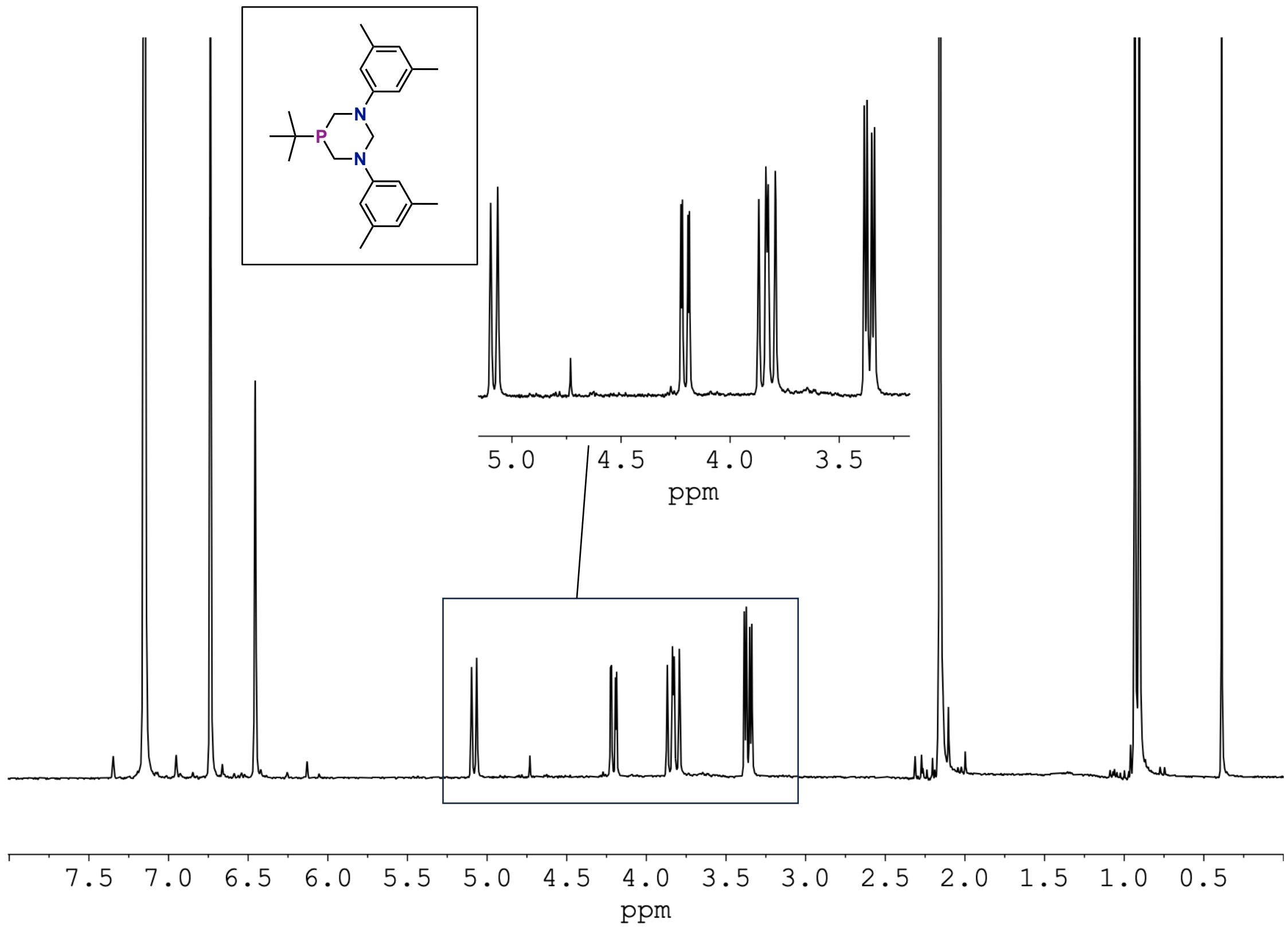

Figure S15. ^{1}H NMR spectrum of crude **^{Ar}BiAMP^{tBu}-CH$_2$** acquired in C_6D_6 at room temperature directly from the crude solid without further purification.

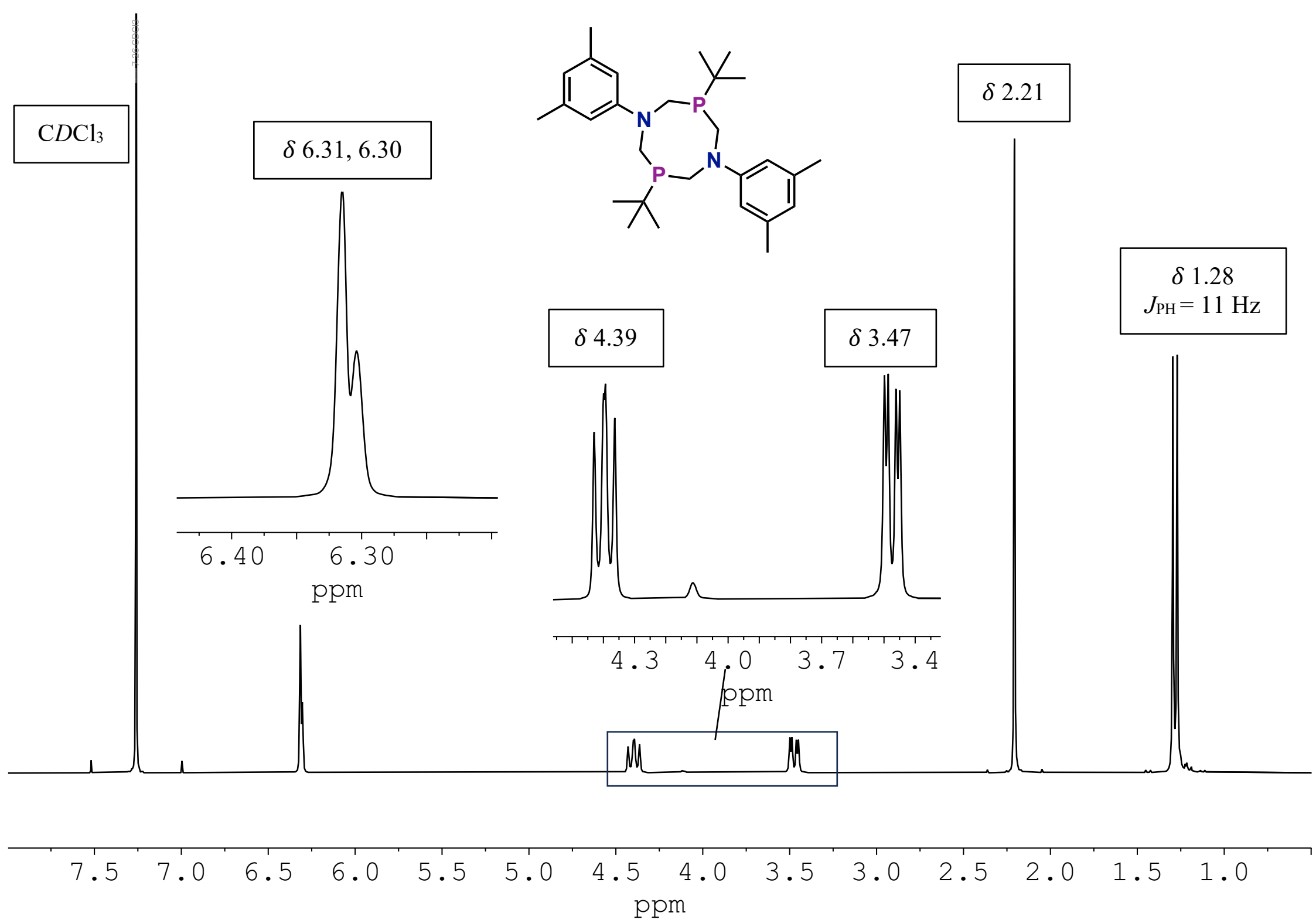

Figure S16. ^{1}H NMR spectrum of **(^{tBu}P$_2$N$_2$^{Ar})**, acquired in $CDCl_3$ at room temperature.

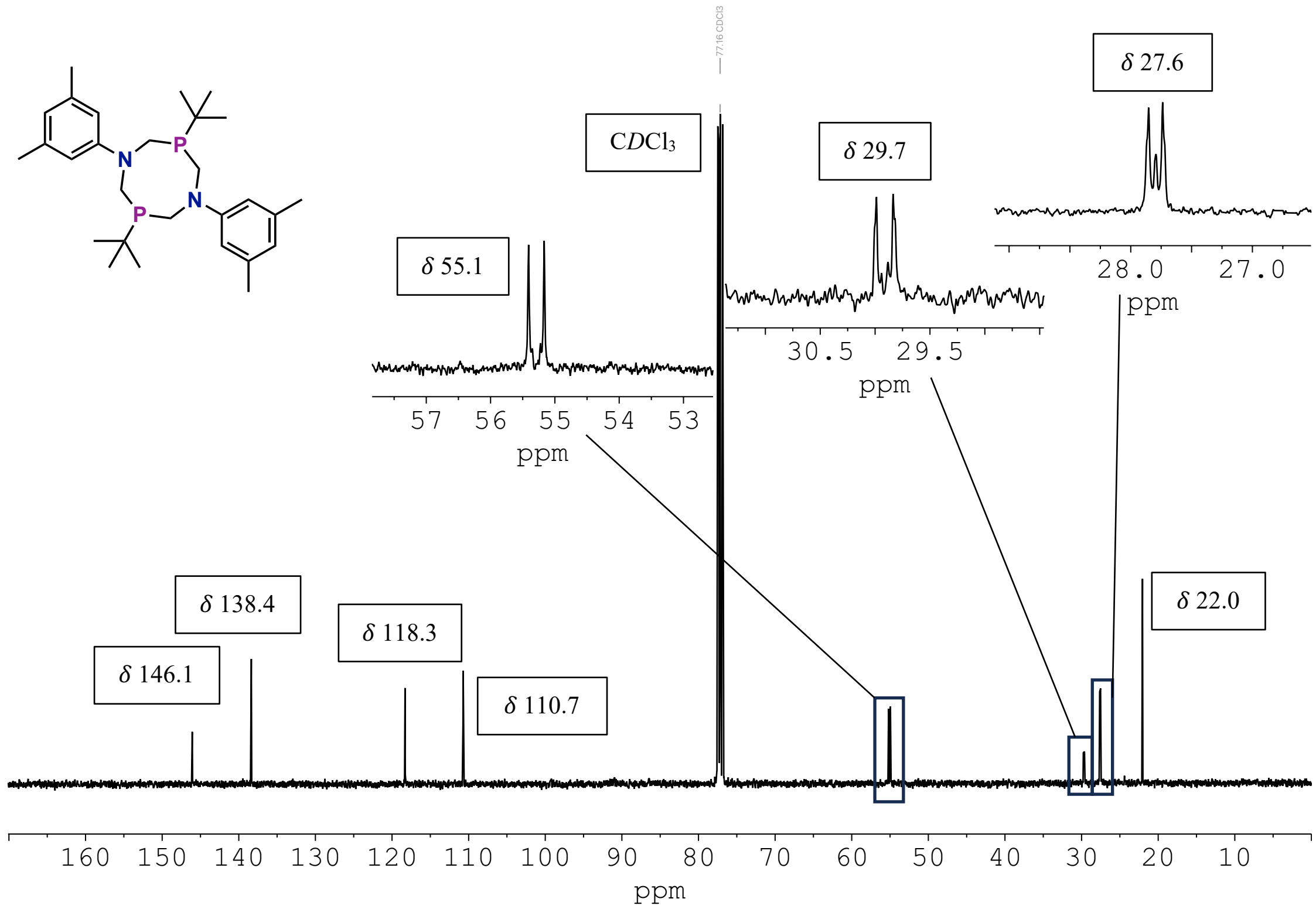

Figure S17. ^{13}C NMR spectrum of (**$^{tBu}P_2N_2^{Ar}$**) acquired in C*D*Cl$_3$ at room temperature.

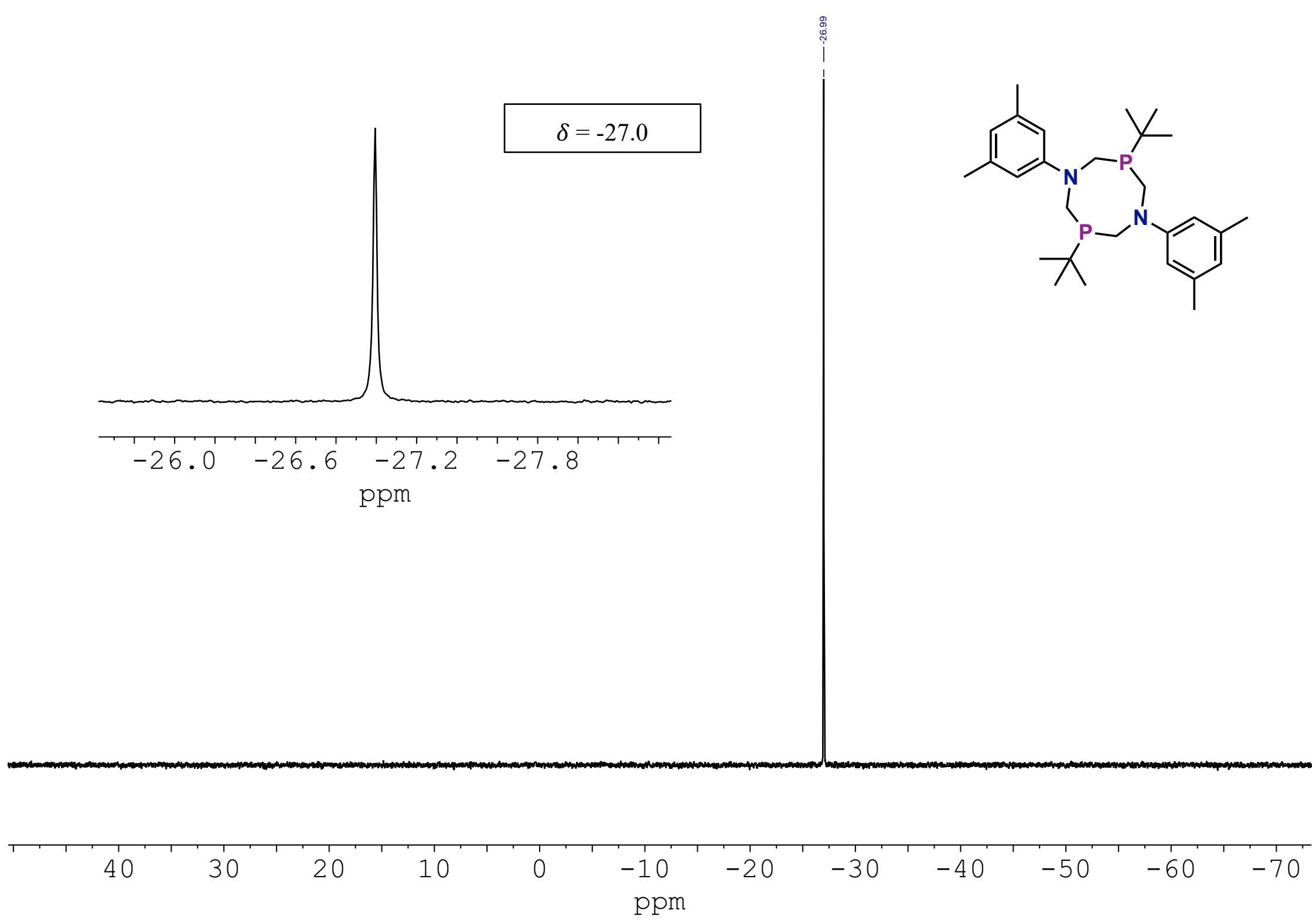

Figure S18. ^{31}P NMR spectrum of (**$^{tBu}P_2N_2^{Ar}$**) acquired in C*D*Cl$_3$ at room temperature.

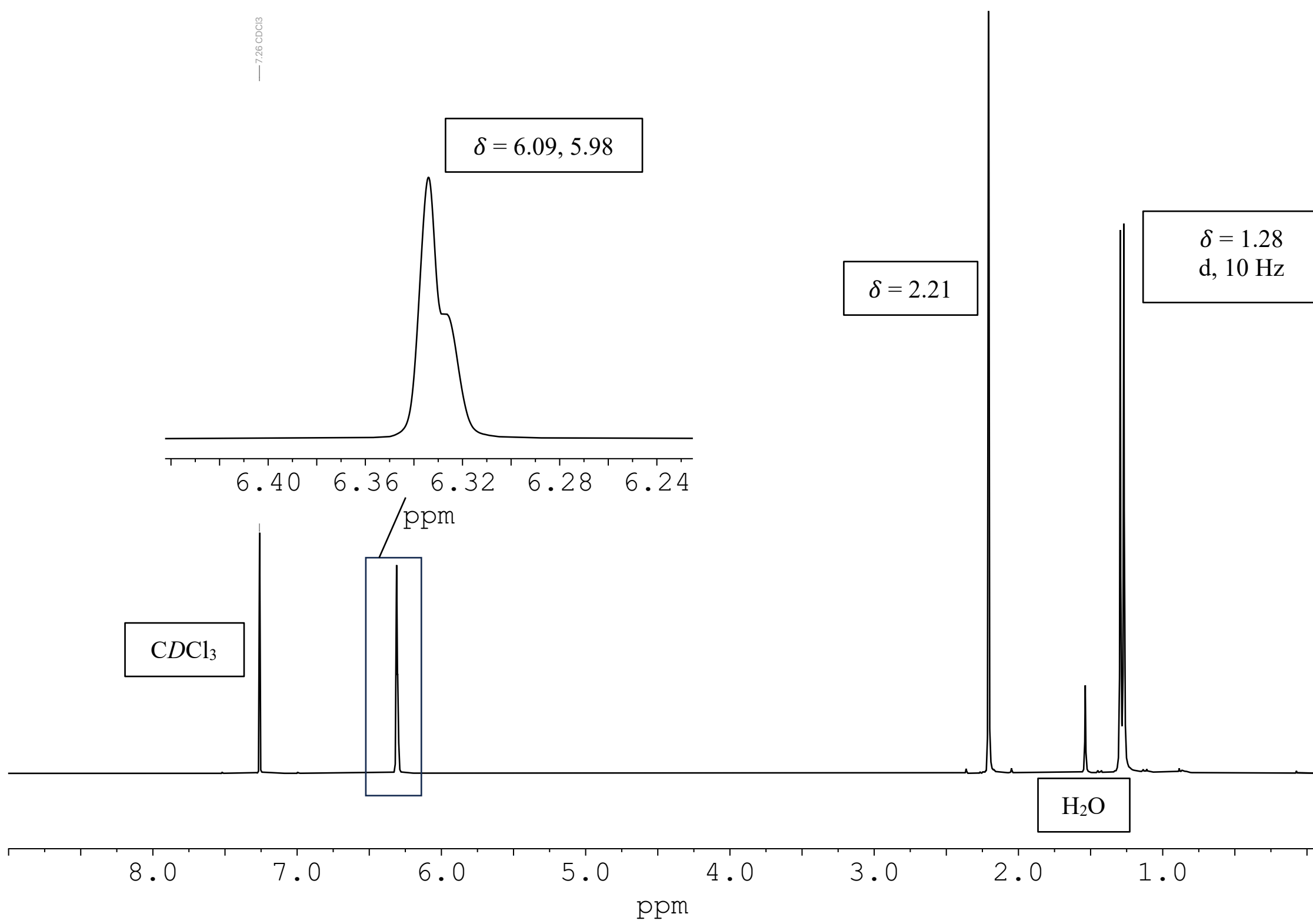

Figure S19. ^{1}H NMR spectrum of **(d_8- $^{tBu}P_2N_2^{Ar}$)** acquired in C*D*Cl_3 at room temperature.

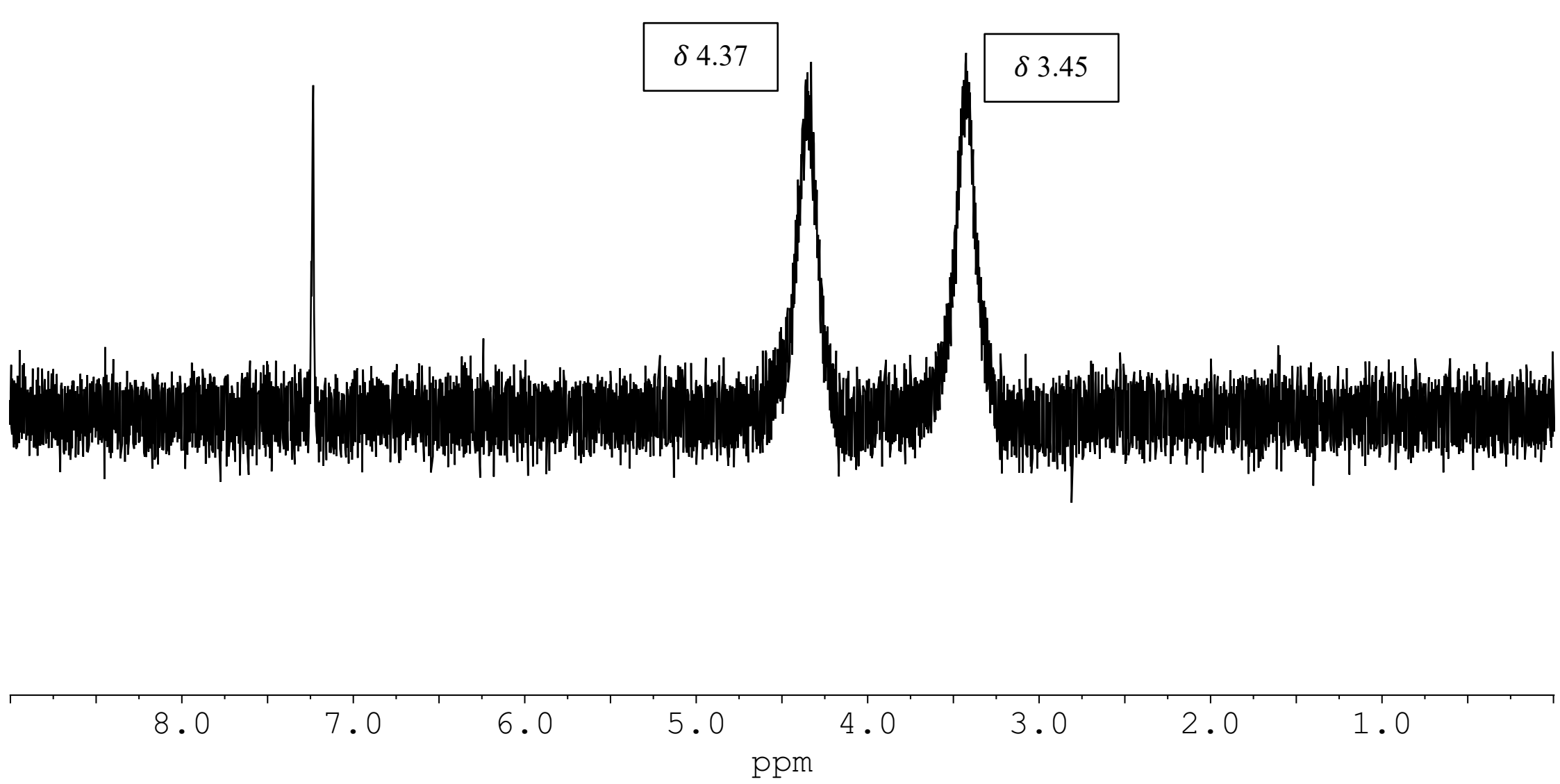

Figure S20. ^{2}H NMR spectrum of **(d_8- $^{tBu}P_2N_2^{Ar}$)** acquired in C*D*Cl_3 at room temperature.

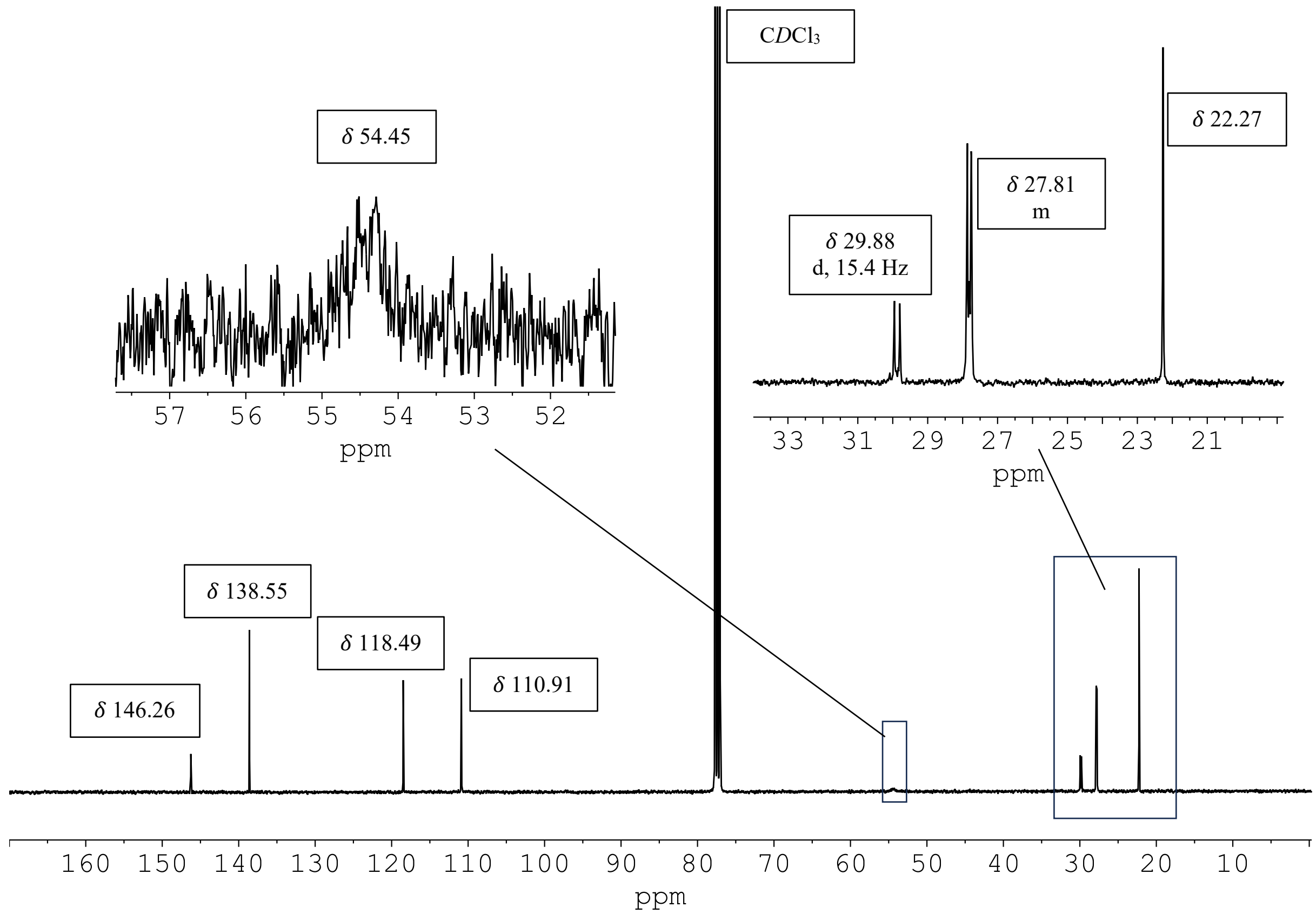

Figure S21. ^{13}C NMR spectrum of **(d$_8$- ^{tBu}P$_2$N$_2$^{Ar})** acquired in C*D*Cl$_3$ at room temperature.

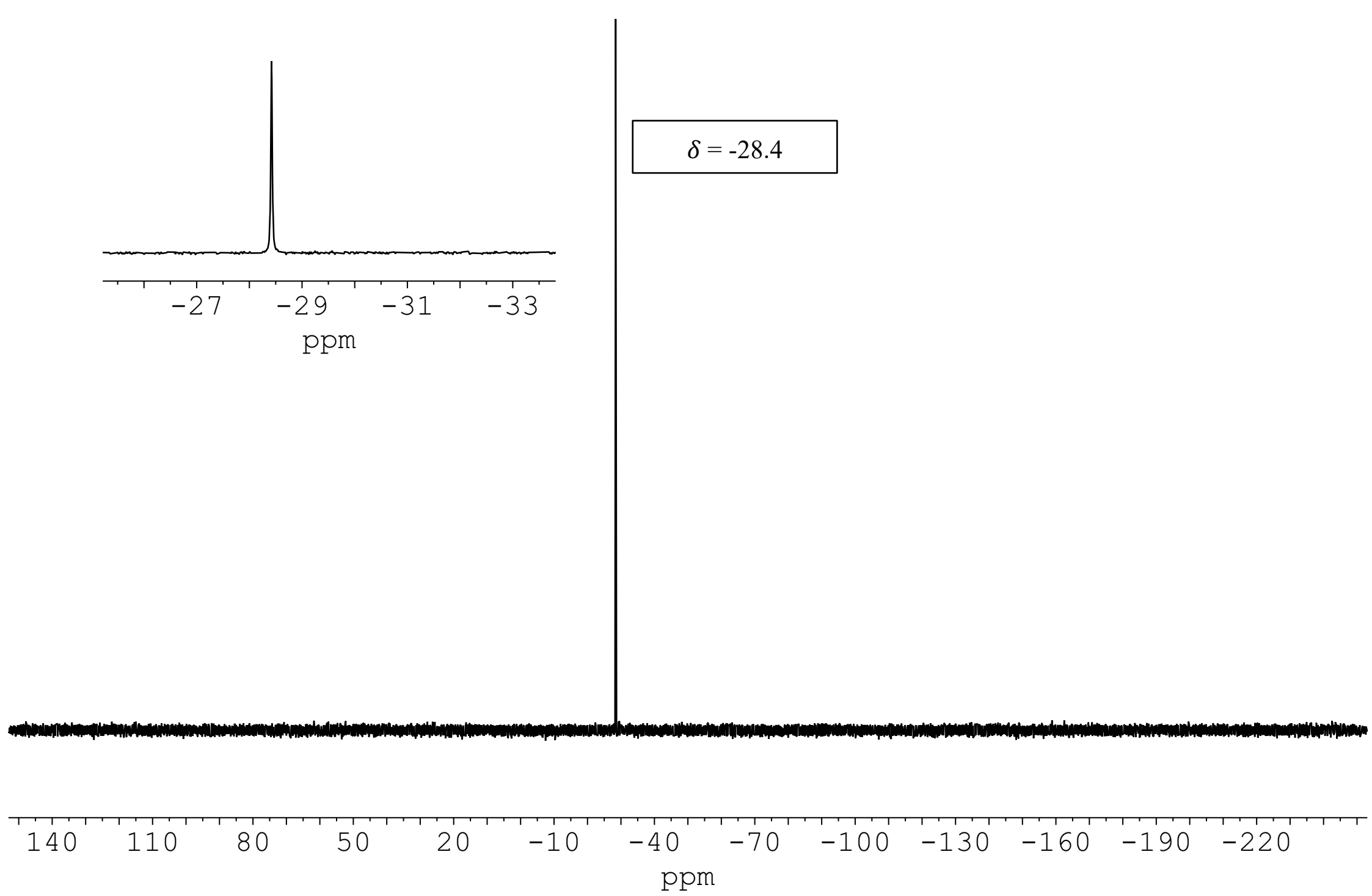

Figure S22. ^{31}P NMR spectrum of **(d$_8$- ^{tBu}P$_2$N$_2$^{Ar})** acquired in C*D*Cl$_3$ at room temperature.

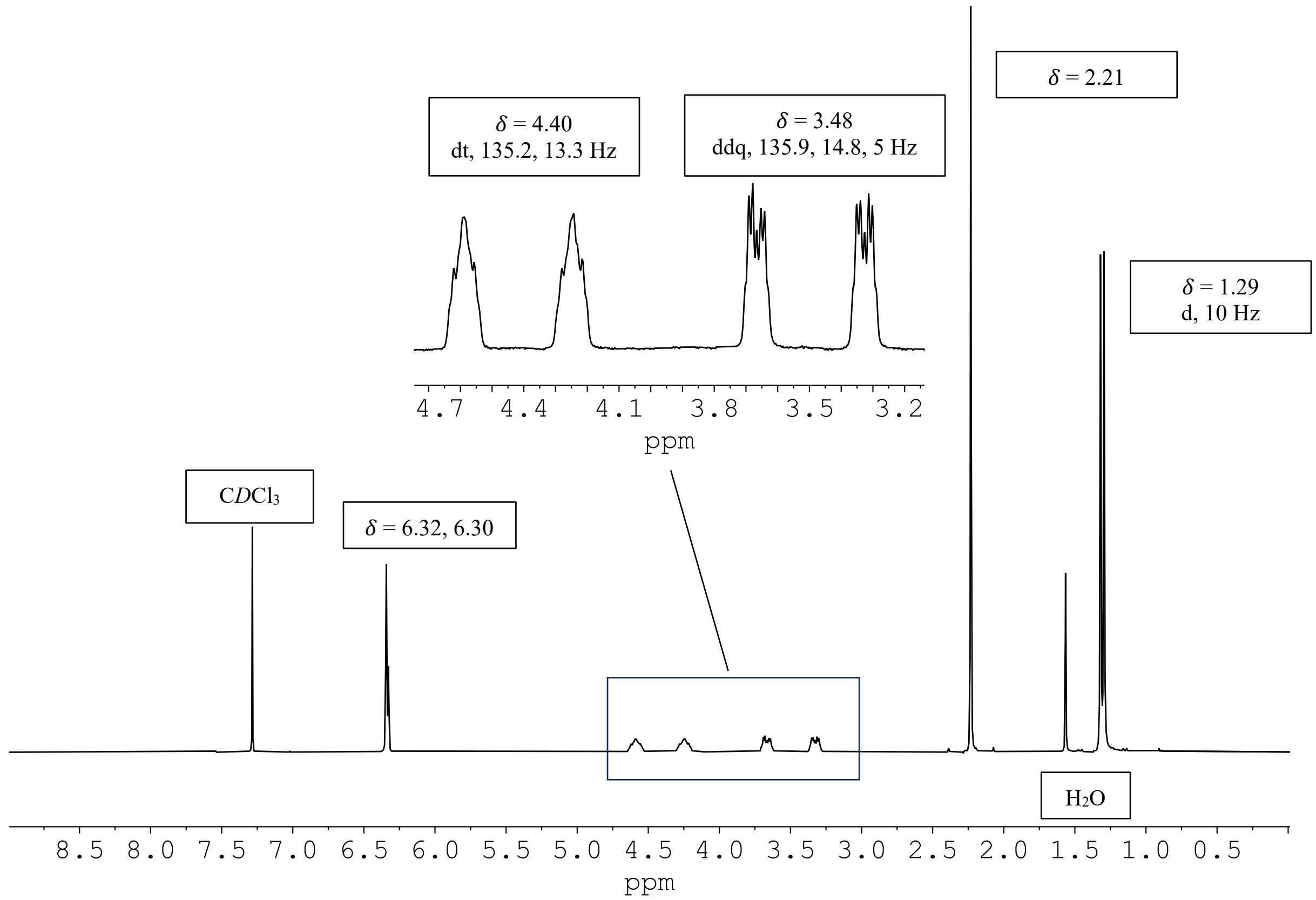

Figure S23. ^{1}H NMR spectrum of (**^{13}C-$^{tBu}P_2N_2^{Ar}$**) acquired in C*D*Cl$_3$ at room temperature with an insert of an expansion of the methylene region of the spectrum.

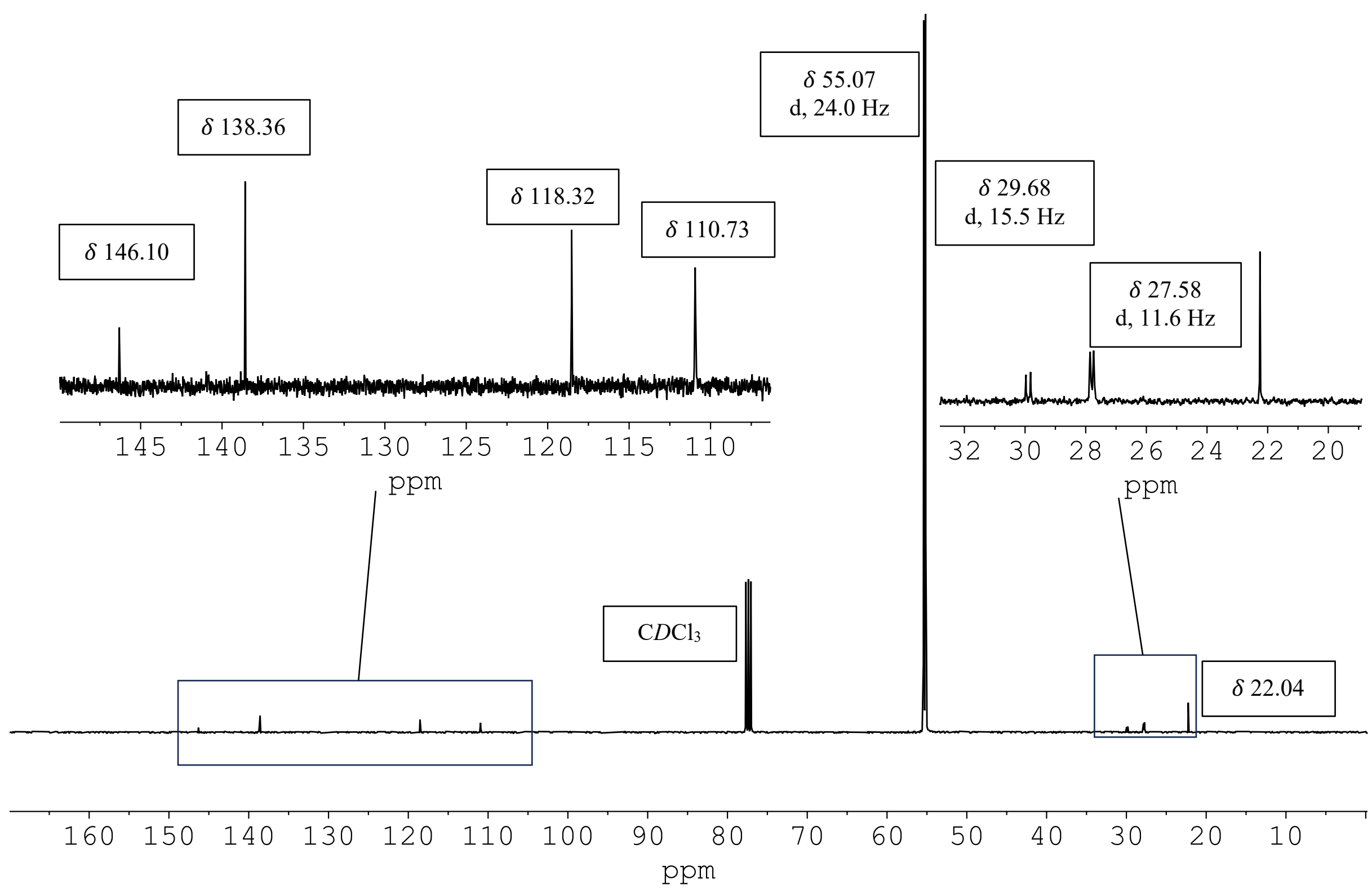

Figure S24. ^{13}C NMR spectrum of (**^{13}C-$^{tBu}P_2N_2^{Ar}$**) acquired in C*D*Cl$_3$ at room temperature.

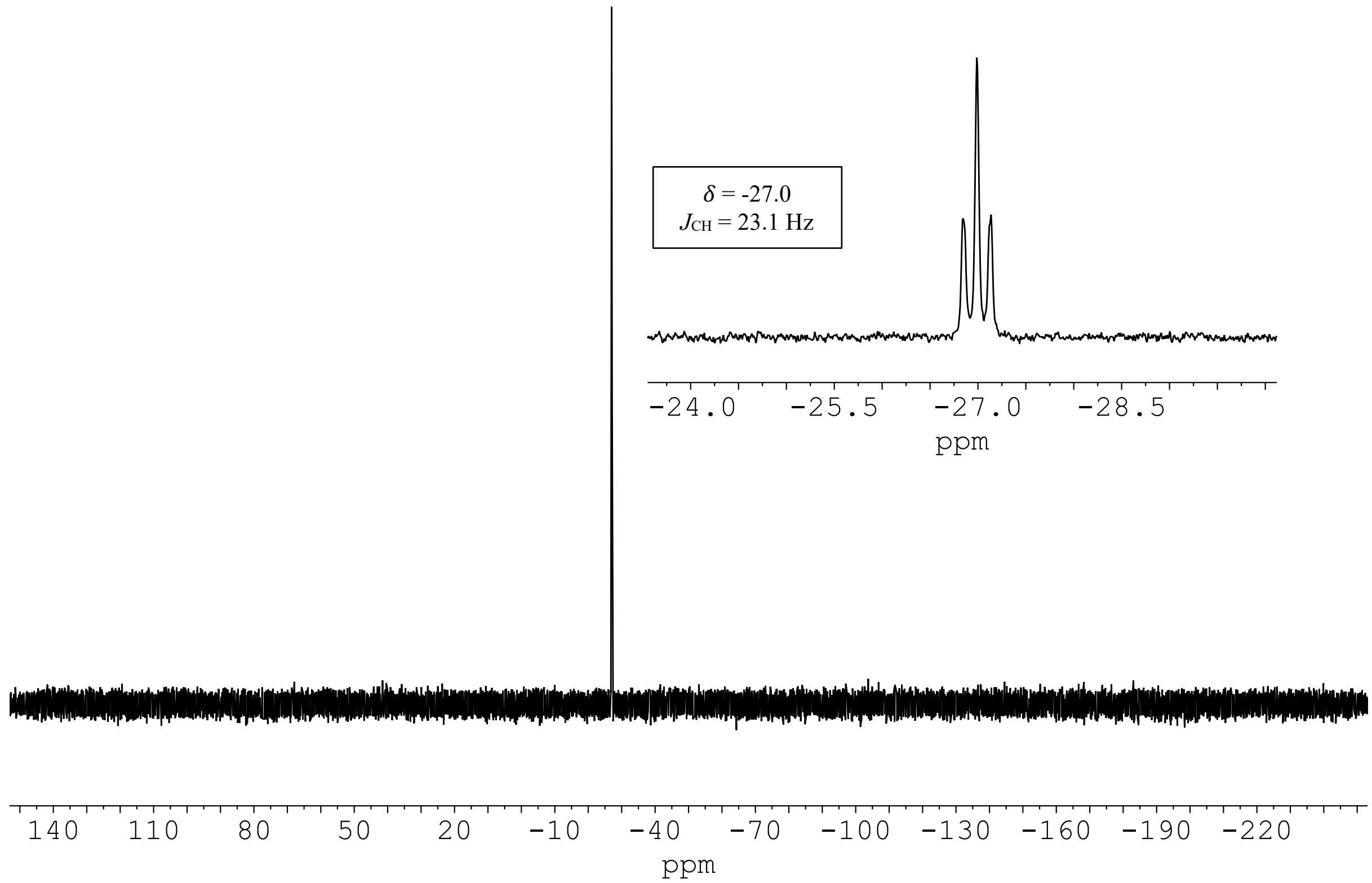

Figure S25. ^{31}P NMR spectrum of **(^{13}C- ^{tBu}P$_2$N$_2$^{Ar})** acquired in C*D*Cl$_3$ at room temperature.

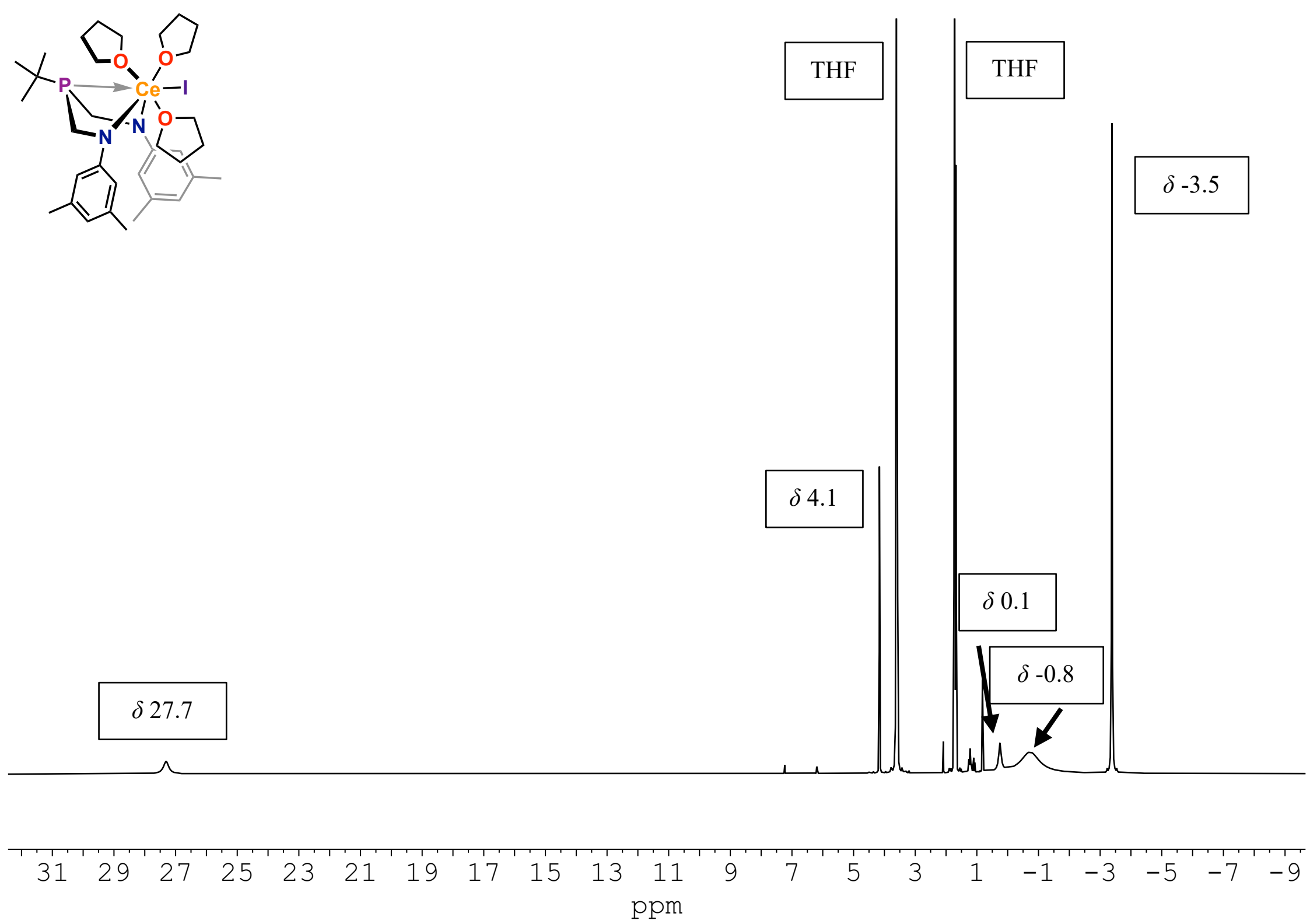

Figure S26. ^{1}H NMR spectrum of **CeI(^{Ar}BiAMP^{tBu})(thf)$_3$** acquired in THF-*d*$_8$ at room temperature.

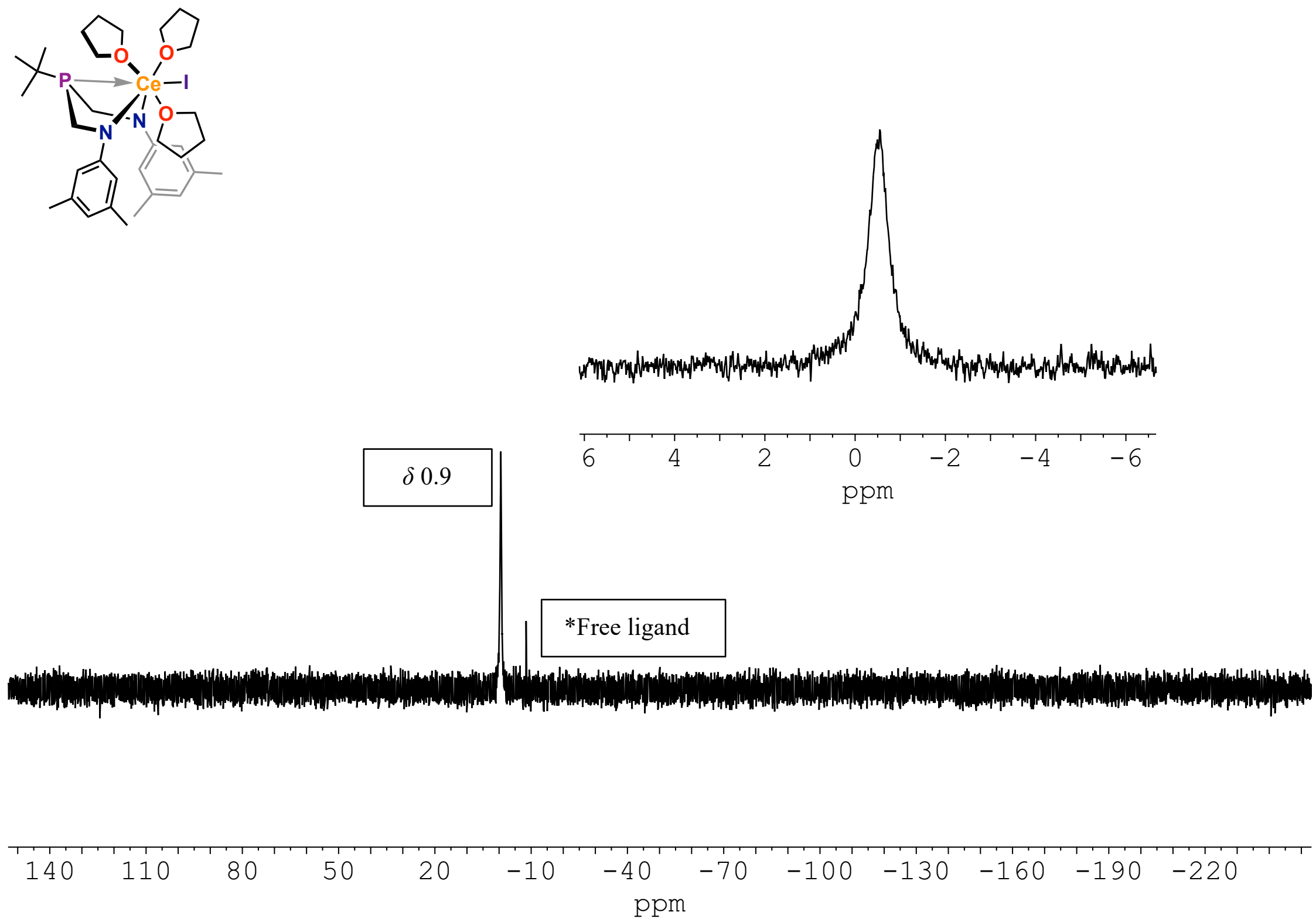

Figure S27. ^{31}P{^{1}H} NMR spectrum of **CeI(^{Ar}BiAMP^{tBu})(thf)$_3$** acquired in THF-d_8 at room temperature.

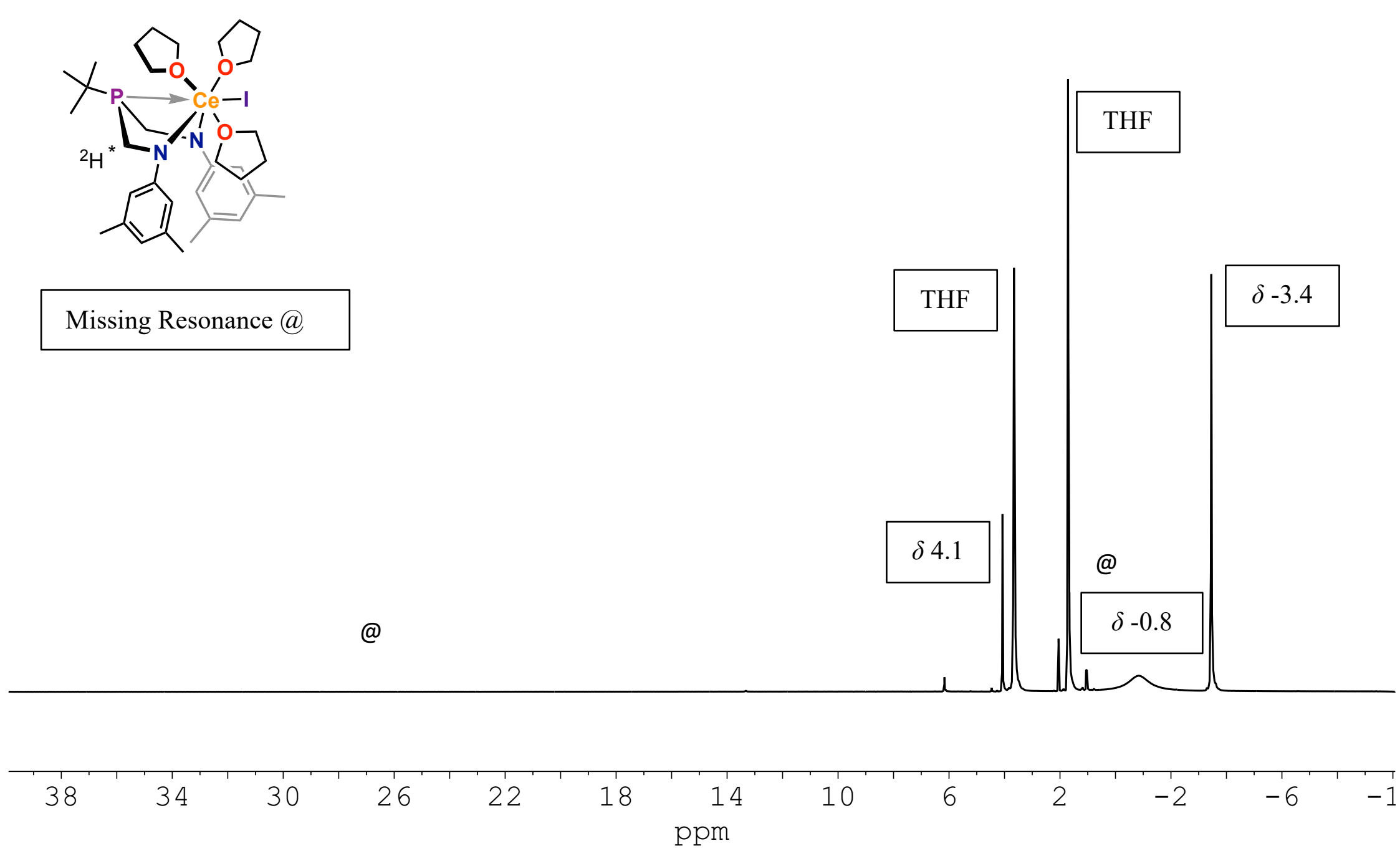

Figure S28. ^{1}H NMR spectrum of **CeI(d_4-^{Ar}BiAMP^{tBu})(thf)$_3$** acquired in THF-d_8 at room temperature.

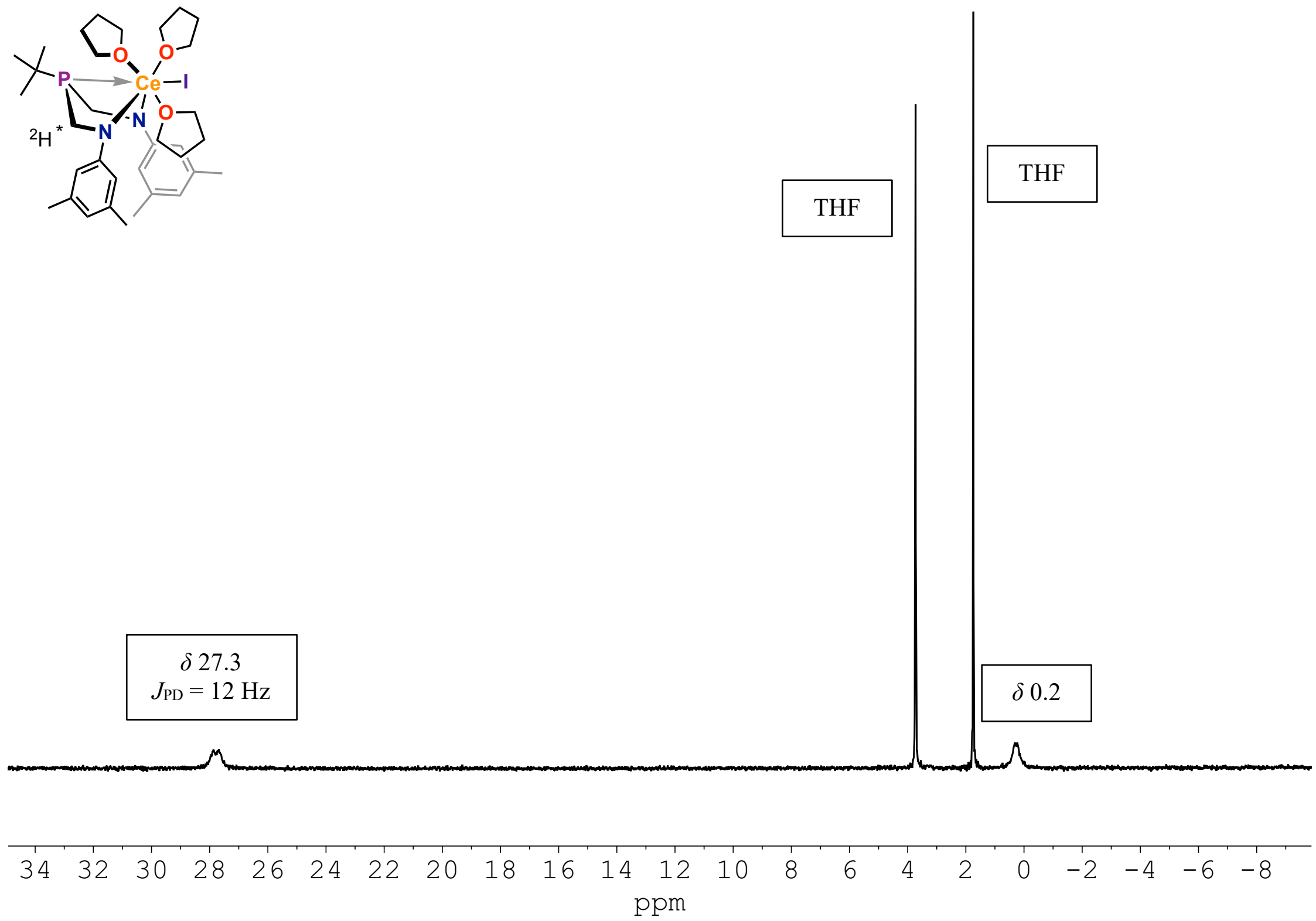

Figure S29. ^{2}H NMR spectrum of **CeI(*d*$_4$-^{Ar}BiAMP^{tBu})(thf)$_3$** acquired in THF at room temperature.

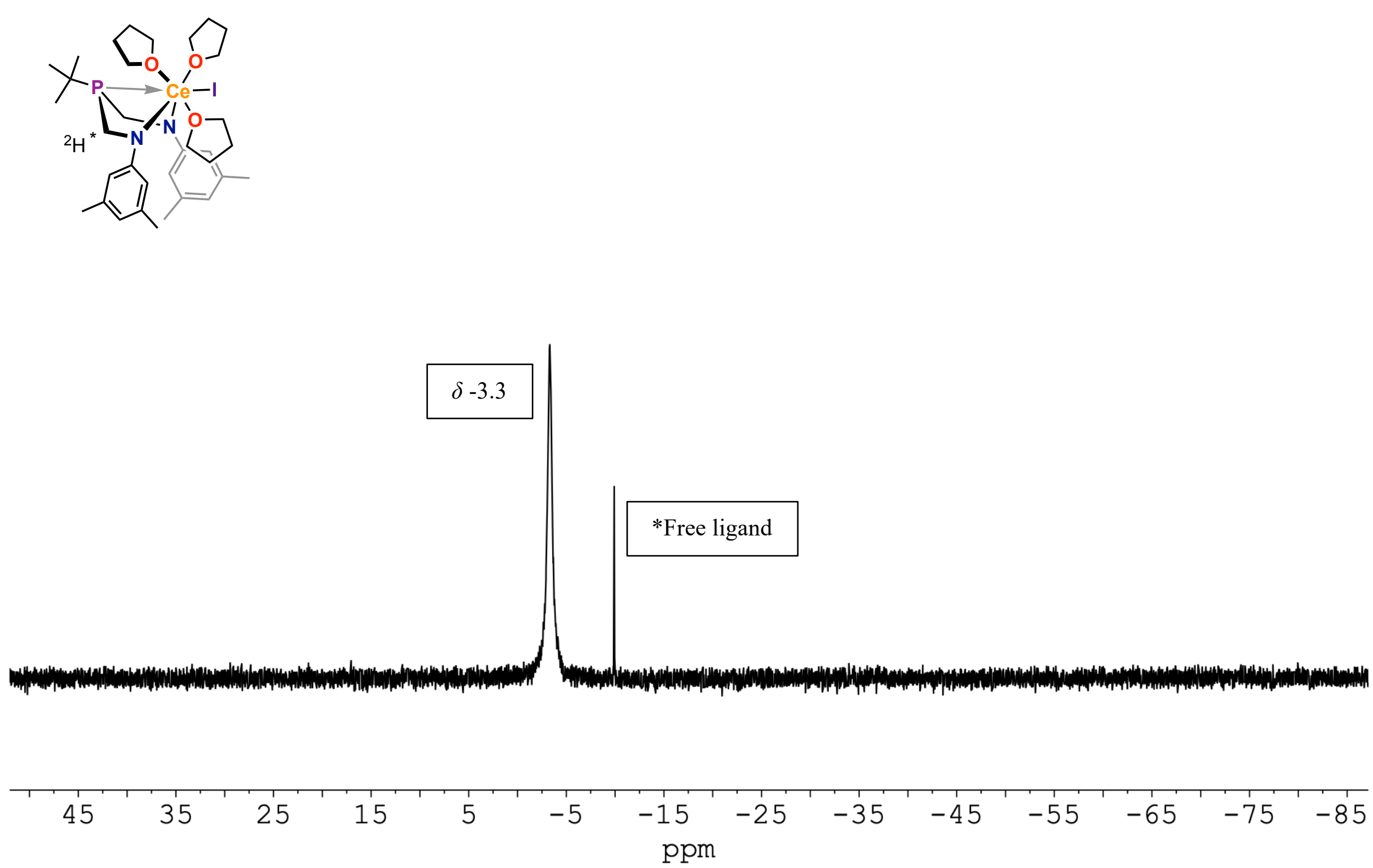

Figure S30. $^{31}P\{^{1}H\}$ NMR spectrum of **CeI(*d*$_4$-^{Ar}BiAMP^{tBu})(thf)$_3$** acquired in THF-*d*$_8$ at room temperature.

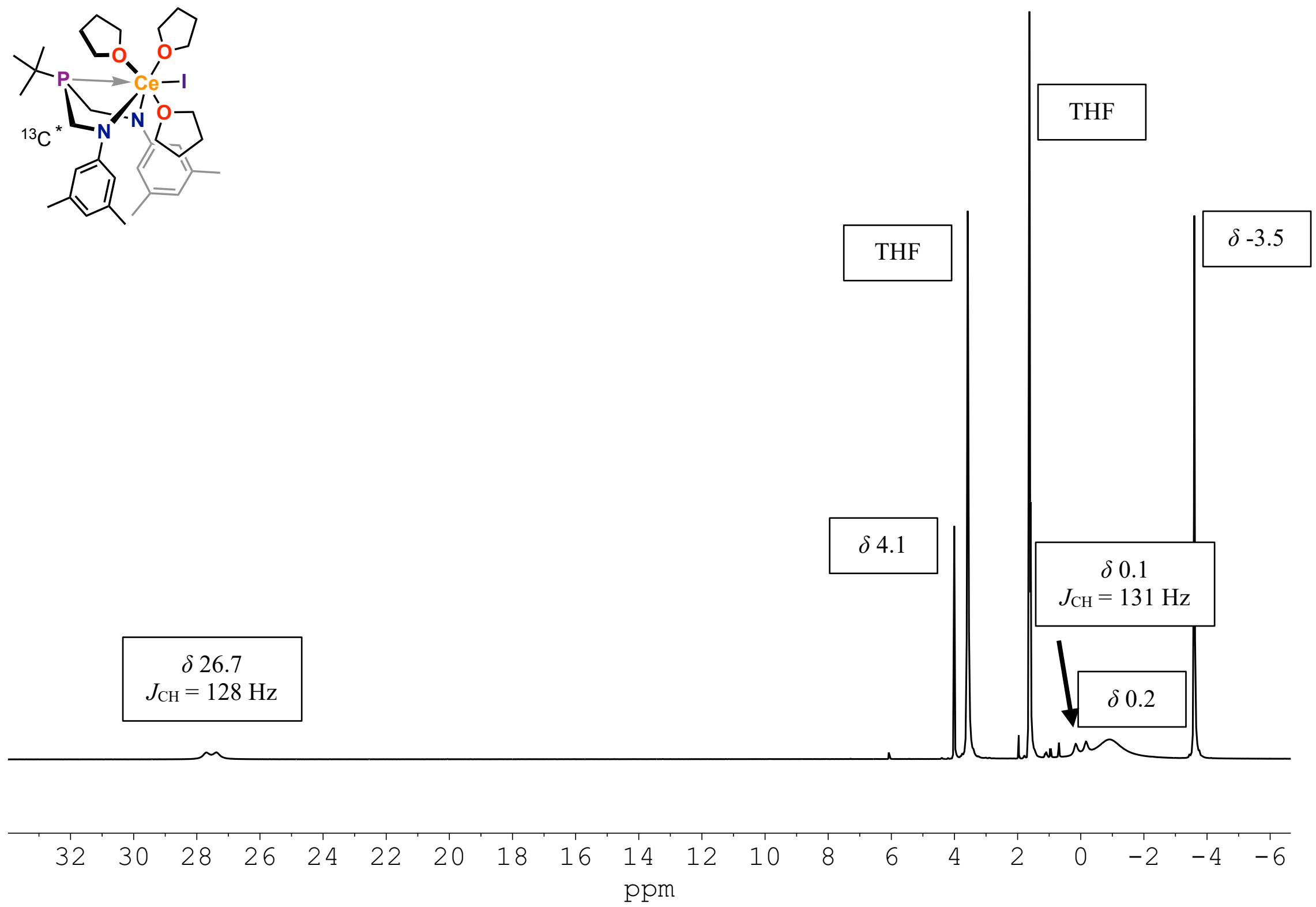

Figure S31. ^{1}H NMR spectrum of **CeI($^{13}C_2$-^{Ar}BiAMP^{tBu})(thf)$_3$** acquired in THF-d_8 at room temperature.

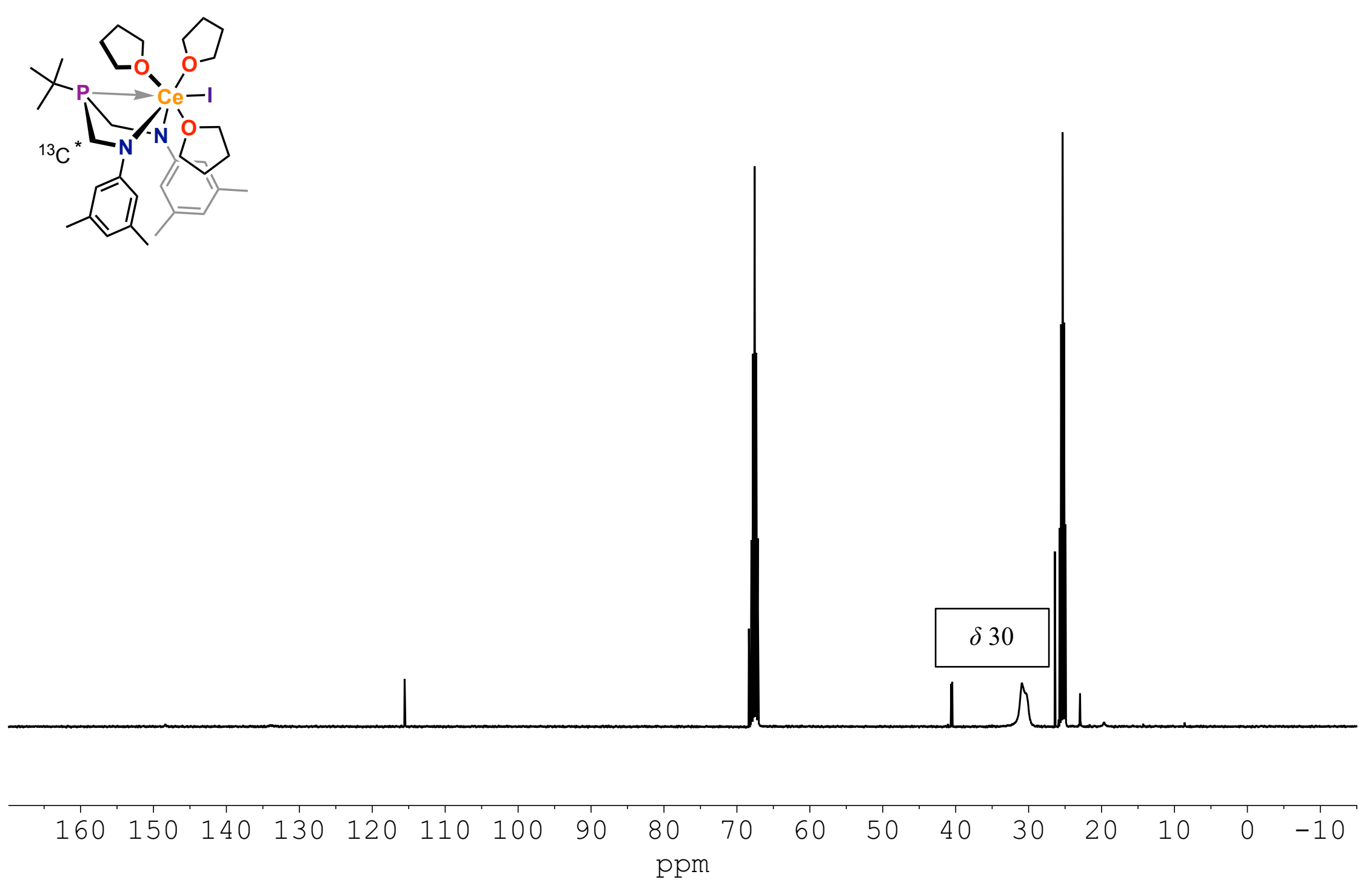

Figure S32. $^{13}C\{^1H\}$ NMR spectrum of **CeI($^{13}C_2$-^{Ar}BiAMP^{tBu})(thf)$_3$** acquired in THF-d_8 at room temperature.

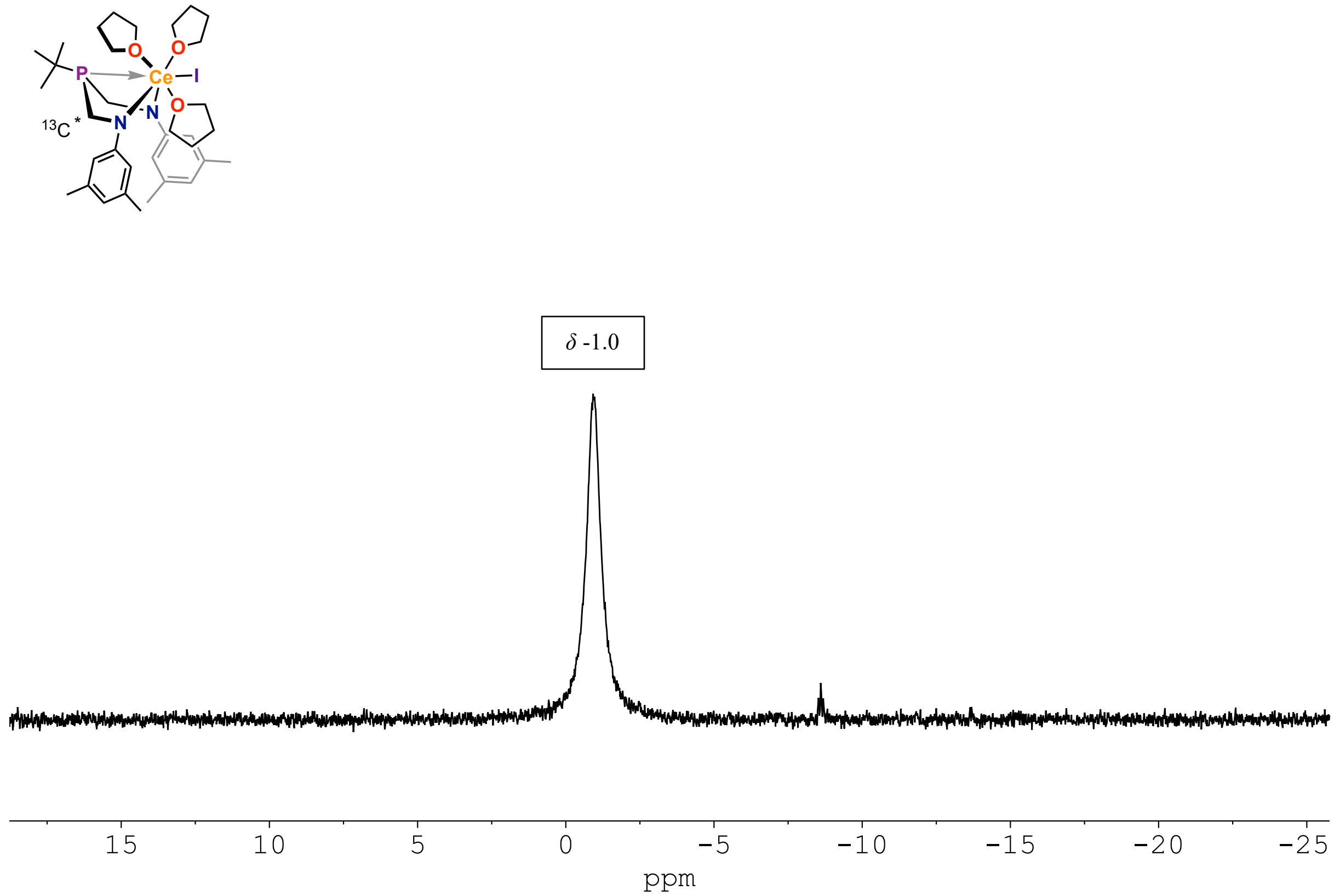

Figure S33. $^{31}P\{^{1}H\}$ NMR spectrum of **CeI($^{13}C_2$-^{Ar}BiAMP^{tBu})(thf)$_3$** acquired in THF-d_8 at room temperature.

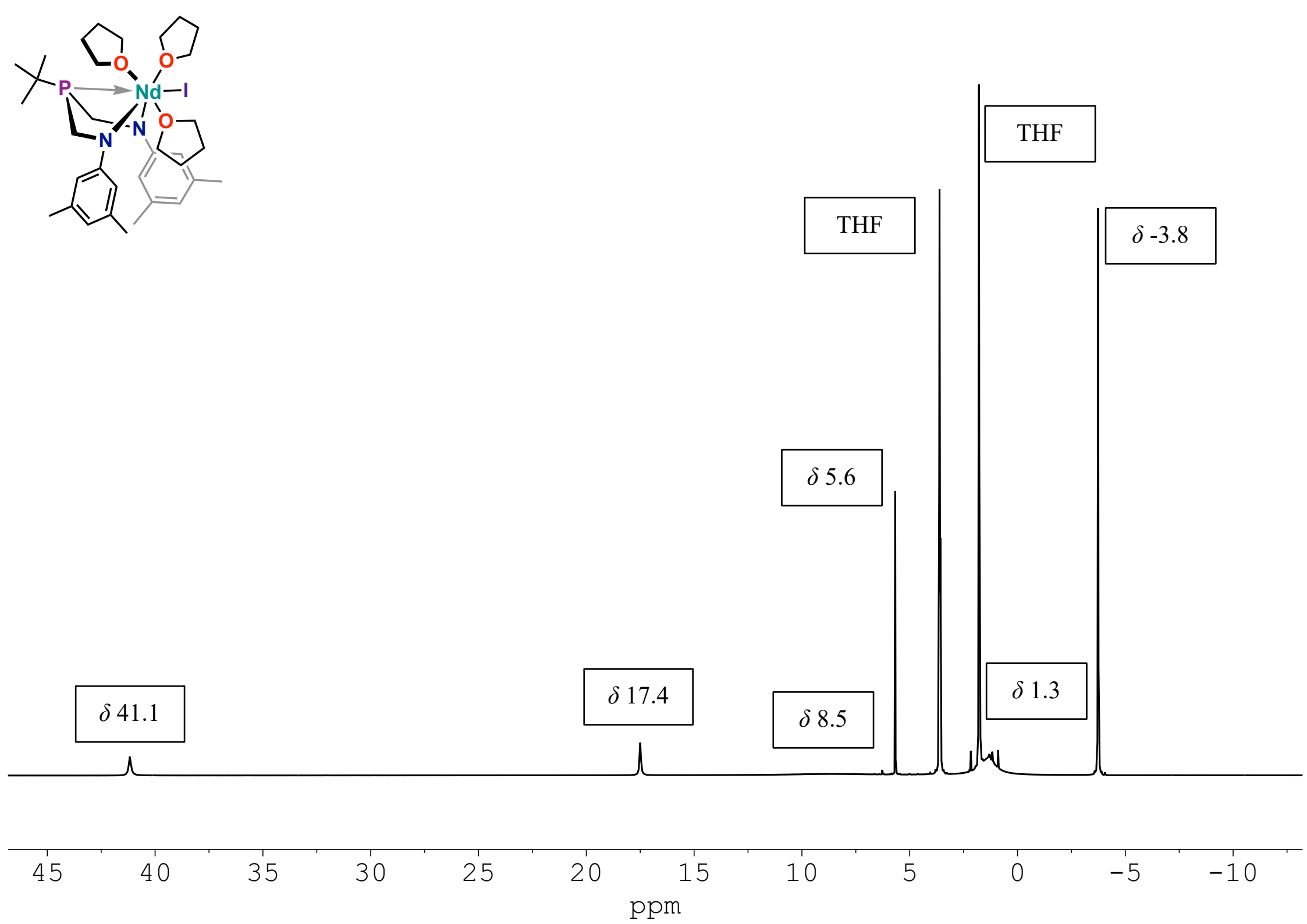

Figure S34. ^{1}H NMR spectrum of **NdI(^{Ar}BiAMP^{tBu})(thf)$_3$** acquired in THF-d_8 at room temperature.

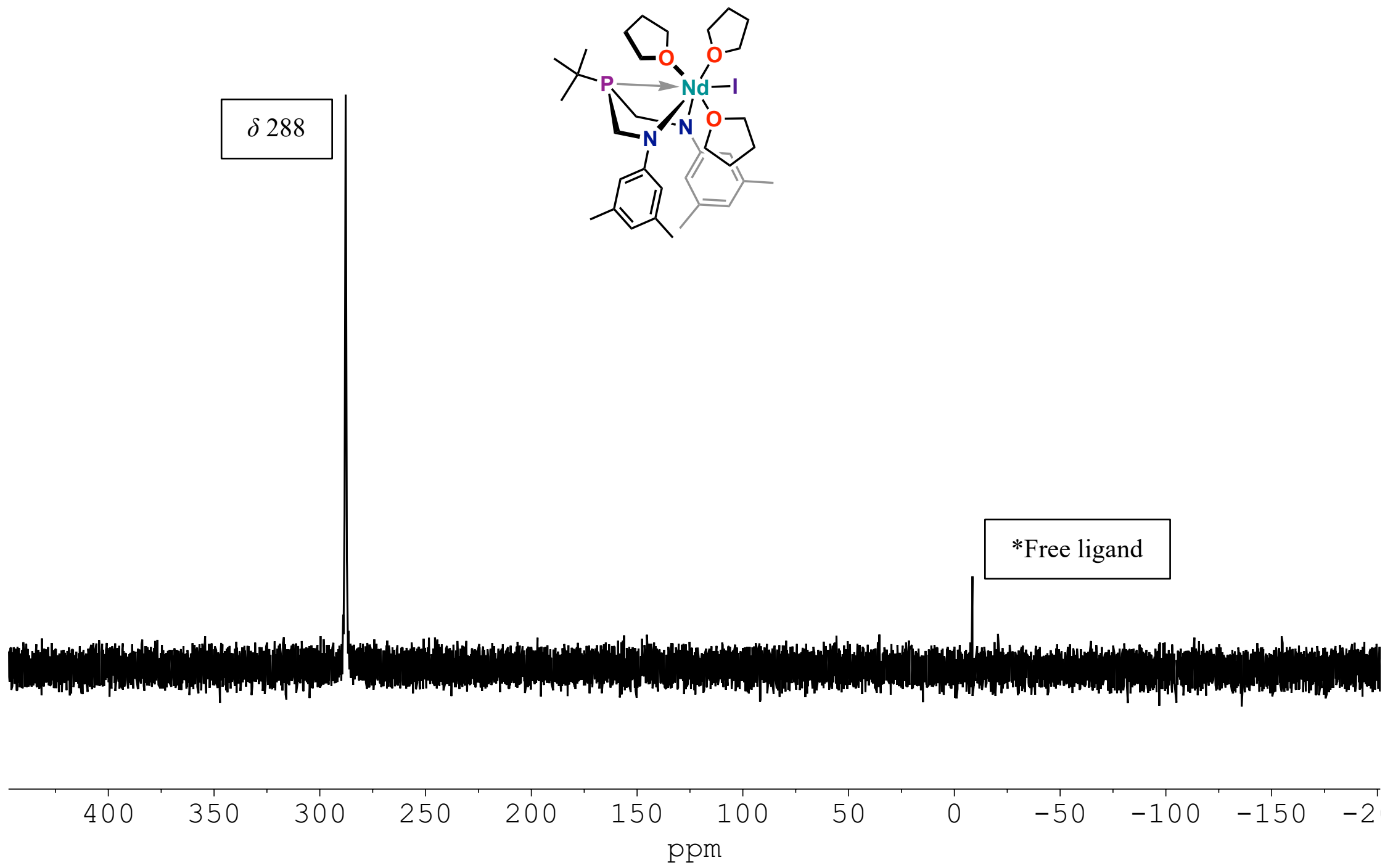

Figure S35. ^{31}P{^{1}H} NMR spectrum of **NdI(^{Ar}BiAMP^{tBu})(thf)$_3$** acquired in THF-d_8 at room temperature.

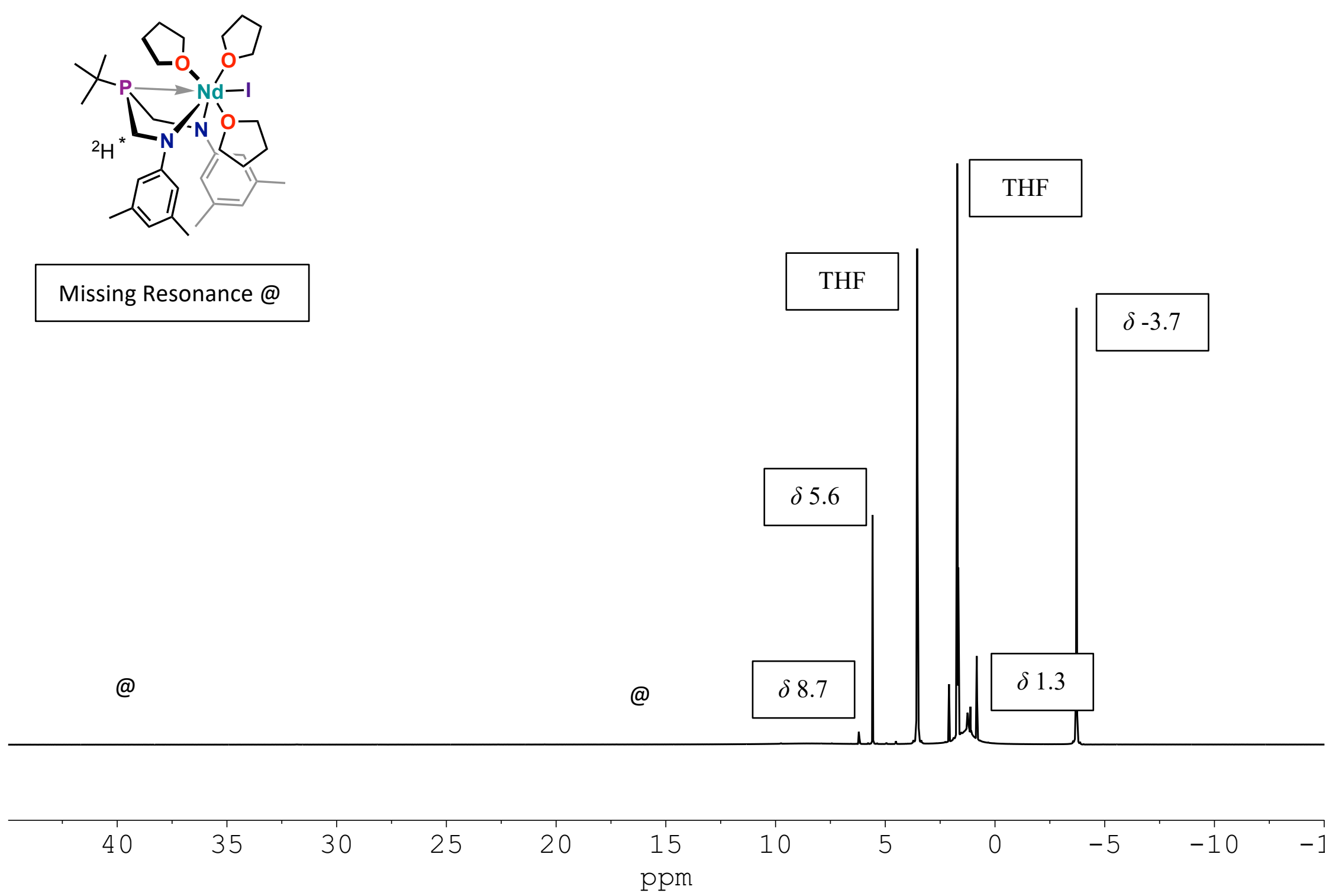

Figure S36. ^{1}H NMR spectrum of **NdI(d_4-^{Ar}BiAMP^{tBu})(thf)$_3$** acquired in THF-d_8 at room temperature.

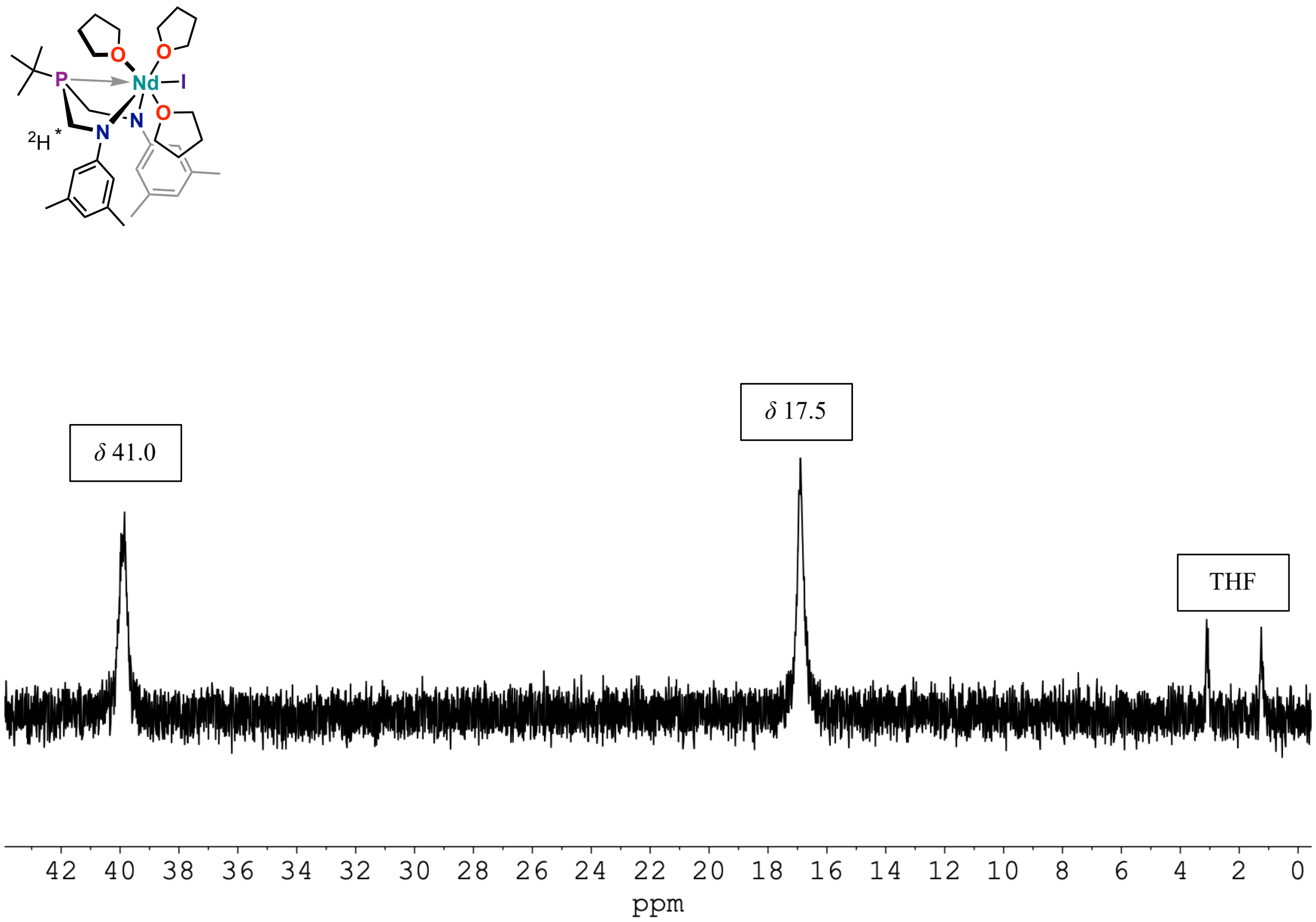

Figure S37. ^{2}H NMR spectrum of **NdI(d_4-^{Ar}BiAMP^{tBu})(thf)$_3$** acquired in THF at room temperature.

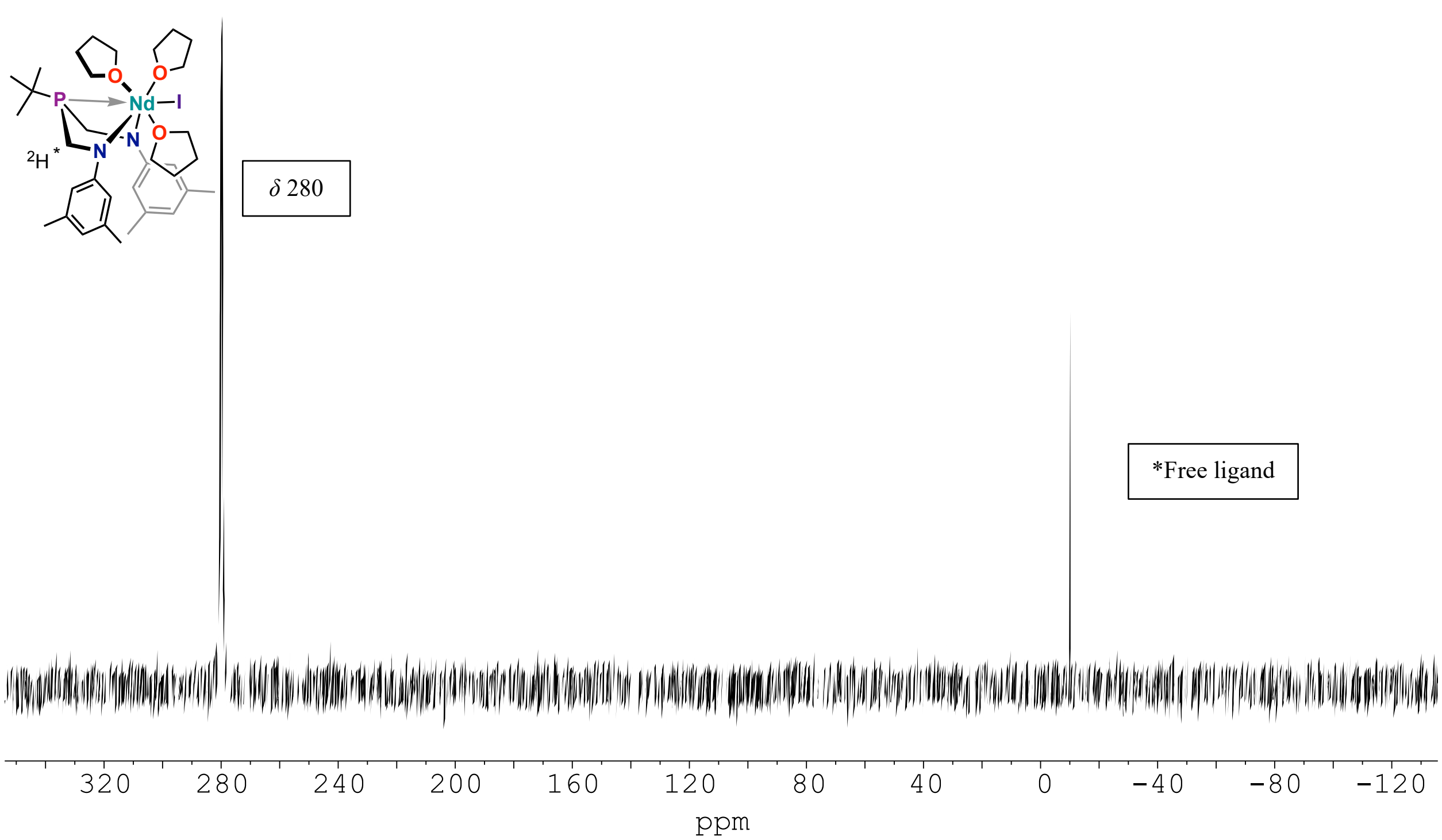

Figure S38. $^{31}P\{^{1}H\}$ NMR spectrum of **NdI(d_4-^{Ar}BiAMP^{tBu})(thf)$_3$** acquired in THF-d_8 at room temperature.

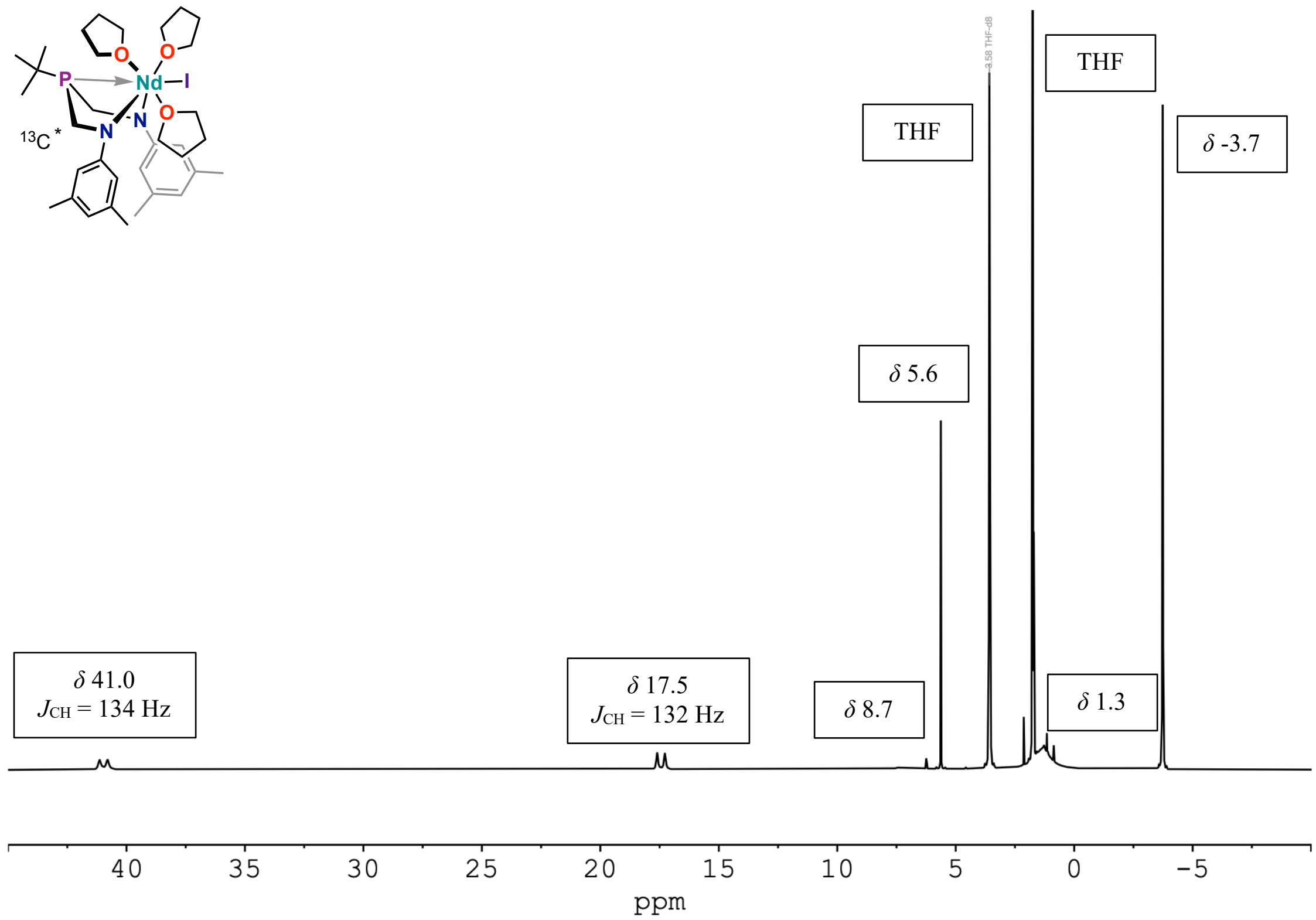

Figure S39. ^{1}H NMR spectrum of **NdI(^{13}C$_2$-^{Ar}BiAMP^{tBu})(thf)$_3$** acquired in THF-d_8 at room temperature.

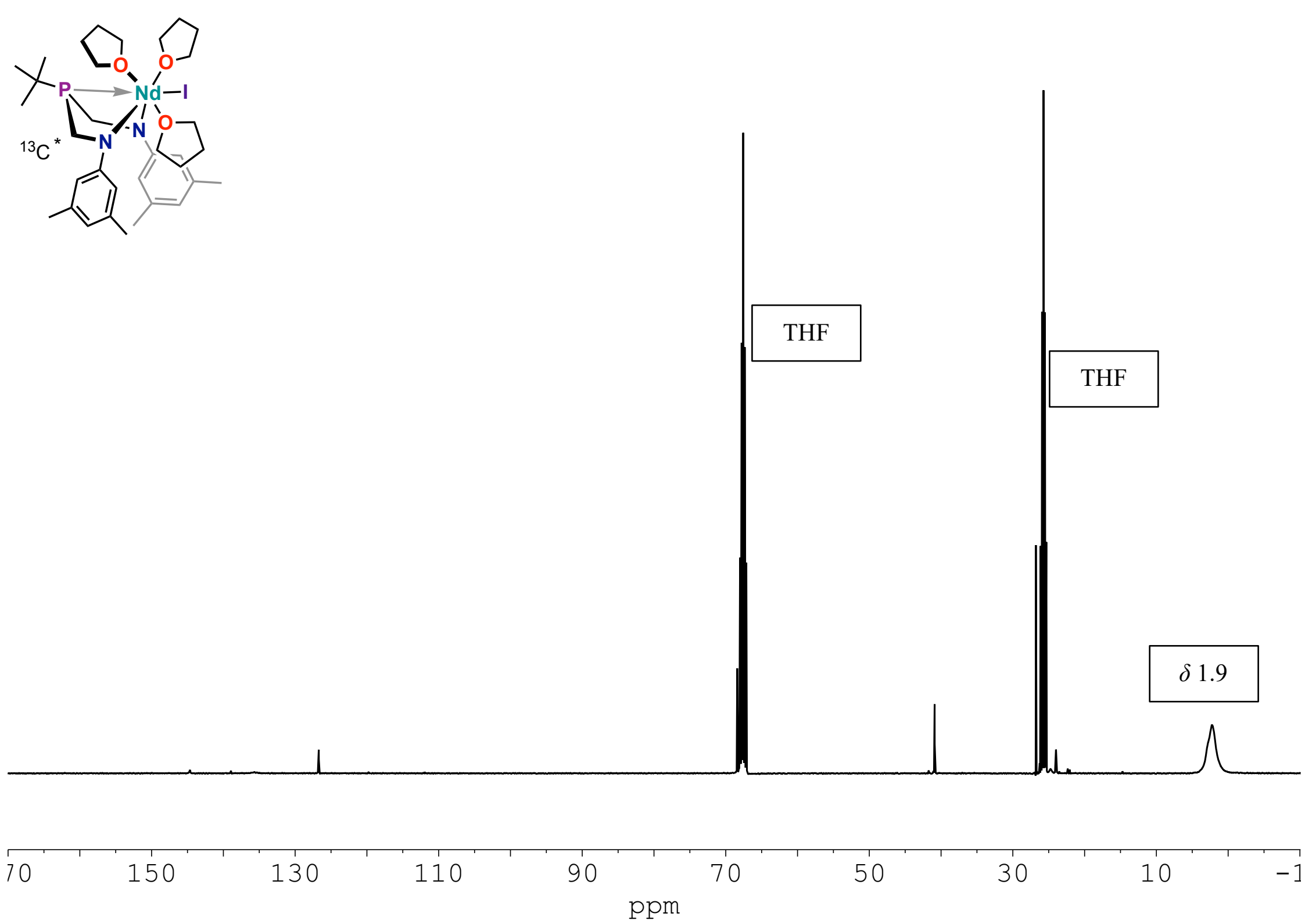

Figure S40. ^{13}C{^{1}H} NMR spectrum of **NdI(^{13}C$_2$-^{Ar}BiAMP^{tBu})(thf)$_3$** acquired in THF-d_8 at room temperature.

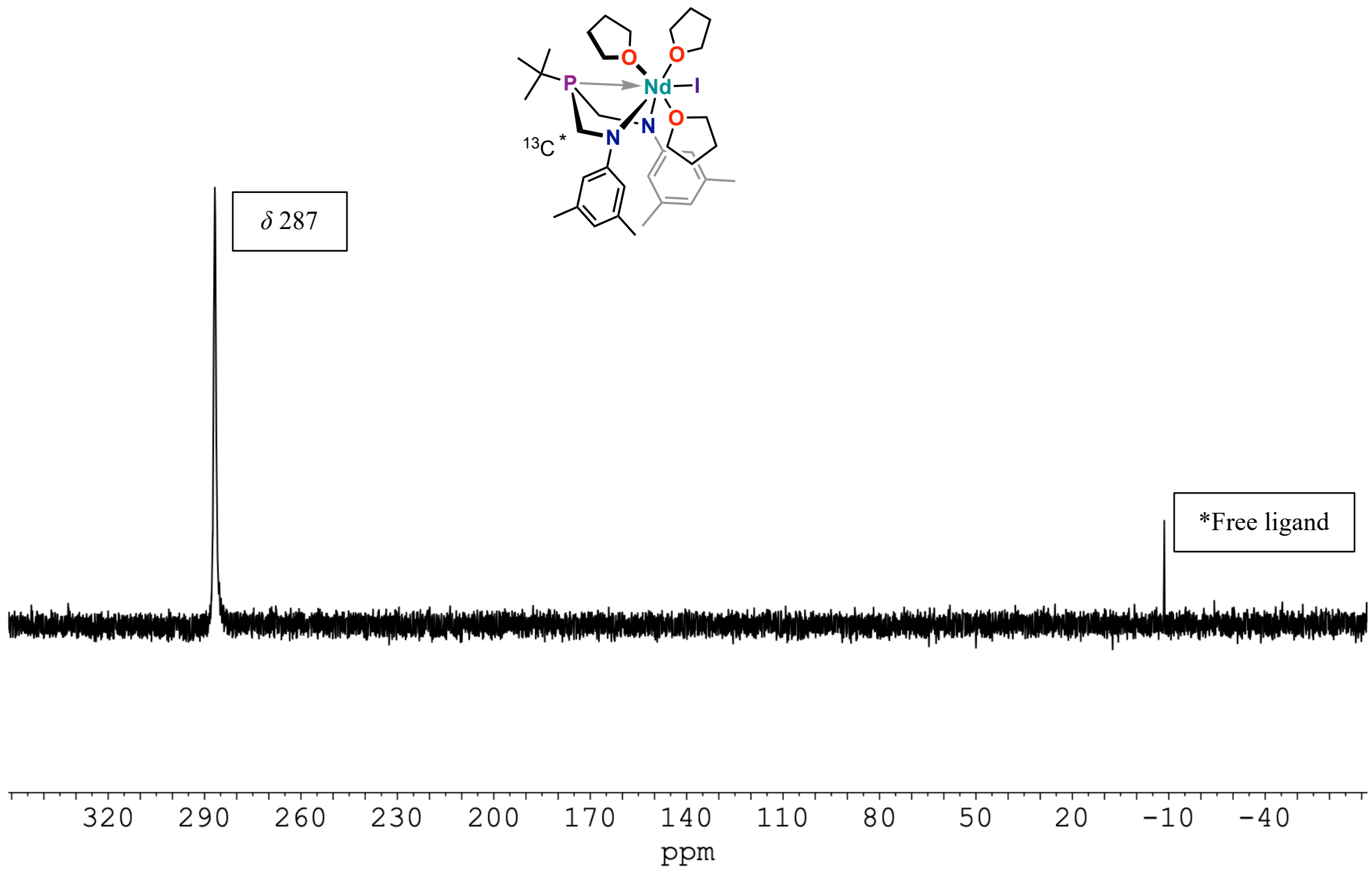

Figure S41. $^{31}P\{^{1}H\}$ NMR spectrum of **NdI($^{13}C_2$-^{Ar}BiAMP^{tBu})(thf)$_3$** acquired in THF-d_8 at room temperature.

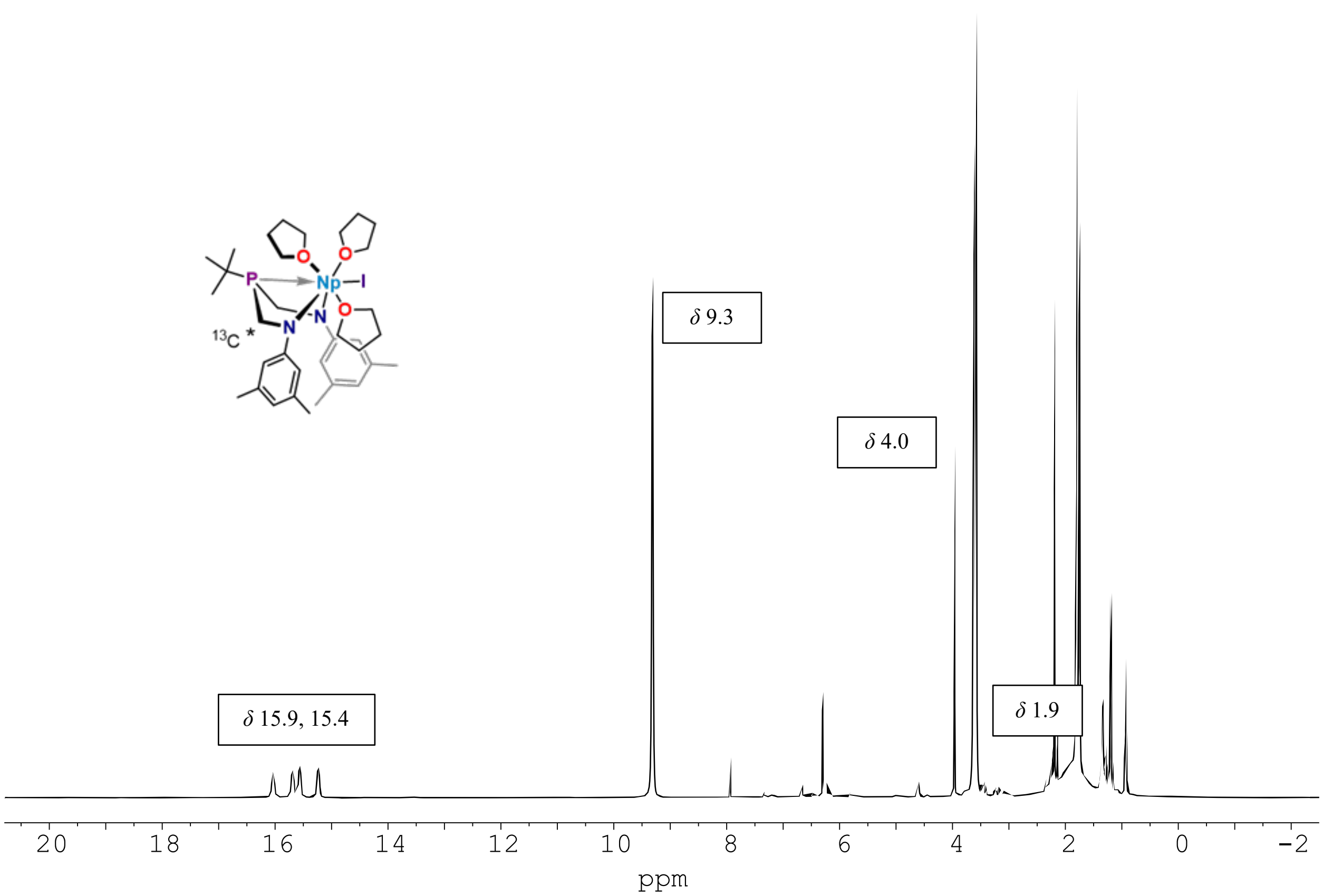

Figure S42. ^{1}H NMR spectrum of **NpI($^{13}C_2$-^{Ar}BiAMP^{tBu})(thf)$_3$** acquired in THF-d_8 at room temperature.

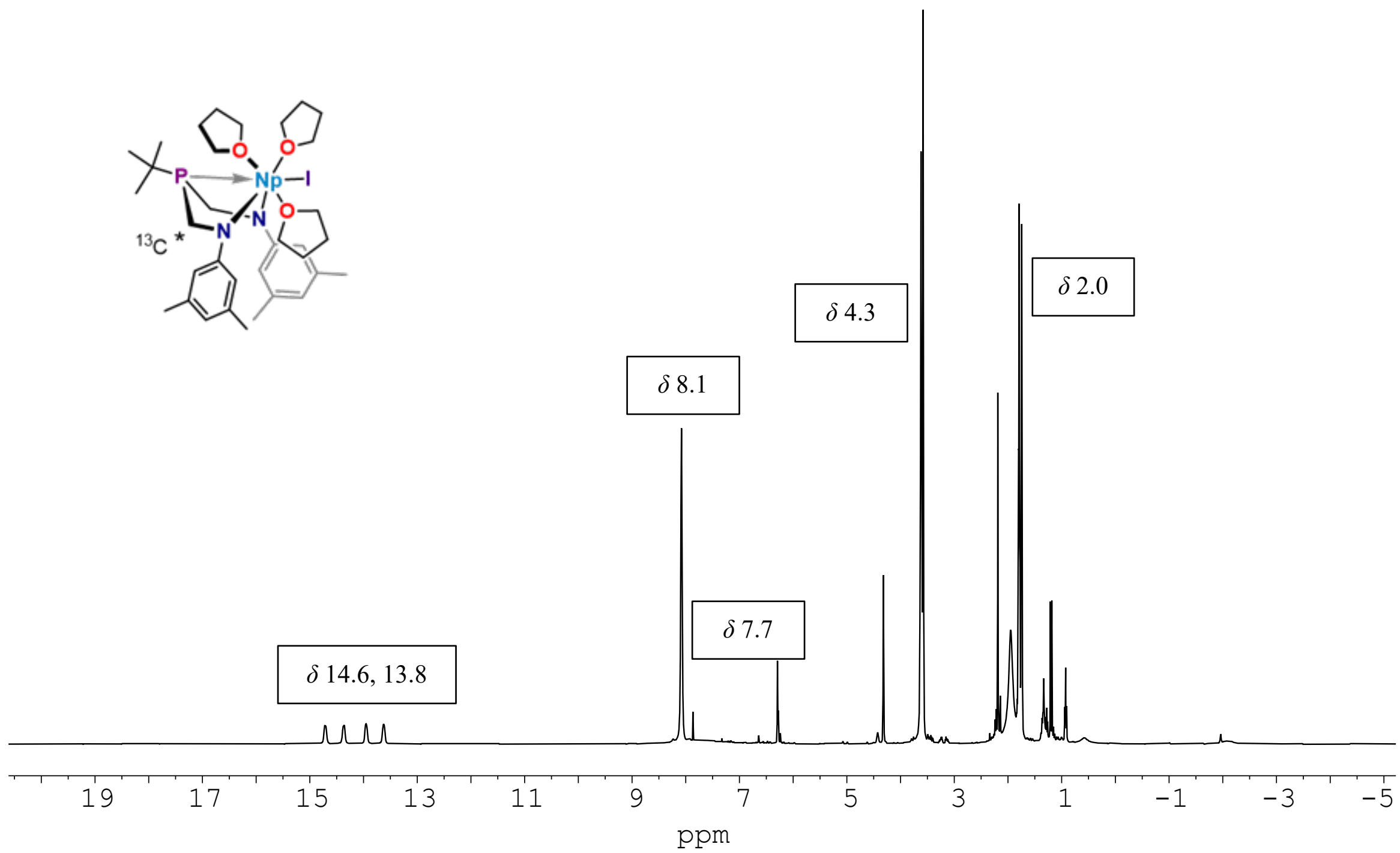

Figure S43. ^{1}H NMR spectrum of **NpI(^{13}C$_2$-^{Ar}BiAMP^{tBu})(thf)$_3$** acquired in THF-d_8 at 50 °C.

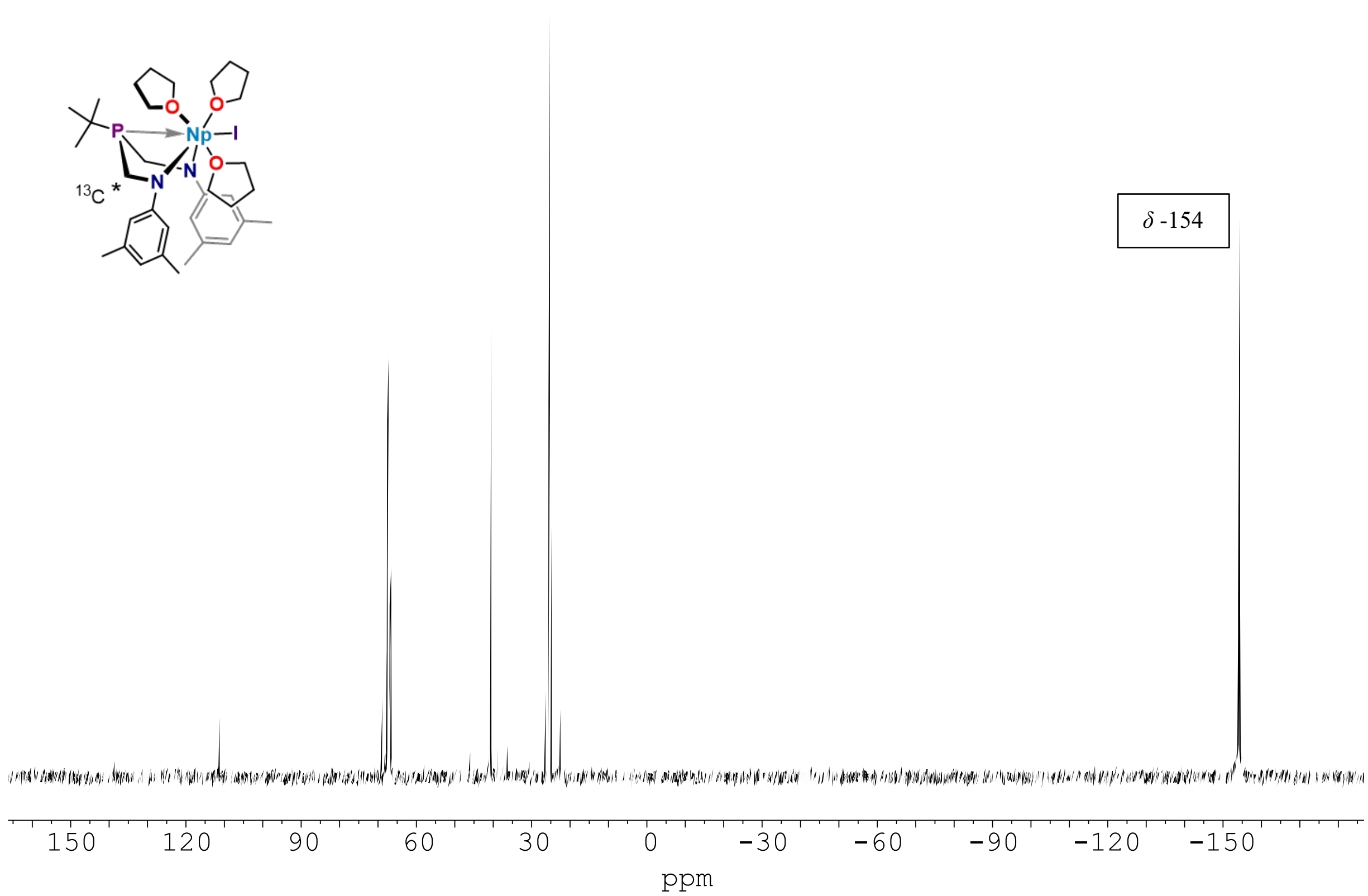

Figure S44. ^{13}C{^{1}H} NMR spectrum of **NpI(^{13}C$_2$-^{Ar}BiAMP^{tBu})(thf)$_3$** acquired in THF-d_8 at room temperature.

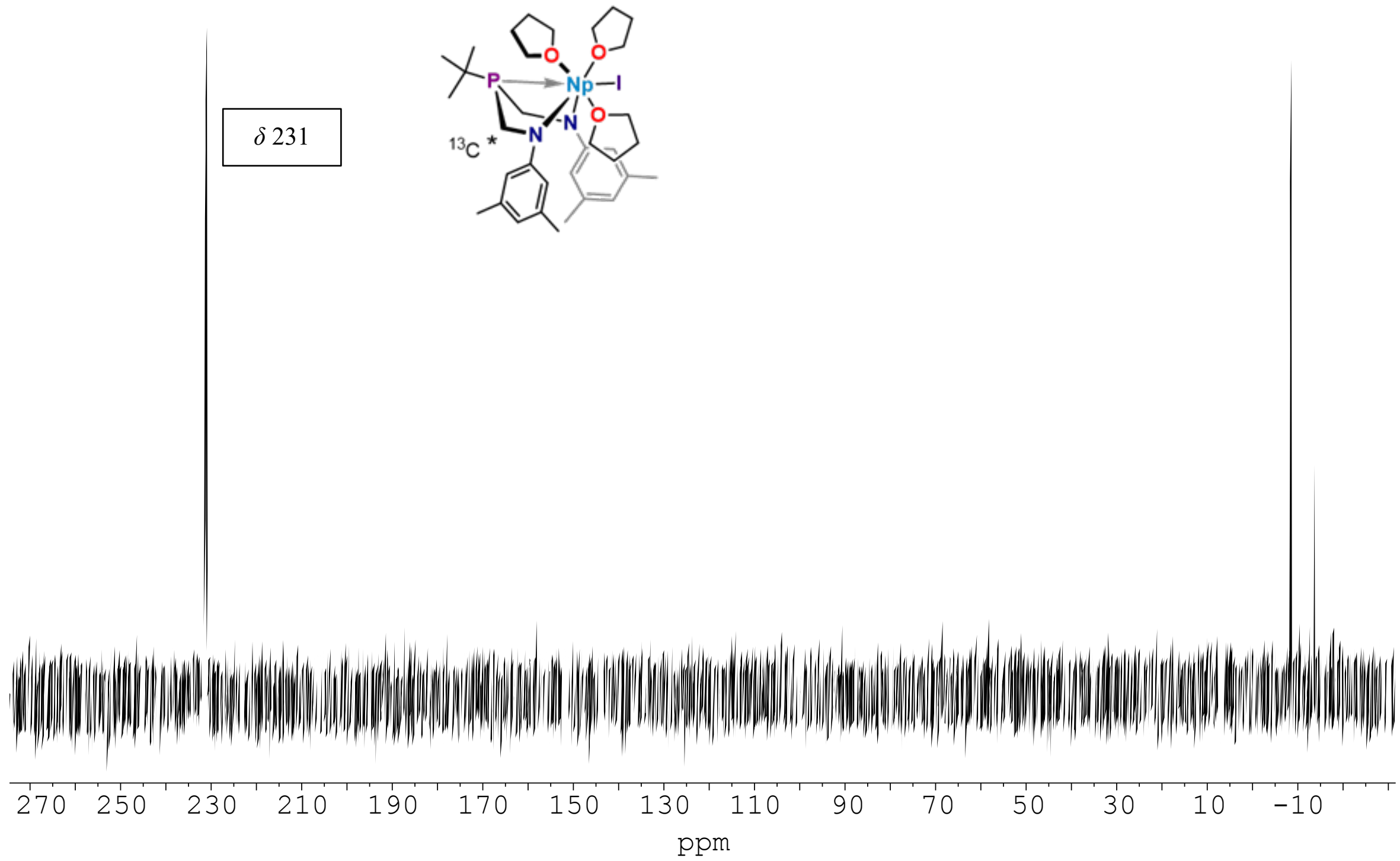

Figure S45. $^{31}P\{^{1}H\}$ NMR spectrum of **NpI($^{13}C_2$-^{Ar}BiAMP^{tBu})(thf)$_3$** acquired in THF-d_8 at room temperature.

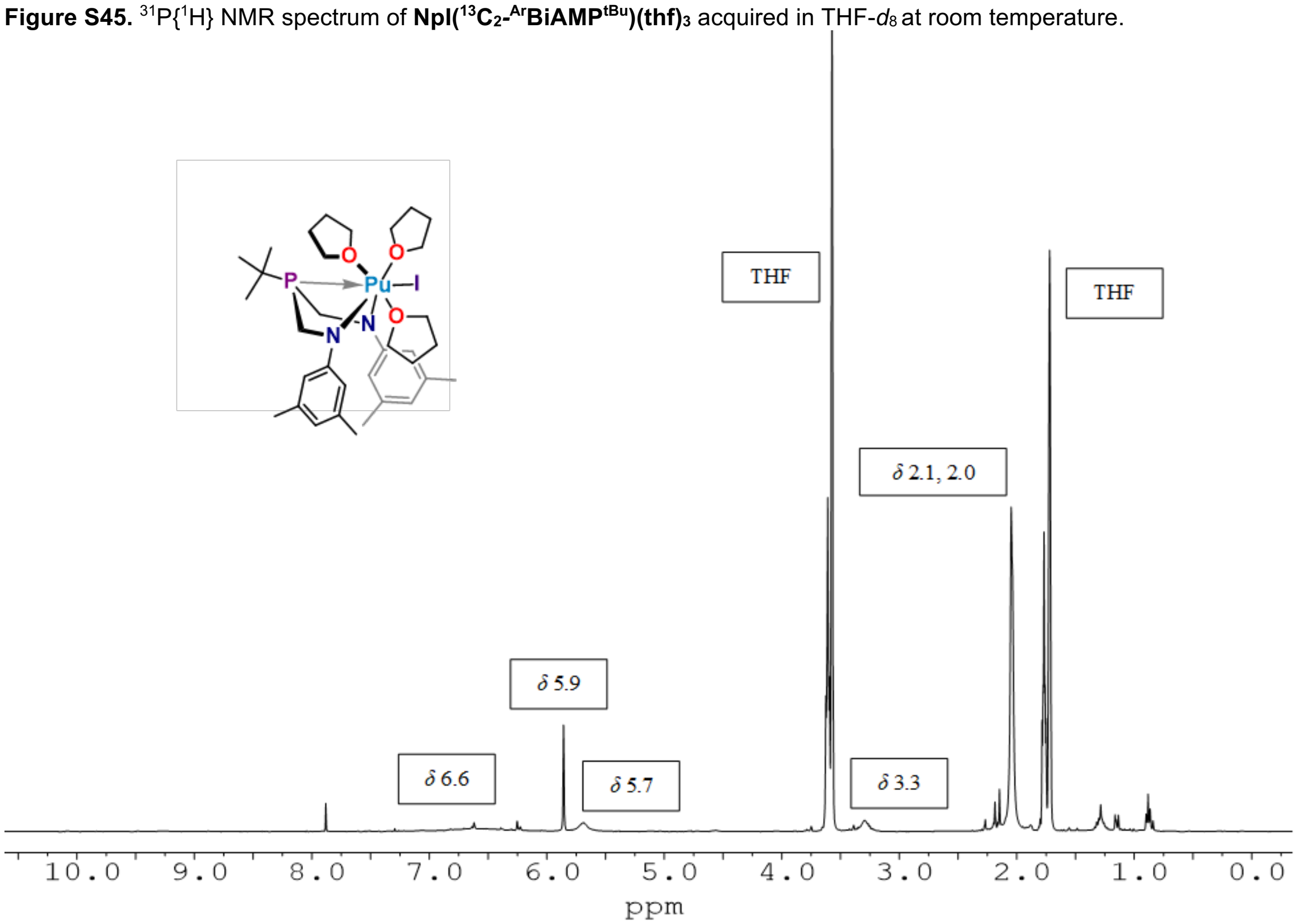

Figure S46. ^{1}H NMR spectrum **PuI($^{13}C_2$-^{Ar}BiAMP^{tBu})(thf)$_3$** acquired in THF-d_8 at room temperature.

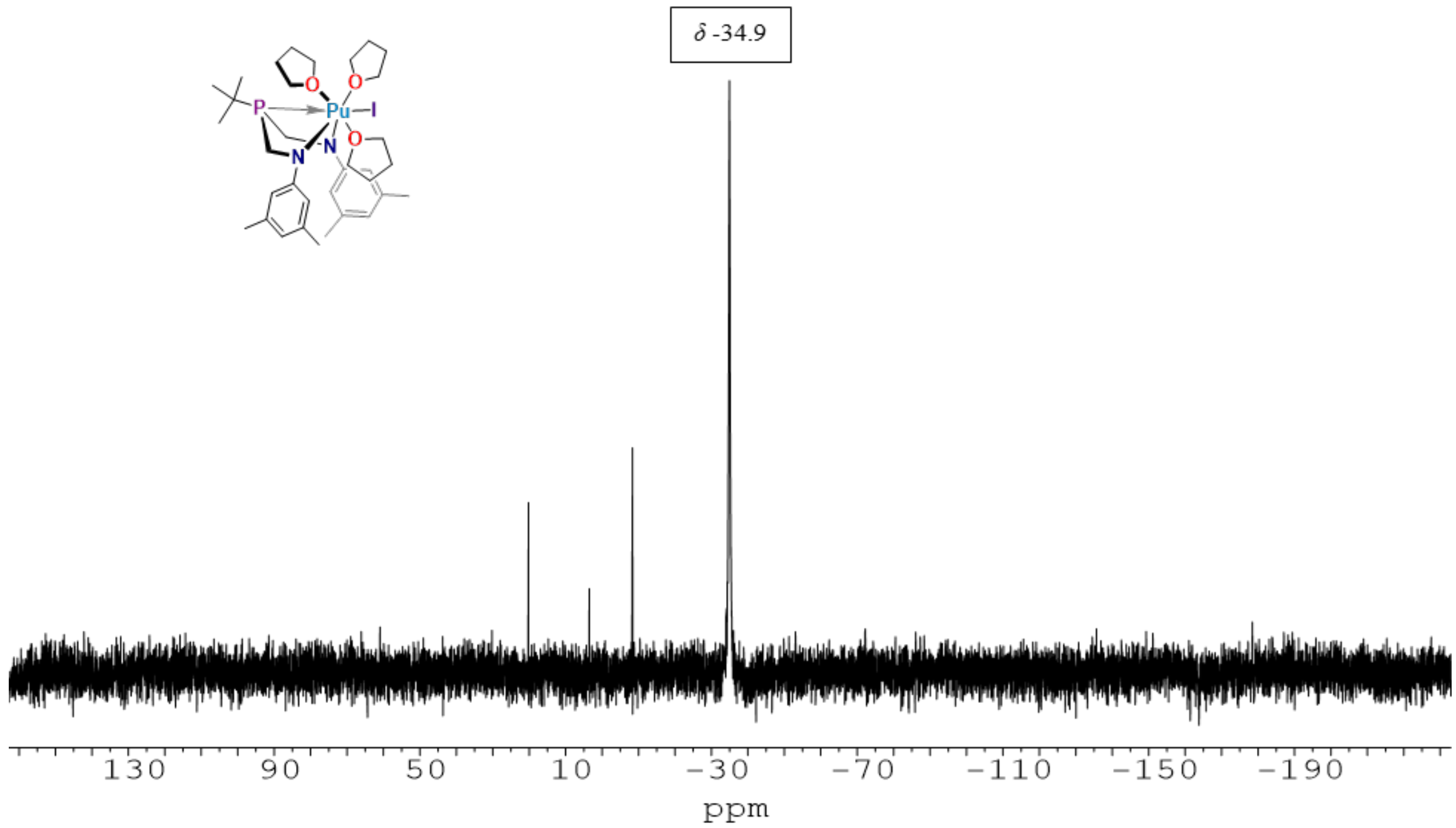

Figure S47. $^{31}P\{^{1}H\}$ NMR spectrum of **PuI($^{13}C_2$-^{Ar}BiAMP^{tBu})(thf)$_3$** acquired in THF-d_8 at room temperature.

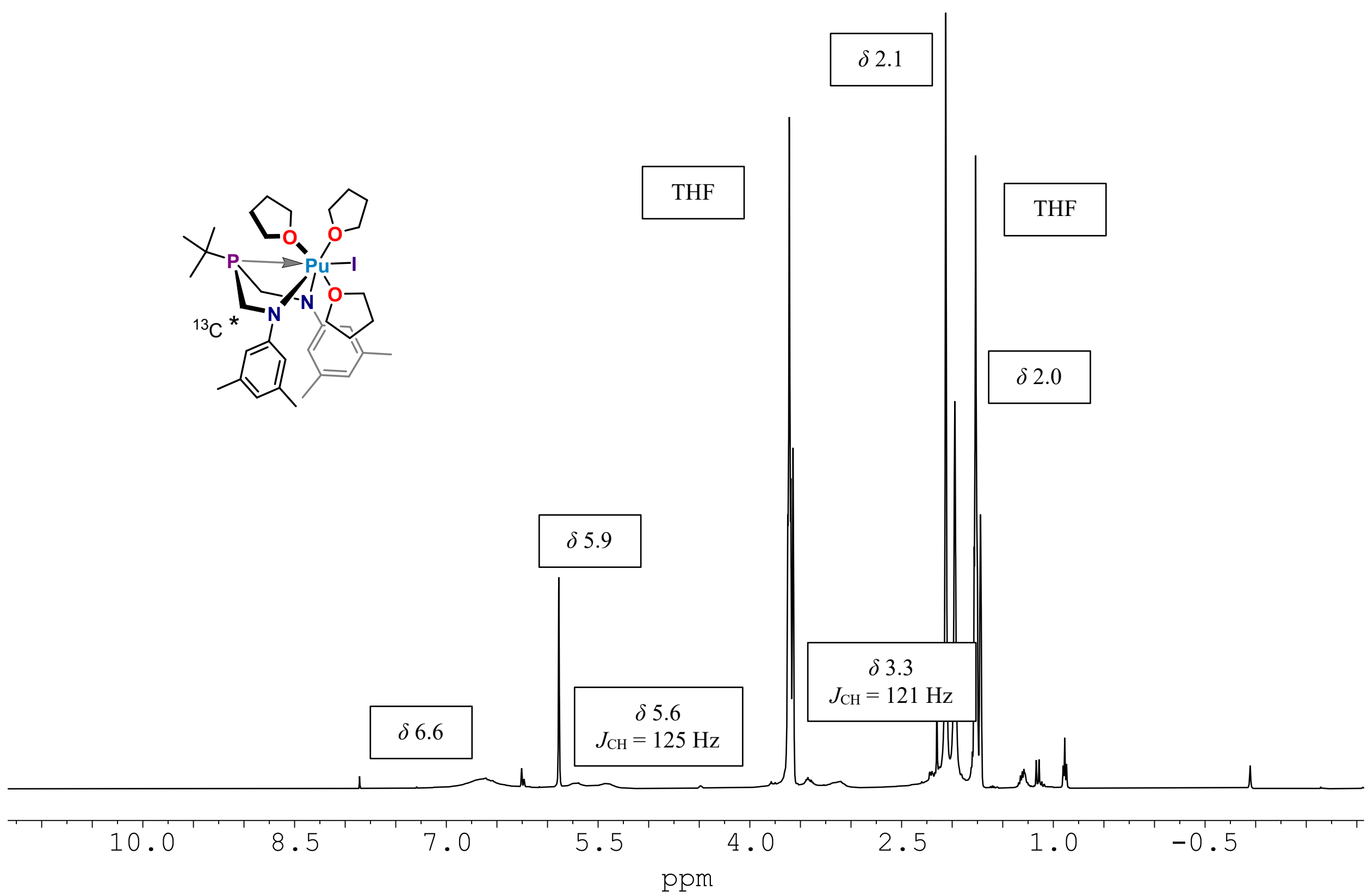

Figure S48. ^{1}H NMR spectrum of **PuI($^{13}C_2$-^{Ar}BiAMP^{tBu})(thf)$_3$** acquired in THF-d_8 at 35 °C.

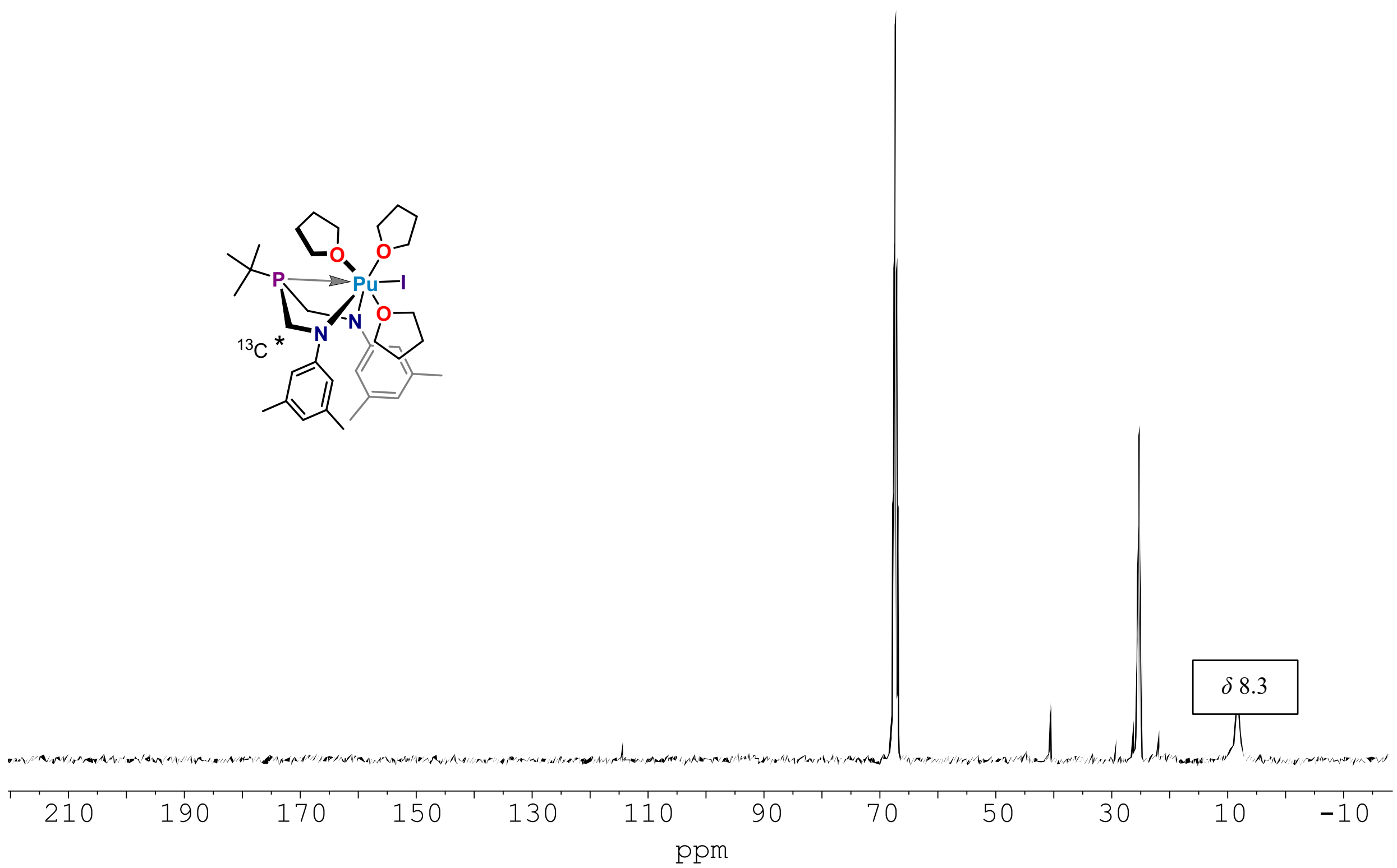

Figure S49. $^{13}C\{^1H\}$ NMR spectrum of **PuI($^{13}C_2$-^{Ar}BiAMP^{tBu})(thf)$_3$** acquired in THF-d_8 at 25 °C.

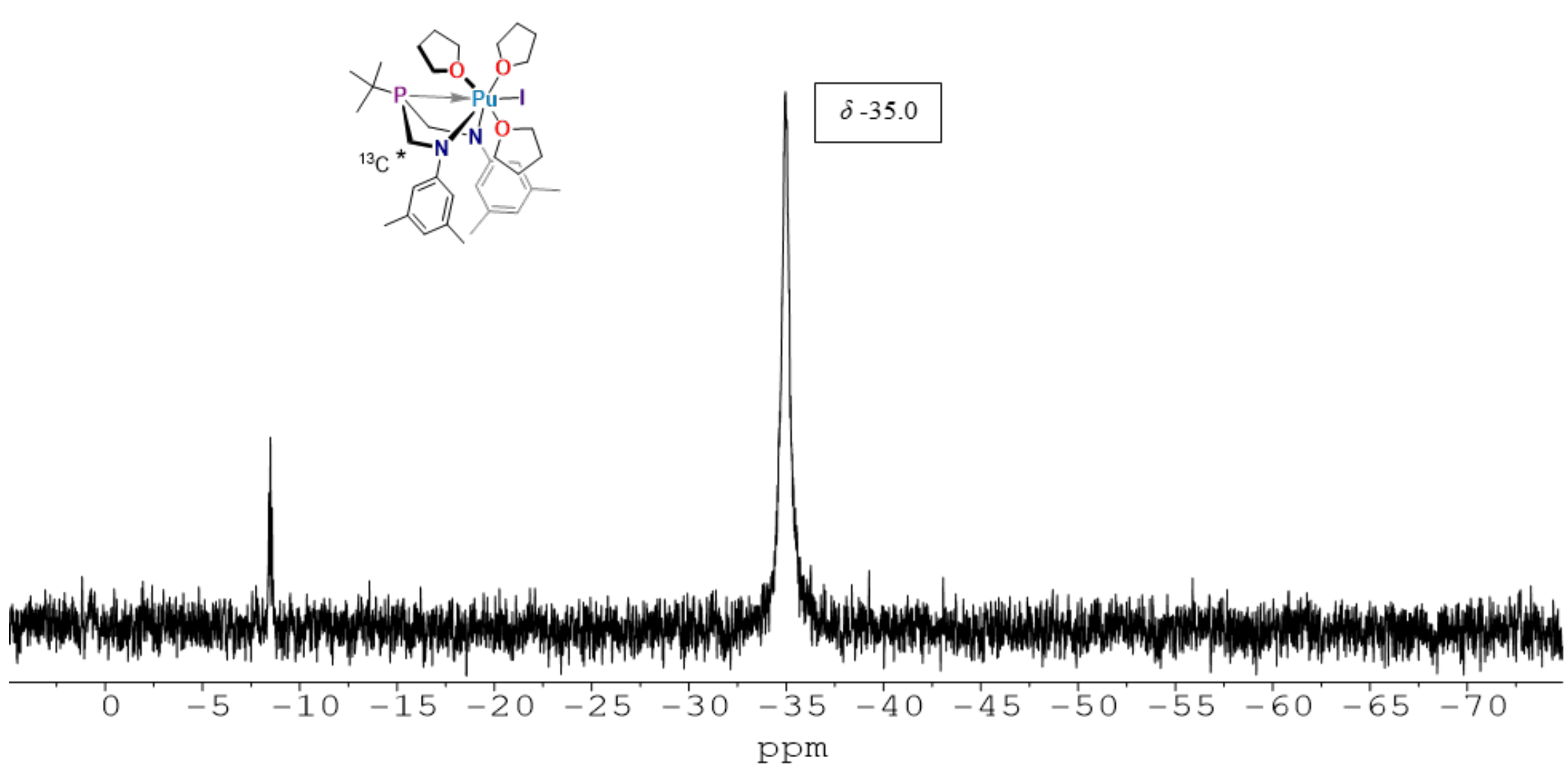

Figure S50. $^{31}P\{^1H\}$ NMR spectrum of **PuI($^{13}C_2$-^{Ar}BiAMP^{tBu})(thf)$_3$** acquired in THF-d_8 at 25 °C.

NMR Overlays – Labelled and Natural Abundance

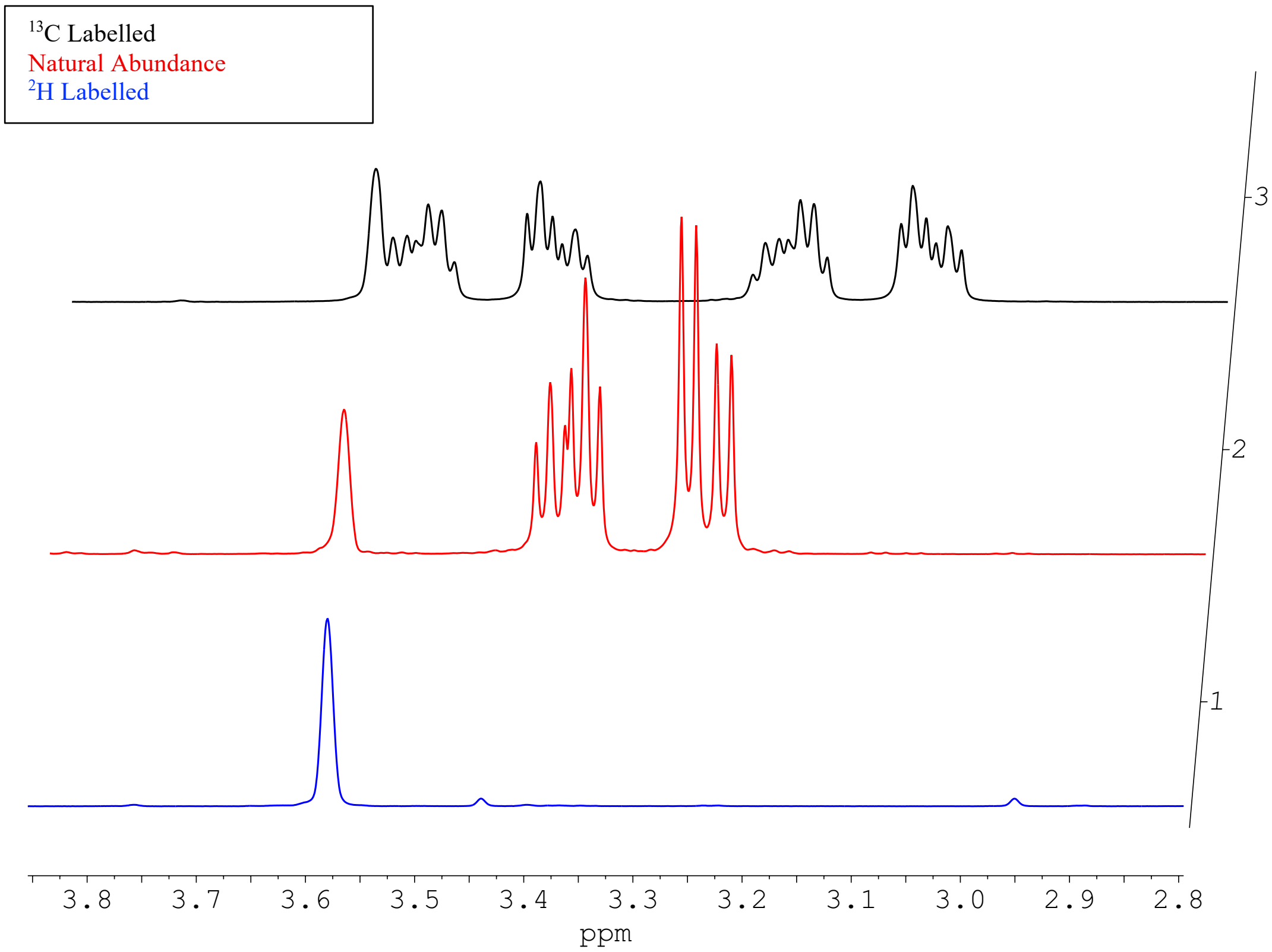

Figure S51. Overlay of expansions of the methylene region for: **^{13}C$_2$-^{Ar}BiAMP^{tBu}**, **^{Ar}BiAMP^{tBu}**, and **d$_4$-^{Ar}BiAMP^{tBu}** in the ^{1}H NMR spectrum.

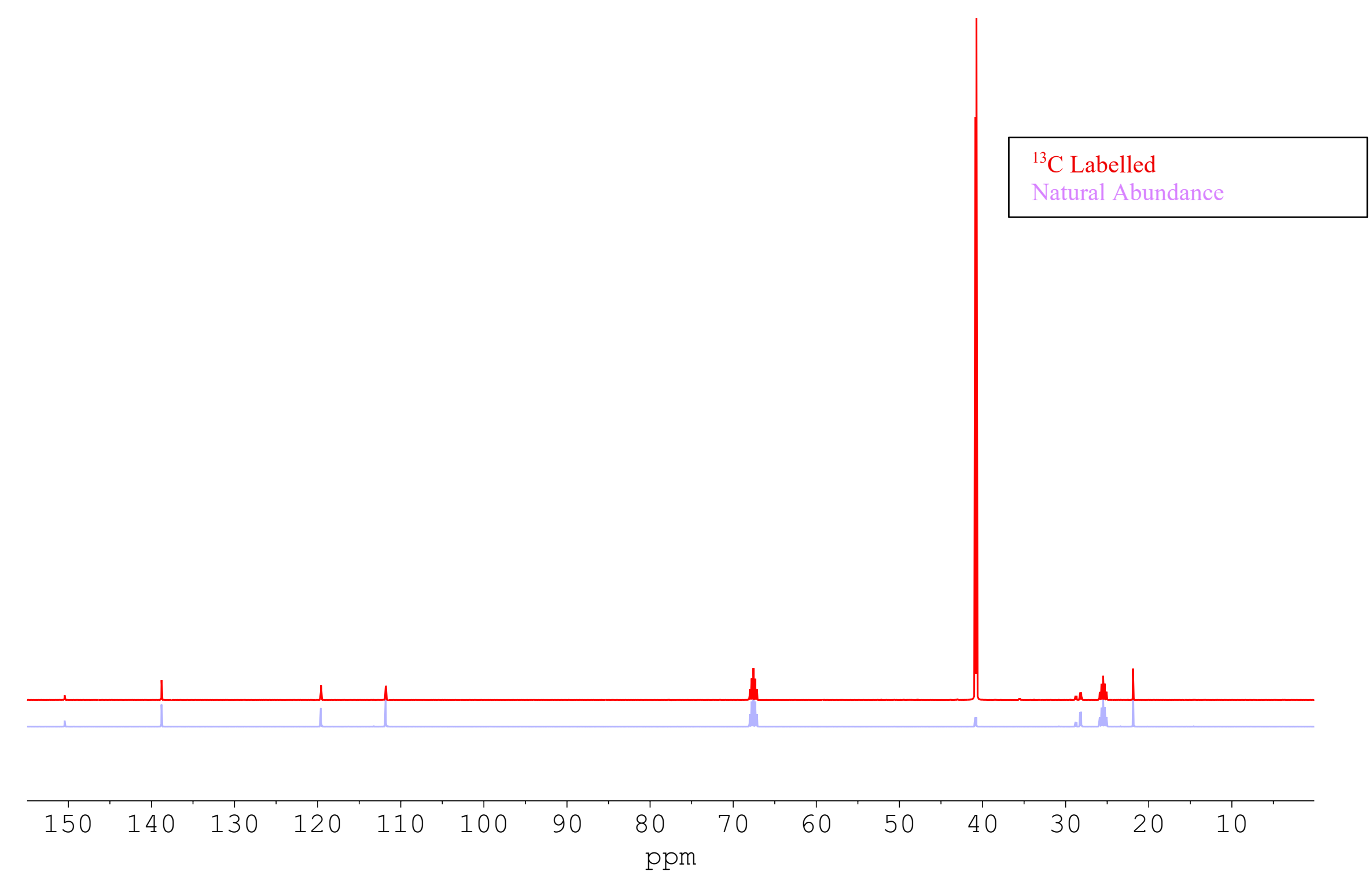

Figure S52. Overlay of the ^{13}C{^{1}H} NMR spectra of **^{13}C$_2$-^{Ar}BiAMP^{tBu}** and **^{Ar}BiAMP^{tBu}**.

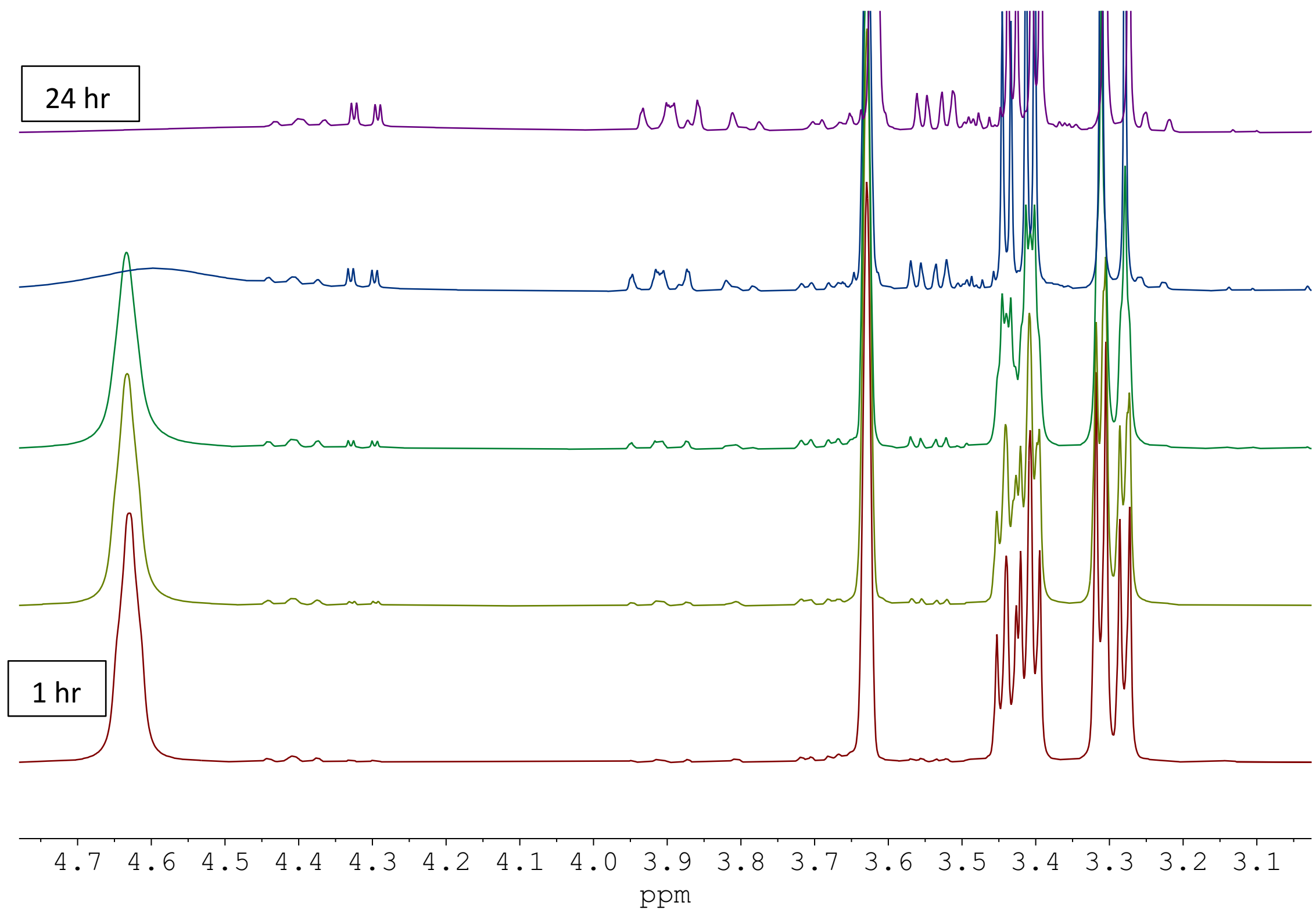

Figure S53. Expansion of the methylene resonances of the ^{1}H NMR spectra of **^{Ar}BiAMP^{tBu}** acquired in THF-d_8 at room temperature at various time points over the course of 24 hours in solution.

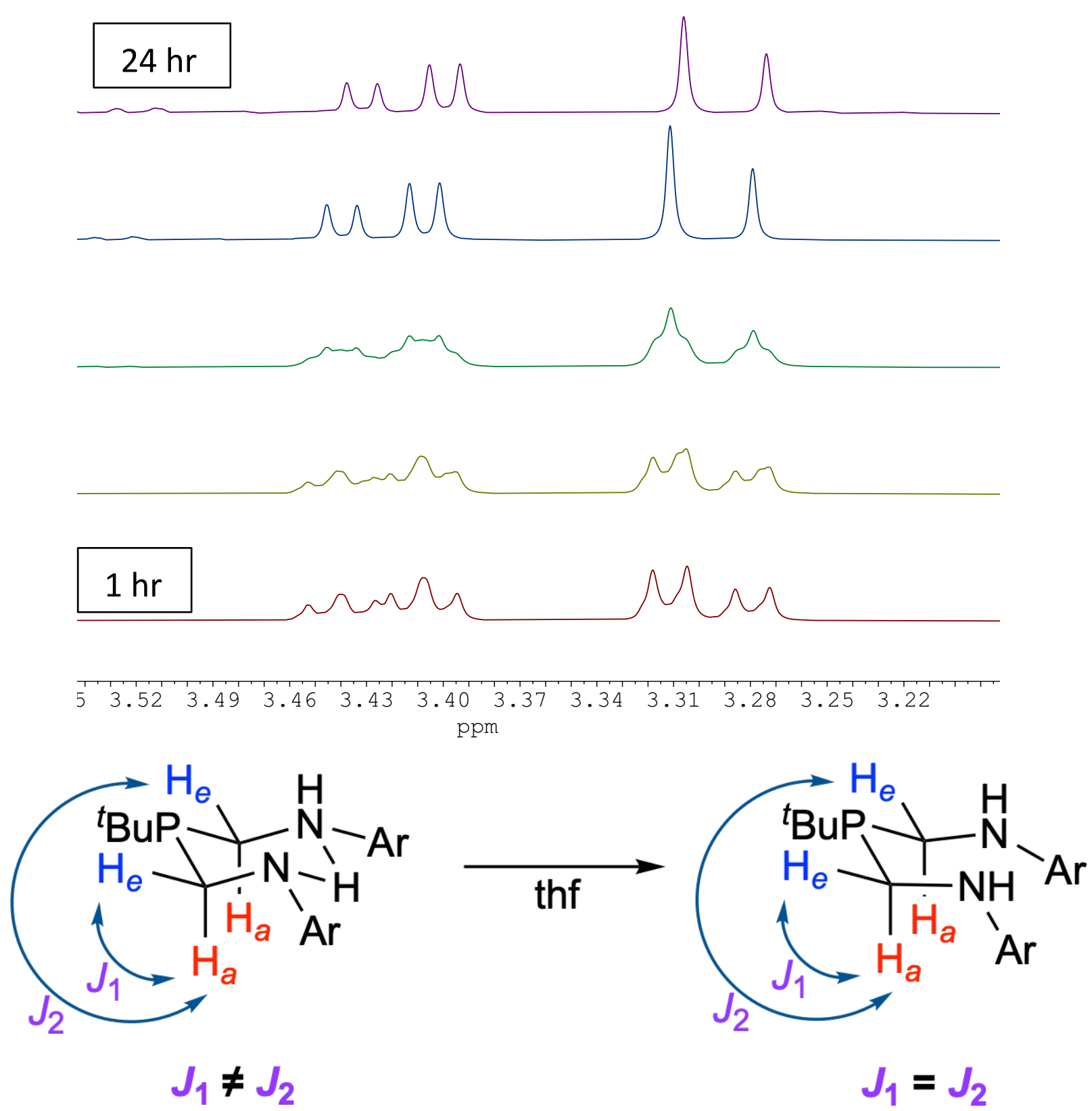

Figure S54. Expansion of the methylene resonances of the ^{1}H NMR spectra of **^{Ar}BiAMP^{tBu}** acquired in THF-d_8 at room temperature at various time points over the course of 24 hours in solution (**top**); coupling patterns arising from the changes in the proposed break-up of the hydrogen bonding in **^{Ar}BiAMP^{tBu}** in THF (**bottom**). Hydrogen bond donor/acceptor pair exchange is likely present.

2.2 UV-vis-NIR Spectra

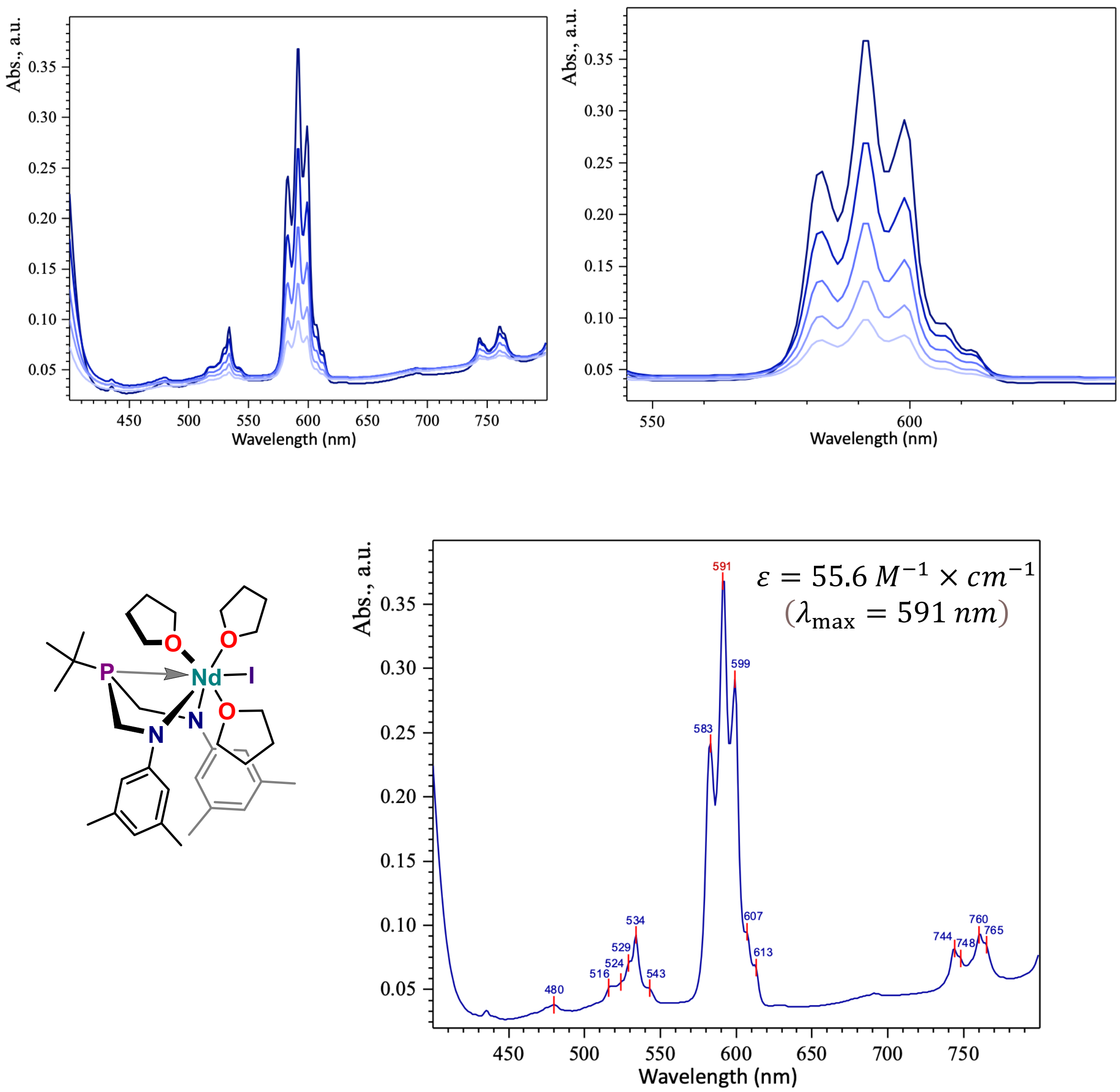

Figure S55. Solution-state UV-vis-NIR spectra of **NdI(^{Ar}BiAMP^{tBu})(thf)$_3$** (**2**) recorded in THF (1-5 mM, **top**) at room temperature, and the maximum extinction coefficient (**bottom**).

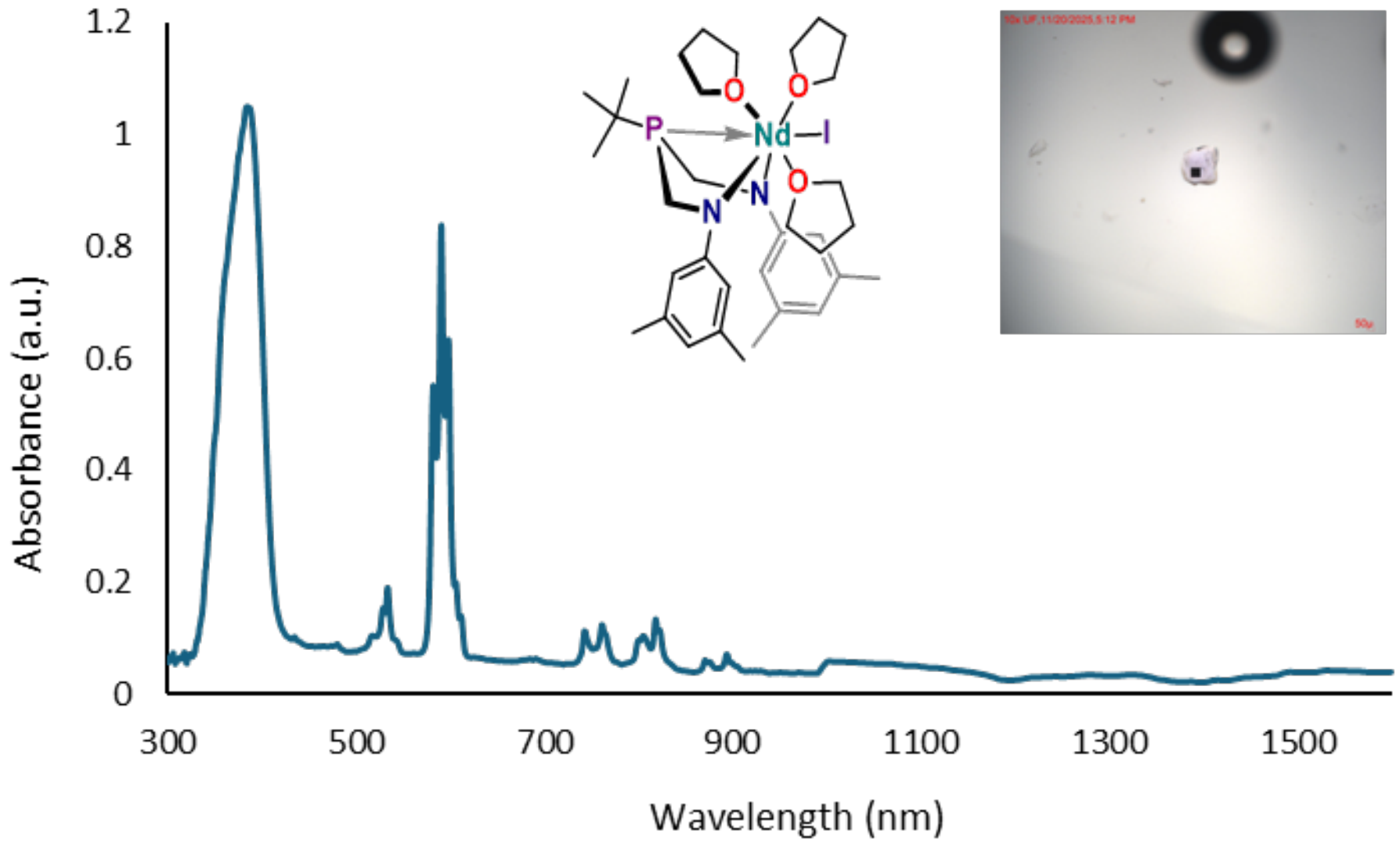

Figure S56. Solid-state UV-vis-NIR spectrum of **NdI(^{Ar}BiAMP^{tBu})(thf)$_3$** (**2**) recorded at room temperature. The photo depicts the crystal used for the measurement.

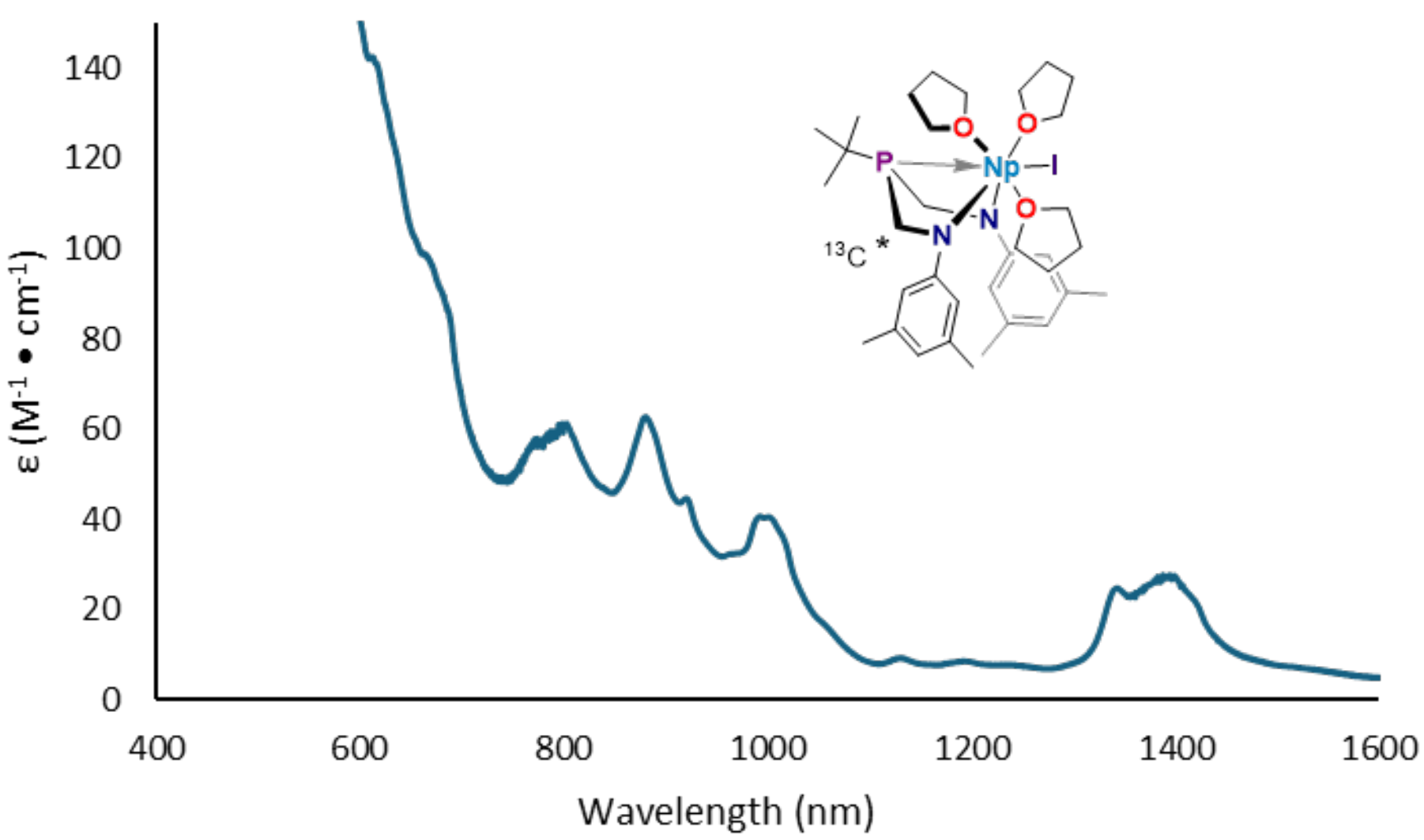

Figure S57. Solution-state vis-NIR spectrum of **NpI(^{13}C$_2$-^{Ar}BiAMP^{tBu})(thf)$_3$** (**^{13}C-3**) recorded in THF (2.6 mM) at room temperature.

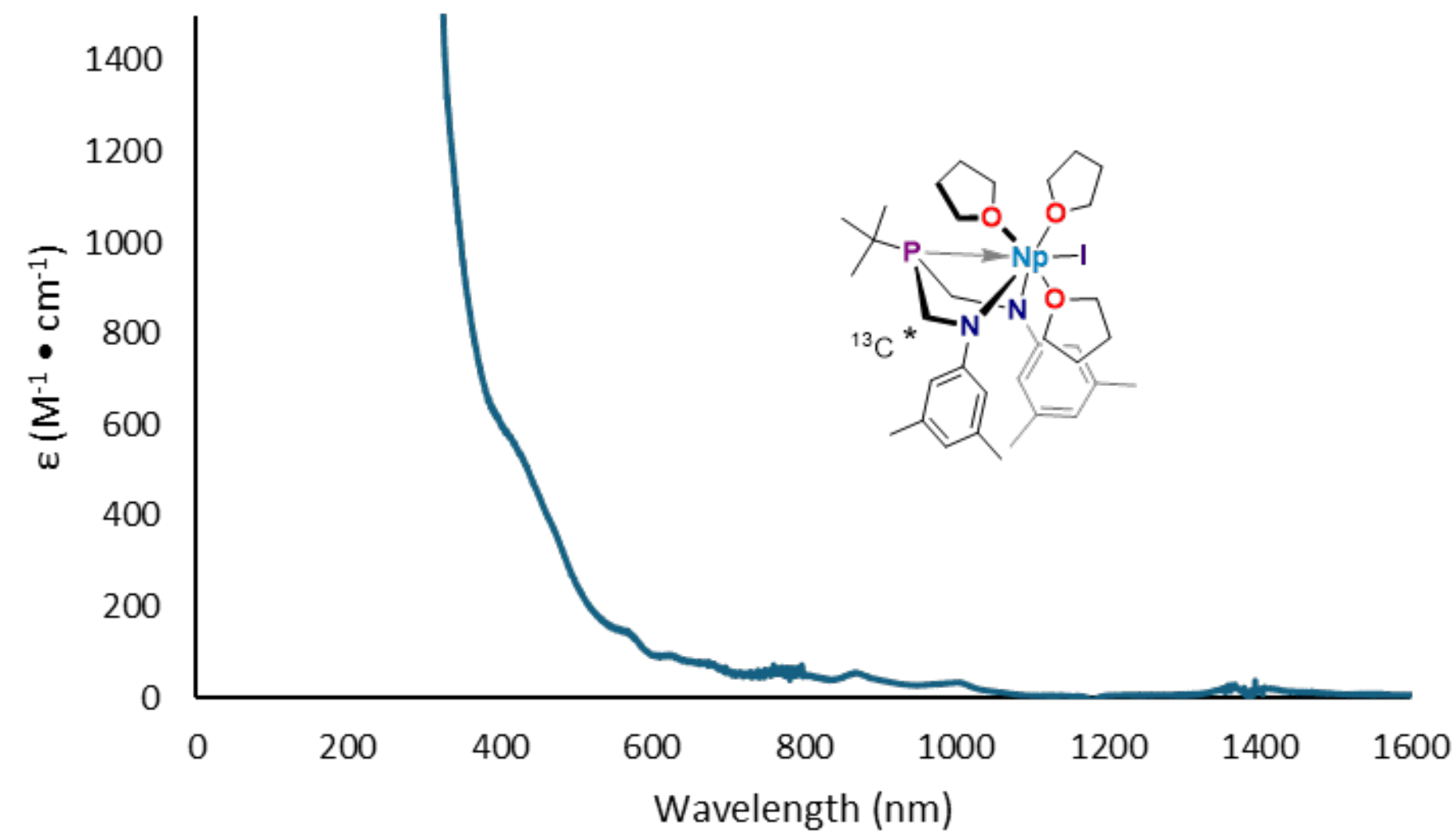

Figure S58. Solution-state UV-vis-NIR spectrum of **NpI(^{13}C$_2$-^{Ar}BiAMP^{tBu})(thf)$_3$** (**^{13}C-3**) recorded in THF (0.25 mM) at room temperature.

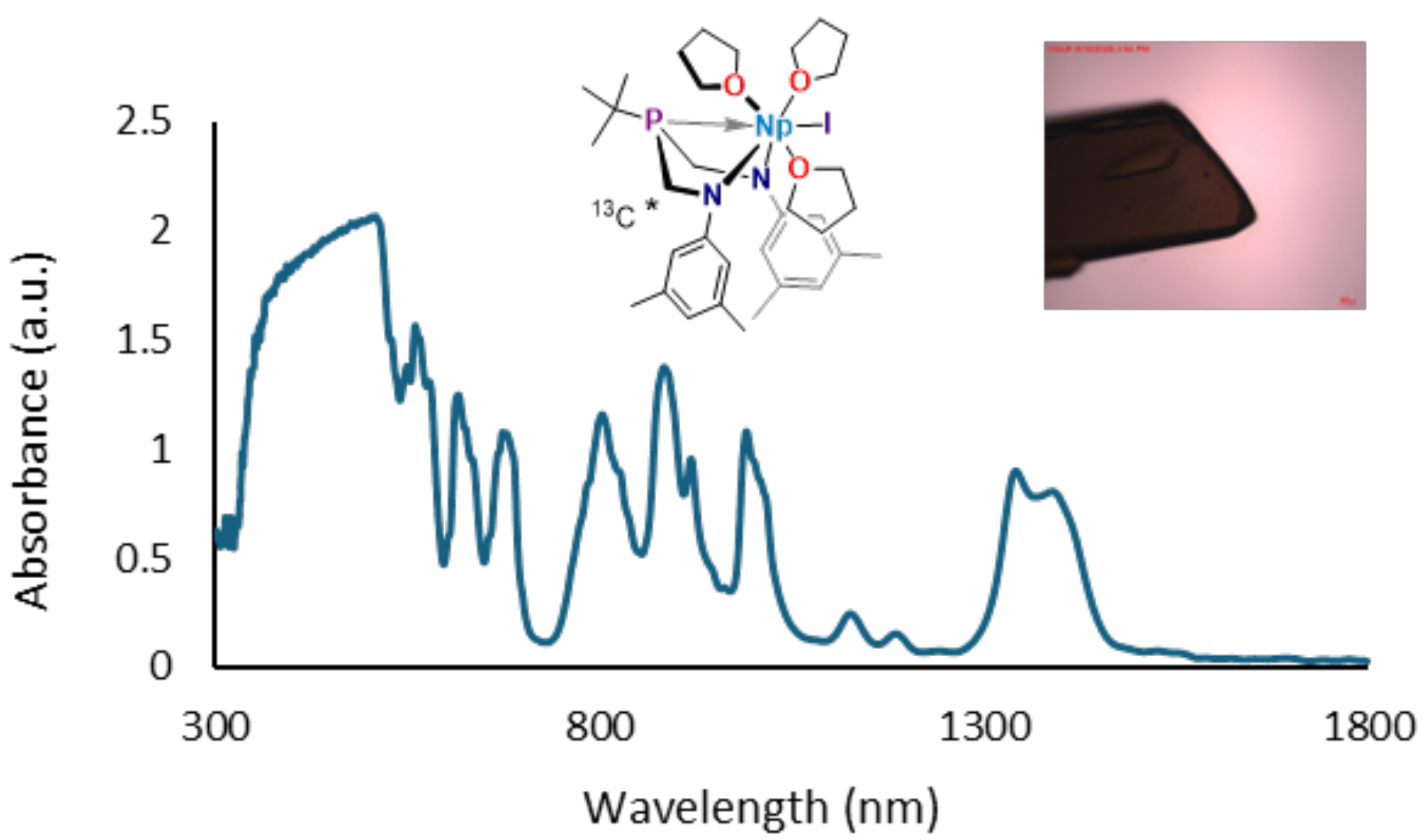

Figure S59. Solid-state UV-vis-NIR spectrum of **NpI(^{13}C$_2$-^{Ar}BiAMP^{tBu})(thf)$_3$** (**^{13}C-3**) recorded at room temperature. The photo depicts the crystal used for the measurement.

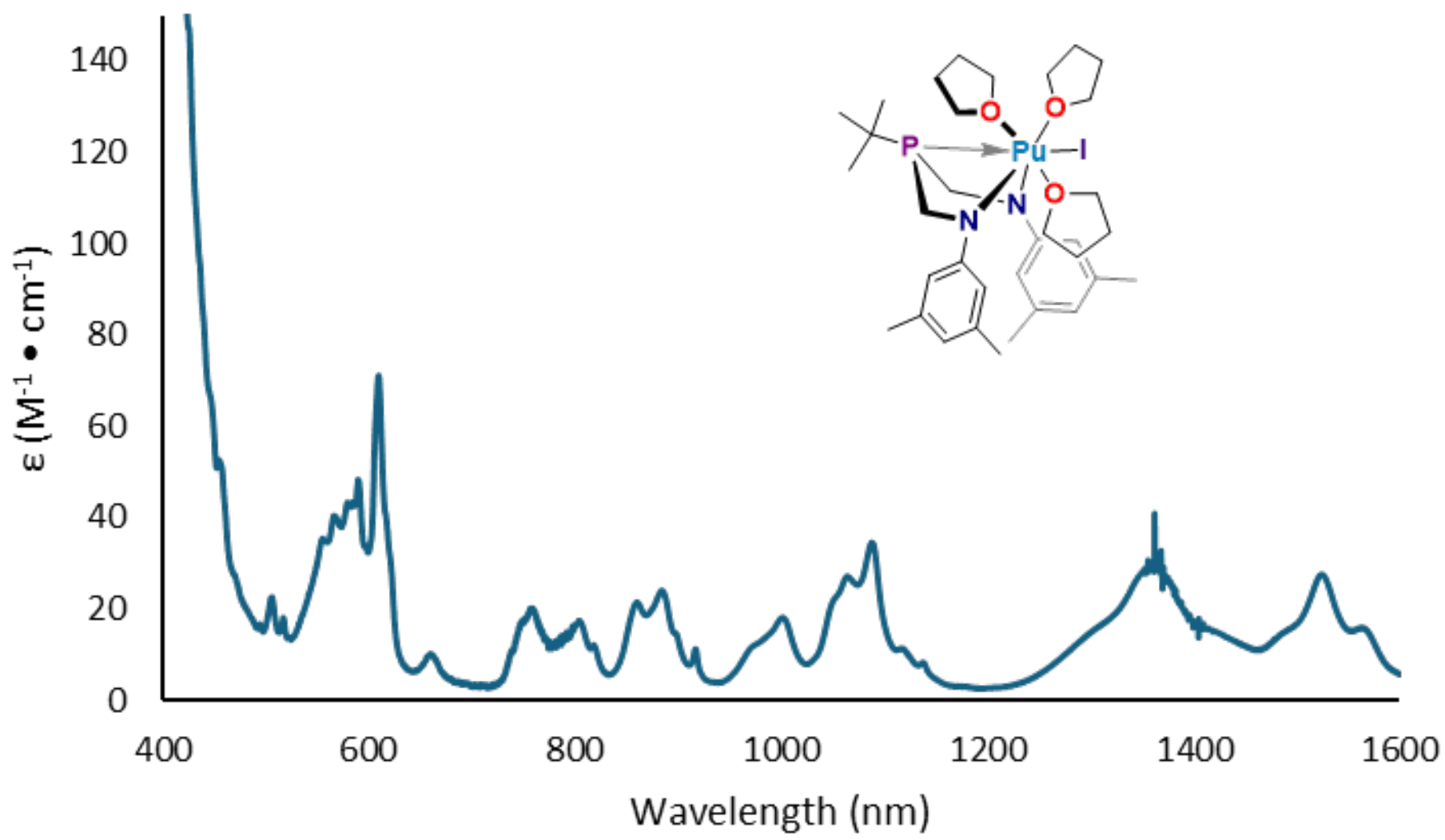

Figure S60. Solution-state vis-NIR spectrum of **PuI(^{Ar}BiAMP^{tBu})(thf)$_3$** (**4**) recorded in THF (2.7 mM) at room temperature.

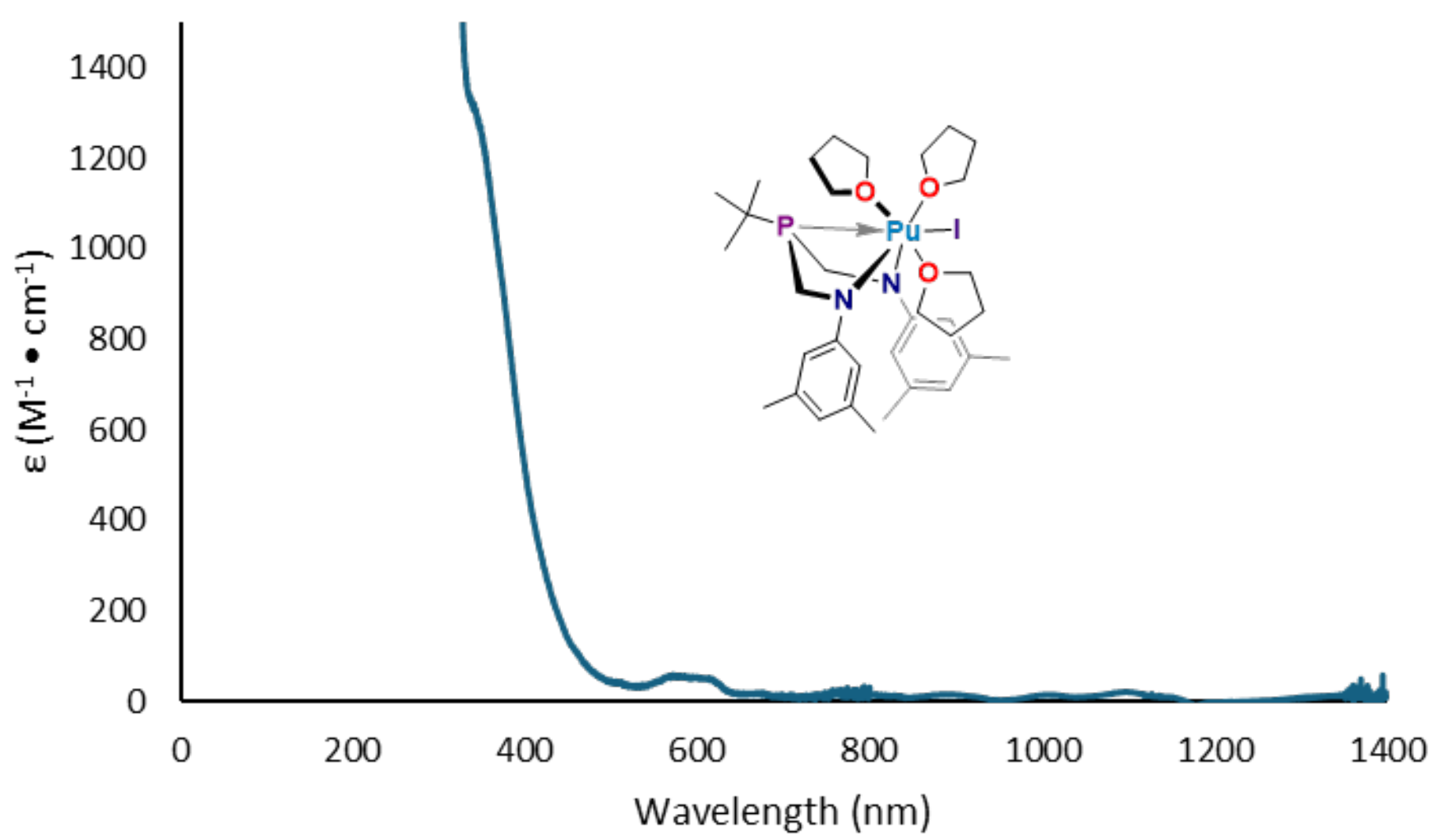

Figure S61. Solution-state UV-vis-NIR spectrum of **PuI(^{Ar}BiAMP^{tBu})(thf)$_3$** (**4**) recorded in THF (0.22 mM) at room temperature.

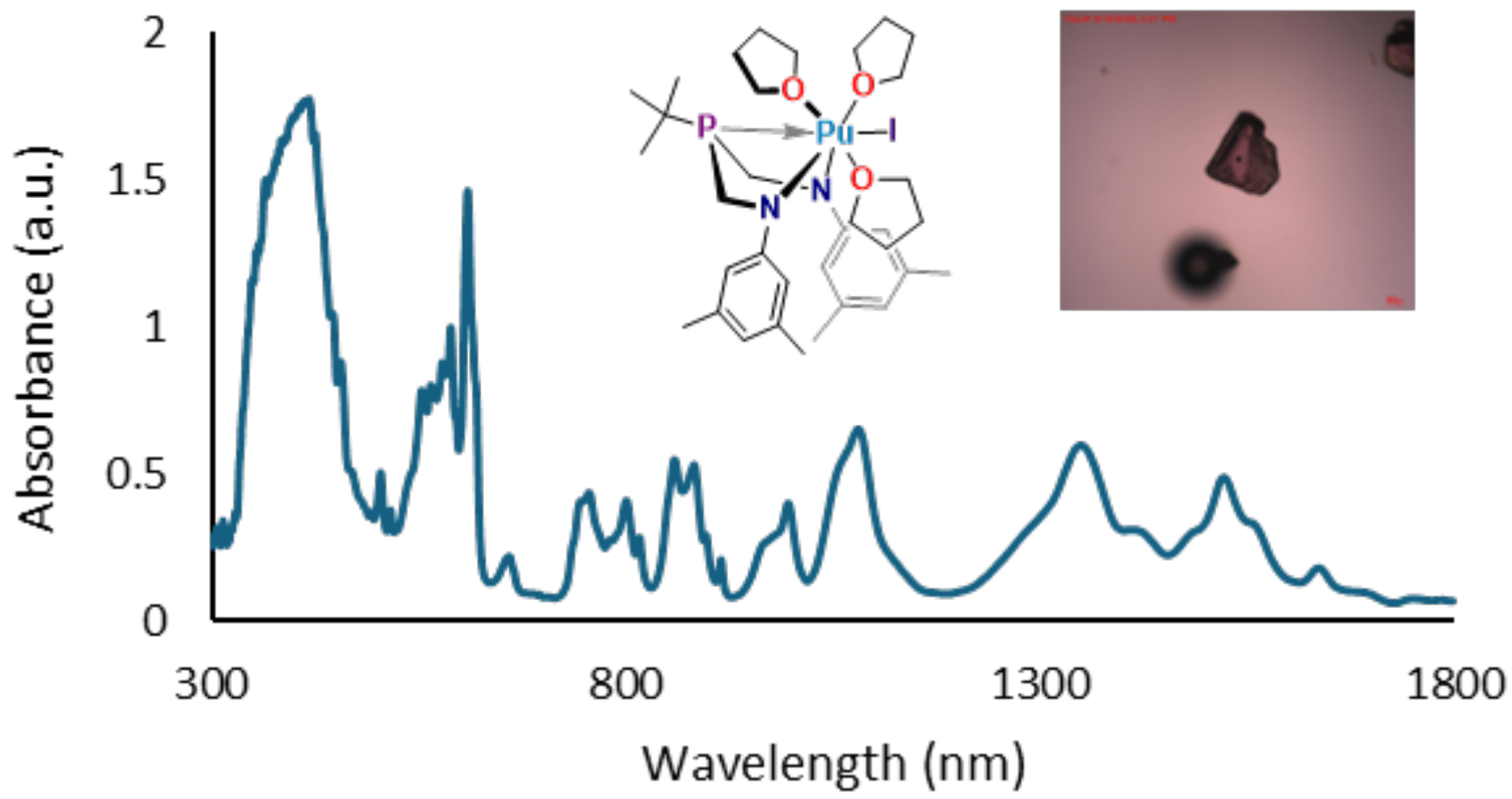

Figure S62. Solid-state UV-vis-NIR spectrum of **PuI(^{Ar}BiAMP^{tBu})(thf)$_3$** (**4**) recorded at room temperature. The photo depicts the crystal used for the measurement.

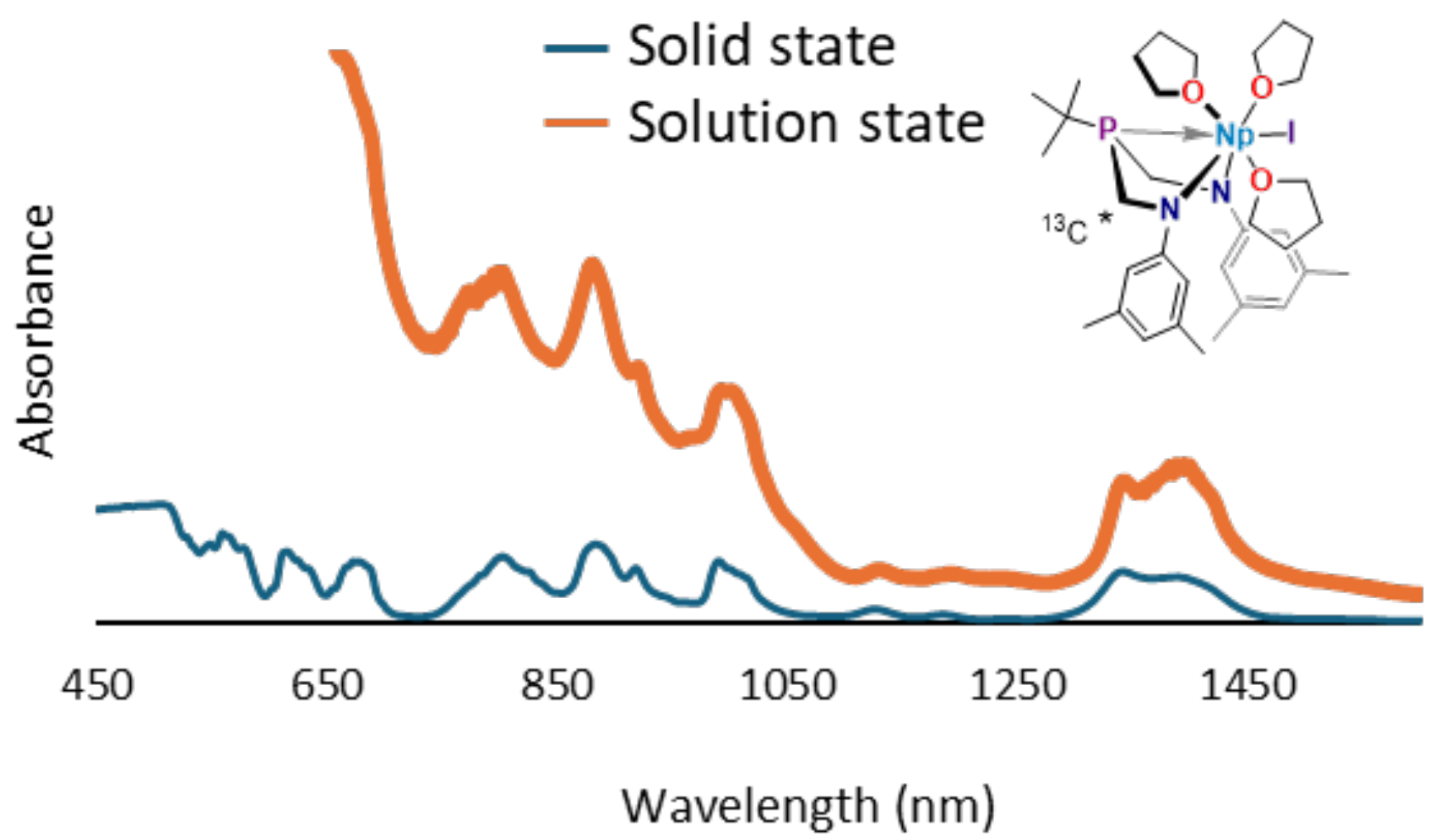

Figure S63. Overlay of the solution- (orange trace) and solid-state (blue trace) vis-NIR spectra of **NpI(^{13}C$_2$-^{Ar}BiAMP^{tBu})(thf)$_3$** (**^{13}C-3**) recorded at room temperature.

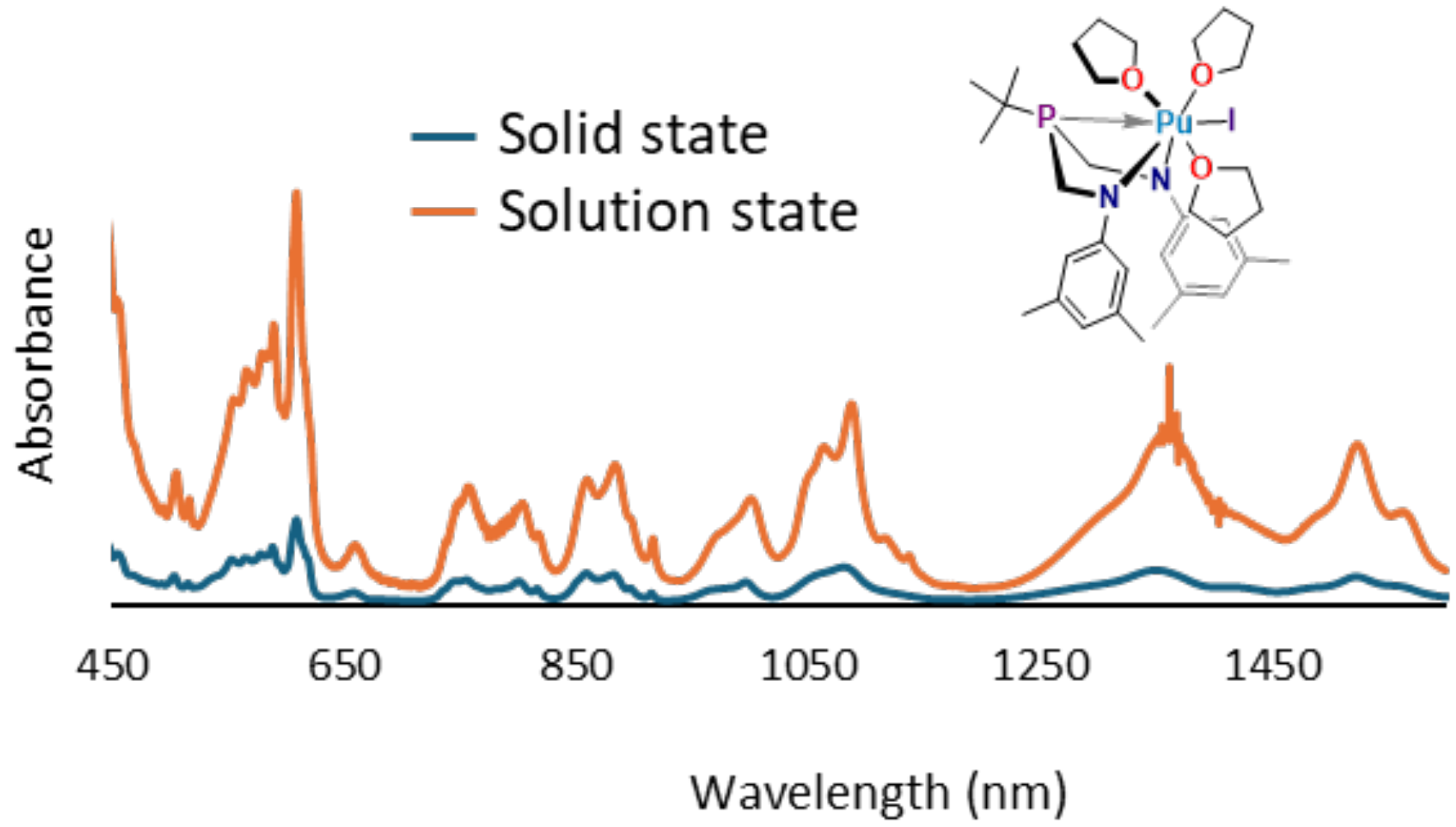

Figure S64. Overlay of the solution- (orange trace) and solid-state (blue trace) vis-NIR spectra of **PuI(^{Ar}BiAMP^{tBu})(thf)$_3$** (**4**) recorded at room temperature.

2.3 Supplemental Correlation Graphs

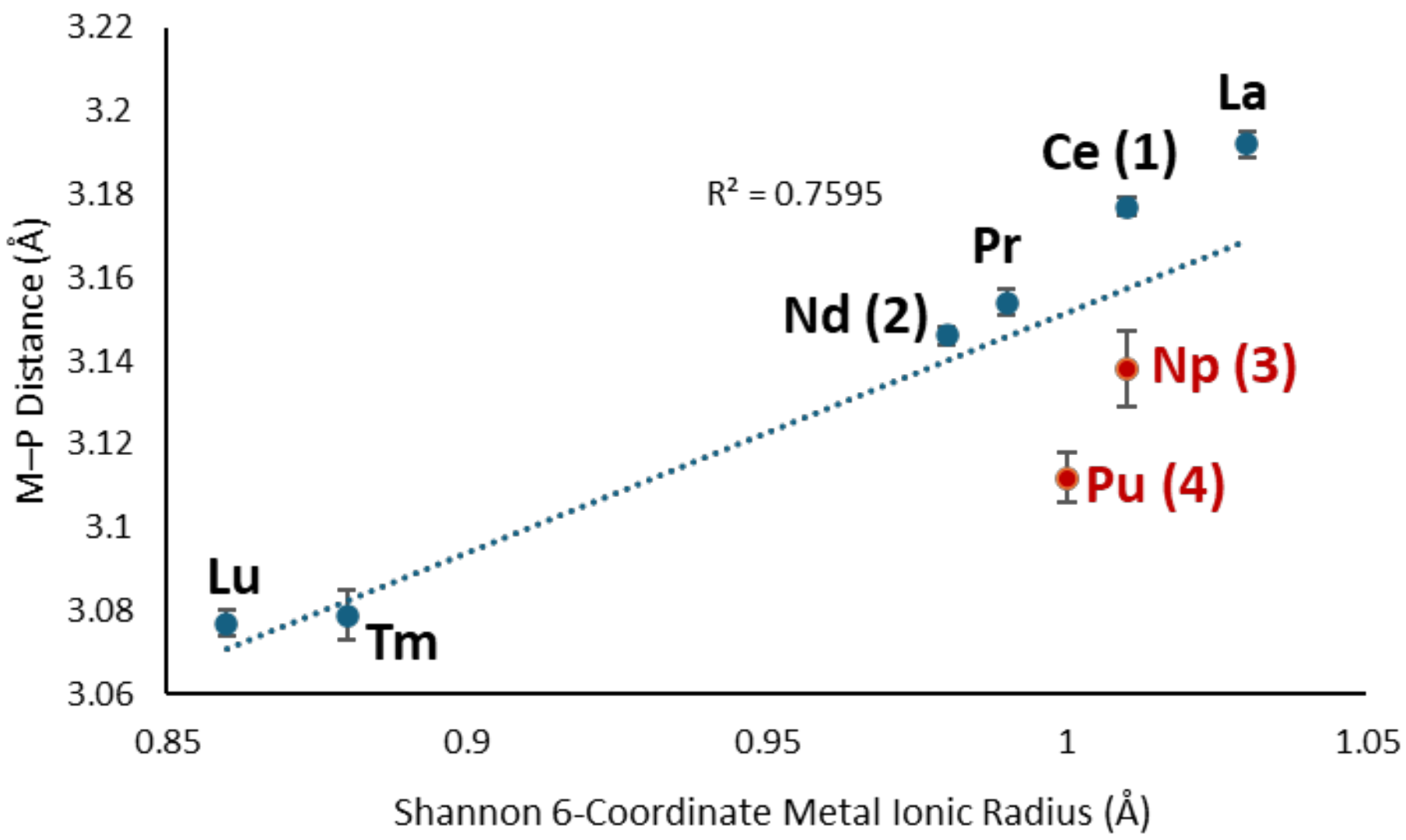

Figure S65. Correlation plot of the M–P distances vs. 6-coordinate trivalent metal ionic radii for complexes **1**–**4** and **MI(^{Ar}BiAMP^{tBu})(thf)$_3$** (M = La, Pr, Tm, Lu). Actinide complexes **3** (Np) and **4** (Pu) are denoted as red points on the graph. Linear regression analysis (R^2 value) reflects both the lanthanide (blue) and actinide (red) members of the plot. Error bars are shown to 3 standard deviations.

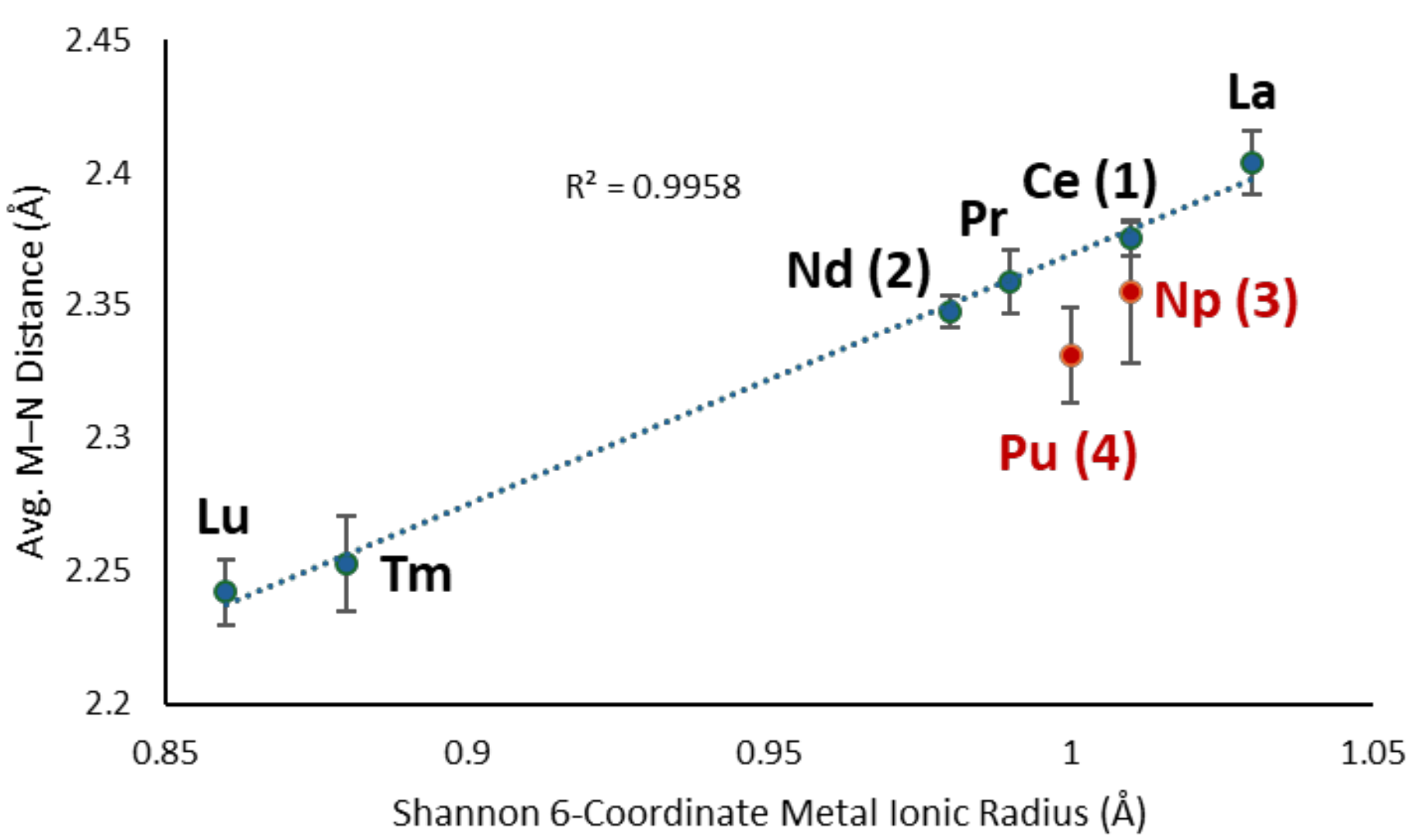

Figure S66. Correlation plot of the average M–N distances vs. 6-coordinate trivalent metal ionic radii for complexes **1**–**4** and **MI(^{Ar}BiAMP^{tBu})(thf)$_3$** (M = La, Pr, Tm, Lu). Actinide complexes **3** (Np) and **4** (Pu) are denoted as red points on the graph. Linear regression analysis (R^2 value) reflects only the lanthanide members denoted as blue points on the plot. Error bars are shown to 3 standard deviations.

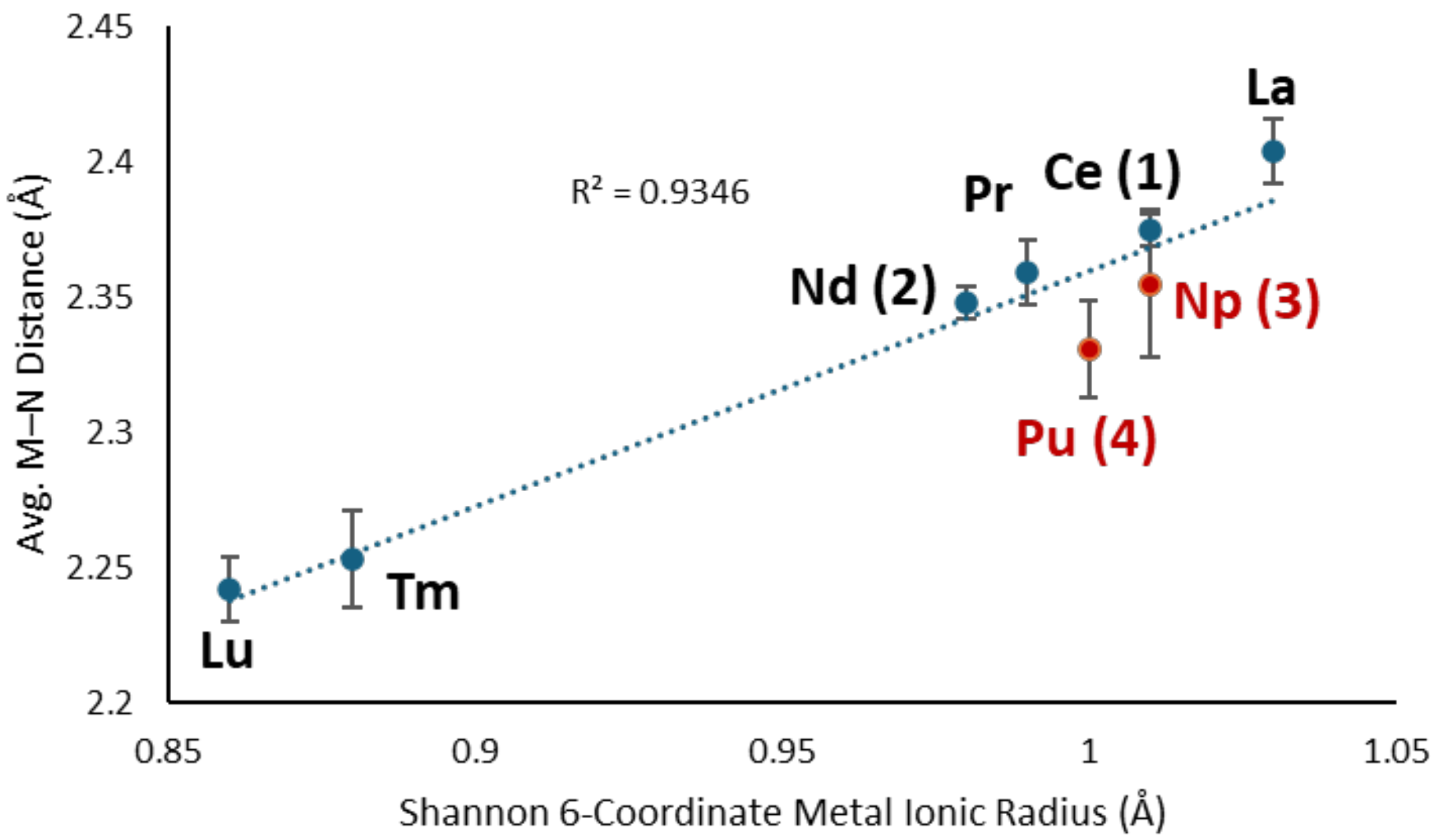

Figure S67. Correlation plot of the average M–N distances vs. 6-coordinate trivalent metal ionic radii for complexes **1**–**4** and **MI(^{Ar}BiAMP^{tBu})(thf)$_3$** (M = La, Pr, Tm, Lu). Actinide complexes **3** (Np) and **4** (Pu) are denoted as red points on the graph. Linear regression analysis (R^2 value) reflects both the lanthanide (blue) and actinide (red) members of the plot. Error bars are shown to 3 standard deviations.

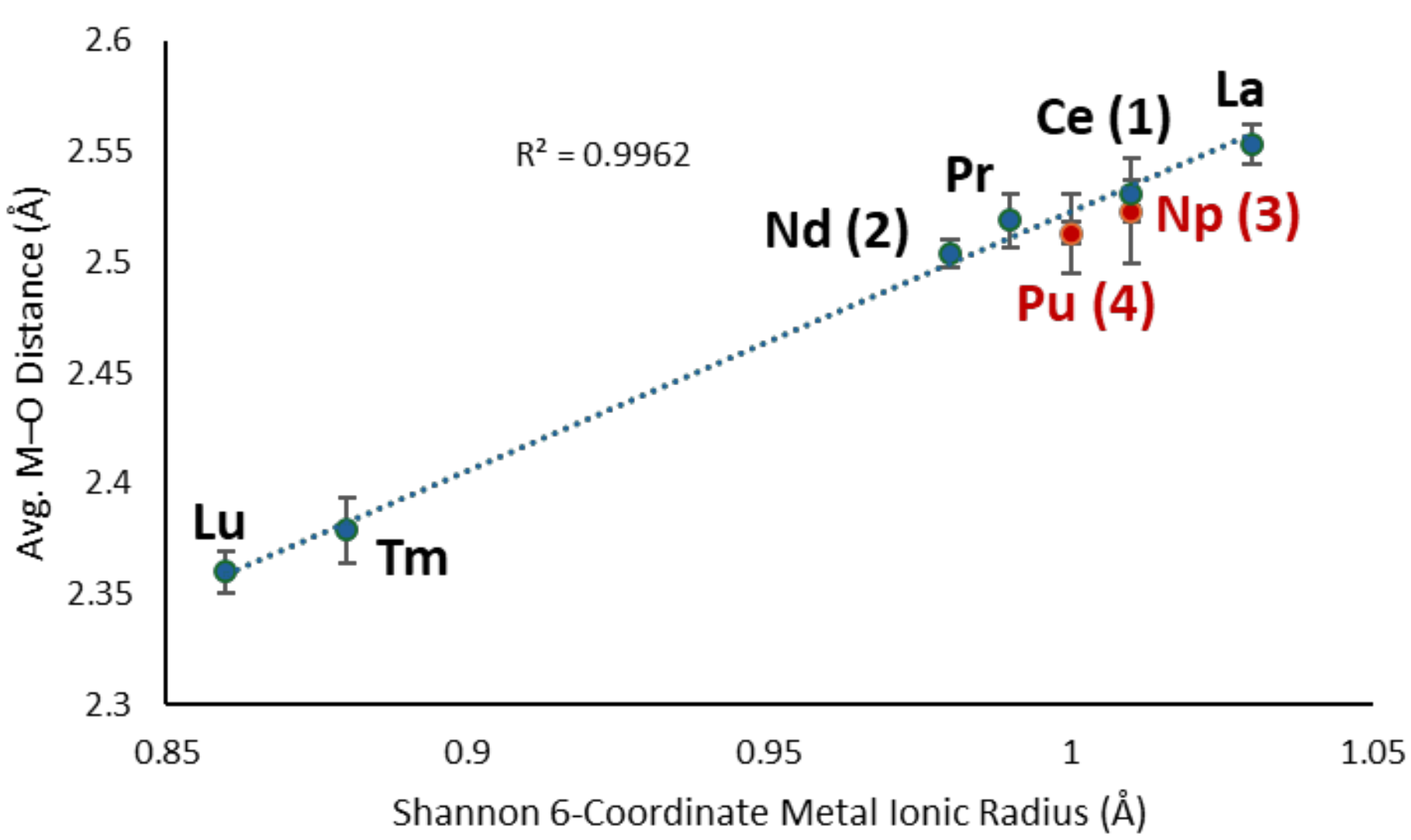

Figure S68. Correlation plot of the average M–O distances vs. 6-coordinate trivalent metal ionic radii for complexes **1**–**4** and **MI(^{Ar}BiAMP^{tBu})(thf)$_3$** (M = La, Pr, Tm, Lu). Actinide complexes **3** (Np) and **4** (Pu) are denoted as red points on the graph. Linear regression analysis (R^2 value) reflects only the lanthanide members denoted as blue points on the plot. Error bars are shown to 3 standard deviations.

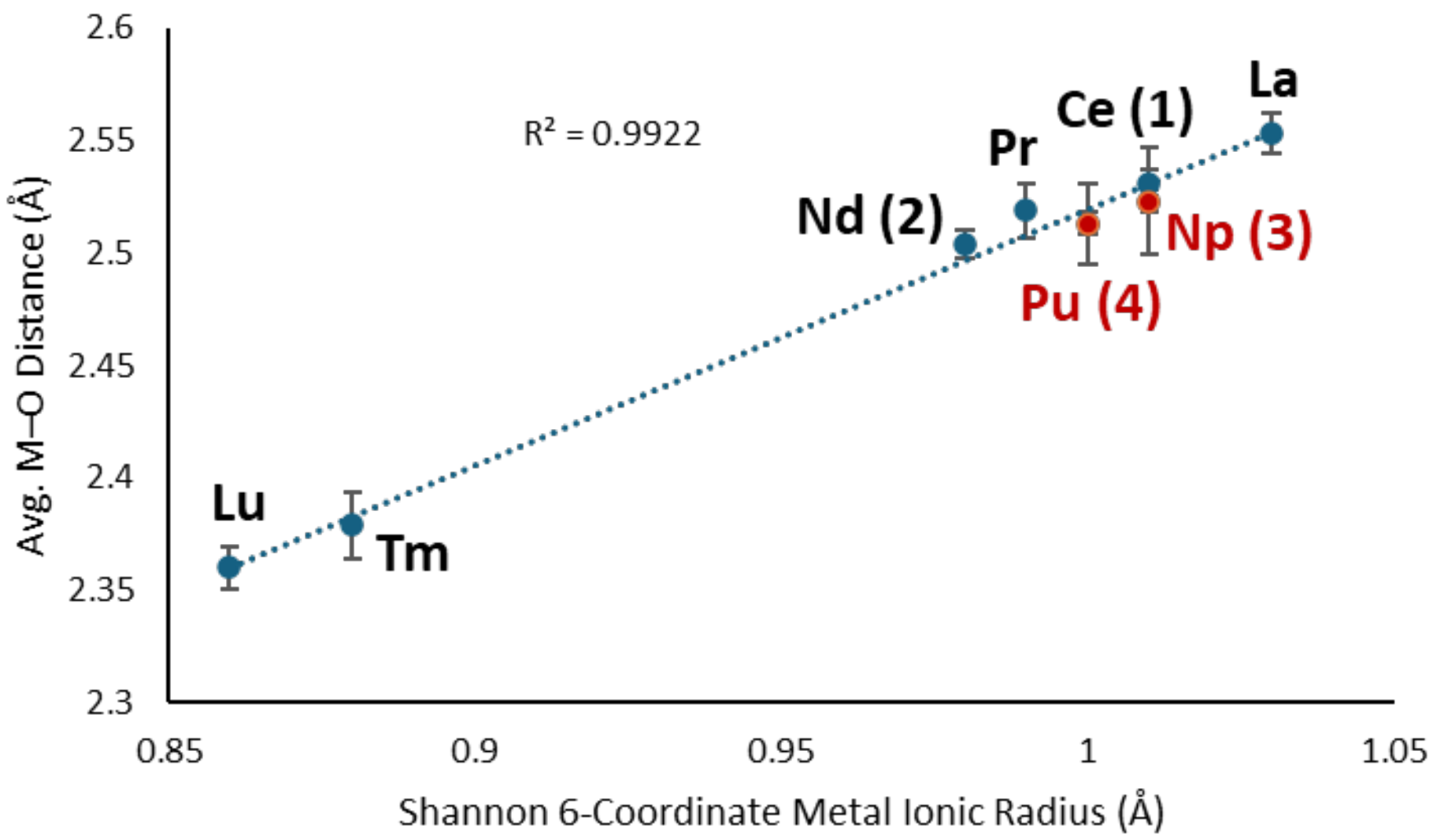

Figure S69. Correlation plot of the average M–O distances vs. 6-coordinate trivalent metal ionic radii for complexes **1**–**4** and **MI(^{Ar}BiAMP^{tBu})(thf)$_3$** (M = La, Pr, Tm, Lu). Actinide complexes **3** (Np) and **4** (Pu) are denoted as red points on the graph. Linear regression analysis (R^2 value) reflects both the lanthanide (blue) and actinide (red) members of the plot. Error bars are shown to 3 standard deviations.

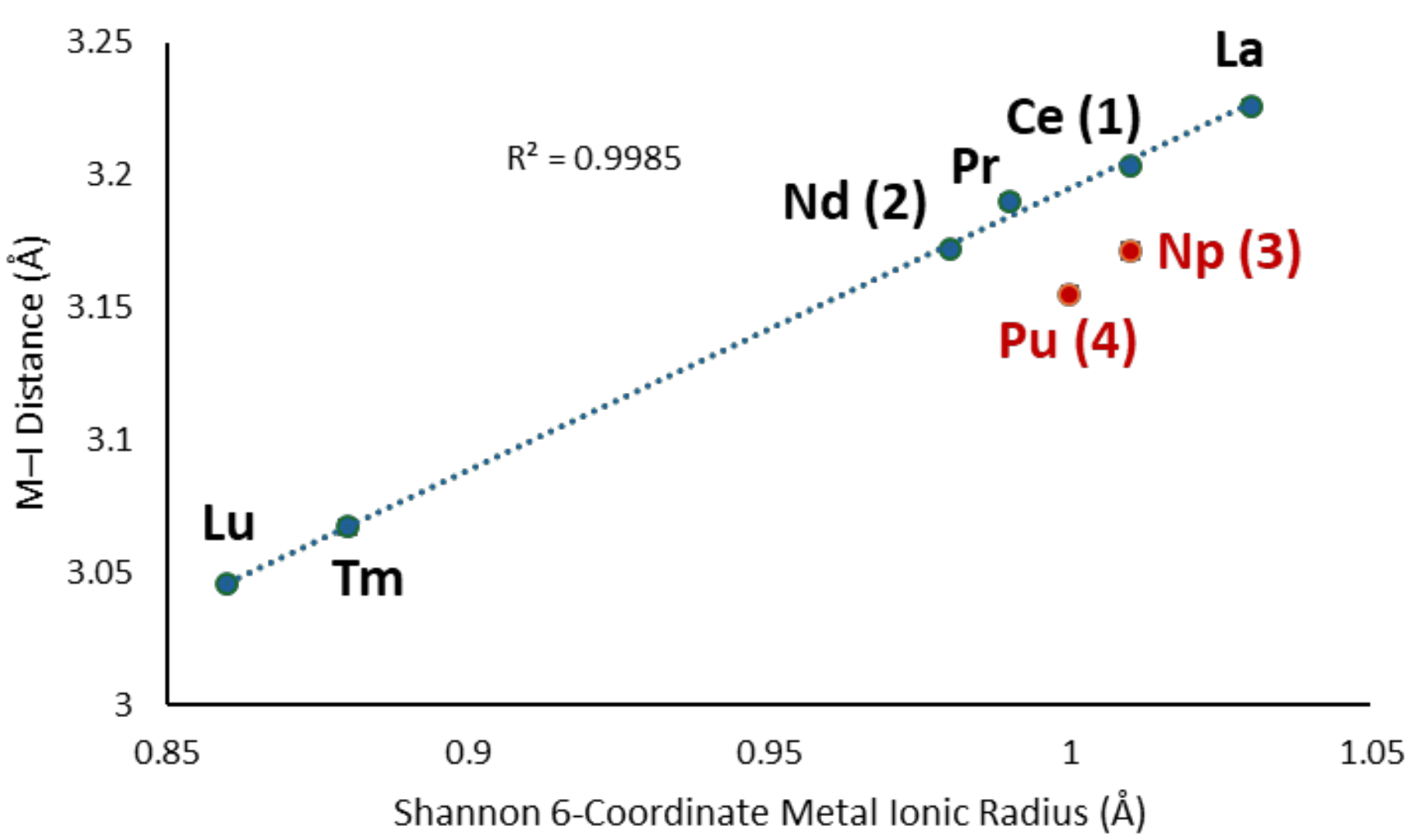

Figure S70. Correlation plot of the M–I distances vs. 6-coordinate trivalent metal ionic radii for complexes **1**–**4** and **MI(^{Ar}BiAMP^{tBu})(thf)$_3$** (M = La, Pr, Tm, Lu). Actinide complexes **3** (Np) and **4** (Pu) are denoted as red points on the graph. Linear regression analysis (R^2 value) reflects only the lanthanide members denoted as blue points on the plot. Error bars are shown to 3 standard deviations.

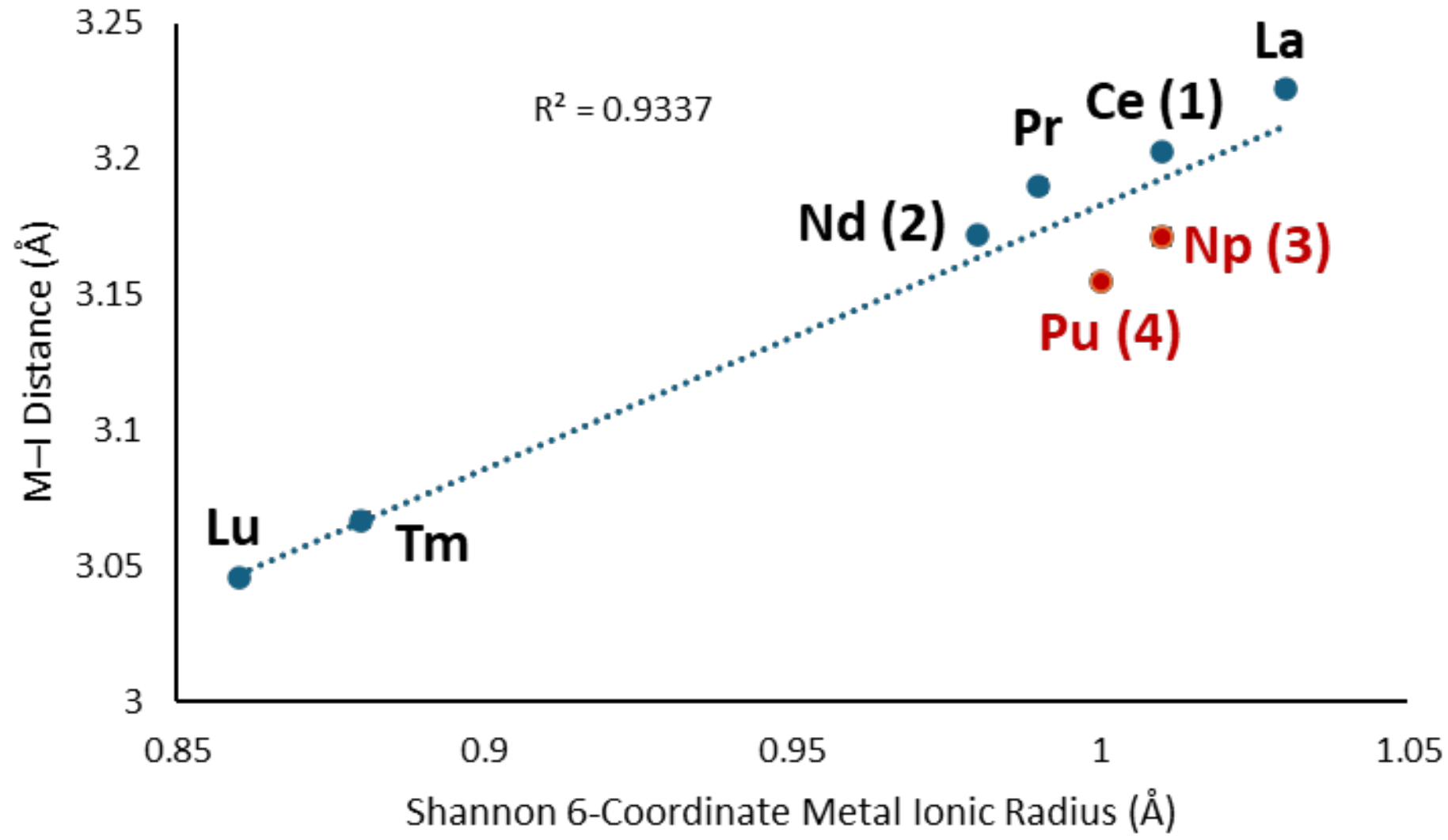

Figure S71. Correlation plot of the M–I distances vs. 6-coordinate trivalent metal ionic radii for complexes **1**–**4** and **MI(^{Ar}BiAMP^{tBu})(thf)$_3$** (M = La, Pr, Tm, Lu). Actinide complexes **3** (Np) and **4** (Pu) are denoted as red points on the graph. Linear regression analysis (R^2 value) reflects both the lanthanide (blue) and actinide (red) members of the plot. Error bars are shown to 3 standard deviations.

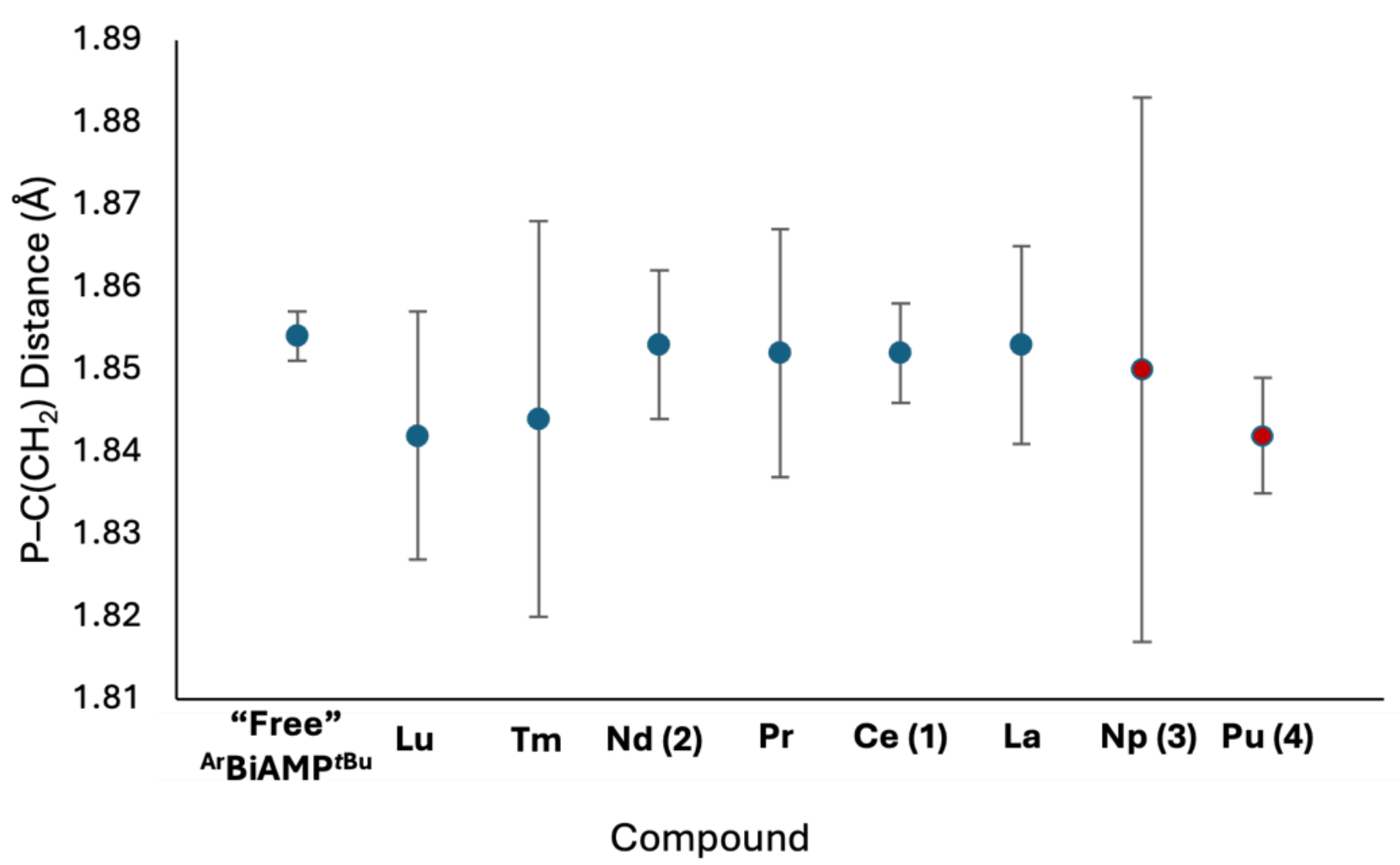

Figure S72. Plot of the average of the P–C(methylene) distances for select 4*f* and 5*f* **MI(^{Ar}BiAMP^{tBu})(thf)$_3$** complexes.

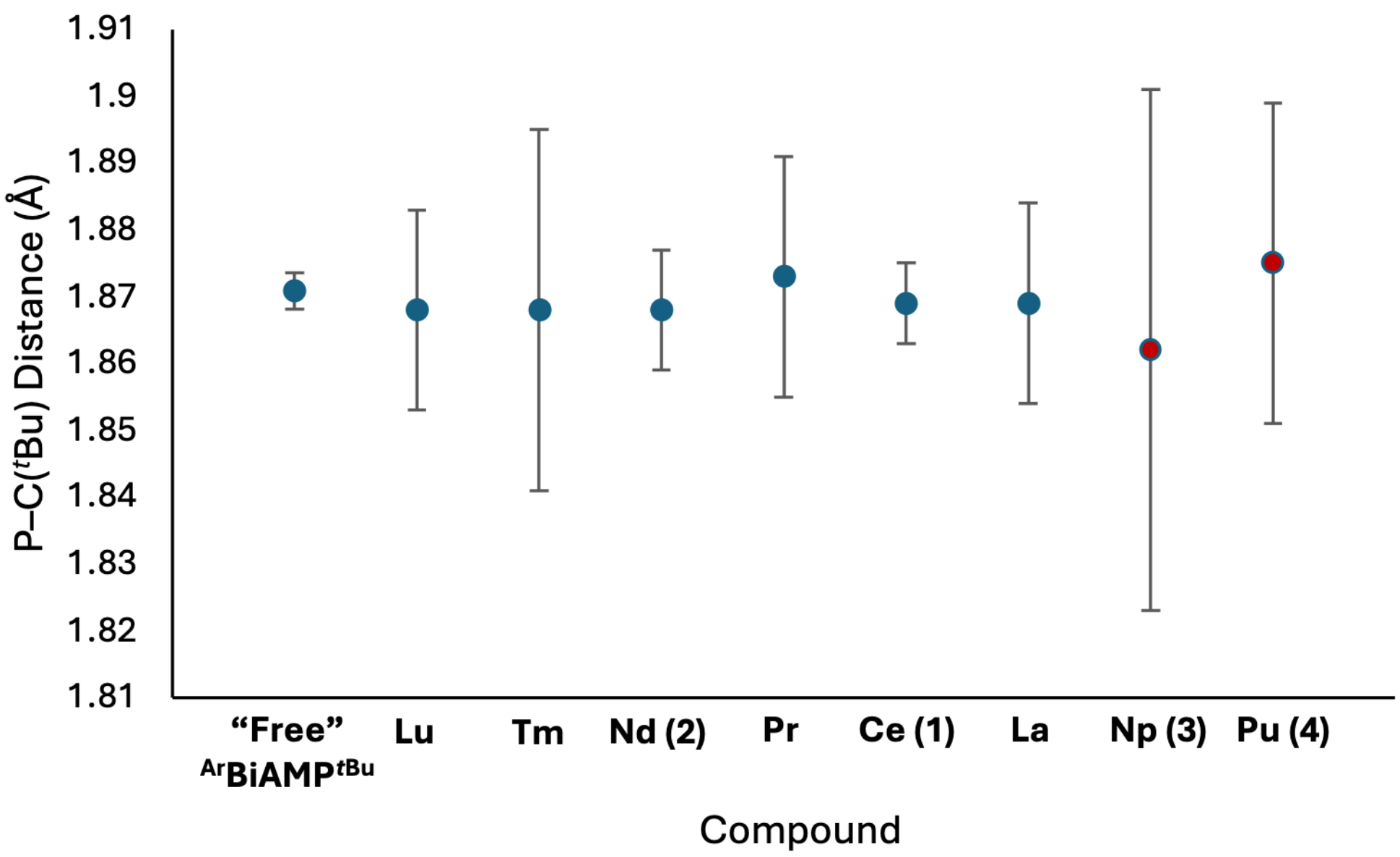

Figure S73. Plot of the average of the P–C(^{tert}Bu) distances for select 4*f* and 5*f* **MI(^{Ar}BiAMP^{tBu})(thf)$_3$** complexes.

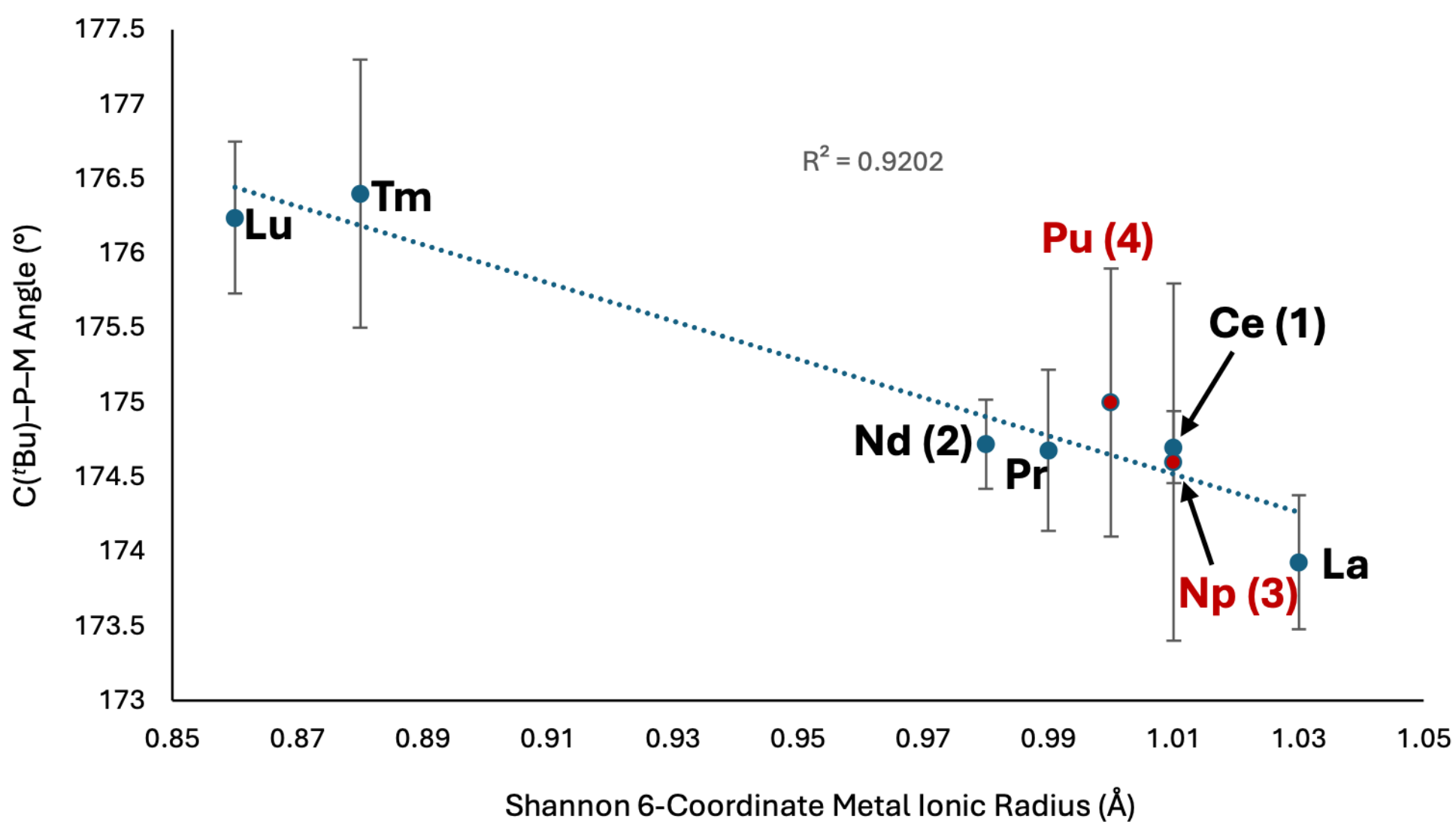

Figure S74. Correlation plot of the average of the C(^{tert}Bu)–P–M angle for select 4*f* and 5*f* **MI(^{Ar}BiAMP^{tBu})(thf)$_3$** complexes.

2.4 Additional P-M Bonding Discussion

Both the central atom and the phosphorus atom found in the complexes $\mathbf{MI(^{Ar}BiAMP^{\mathit{t}Bu})(thf)_3}$ that are reported are found in non-idealized geometries. Attempting to describe the coordination between phosphine and the metal center can vary depending on the rationale employed with the geometric descriptors. Maintaining the normal vector of the "cap" of the geometry of the metal center as the z-axis allows us to compare the effect altering the description of the phosphine has on bonding. The closest description of bonding to that discussed in the manuscript comes from assigning the phosphine as a tetrahedron. This allows sp^3 hybridization of the phosphorus orbitals and poor, off angle overlap with the $5fz^3$ and $6dz^2$ orbitals of the metal. We disfavor this description for several reasons. Requiring sp^3 hybridization of the phosphine orbitals would produce a bent overlap, as well as ignoring the nearly linear C–P–M angle.

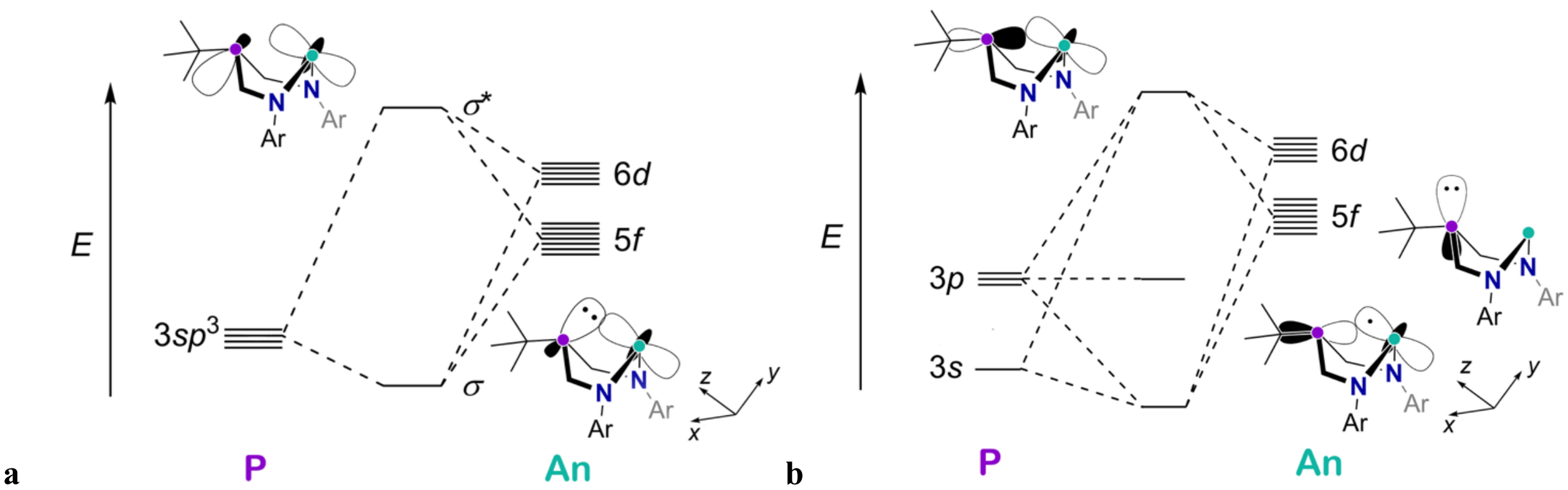

Figure S75. Truncated orbital bonding schemes for disfavored descriptions of the P-M interaction. Left) sp3 hybridized phosphine; Right) seesaw geometry at the phosphine with the lone pair found in an equatorial position.

Alternatively (**Figure S75b**), treating the phosphine as a typical see-saw geometry would see the lone pair in the equatorial position of the phosphine, creating a Lewis-acidic position in the axial position of the phosphorus opposite to the ^{tBu}C. This arrangement necessitates that a singly occupied metal orbital, either the $5fz^3$ and/or $6dz^2$, donate density into that axial position of the phosphorus. We disfavor this option because the metal then becomes the nucleophile in the bonding scheme with the phosphine as the electrophile; the non-bonding lone pair would be expected to be highly basic. We searched the metrical parameters of the P-C bonds—both the ^{tBu}C and methylene CH_2 of the ligand—for evidence of bond elongation or contraction in support of a bonding description, but changes observed in these bond lengths were not significantly (3σ) different from the free ligand (**Figures S72** and **S73**).

3. Single Crystal X-ray Diffraction Data

3.1 CCDC deposition numbers: 2527016 – 2527022

3.2 1,5,3,7-diazadiphosphocane (P_2N_2): 2527016

3.3 CeI(^{Ar}BiAMP^{tBu})(thf)$_3$ (1): 2527017

3.4 NdI(^{Ar}BiAMP^{tBu})(thf)$_3$ (2): 2527018

3.5 NpI(^{Ar}BiAMP^{tBu})(thf)$_3$ (3): 2527019

3.6 PuI(^{Ar}BiAMP^{tBu})(thf)$_3$ (4): 2527020

3.7 PrI(^{Ar}BiAMP^{tBu})(thf)$_3$ —: 2527021

3.8 TmI(^{Ar}BiAMP^{tBu})(thf)$_3$ —: 2527022

3.2 P_2N_2 : 2527016

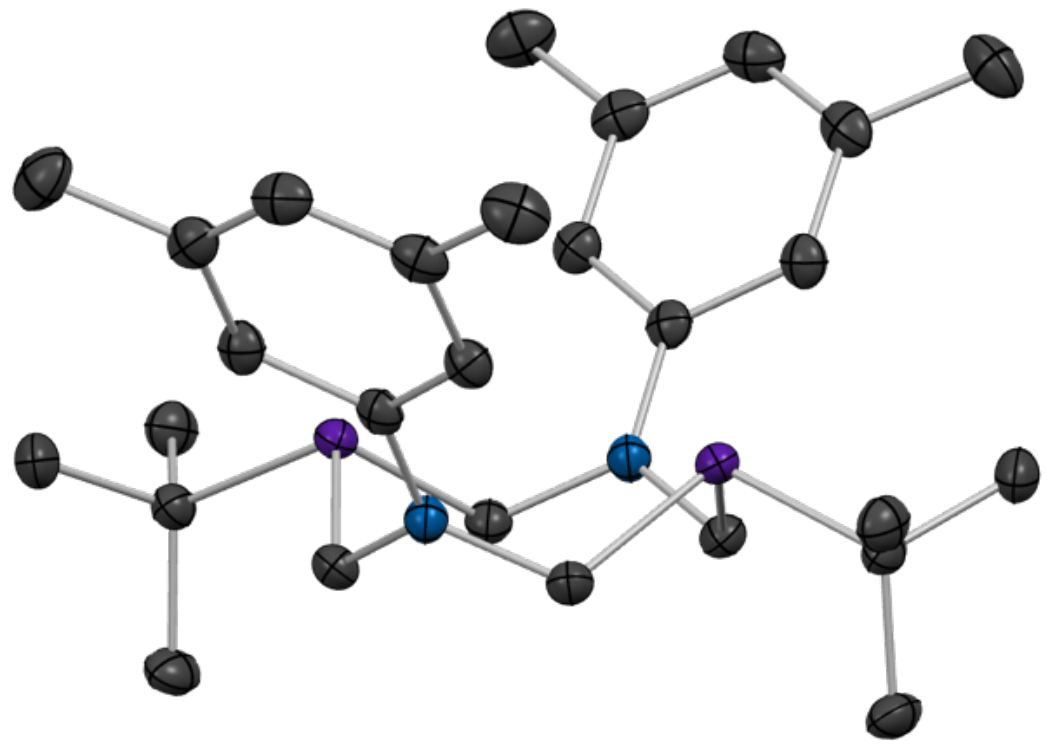

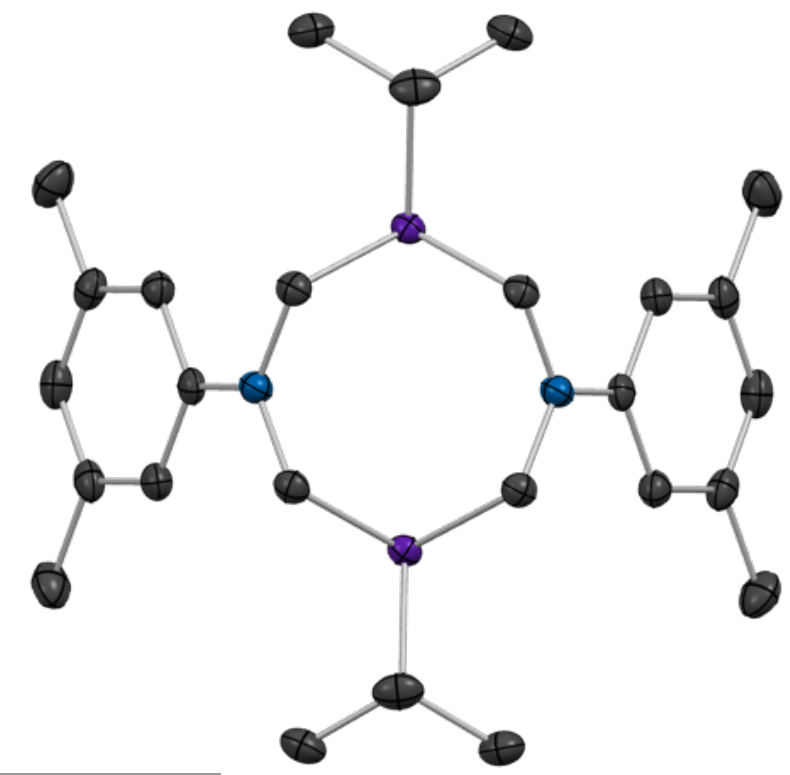

Table S1 Crystal data and structure refinement for P2N2.	
**Identification code**	P2N2
**Empirical formula**	$C_{28}H_{44}N_2P_2$
**Formula weight**	470.59
**Temperature/K**	100.00
**Crystal system**	monoclinic
**Space group**	C2/c
**a/Å**	22.7933(18)
**b/Å**	5.7870(5)
**c/Å**	23.3737(18)
**α/°**	90
**β/°**	118.617(2)
**γ/°**	90
**Volume/Å^3**	2706.5(4)
**Z**	4
**ρ_{calc}g/cm^3**	1.155
**μ/mm^{-1}**	0.179
**F(000)**	1024.0
**Crystal size/mm^3**	0.12 × 0.07 × 0.05
**Radiation**	MoKα (λ = 0.71073)
**2Θ range for data collection/°**	6.918 to 57.76
**Index ranges**	-30 ≤ h ≤ 30, -7 ≤ k ≤ 7, -31 ≤ l ≤ 31
**Reflections collected**	24825
**Independent reflections**	3528 [R_{int} = 0.0532, R_{sigma} = 0.0302]
**Data/restraints/parameters**	3528/0/150
**Goodness-of-fit on F^2**	1.041
**Final R indexes [I>=2σ (I)]**	R_1 = 0.0344, wR_2 = 0.0881
**Final R indexes [all data]**	R_1 = 0.0454, wR_2 = 0.0949
**Largest diff. peak/hole / e Å^{-3}**	0.31/-0.26

Table S2 Bond Lengths for P2N2.

Atom	Atom	Length/Å		Atom	Atom	Length/Å
**P2**	C11	1.8854(13)		C11	C14	1.5400(18)
**P2**	C10	1.8792(12)		C11	C13	1.5343(18)
**P2**	C9^1	1.8793(12)		C5	C6	1.3938(18)
**N1**	C4	1.3856(16)		C6	C1	1.389(2)
**N1**	C10	1.4536(15)		C6	C8	1.5101(18)
**N1**	C9	1.4508(15)		C2	C3	1.3916(18)
**C4**	C5	1.4100(17)		C2	C1	1.3898(19)
**C4**	C3	1.4086(17)		C2	C7	1.5131(19)
**C11**	C12	1.5384(18)				

Table S3 Bond Angles for P2N2.

Atom	Atom	Atom	Angle/˚		Atom	Atom	Atom	Angle/˚
**C10**	P2	C11	101.03(5)		C13	C11	C12	109.65(11)

**C10**	P2	C9[1]	98.80(6)		C13	C11	C14	109.64(11)
**C9[1]**	P2	C11	100.59(5)		N1	C10	P2	112.88(8)
**C4**	N1	C10	121.01(10)		C6	C5	C4	121.04(12)
**C4**	N1	C9	121.01(10)		N1	C9	P2[1]	113.53(8)
**C9**	N1	C10	117.90(10)		C5	C6	C8	119.61(13)
**N1**	C4	C5	121.22(11)		C1	C6	C5	119.98(12)
**N1**	C4	C3	121.18(11)		C1	C6	C8	120.40(13)
**C3**	C4	C5	117.59(11)		C3	C2	C7	119.83(13)
**C12**	C11	P2	107.75(9)		C1	C2	C3	119.85(12)
**C12**	C11	C14	108.21(10)		C1	C2	C7	120.31(13)
**C14**	C11	P2	107.31(9)		C2	C3	C4	121.30(12)
**C13**	C11	P2	114.09(9)		C6	C1	C2	120.22(12)

3.3 CeI(^{Ar}BiAMP^{tBu})(thf)$_3$: 2527017

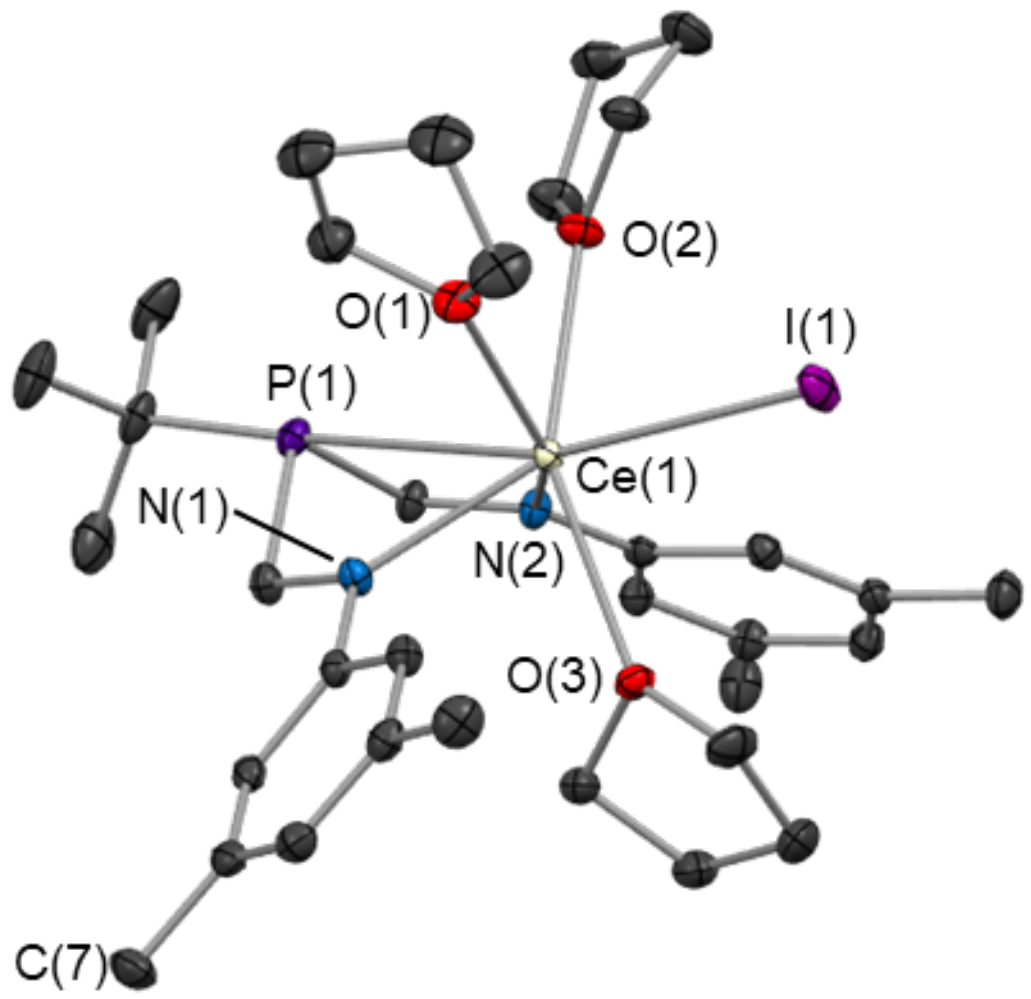

Table S4 Crystal data and structure refinement for (^{Ar}BiAMP^{tBu})CeI(thf)$_3$ (1).	
**Identification code**	CeBiAMP
**Empirical formula**	$C_{34}H_{55}CeIN_2O_3P$
**Formula weight**	837.79
**Temperature/K**	100.0
**Crystal system**	orthorhombic
**Space group**	$P2_12_12_1$
**a/Å**	13.313(2)
**b/Å**	14.519(2)
**c/Å**	19.166(3)
**α/°**	90
**β/°**	90
**γ/°**	90
**Volume/Å^3**	3704.5(10)
**Z**	4
**ρ_{calc}g/cm^3**	1.502
**μ/mm^{-1}**	2.138
**F(000)**	1692.0
**Crystal size/mm^3**	0.31 × 0.25 × 0.17
**Radiation**	MoKα (λ = 0.71073)
**2Θ range for data collection/°**	5.942 to 61.016
**Index ranges**	-19 ≤ h ≤ 19, -20 ≤ k ≤ 20, -27 ≤ l ≤ 27
**Reflections collected**	73388
**Independent reflections**	11272 [R_{int} = 0.0274, R_{sigma} = 0.0196]
**Data/restraints/parameters**	11272/0/386
**Goodness-of-fit on F^2**	1.094
**Final R indexes [I>=2σ (I)]**	R_1 = 0.0149, wR_2 = 0.0361
**Final R indexes [all data]**	R_1 = 0.0150, wR_2 = 0.0362
**Largest diff. peak/hole / e Å^{-3}**	1.36/-0.88
**Flack parameter**	0.007(3)

Table S5 Bond Lengths for CeI(^{Ar}BiAMP^{tBu})(thf)$_3$ (1).

Atom	Atom	Length/Å		Atom	Atom	Length/Å
**Ce1**	I1	3.2032(4)		C19	C20	1.533(4)
**Ce1**	P1	3.1773(7)		C19	C21	1.532(3)
**Ce1**	O1	2.5356(17)		C16	C13	1.505(3)
**Ce1**	O2	2.5471(15)		C23	C24	1.532(3)
**Ce1**	O3	2.5104(16)		C14	C13	1.374(3)
**Ce1**	N2	2.3775(18)		C14	C9	1.418(3)
**Ce1**	N1	2.3730(17)		C28	C27	1.515(3)
**P1**	C19	1.869(2)		C13	C12	1.404(3)

**P1**	C18	1.849(2)		N2	C18	1.457(3)
**P1**	C17	1.855(2)		N2	C9	1.381(3)
**O1**	C23	1.454(3)		C10	C9	1.411(3)
**O1**	C26	1.443(3)		C8	C5	1.509(3)
**O2**	C27	1.463(3)		C24	C25	1.519(5)
**O2**	C30	1.454(3)		C26	C25	1.501(4)
**O3**	C31	1.451(3)		C4	C5	1.400(3)
**O3**	C34	1.451(3)		C4	C3	1.393(3)
**C29**	C28	1.523(4)		C33	C34	1.519(4)
**C29**	C30	1.513(3)		N1	C17	1.453(3)
**C32**	C31	1.518(3)		N1	C1	1.378(3)
**C32**	C33	1.535(4)		C6	C5	1.383(3)
**C11**	C10	1.398(3)		C6	C1	1.416(3)
**C11**	C12	1.386(3)		C2	C1	1.409(3)
**C11**	C15	1.509(3)		C2	C3	1.389(3)
**C19**	C22	1.533(4)		C7	C3	1.509(3)

Table S6 Bond Angles for CeI(^{Ar}BiAMP^{tBu})(thf)$_3$ (1).

Atom	Atom	Atom	Angle/˚		Atom	Atom	Atom	Angle/˚
**P1**	Ce1	I1	162.685(12)		C20	C19	P1	112.92(17)
**O1**	Ce1	I1	83.22(4)		C21	C19	P1	107.06(16)
**O1**	Ce1	P1	92.64(4)		C21	C19	C22	108.6(2)
**O1**	Ce1	O2	76.03(6)		C21	C19	C20	110.0(2)
**O2**	Ce1	I1	82.54(4)		O1	C23	C24	105.7(2)
**O2**	Ce1	P1	80.14(4)		C13	C14	C9	122.53(19)
**O3**	Ce1	I1	81.14(4)		C27	C28	C29	103.9(2)
**O3**	Ce1	P1	113.81(4)		C14	C13	C16	120.7(2)
**O3**	Ce1	O1	129.90(5)		C14	C13	C12	119.43(19)
**O3**	Ce1	O2	146.84(5)		C12	C13	C16	119.83(19)
**N2**	Ce1	I1	122.09(4)		C18	N2	Ce1	120.74(13)
**N2**	Ce1	P1	54.50(4)		C9	N2	Ce1	122.75(13)
**N2**	Ce1	O1	142.31(6)		C9	N2	C18	116.48(17)
**N2**	Ce1	O2	80.05(6)		C11	C10	C9	121.33(19)
**N2**	Ce1	O3	84.50(6)		C25	C24	C23	103.5(2)
**N1**	Ce1	I1	140.05(4)		C11	C12	C13	120.05(19)
**N1**	Ce1	P1	55.54(4)		O2	C27	C28	106.7(2)
**N1**	Ce1	O1	84.73(6)		O1	C26	C25	104.3(2)
**N1**	Ce1	O2	130.65(6)		C26	C25	C24	101.9(2)
**N1**	Ce1	O3	77.87(6)		N2	C18	P1	105.45(14)
**N1**	Ce1	N2	89.32(6)		O3	C31	C32	104.94(18)
**C19**	P1	Ce1	174.70(8)		C3	C4	C5	119.7(2)
**C18**	P1	Ce1	79.22(7)		C34	C33	C32	104.4(2)
**C18**	P1	C19	105.94(10)		N2	C9	C14	118.33(18)
**C18**	P1	C17	100.75(10)		N2	C9	C10	125.12(19)
**C17**	P1	Ce1	75.98(7)		C10	C9	C14	116.56(19)
**C17**	P1	C19	103.89(11)		C17	N1	Ce1	115.22(12)
**C23**	O1	Ce1	119.49(14)		C1	N1	Ce1	126.65(13)
**C26**	O1	Ce1	131.15(16)		C1	N1	C17	115.44(17)
**C26**	O1	C23	108.99(19)		N1	C17	P1	107.74(14)
**C27**	O2	Ce1	123.98(14)		O2	C30	C29	104.56(19)
**C30**	O2	Ce1	127.35(13)		C5	C6	C1	122.0(2)
**C30**	O2	C27	108.67(17)		C3	C2	C1	121.5(2)
**C31**	O3	Ce1	119.29(12)		C4	C5	C8	119.9(2)
**C34**	O3	Ce1	136.56(14)		C6	C5	C8	120.4(2)
**C34**	O3	C31	104.13(17)		C6	C5	C4	119.7(2)
**C30**	C29	C28	102.5(2)		O3	C34	C33	104.5(2)
**C31**	C32	C33	104.3(2)		N1	C1	C6	118.73(19)
**C10**	C11	C15	119.9(2)		N1	C1	C2	124.47(19)
**C12**	C11	C10	120.08(19)		C2	C1	C6	116.77(19)
**C12**	C11	C15	120.0(2)		C4	C3	C7	120.4(2)
**C22**	C19	P1	107.78(18)		C2	C3	C4	120.2(2)
**C22**	C19	C20	110.3(2)		C2	C3	C7	119.4(2)

3.4 NdI(^{Ar}BiAMP^{tBu})(thf)$_3$: 2527018

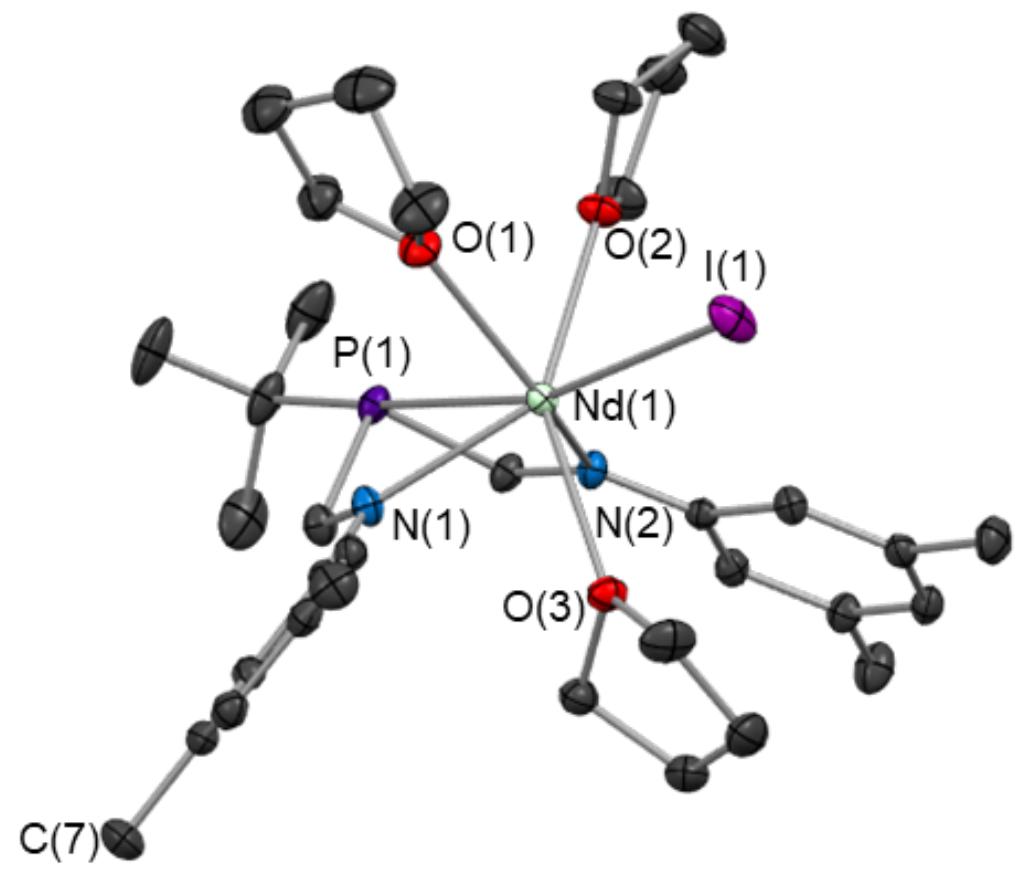

Table S7 Crystal data and structure refinement for NdI(^{Ar}BiAMP^{tBu})(thf)$_3$ (2).	
**Identification code**	BiAMPNdI
**Empirical formula**	$C_{34}H_{55}IN_2NdO_3P$
**Formula weight**	841.91
**Temperature/K**	100.0
**Crystal system**	orthorhombic
**Space group**	$P2_12_12_1$
**a/Å**	13.3405(4)
**b/Å**	14.4692(4)
**c/Å**	19.1457(5)
**α/°**	90
**β/°**	90
**γ/°**	90
**Volume/Å^3**	3695.62(18)
**Z**	4
**ρ_{calc}g/cm^3**	1.513
**μ/mm^{-1}**	2.316
**F(000)**	1700.0
**Crystal size/mm^3**	0.28 × 0.28 × 0.25
**Radiation**	MoKα (λ = 0.71073)
**2Θ range for data collection/°**	4.666 to 66.26
**Index ranges**	-20 ≤ h ≤ 19, -22 ≤ k ≤ 22, -29 ≤ l ≤ 29
**Reflections collected**	112609
**Independent reflections**	14063 [R_{int} = 0.0597, R_{sigma} = 0.0339]
**Data/restraints/parameters**	14063/0/387
**Goodness-of-fit on F^2**	1.036
**Final R indexes [I>=2σ (I)]**	R_1 = 0.0241, wR_2 = 0.0584
**Final R indexes [all data]**	R_1 = 0.0270, wR_2 = 0.0590
**Largest diff. peak/hole / e Å^{-3}**	2.65/-0.70
**Flack parameter**	0.029(9)

Table S8 Bond Lengths for NdI(^{Ar}BiAMP^{tBu})(thf)$_3$ (2).

Atom	Atom	Length/Å		Atom	Atom	Length/Å
**Nd01**	I1	3.1724(2)		C11	C12	1.383(4)
**Nd01**	P1	3.1461(7)		C6	C1	1.412(4)
**Nd01**	O1	2.508(2)		C6	C5	1.386(4)
**Nd01**	O3	2.485(2)		C16	C13	1.511(4)
**Nd01**	O2	2.520(2)		C19	C22	1.528(5)
**Nd01**	N1	2.349(2)		C19	C21	1.534(4)
**Nd01**	N2	2.347(2)		C19	C20	1.536(5)
**P1**	C18	1.850(3)		C31	C32	1.521(4)
**P1**	C17	1.855(3)		C7	C3	1.512(4)
**P1**	C19	1.868(3)		C13	C14	1.383(4)

**O1**	C23	1.449(4)		C13	C12	1.403(4)
**O1**	C26	1.450(4)		C30	C29	1.525(5)
**O3**	C31	1.456(4)		C2	C1	1.404(4)
**O3**	C34	1.446(4)		C2	C3	1.387(4)
**O2**	C27	1.463(4)		C14	C9	1.416(4)
**O2**	C30	1.456(4)		C29	C28	1.522(5)
**N1**	C17	1.454(4)		C34	C33	1.516(5)
**N1**	C1	1.380(4)		C10	C9	1.411(4)
**N2**	C18	1.449(4)		C8	C5	1.514(4)
**N2**	C9	1.387(4)		C5	C4	1.396(4)
**C27**	C28	1.510(5)		C3	C4	1.388(4)
**C23**	C24	1.522(5)		C26	C25	1.499(6)
**C11**	C15	1.501(4)		C24	C25	1.530(6)
**C11**	C10	1.400(4)		C32	C33	1.532(5)

Table S9 Bond Angles for NdI(^{Ar}BiAMP^{tBu})(thf)$_3$ (2).

**Atom**	**Atom**	**Atom**	**Angle/˚**		**Atom**	**Atom**	**Atom**	**Angle/˚**
**P1**	Nd01	I1	162.491(16)		O2	C27	C28	107.0(3)
**O1**	Nd01	I1	83.10(6)		O1	C23	C24	106.0(3)
**O1**	Nd01	P1	92.01(6)		C10	C11	C15	120.1(3)
**O1**	Nd01	O2	76.08(8)		C12	C11	C15	119.8(3)
**O3**	Nd01	I1	80.76(5)		C12	C11	C10	120.1(3)
**O3**	Nd01	P1	114.69(5)		C5	C6	C1	122.0(3)
**O3**	Nd01	O1	129.97(7)		N1	C17	P1	107.48(19)
**O3**	Nd01	O2	146.32(7)		C22	C19	P1	108.0(3)
**O2**	Nd01	I1	82.42(5)		C22	C19	C21	108.3(3)
**O2**	Nd01	P1	80.09(5)		C22	C19	C20	111.1(3)
**N1**	Nd01	I1	139.25(6)		C21	C19	P1	106.9(2)
**N1**	Nd01	P1	56.13(6)		C21	C19	C20	109.7(3)
**N1**	Nd01	O1	84.12(8)		C20	C19	P1	112.7(2)
**N1**	Nd01	O3	78.35(8)		O3	C31	C32	104.7(2)
**N1**	Nd01	O2	131.08(8)		C14	C13	C16	120.7(3)
**N2**	Nd01	I1	122.21(6)		C14	C13	C12	119.1(3)
**N2**	Nd01	P1	55.07(6)		C12	C13	C16	120.2(3)
**N2**	Nd01	O1	142.29(8)		O2	C30	C29	104.1(3)
**N2**	Nd01	O3	84.60(8)		C3	C2	C1	122.0(3)
**N2**	Nd01	O2	80.05(8)		N1	C1	C6	119.0(2)
**N2**	Nd01	N1	90.17(8)		N1	C1	C2	124.5(3)
**C18**	P1	Nd01	78.82(9)		C2	C1	C6	116.5(3)
**C18**	P1	C17	100.74(14)		C13	C14	C9	122.4(3)
**C18**	P1	C19	106.28(13)		C28	C29	C30	102.6(3)
**C17**	P1	Nd01	76.03(9)		O3	C34	C33	104.6(3)
**C17**	P1	C19	103.97(15)		C11	C10	C9	121.2(3)
**C19**	P1	Nd01	174.72(10)		C11	C12	C13	120.4(3)
**C23**	O1	Nd01	119.84(18)		N2	C9	C14	118.5(2)
**C23**	O1	C26	108.9(3)		N2	C9	C10	124.8(3)
**C26**	O1	Nd01	130.6(2)		C10	C9	C14	116.7(2)
**C31**	O3	Nd01	118.79(17)		C6	C5	C8	120.3(3)
**C34**	O3	Nd01	137.10(19)		C6	C5	C4	119.6(3)
**C34**	O3	C31	104.1(2)		C4	C5	C8	120.1(3)
**C27**	O2	Nd01	124.17(19)		C27	C28	C29	103.9(3)
**C30**	O2	Nd01	126.95(18)		C2	C3	C7	119.3(3)
**C30**	O2	C27	108.9(2)		C2	C3	C4	119.9(3)
**C17**	N1	Nd01	115.04(17)		C4	C3	C7	120.8(3)
**C1**	N1	Nd01	127.05(18)		O1	C26	C25	103.7(3)
**C1**	N1	C17	115.5(2)		C23	C24	C25	103.6(3)
**C18**	N2	Nd01	120.53(17)		C31	C32	C33	104.3(3)
**C9**	N2	Nd01	123.09(18)		C3	C4	C5	119.9(3)
**C9**	N2	C18	116.3(2)		C26	C25	C24	101.8(3)
**N2**	C18	P1	105.48(19)		C34	C33	C32	104.6(3)

3.5 NpI(^{Ar}BiAMP^{tBu})(thf)$_3$: 2527019

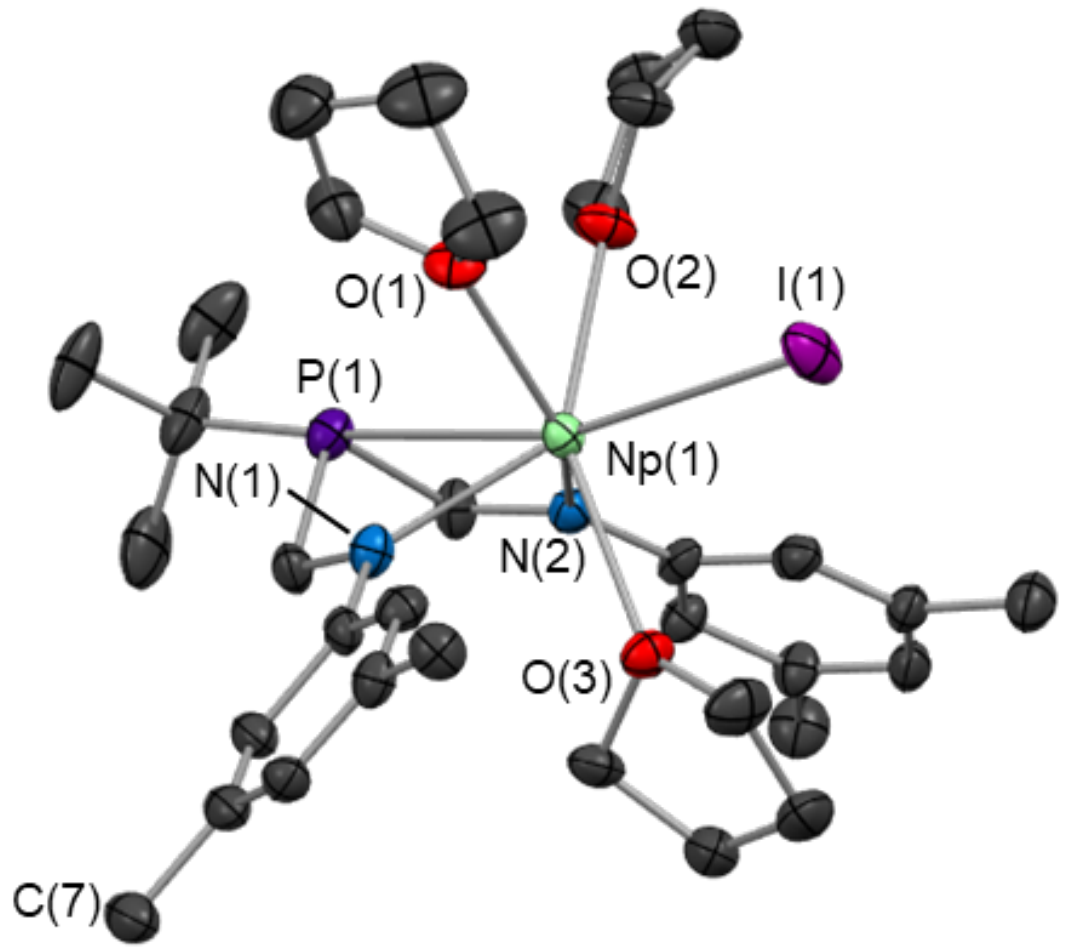

Table S10 Crystal data and structure refinement for NpI(^{Ar}BiAMP^{tBu})(thf)$_3$ (3).	
**Identification code**	NpBiAMP
**Empirical formula**	$C_{34}H_{55}IN_2NpO_3P$
**Formula weight**	934.67
**Temperature/K**	100.00
**Crystal system**	orthorhombic
**Space group**	$P2_12_12_1$
**a/Å**	13.362(3)
**b/Å**	14.516(2)
**c/Å**	19.120(3)
**α/°**	90
**β/°**	90
**γ/°**	90
**Volume/Å^3**	3708.7(12)
**Z**	4
**ρ_{calc}g/cm^3**	1.674
**μ/mm^{-1}**	3.710
**F(000)**	1832.0
**Crystal size/mm^3**	0.32 × 0.28 × 0.12
**Radiation**	MoKα (λ = 0.71073)
**2Θ range for data collection/°**	4.658 to 63.122
**Index ranges**	-19 ≤ h ≤ 19, -21 ≤ k ≤ 21, -28 ≤ l ≤ 28
**Reflections collected**	87074
**Independent reflections**	12408 [R_{int} = 0.1581, R_{sigma} = 0.0943]
**Data/restraints/parameters**	12408/0/387
**Goodness-of-fit on F^2**	1.080
**Final R indexes [I>=2σ (I)]**	R_1 = 0.0579, wR_2 = 0.1190
**Final R indexes [all data]**	R_1 = 0.0767, wR_2 = 0.1365
**Largest diff. peak/hole / e Å^{-3}**	1.97/-2.16
**Flack parameter**	0.022(16)

Table S11 Bond Lengths for NpI(^{Ar}BiAMP^{tBu})(thf)$_3$ (3).

tom	Ato m	Length/Å		Atom	Atom	Length/Å
**Np1**	I1	3.1708(10)		C19	C22	1.53(2)
**Np1**	P1	3.138(3)		C19	C20	1.554(16)
**Np1**	O1	2.526(8)		C19	C21	1.50(2)
**Np1**	O2	2.546(7)		C6	C5	1.369(16)
**Np1**	O3	2.498(8)		C6	C1	1.407(15)
**Np1**	N1	2.358(9)		C27	C28	1.504(18)
**Np1**	N2	2.352(9)		C5	C8	1.508(15)
**P1**	C17	1.851(11)		C5	C4	1.383(16)

**P1**	C19	1.862(13)		C16	C13	1.511(15)
**P1**	C18	1.848(11)		C14	C9	1.432(16)
**O1**	C23	1.428(17)		C14	C13	1.358(15)
**O1**	C26	1.426(15)		C2	C1	1.426(14)
**O2**	C27	1.443(14)		C2	C3	1.386(16)
**O2**	C30	1.448(13)		C30	C29	1.510(17)
**O3**	C31	1.466(13)		C31	C32	1.512(17)
**O3**	C34	1.435(14)		C29	C28	1.499(18)
**N1**	C17	1.455(14)		C7	C3	1.519(16)
**N1**	C1	1.382(14)		C4	C3	1.380(16)
**N2**	C18	1.452(14)		C26	C25	1.51(2)
**N2**	C9	1.394(13)		C9	C10	1.392(14)
**C23**	C24	1.570(17)		C24	C25	1.46(2)
**C11**	C15	1.512(15)		C13	C12	1.410(15)
**C11**	C10	1.382(15)		C34	C33	1.516(18)
**C11**	C12	1.391(16)		C32	C33	1.546(19)

Table S12 Bond Angles for NpI(^{Ar}BiAMP^{tBu})(thf)$_3$ (3).

**Atom**	**Atom**	**Atom**	**Angle/˚**		**Atom**	**Atom**	**Atom**	**Angle/˚**
**P1**	Np1	I1	162.65(6)		N1	C17	P1	107.6(7)
**O1**	Np1	I1	83.8(2)		C10	C11	C15	119.9(11)
**O1**	Np1	P1	90.1(2)		C10	C11	C12	121.0(10)
**O1**	Np1	O2	75.3(3)		C12	C11	C15	119.0(11)
**O2**	Np1	I1	83.05(18)		C22	C19	P1	108.1(10)
**O2**	Np1	P1	79.70(19)		C22	C19	C20	107.9(12)
**O3**	Np1	I1	81.06(19)		C20	C19	P1	106.8(8)
**O3**	Np1	P1	114.60(19)		C21	C19	P1	112.4(9)
**O3**	Np1	O1	132.4(3)		C21	C19	C22	111.2(12)
**O3**	Np1	O2	145.6(3)		C21	C19	C20	110.3(12)
**N1**	Np1	I1	138.6(2)		C5	C6	C1	123.0(11)
**N1**	Np1	P1	56.2(2)		O2	C27	C28	107.4(10)
**N1**	Np1	O1	84.2(3)		C6	C5	C8	121.1(11)
**N1**	Np1	O2	131.2(3)		C6	C5	C4	119.0(11)
**N1**	Np1	O3	78.3(3)		C4	C5	C8	119.9(10)
**N2**	Np1	I1	123.0(2)		N2	C18	P1	105.8(7)
**N2**	Np1	P1	55.3(2)		C13	C14	C9	121.5(11)
**N2**	Np1	O1	140.3(3)		C3	C2	C1	120.7(11)
**N2**	Np1	O2	79.4(3)		O2	C30	C29	103.9(10)
**N2**	Np1	O3	84.0(3)		O3	C31	C32	104.8(9)
**N2**	Np1	N1	90.1(3)		C28	C29	C30	103.9(11)
**C17**	P1	Np1	76.3(4)		C3	C4	C5	121.0(10)
**C17**	P1	C19	104.6(6)		O1	C26	C25	104.2(13)
**C19**	P1	Np1	174.6(4)		N1	C1	C6	119.9(10)
**C18**	P1	Np1	79.0(4)		N1	C1	C2	123.9(10)
**C18**	P1	C17	100.9(5)		C6	C1	C2	116.2(10)
**C18**	P1	C19	106.0(5)		N2	C9	C14	117.3(10)
**C23**	O1	Np1	121.6(7)		C10	C9	N2	125.4(10)
**C26**	O1	Np1	128.2(9)		C10	C9	C14	117.3(9)
**C26**	O1	C23	109.1(10)		C25	C24	C23	103.6(13)
**C27**	O2	Np1	124.6(7)		C29	C28	C27	103.8(10)
**C27**	O2	C30	108.9(9)		C11	C10	C9	121.1(10)
**C30**	O2	Np1	126.5(7)		C14	C13	C16	120.9(10)
**C31**	O3	Np1	119.0(6)		C14	C13	C12	120.4(11)
**C34**	O3	Np1	136.5(7)		C12	C13	C16	118.8(10)
**C34**	O3	C31	104.4(9)		C11	C12	C13	118.7(10)
**C17**	N1	Np1	114.5(6)		O3	C34	C33	106.0(10)
**C1**	N1	Np1	127.1(7)		C2	C3	C7	118.2(11)
**C1**	N1	C17	116.0(9)		C4	C3	C2	120.1(11)
**C18**	N2	Np1	119.8(7)		C4	C3	C7	121.7(11)
**C9**	N2	Np1	124.3(7)		C24	C25	C26	103.9(13)
**C9**	N2	C18	115.8(9)		C31	C32	C33	104.1(10)
**O1**	C23	C24	105.5(11)		C34	C33	C32	104.4(11)

3.6 PuI(^{Ar}BiAMP^{tBu})(thf)$_3$: 2527020

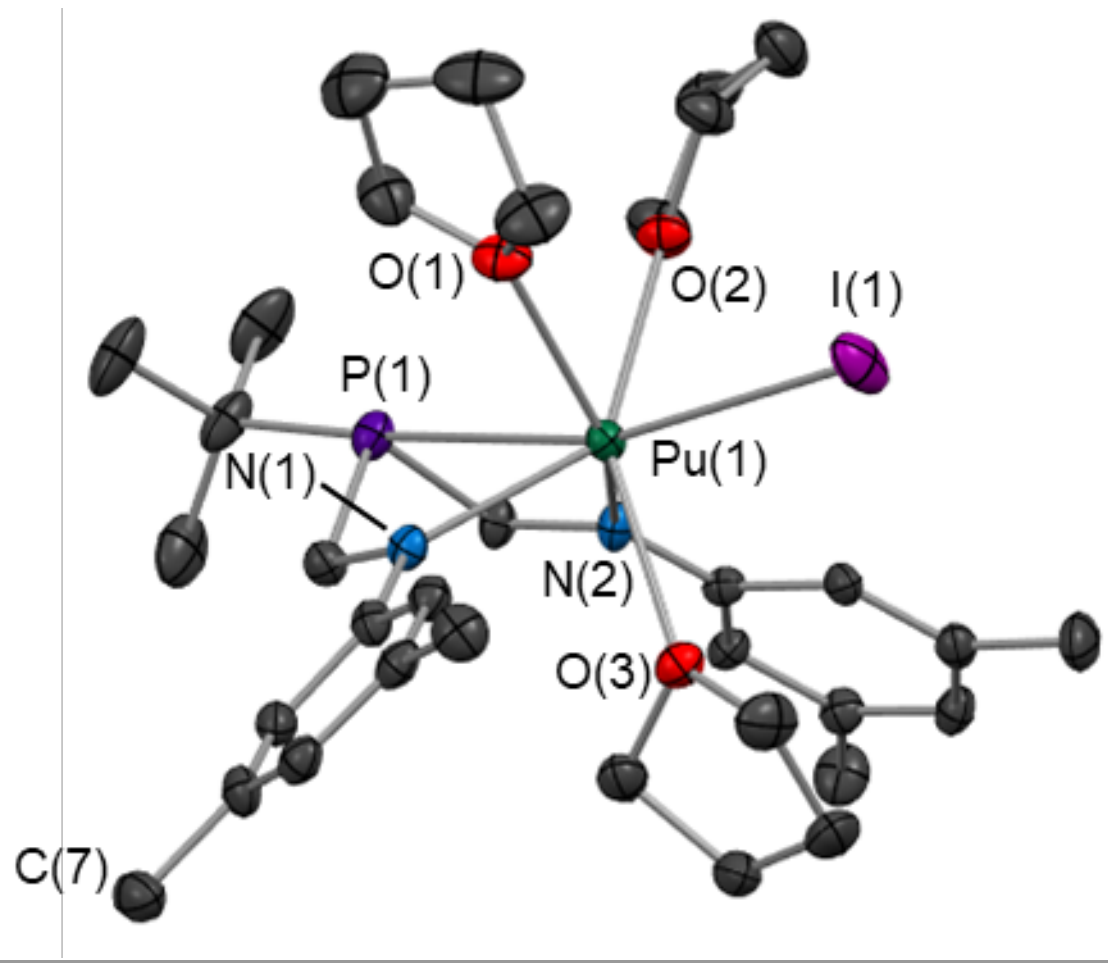

Table S13 Crystal data and structure refinement for PuI(^{Ar}BiAMP^{tBu})(thf)$_3$ (4).	
**Identification code**	PuBiAMP
**Empirical formula**	$C_{34}H_{55}IN_2O_3PPu$
**Formula weight**	939.67
**Temperature/K**	100.00
**Crystal system**	orthorhombic
**Space group**	$P2_12_12_1$
**a/Å**	13.341(3)
**b/Å**	14.487(3)
**c/Å**	19.107(4)
**α/°**	90
**β/°**	90
**γ/°**	90
**Volume/Å^3**	3693.0(14)
**Z**	4
**ρ_{calc}g/cm^3**	1.690
**μ/mm^{-1}**	2.697
**F(000)**	1836.0
**Crystal size/mm^3**	0.38 × 0.32 × 0.18
**Radiation**	MoKα (λ = 0.71073)
**2Θ range for data collection/°**	4.666 to 63.164
**Index ranges**	-19 ≤ h ≤ 19, -21 ≤ k ≤ 21, -28 ≤ l ≤ 28
**Reflections collected**	87792
**Independent reflections**	12328 [R_{int} = 0.1184, R_{sigma} = 0.0683]
**Data/restraints/parameters**	12328/0/387
**Goodness-of-fit on F^2**	1.057
**Final R indexes [I>=2σ (I)]**	R_1 = 0.0440, wR_2 = 0.0779
**Final R indexes [all data]**	R_1 = 0.0519, wR_2 = 0.0826
**Largest diff. peak/hole / e Å^{-3}**	2.10/-1.74
**Flack parameter**	0.007(12)

Table S14 Bond Lengths for PuI(^{Ar}BiAMP^{tBu})(thf)$_3$ (4).

Atom	Atom	Length/Å		Atom	Atom	Length/Å
**Pu1**	I1	3.1552(8)		C13	C16	1.504(10)
**Pu1**	P1	3.112(2)		C27	C28	1.501(12)
**Pu1**	O3	2.496(6)		C3	C7	1.502(11)
**Pu1**	O2	2.530(5)		C3	C4	1.382(11)
**Pu1**	N1	2.329(6)		N2	C18	1.464(9)
**Pu1**	O1	2.512(6)		N2	C9	1.380(9)
**Pu1**	N2	2.332(6)		C11	C10	1.399(10)
**P1**	C19	1.875(8)		C11	C15	1.500(10)
**P1**	C18	1.843(7)		C6	C1	1.414(11)

**P1**	C17	1.840(7)		C6	C5	1.376(11)
**O3**	C31	1.451(10)		C31	C32	1.533(11)
**O3**	C34	1.446(10)		C28	C29	1.512(12)
**O2**	C27	1.458(10)		C19	C22	1.532(14)
**O2**	C30	1.443(9)		C19	C20	1.500(13)
**C14**	C13	1.386(10)		C19	C21	1.547(11)
**C14**	C9	1.423(10)		C4	C5	1.394(11)
**N1**	C1	1.381(10)		C32	C33	1.532(13)
**N1**	C17	1.462(9)		C8	C5	1.497(11)
**C12**	C13	1.415(11)		C29	C30	1.497(12)
**C12**	C11	1.386(10)		C10	C9	1.401(10)
**O1**	C23	1.416(12)		C33	C34	1.504(12)
**O1**	C26	1.435(10)		C23	C24	1.547(12)
**C2**	C3	1.378(11)		C24	C25	1.485(16)
**C2**	C1	1.413(10)		C26	C25	1.506(14)

Table S15 Bond Angles for PuI(^{Ar}BiAMP^{tBu})(thf)$_3$ (4).

**Atom**	**Atom**	**Atom**	**Angle/˚**		**Atom**	**Atom**	**Atom**	**Angle/˚**
**P1**	Pu1	I1	162.68(4)		C14	C13	C16	120.4(7)
**O3**	Pu1	I1	81.18(13)		C12	C13	C16	120.4(7)
**O3**	Pu1	P1	114.45(13)		O2	C27	C28	107.3(7)
**O3**	Pu1	O2	145.04(18)		C2	C3	C7	119.0(7)
**O3**	Pu1	O1	132.17(18)		C2	C3	C4	119.9(8)
**O2**	Pu1	I1	82.44(12)		C4	C3	C7	121.2(8)
**O2**	Pu1	P1	80.34(13)		C18	N2	Pu1	119.1(4)
**N1**	Pu1	I1	138.03(15)		C9	N2	Pu1	124.8(5)
**N1**	Pu1	P1	56.74(15)		C9	N2	C18	116.0(6)
**N1**	Pu1	O3	77.76(19)		C12	C11	C10	119.7(7)
**N1**	Pu1	O2	132.46(18)		C12	C11	C15	120.1(7)
**N1**	Pu1	O1	84.2(2)		C10	C11	C15	120.2(7)
**N1**	Pu1	N2	90.7(2)		C5	C6	C1	123.9(8)
**O1**	Pu1	I1	83.74(15)		O3	C31	C32	104.2(6)
**O1**	Pu1	P1	90.20(15)		C27	C28	C29	103.3(7)
**O1**	Pu1	O2	75.88(19)		C22	C19	P1	107.2(7)
**N2**	Pu1	I1	122.44(14)		C22	C19	C21	107.9(8)
**N2**	Pu1	P1	55.98(15)		C20	C19	P1	113.0(6)
**N2**	Pu1	O3	83.35(19)		C20	C19	C22	111.3(8)
**N2**	Pu1	O2	79.8(2)		C20	C19	C21	111.0(9)
**N2**	Pu1	O1	141.2(2)		C21	C19	P1	106.2(5)
**C19**	P1	Pu1	175.0(3)		C3	C4	C5	121.2(8)
**C18**	P1	Pu1	79.0(2)		N1	C1	C2	125.2(7)
**C18**	P1	C19	105.7(3)		N1	C1	C6	119.3(7)
**C17**	P1	Pu1	76.5(3)		C2	C1	C6	115.4(7)
**C17**	P1	C19	103.7(4)		N2	C18	P1	105.8(5)
**C17**	P1	C18	100.8(4)		C33	C32	C31	104.0(7)
**C31**	O3	Pu1	119.1(4)		N1	C17	P1	107.7(5)
**C34**	O3	Pu1	136.7(5)		C30	C29	C28	103.8(7)
**C34**	O3	C31	104.1(6)		C6	C5	C4	117.6(7)
**C27**	O2	Pu1	124.4(5)		C6	C5	C8	122.0(8)
**C30**	O2	Pu1	127.0(5)		C4	C5	C8	120.3(7)
**C30**	O2	C27	108.6(6)		C11	C10	C9	122.5(7)
**C13**	C14	C9	122.4(7)		C34	C33	C32	104.7(7)
**C1**	N1	Pu1	128.4(5)		N2	C9	C14	118.0(6)
**C1**	N1	C17	115.2(6)		N2	C9	C10	125.8(7)
**C17**	N1	Pu1	114.5(4)		C10	C9	C14	116.2(7)
**C11**	C12	C13	120.0(7)		O3	C34	C33	105.1(7)
**C23**	O1	Pu1	121.8(5)		O1	C23	C24	106.5(8)
**C23**	O1	C26	109.2(7)		O2	C30	C29	104.3(7)
**C26**	O1	Pu1	128.1(5)		C25	C24	C23	103.2(9)
**C3**	C2	C1	121.9(8)		O1	C26	C25	103.7(8)
**C14**	C13	C12	119.2(7)		C24	C25	C26	103.5(9)

3.7 PrI(^{Ar}BiAMP^{tBu})(thf)$_3$: 2527021

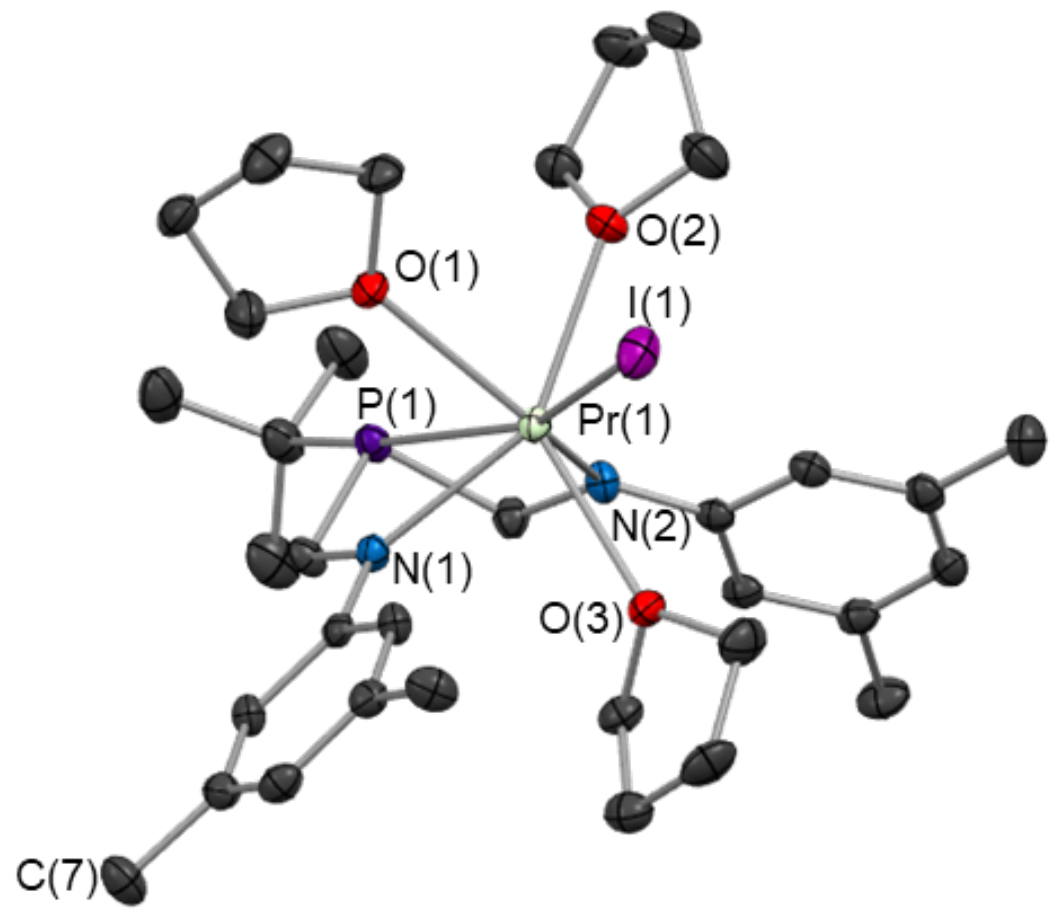

Table S16 Crystal data and structure refinement for PrI(^{Ar}BiAMP^{tBu})(thf)$_3$.	
**Identification code**	PrBiAMP
**Empirical formula**	$C_{34}H_{55}N_2O_3PIPr$
**Formula weight**	838.58
**Temperature/K**	100.00
**Crystal system**	orthorhombic
**Space group**	$P2_12_12_1$
**a/Å**	13.347(3)
**b/Å**	14.497(3)
**c/Å**	19.150(5)
**α/°**	90
**β/°**	90
**γ/°**	90
**Volume/Å^3**	3705.4(15)
**Z**	4
**ρ_{calc}g/cm^3**	1.503
**μ/mm^{-1}**	2.224
**F(000)**	1696.0
**Crystal size/mm^3**	0.2 × 0.18 × 0.06
**Radiation**	MoKα (λ = 0.71073)
**2Θ range for data collection/°**	4.148 to 63.278
**Index ranges**	-19 ≤ h ≤ 19, -21 ≤ k ≤ 21, -28 ≤ l ≤ 28
**Reflections collected**	86025
**Independent reflections**	12405 [R_{int} = 0.0975, R_{sigma} = 0.0609]
**Data/restraints/parameters**	12405/0/386
**Goodness-of-fit on F^2**	1.088
**Final R indexes [I>=2σ (I)]**	R_1 = 0.0386, wR_2 = 0.0725
**Final R indexes [all data]**	R_1 = 0.0488, wR_2 = 0.0781
**Largest diff. peak/hole / e Å^{-3}**	1.03/-0.87
**Flack parameter**	-0.017(9)

Table S17 Bond Lengths for PrI(^{Ar}BiAMP^{tBu})(thf)$_3$.

Atom	Atom	Length/Å		Atom	Atom	Length/Å
**Pr1**	I1	3.1902(7)		C19	C21	1.529(9)
**Pr1**	P1	3.1539(14)		C19	C20	1.518(8)
**Pr1**	O3	2.503(4)		C19	C22	1.539(7)
**Pr1**	O1	2.534(3)		C23	C24	1.507(8)
**Pr1**	O2	2.519(4)		C3	C4	1.387(7)
**Pr1**	N1	2.363(4)		C3	C2	1.396(7)
**Pr1**	N2	2.354(4)		C3	C7	1.518(7)
**P1**	C18	1.857(5)		C31	C32	1.516(7)
**P1**	C17	1.846(5)		C1	C2	1.411(7)
**P1**	C19	1.873(6)		C32	C33	1.530(8)

**O3**	C31	1.452(6)		C27	C28	1.533(7)
**O3**	C34	1.441(6)		C15	C11	1.508(7)
**O1**	C23	1.456(7)		C10	C9	1.406(7)
**O1**	C26	1.464(6)		C10	C11	1.388(7)
**O2**	C27	1.447(7)		C25	C26	1.507(8)
**O2**	C30	1.445(7)		C25	C24	1.510(8)
**N1**	C17	1.449(6)		C14	C9	1.406(7)
**N1**	C1	1.390(6)		C14	C13	1.377(7)
**N2**	C18	1.452(6)		C16	C13	1.510(7)
**N2**	C9	1.380(6)		C34	C33	1.524(8)
**C6**	C5	1.369(7)		C28	C29	1.518(10)
**C6**	C1	1.421(7)		C11	C12	1.386(8)
**C5**	C4	1.398(7)		C29	C30	1.515(9)
**C5**	C8	1.515(7)		C12	C13	1.404(7)

Table S18 Bond Angles for PrI(^{Ar}BiAMP^{tBu})(thf)$_3$.

Atom	Atom	Atom	Angle/˚		Atom	Atom	Atom	Angle/˚
**P1**	Pr1	I1	162.72(3)		C6	C5	C4	119.6(5)
**O3**	Pr1	I1	80.74(8)		C6	C5	C8	120.7(5)
**O3**	Pr1	P1	114.31(9)		C4	C5	C8	119.6(5)
**O3**	Pr1	O1	145.88(12)		N2	C18	P1	107.7(3)
**O3**	Pr1	O2	130.33(12)		N1	C17	P1	105.6(3)
**O1**	Pr1	I1	82.39(8)		C21	C19	P1	107.7(4)
**O1**	Pr1	P1	80.34(9)		C21	C19	C22	108.9(5)
**O2**	Pr1	I1	83.32(9)		C20	C19	P1	112.9(4)
**O2**	Pr1	P1	92.13(9)		C20	C19	C21	110.4(5)
**O2**	Pr1	O1	76.27(12)		C20	C19	C22	110.3(5)
**N1**	Pr1	I1	122.14(10)		C22	C19	P1	106.6(4)
**N1**	Pr1	P1	54.84(10)		O1	C23	C24	107.1(5)
**N1**	Pr1	O3	84.43(13)		C4	C3	C2	120.7(5)
**N1**	Pr1	O1	79.82(14)		C4	C3	C7	120.0(5)
**N1**	Pr1	O2	142.09(14)		C2	C3	C7	119.3(5)
**N2**	Pr1	I1	139.33(10)		O3	C31	C32	104.5(4)
**N2**	Pr1	P1	56.07(10)		N1	C1	C6	118.6(4)
**N2**	Pr1	O3	78.03(13)		N1	C1	C2	124.7(5)
**N2**	Pr1	O1	131.54(13)		C2	C1	C6	116.7(5)
**N2**	Pr1	O2	84.57(14)		C3	C4	C5	119.7(5)
**N2**	Pr1	N1	89.85(15)		C31	C32	C33	104.8(4)
**C18**	P1	Pr1	75.86(17)		O2	C27	C28	106.5(5)
**C18**	P1	C19	104.1(3)		C3	C2	C1	120.7(5)
**C17**	P1	Pr1	79.07(16)		C11	C10	C9	121.5(5)
**C17**	P1	C18	100.7(2)		C26	C25	C24	103.1(5)
**C17**	P1	C19	106.1(2)		O1	C26	C25	104.1(4)
**C19**	P1	Pr1	174.68(18)		C13	C14	C9	122.6(5)
**C31**	O3	Pr1	118.9(3)		O3	C34	C33	104.4(4)
**C34**	O3	Pr1	136.9(3)		C23	C24	C25	103.9(5)
**C34**	O3	C31	104.2(4)		C29	C28	C27	103.0(5)
**C23**	O1	Pr1	124.5(3)		N2	C9	C10	124.4(5)
**C23**	O1	C26	108.3(4)		N2	C9	C14	118.7(5)
**C26**	O1	Pr1	127.2(3)		C14	C9	C10	116.9(5)
**C27**	O2	Pr1	120.1(3)		C10	C11	C15	119.6(5)
**C30**	O2	Pr1	130.4(3)		C12	C11	C15	120.6(5)
**C30**	O2	C27	108.9(4)		C12	C11	C10	119.8(5)
**C17**	N1	Pr1	120.4(3)		C30	C29	C28	102.3(5)
**C1**	N1	Pr1	122.9(3)		C11	C12	C13	120.3(5)
**C1**	N1	C17	116.7(4)		C34	C33	C32	103.9(4)
**C18**	N2	Pr1	114.9(3)		O2	C30	C29	103.6(5)
**C9**	N2	Pr1	127.2(3)		C14	C13	C16	122.0(5)
**C9**	N2	C18	115.4(4)		C14	C13	C12	118.9(5)
**C5**	C6	C1	122.5(5)		C12	C13	C16	119.1(5)

3.8 TmI(^{Ar}BiAMP^{tBu})(thf)$_3$: 2527022

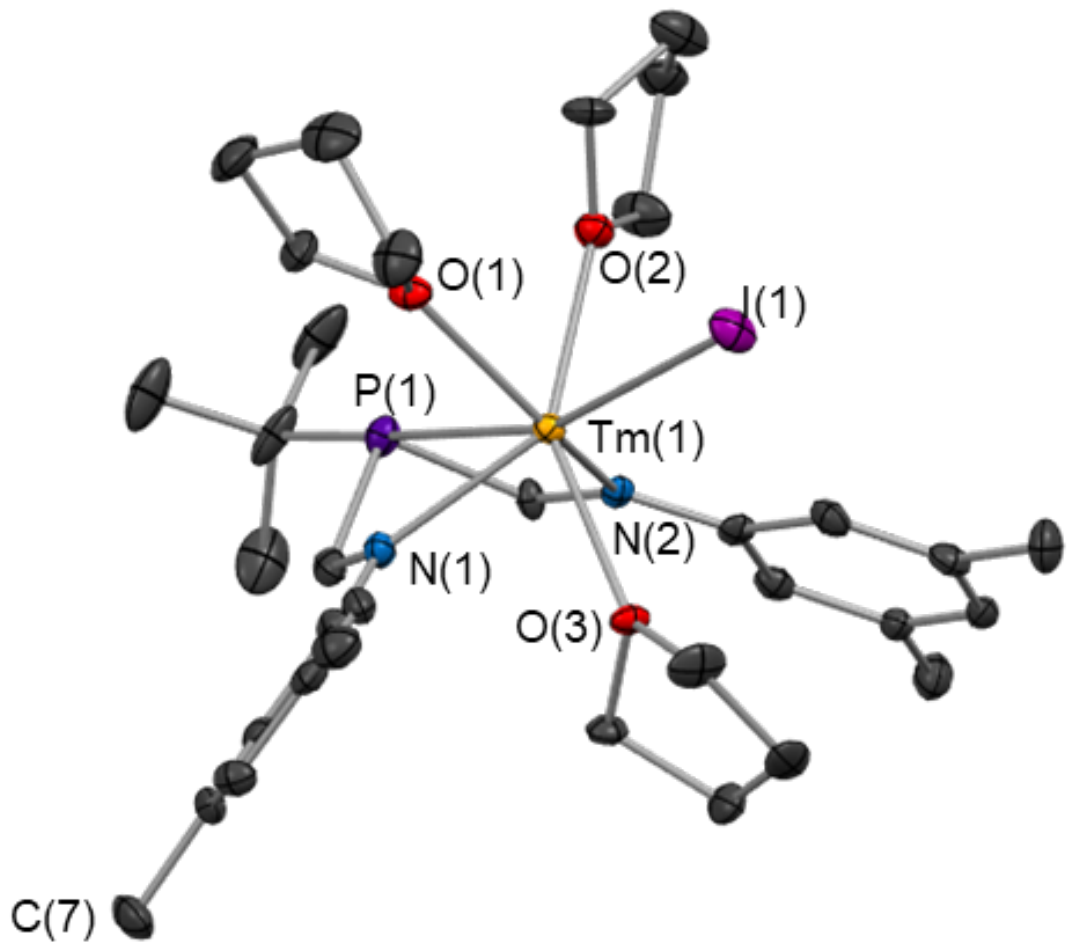

Table S19 Crystal data and structure refinement for TmI(^{Ar}BiAMP^{tBu})(thf)$_3$.	
**Identification code**	TmBiAMP
**Empirical formula**	$C_{34}H_{55}IN_2O_3PTm$
**Formula weight**	866.60
**Temperature/K**	100.00
**Crystal system**	orthorhombic
**Space group**	$P2_12_12_1$
**a/Å**	13.396(3)
**b/Å**	14.222(3)
**c/Å**	19.090(4)
**α/°**	90
**β/°**	90
**γ/°**	90
**Volume/Å^3**	3636.7(13)
**Z**	4
**ρ_{calc}g/cm^3**	1.583
**μ/mm^{-1}**	3.366
**F(000)**	1736.0
**Crystal size/mm^3**	0.17 × 0.17 × 0.1
**Radiation**	MoKα (λ = 0.71073)
**2Θ range for data collection/°**	5.14 to 58.048
**Index ranges**	-18 ≤ h ≤ 18, -19 ≤ k ≤ 19, -25 ≤ l ≤ 26
**Reflections collected**	62523
**Independent reflections**	9578 [R_{int} = 0.1098, R_{sigma} = 0.0761]
**Data/restraints/parameters**	9578/0/388
**Goodness-of-fit on F^2**	1.050
**Final R indexes [I>=2σ (I)]**	R_1 = 0.0413, wR_2 = 0.0701
**Final R indexes [all data]**	R_1 = 0.0594, wR_2 = 0.0744
**Largest diff. peak/hole / e Å^{-3}**	2.14/-0.86
**Flack parameter**	0.177(11)

Table S20 Bond Lengths for TmI(^{Ar}BiAMP^{tBu})(thf)$_3$.

Atom	Atom	Length/Å		Atom	Atom	Length/Å
**Tm1**	I1	3.0674(8)		C6	C5	1.400(11)
**Tm1**	P1	3.079(2)		C11	C10	1.399(11)
**Tm1**	O3	2.357(5)		C11	C15	1.510(11)
**Tm1**	O1	2.383(5)		C11	C12	1.366(11)
**Tm1**	N2	2.254(6)		C14	C13	1.374(11)
**Tm1**	N1	2.252(6)		C14	C9	1.427(11)
**Tm1**	O2	2.398(5)		C10	C9	1.433(10)
**P1**	C17	1.839(7)		C13	C12	1.405(11)
**P1**	C19	1.868(9)		C13	C16	1.513(11)

**P1**	C18	1.848(8)		C31	C32	1.519(11)
**O3**	C31	1.455(9)		C2	C1	1.422(11)
**O3**	C34	1.450(9)		C2	C3	1.382(11)
**O1**	C26	1.451(9)		C26	C25	1.498(13)
**O1**	C23	1.449(10)		C5	C4	1.395(11)
**N2**	C18	1.459(9)		C5	C8	1.495(11)
**N2**	C9	1.375(10)		C4	C3	1.387(11)
**N1**	C17	1.454(10)		C34	C33	1.522(12)
**N1**	C1	1.373(10)		C3	C7	1.511(10)
**O2**	C27	1.450(10)		C23	C24	1.542(11)
**O2**	C30	1.450(9)		C32	C33	1.523(12)
**C19**	C20	1.514(13)		C24	C25	1.507(14)
**C19**	C22	1.538(13)		C27	C28	1.505(12)
**C19**	C21	1.528(11)		C29	C30	1.503(11)
**C6**	C1	1.397(11)		C29	C28	1.505(12)

Table S21 Bond Angles for TmI(^{Ar}BiAMP^{tBu})(thf)$_3$.

Atom	Atom	Atom	Angle/˚		Atom	Atom	Atom	Angle/˚
**I1**	Tm1	P1	159.81(4)		C20	C19	P1	113.2(6)
**O3**	Tm1	I1	81.56(13)		C20	C19	C22	111.3(8)
**O3**	Tm1	P1	116.41(13)		C20	C19	C21	110.0(8)
**O3**	Tm1	O1	128.65(18)		C22	C19	P1	107.5(7)
**O3**	Tm1	O2	146.43(18)		C21	C19	P1	106.4(6)
**O1**	Tm1	I1	82.44(13)		C21	C19	C22	108.3(8)
**O1**	Tm	P1	91.96(14)		C1	C6	C5	123.3(7)
**O1**	Tm1	O2	76.46(19)		C10	C11	C15	118.9(7)
**N2**	Tm1	I1	120.23(16)		C12	C11	C10	120.5(7)
**N2**	Tm1	P1	57.13(16)		C12	C11	C15	120.5(7)
**N2**	Tm1	O3	83.8(2)		N2	C18	P1	106.0(5)
**N2**	Tm1	O1	144.8(2)		C13	C14	C9	121.9(7)
**N2**	Tm1	O2	81.1(2)		C11	C10	C9	121.0(7)
**N1**	Tm1	I1	139.81(17)		C14	C13	C12	120.0(7)
**N1**	Tm1	P1	57.60(16)		C14	C13	C16	120.1(7)
**N1**	Tm1	O3	78.9(2)		C12	C13	C16	119.9(7)
**N1**	Tm1	O1	83.0(2)		N2	C9	C14	119.4(7)
**N1**	Tm1	N2	92.1(2)		N2	C9	C10	124.4(7)
**N1**	Tm1	O2	131.32(19)		C14	C9	C10	116.2(7)
**O2**	Tm1	I1	80.49(13)		O3	C31	C32	104.7(6)
**O2**	Tm1	P1	79.35(13)		C3	C2	C1	121.4(8)
**C17**	P1	Tm1	75.3(3)		O1	C26	C25	103.4(7)
**C17**	P1	C19	104.2(4)		C11	C12	C13	120.5(7)
**C17**	P1	C18	100.2(4)		N1	C1	C6	119.5(7)
**C19**	P1	Tm1	176.4(3)		N1	C1	C2	124.5(7)
**C18**	P1	Tm1	77.4(2)		C6	C1	C2	116.1(7)
**C18**	P1	C19	106.1(4)		C6	C5	C8	121.6(7)
**C31**	O3	Tm1	119.0(4)		C4	C5	C6	118.5(8)
**C34**	O3	Tm1	137.1(5)		C4	C5	C8	120.0(7)
**C34**	O3	C31	103.9(6)		C3	C4	C5	120.0(8)
**C26**	O1	Tm1	132.2(5)		O3	C34	C33	104.4(6)
**C23**	O1	Tm1	119.6(4)		C2	C3	C4	120.8(8)
**C23**	O1	C26	106.9(6)		C2	C3	C7	118.5(8)
**C18**	N2	Tm1	119.3(5)		C4	C3	C7	120.7(7)
**C9**	N2	Tm1	125.4(5)		O1	C23	C24	106.8(7)
**C9**	N2	C18	115.3(6)		C31	C32	C33	104.6(6)
**C17**	N1	Tm1	115.3(4)		C25	C24	C23	102.9(8)
**C1**	N1	Tm1	127.9(5)		O2	C27	C28	106.6(7)
**C1**	N1	C17	115.3(6)		C30	C29	C28	103.4(7)
**C27**	O2	Tm1	123.6(5)		O2	C30	C29	103.7(7)
**C27**	O2	C30	109.3(6)		C26	C25	C24	102.9(8)
**C30**	O2	Tm1	127.0(4)		C34	C33	C32	104.6(7)
**N1**	C17	P1	107.7(5)		C27	C28	C29	102.4(7)

4. References

[64] C. A. P. Goodwin, M. T. Janicke, B. L. Scott, A. J. Gaunt, "[$AnI_3(THF)_4$] (An = Np, Pu) Preparation Bypassing An^0 Metal Precursors: Access to Np^{3+}/Pu^{3+} Nonaqueous and Organometallic Complexes," *Journal of the American Chemical Society* 143, (2021): 20680. https://doi.org/10.1021/jacs.1c07967

[73] B. Conway, D. V. Graham, E. Hevia, A. R. Kennedy, J. Klett, R. E. Mulvey, "Structurally-defined potassium-mediated regioselective zincation of amino- and alkoxy-substituted pyridines," *Chemical Communications* 23, (2008): 2638–2640. https://doi.org/10.1039/B805606D

[49] Y. Minko, T. V. Fetrow, S. Sharma, B. K. Cashman, A. M. Tondreau, "Flexible Interactions of the Rare-Earth Elements Y, La, and Lu with a Phosphine-Bearing Diamine Ligand," *Chemical Science* 15, (2024): 12138. https://doi.org/10.1039/D4SC02077D

[74] Bruker Instrument Service, Version 2010.1.0.0; Bruker AXS Inc.: Madison, WI, USA, **2010**.

[75] G. M. Sheldrick, "SHELXT - Integrated Space-Group and Crystal-Structure Determination," *Acta Crystallographica Section A: Foundations Advances* A71, (2015): 3. https://doi.org/10.1107/S2053273314026370.

[76] G. M. Sheldrick, SADABS, University of Göttingen, Germany: 2005.

[77] G. M. Sheldrick, "Crystal Structure Refinement with SHELXL," *Acta Crystallographica Section C: Structural Chemistry* C71, (2015): 3. https://doi.org/10.1107/S2053229614024218.

[78] APEX3, Version 2019, Bruker Nano, Inc.; Madison, WI, USA, 2019.

[79] Olex2 1.2 (compiled 2014.06.27 svn.r2953 for OlexSys, GUI svn.r4855).

[80] O. V. Dolomanov, R. J. Gildea, J. A. K. Howard, H. Puschmann, "*OLEX2*: A Complete Structure Solution, Refinement and Analysis Program," *Journal of Applied Crystallography* 42, (2009): 339. https://doi.org/10.1107/S0021889808042726.
